# Disparities in efficacy and safety of sodium-glucose cotransporter 2 inhibitor among patients with different extents of renal dysfunction: A systematic review and meta-analysis of randomized controlled trials

**DOI:** 10.3389/fphar.2022.1018720

**Published:** 2022-11-22

**Authors:** Suiyuan Hu, Chu Lin, Xiaoling Cai, Xingyun Zhu, Fang Lv, Wenjia Yang, Linong Ji

**Affiliations:** Department of Endocrinology and Metabolism, Peking University People’s Hospital, Beijing, China

**Keywords:** renal function impairment, sodium-glucose cotransporter 2 (SGLT2) inhibitor, blood glucose, weight, blood pressure, estimated glomerular alteration rate (eGFR)

## Abstract

**Background:** The pleiotropic efficacy of SGLT2is in patients with different eGFR levels has not been well-understood. This systematic review and meta-analysis assessed the disparities in the efficacy and safety of SGLT2i treatment across stratified renal function.

**Methods:** We searched four databases from inception to December 2021. We included randomized controlled trials (RCTs) with reported baseline eGFR levels and absolute changes from baseline in at least one of the following outcomes: HbA1c, body weight, blood pressure, and eGFR. Continuous outcomes were evaluated as the weighted mean differences (WMDs) and 95% confidence intervals (CIs). Categorical outcomes were evaluated as odds ratios (ORs) and accompanying 95% CIs.

**Results:** In total, 86 eligible RCTs were included. SGLT2is produces a substantial benefit in glycemic control, weight control, and blood pressure control even in patients with impaired renal function. HbA1c and weight reductions observed in SGLT2i users were generally parallel with the renal function levels, although there was an augmented weight reduction in severe renal dysfunction stratum [HbA1c: −0.49% (−0.58 to −0.39%) for normal renal function, −0.58% (−0.66 to −0.50%) for mild renal function impairment, −0.22% (−0.35 to −0.09%) for moderate renal function impairment, and −0.13% (−0.67 to 0.42%) for severe renal function impairment (*p* < 0.001 for subgroup differences); weight: −2.12 kg (−2.66 to −1.59 kg) for normal renal function, −2.06 kg (−2.31 to −1.82 kg) for mild renal function impairment; −1.23 kg (−1.59 to −0.86 kg) for moderate renal function impairment; −1.88 kg (−3.04 to −0.72 kg) for severe renal function impairment (*p* = 0.002 for subgroup differences)]. However, the blood pressure reduction observed in SGLT2i users was independent of renal function. When compared with the placebo, the occurrence of hypoglycemia was more frequent in patients with favorable renal function rather than in those with substantial renal dysfunction.

**Conclusion:** The HbA1c and body weight reductions observed in SGLT2i users were generally parallel with their baseline eGFR levels, while blood pressure reductions in SGLT2i users were independent of their baseline eGFR levels. Consistently, when compared with the placebo, hypoglycemia was more frequent in patients with favorable renal function, where the HbA1c reduction was profound.

## Introduction

Sodium-glucose cotransporter 2 inhibitors (SGLT2is) have gained extensive attention in recent years as a novel type of anti-hyperglycemic drugs due to their additional cardiovascular and renal benefits beyond blood glucose control (Nair and Wilding, 2010; Bailey, 2011). SGLT2is exert their function by blocking SGLT2, which plays an important role in glucose reabsorption (Vallon and Thomson, 2017).

Available evidence showed that there was a significant difference in urinary glucose excretion (UGE) induced by SGLT2i among patients with different levels of renal function. UGE gradually decreased with worsening renal impairment, indicated by a reduction in the estimated glomerular filtration rate (eGFR). Therefore, it is important to explore whether the benefits of SGLT2 inhibition on blood glucose control, weight and blood pressure reduction, and eGFR preservation are fairly comparable in patients with impaired renal function to those with normal renal function. However, SGLT2is are generally contraindicated in patients with severe renal impairment (eGFR <30 ml/min per 1.73 m^2^) due to concerns that SGLT2 inhibition may increase the risk of acute kidney injury (Levey et al., 2011; Scheen, 2015; Zhang et al., 2018; Davidson, 2019). Few studies have assessed the exact role of SGLT2is in patients with different baseline renal function. However, the pleiotropic efficacy and safety outcomes of SGLT2is in patients with different eGFR levels have not been well-characterized. In this systematic review and meta-analysis, we aimed to assess the similarities and disparities regarding the efficacy and safety of SGLT2is across stratified renal function.

## Material and methods

### Data sources and searches

Conforming to the recommendations from the Cochrane Handbook for Systematic Reviews for meta-analysis, we conducted systematic searches manually in PubMed, Medline, Embase, and Cochrane Central Register of Controlled Trials (CENTRAL) databases. The systematic database research was first conducted in May 2021 and updated in December 2021. We used the following medical subject headings and free-text search terms: SGLT2 inhibitors, canagliflozin, dapagliflozin, empagliflozin, ertugliflozin, ipragliflozin, luseogliflozin, remogliflozin, sotagliflozin, tofogliflozin, and randomized controlled trial (RCT). We also screened references of existing reviews in this field in order to identify every possibly eligible relevant study.

### Study selection

Studies were included if they met the following criteria: 1) RCTs of SGLT2i; 2) RCTs with reported baseline eGFR levels and absolute changes from baseline in at least one of the following outcomes: glycated hemoglobin (HbA1c) levels, body weight, blood pressure, and eGFR; 3) studies published in English. There were no restrictions on the length of the follow‐up. Two investigators (CL and SH) independently browsed the titles, abstracts, full texts, and supplementary materials of potentially eligible studies. Any disagreements were resolved by consensus with a third investigator (XZ).

### Data extraction and quality assessment

Two investigators (CL and SH) used predefined forms to record data from eligible studies, including study characteristics (first author, publication year, study design, sample size, and mean duration of follow-up), participant characteristics (age, sex, duration of diabetes, baseline eGFR, baseline HbA1c level, blood pressure, and body weight), therapeutic intervention (subtypes of SGLT2i and dosages), comparison groups (placebo or active agent control), and outcomes of interest (changes in HbA1c level, body weight, blood pressure, and eGFR in treatment and control groups). Adverse events such as urinary tract infection, genital infection, amputation, hypovolemia, orthostatic hypotension, bone fracture, diabetic ketoacidosis and hypoglycemia were also collected for evaluations of safety as additional outcomes. Study quality was evaluated by using the Cochrane risk of bias tool. A third investigator (FL) checked for the accuracy of the abstractions and study quality evaluation. Any disagreement among investigators would be resolved by consensus.

### Data synthesis and analysis

The efficacy outcomes included changes in HbA1c, body weight, systolic and dilated blood pressure, and eGFR. The safety outcomes included the incidence of urinary tract infection, genital infection, amputation, hypovolemia, orthostatic hypotension, bone fracture, diabetic ketoacidosis, and hypoglycemia. Continuous outcomes were evaluated as the weighted mean differences (WMDs) and 95% confidence intervals (CIs). Categorical outcomes were evaluated as odds ratios (ORs) and accompanying 95% CIs. The degree of between‐study heterogeneity was evaluated through Higgins I^2^ statistics. An I^2^ level more than 50% was considered a high level of heterogeneity. A fixed-effect model was used when I^2^ <50%, and a random-effect model was used when I^2^ ≥ 50%. Data were represented graphically in forest plots. Publication bias was assessed using funnel plots.

Subgroup analyses were conducted based on baseline eGFR levels. We divided the enrolled patients into four subgroups with the cut-off values at 90, 60, and 45 ml/min per 1.73 m^2^: normal renal function, defined as eGFR ≥90 ml/min/1.73 m^2^; mild renal function impairment, defined as 90 > eGFR ≥60 ml/min per 1.73 m^2^; moderate renal function impairment, defined as 60 > eGFR ≥45 ml/min per 1.73 m^2^; severe renal function impairment, defined as eGFR<45 ml/min per 1.73 m^2^. Meta-analyses were performed by the Review Manager statistical package (version 5.3, Nordic Cochrane Centre, Copenhagen, Denmark) and STATA, version 11.0 (STATA, College Station, TX, United States). A *p*-value less than 0.05 was considered statistically significant for all analyses. This meta-analysis was registered on the PROSPERO platform as CRD42022297648.

## Results

### Characteristics of included studies

**FIGURE 1:**
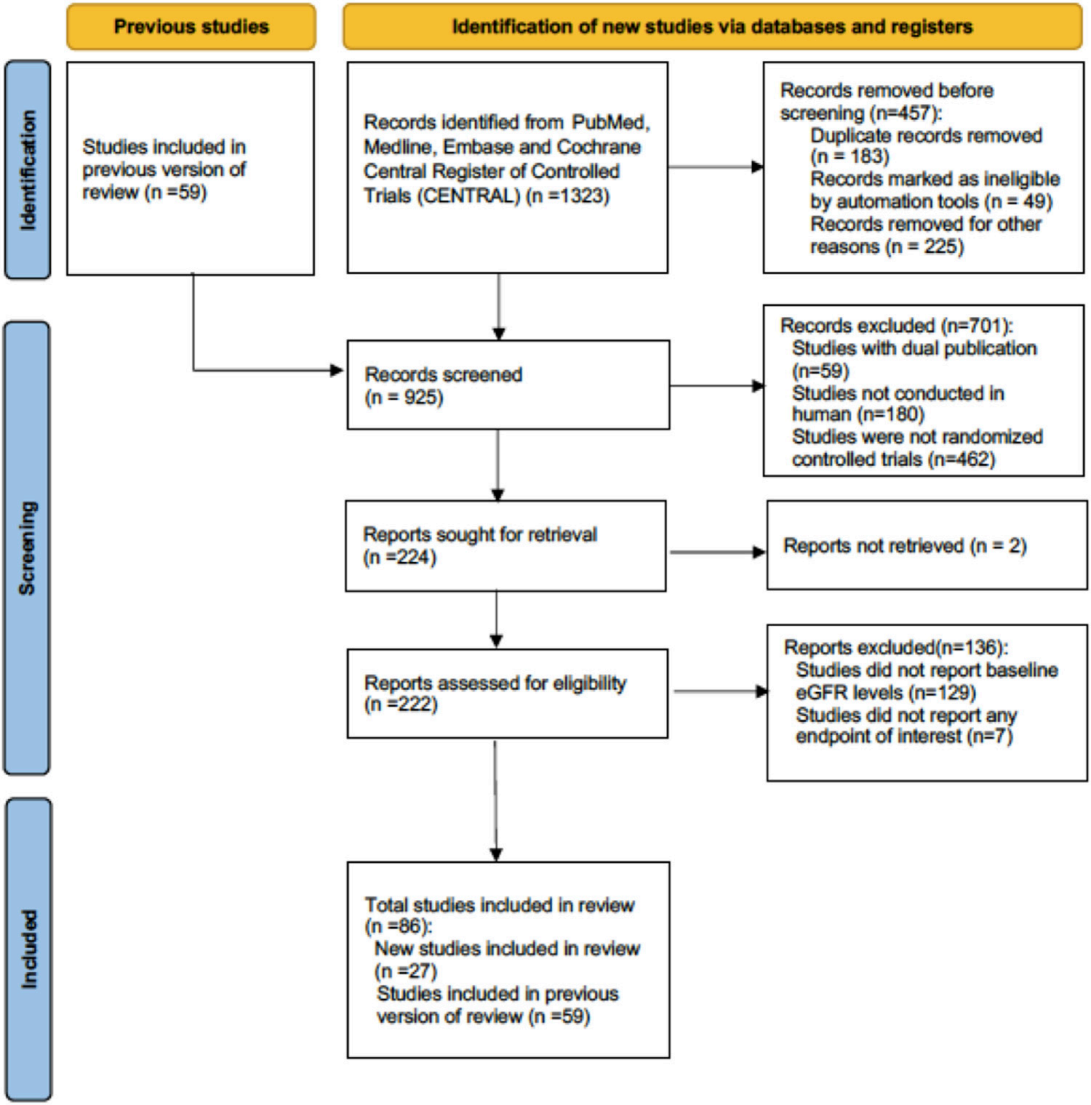
Flowchart of the included studies.

A total of 86 RCTs were included, with 72 placebo-controlled studies and 14 active agent-controlled studies (Figure 1). Eight types of SGLT2is, namely, canagliflozin, dapagliflozin, empagliflozin, ertugliflozin, ipragliflozin, luseogliflozin, sotagliflozin, and tofogliflozin, were assessed. The trial durations ranged from 4 to 135 weeks. Among all included studies, the mean baseline the eGFR ranged from 22.00 to 154.48 ml/min per 1.73 m^2^. The numbers of patients with normal renal function, mild renal impairment, moderate renal impairment, and severe renal impairment were 12,069, 37,533, 1,642, and 15,477, respectively. Baseline characteristics of included studies are summarized in Supplementary Table S1. The risk of bias for RCTs was systematically evaluated by the Cochrane tool, and the overall risk of bias and selective reporting was low (Supplementary Table S2). The funnel plots generally displayed even distributions, which indicated no signs of publication bias (Supplementary Figure S1).

### Effects of SGLT2is on HbA1c

**FIGURE 2:**
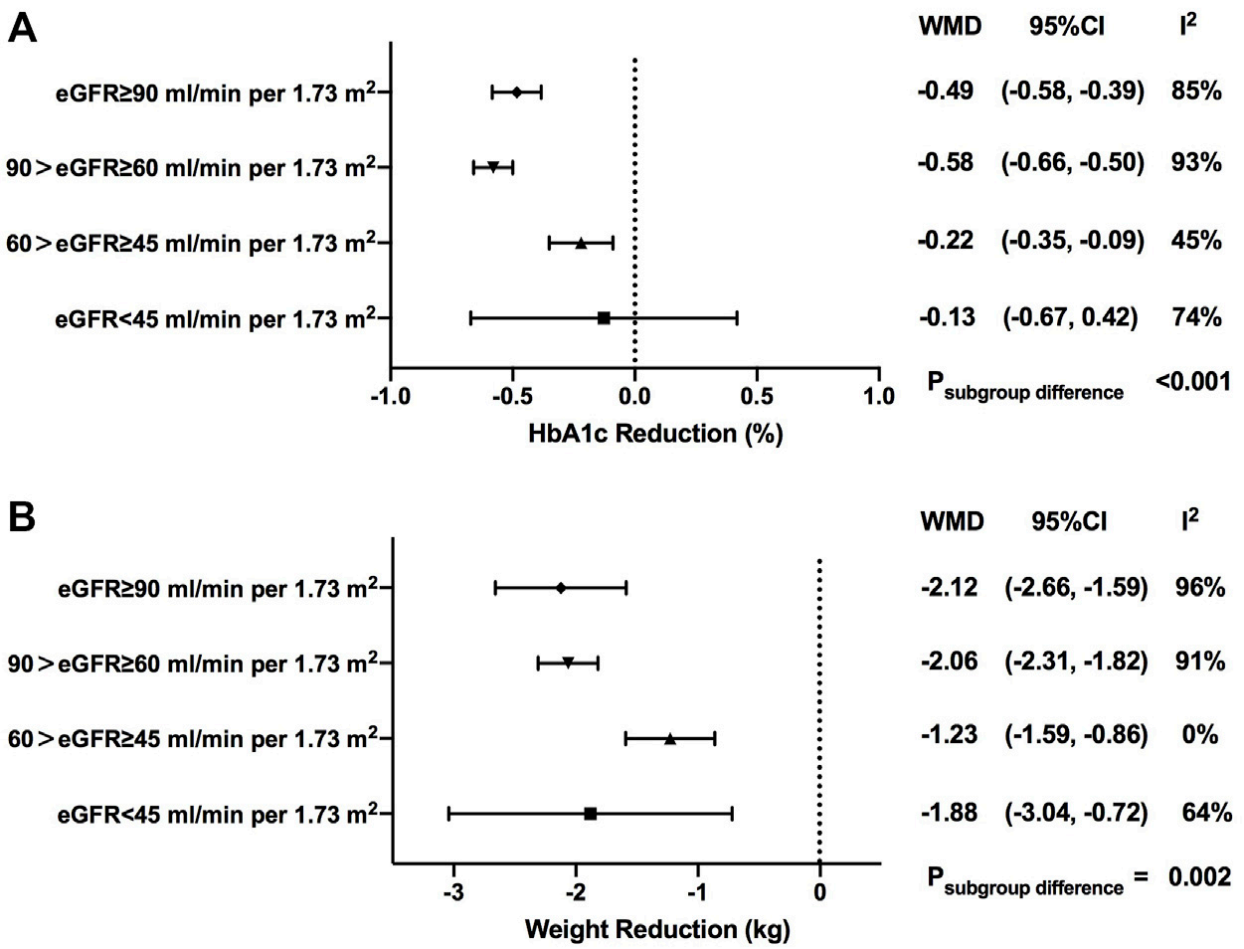
HbA1c and weight changes of SGLT2i treatment in patients with different levels of renal function. HbA1c, glycosylated hemoglobin; eGFR, estimated glomerular filtrationrate; WMD, weighted mean differences; 95% CIs, 95% confidence intervals.

As shown in our results, greater HbA1c reductions were observed in patients with SGLT2i treatment *versus* control (WMD, -0.53%, 95% CI, -0.59 to -0.47%, *p* < 0.001). When stratified by baseline eGFR levels, it was revealed that the HbA1c reduction effect of SGLT2is was attenuated in patients with worse renal impairment, with -0.49% (-0.58 to -0.39%) in normal renal function, -0.58% (-0.66 to -0.50%) in mild renal function impairment, -0.22% (-0.35 to -0.09%) in moderate renal function impairment, and -0.13% (-0.67 to 0.42%) in severe renal function impairment (*p* < 0.001 for subgroup differences) (Figure 2A, Supplementary Figure S2).

When stratified by different drug categories, it was indicated that HbA1c changes with ertugliflozin users [-0.67% (-0.85 to -0.49%) in normal renal function, -0.60% (-0.93 to -0.26%) in mild renal function impairment, and -0.09% (-0.24 to 0.06%) in moderate renal function impairment (*p* < 0.001 for subgroup differences)] and HbA1c changes with luseogliflozin users [-1.13% (-1.34 to -0.92%) in mild renal function impairment and -0.20% (-0.42 to 0.02%) in moderate renal function impairment (*p* < 0.001 for subgroup differences)] basically followed the similar pattern as the overall trend, with a precipitous deceleration in blood glucose improvement observed in the subgroup of eGFR <60 ml/min per 1.73 m^2^. However, no statistically significant subgroup difference was found in other subtypes of SGLT2is in HbA1c reduction when stratified by renal function (Supplementary Table S3). When stratified by the follow-up period, a similar changing trend pattern was observed in HbA1c reduction among different renal function groups with a follow-up less than 1 year (Supplementary Table S4).

### Effects of SGLT2is on body weight

In total, weight loss was more profound in the SGLT2i treatment group when compared with the control one (WMD, -2.05 kg, 95% CI, -2.31 to -1.79 kg, *p* < 0.001). Generally speaking, compared with patients with normal renal function, the magnitude of body weight reduction started to decline in the subgroup of patients with mild-to-moderate renal function impairment [-2.12 kg (-2.66 to -1.59 kg) in normal renal function; -2.06 kg (-2.31 to -1.82 kg) in mild renal function impairment; -1.23 kg (-1.59 to -0.86 kg) in moderate renal function impairment), but not in the subgroup with severe renal function impairment [-1.88 kg (-3.04 to -0.72 kg)]. Significant differences in body weight reduction were observed among patients at different stages of renal function (*p* = 0.002) (Figure 2B, Supplementary Figure S3).

When stratified by different drug categories, it was revealed that weight reduction effects of canagliflozin [-2.5 kg (-3.05 to -1.95 kg) in normal renal function; -1.73 kg (-2.05 to -1.42 kg) in mild renal function impairment; *p* = 0.02 for subgroup differences] and of empagliflozin [-2.17 kg (-2.64 to -1.69 kg) in normal renal function; -1.93 kg (-2.11 to -1.75 kg) in mild renal function impairment; -1.17 kg (-1.75 to -0.59 kg) in moderate renal function impairment; -1.00 kg (-2.57 to 0.57 kg) in severe renal function impairment; *p* = 0.03 for subgroup differences] gradually declined as renal impairment got worse. The results of other subtypes of SGLT2is are also summarized in Supplementary Table S3.

When stratified by the follow-up period, no significant changing trend patterns were identified in patients with a follow-up less or more than 1 year (Supplementary Table S4).

### Effects of SGLT2is on blood pressure

**FIGURE 3:**
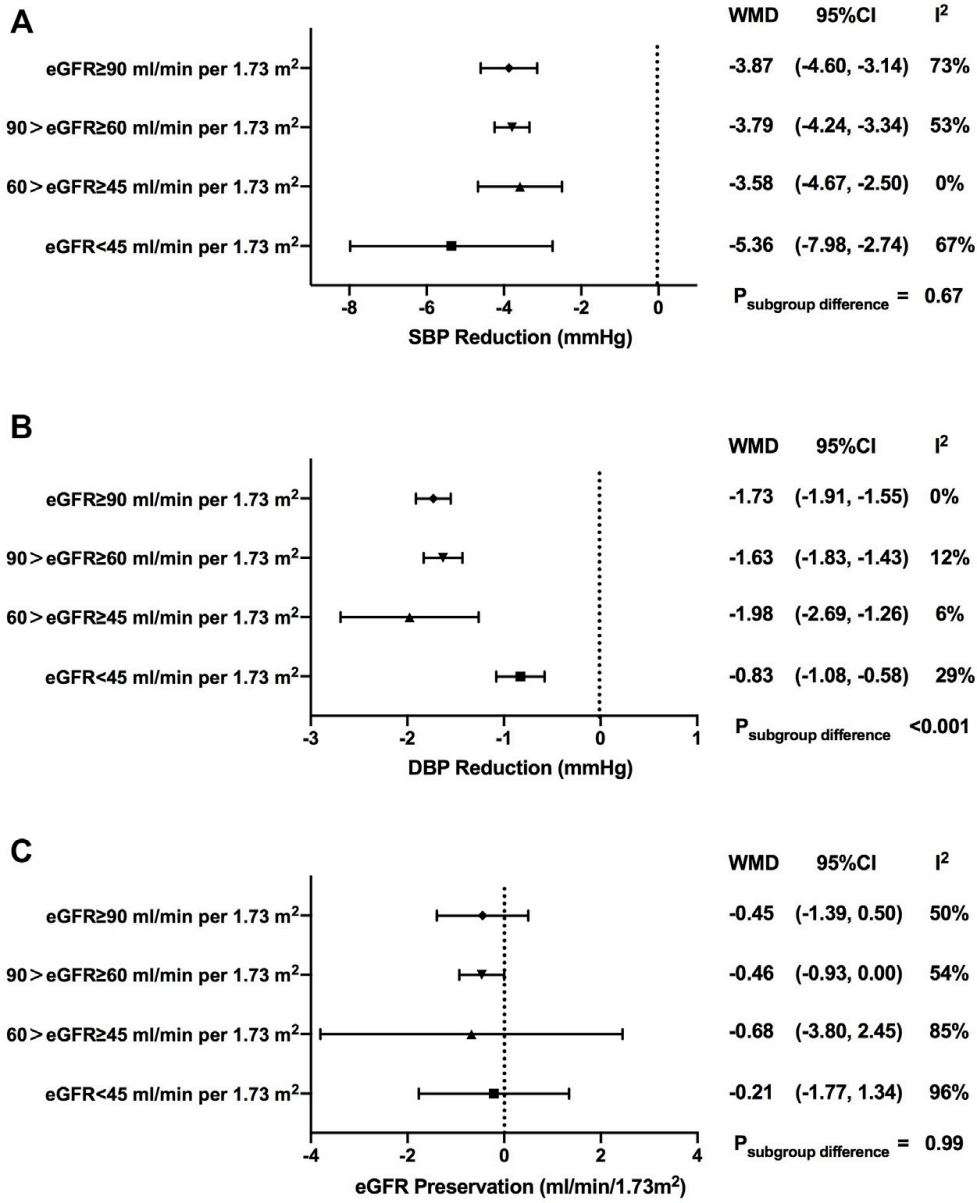
Blood pressure and eGFR changes of SGLT2i treatment in patients with different levels of renal function. SBP, systolic blood pressure; DBP, diastolic blood pressure; eGFR, estimated glomerular filtrationrate; WMD, weighted mean differences; 95% CIs, 95% confidence intervals.

SGLT2i treatment contributed to greater reductions for both systolic blood pressure (SBP) and diastolic blood pressure (DBP) when compared with control treatment [WMD, -3.87 mmHg, 95% CI, -4.30 to -3.44 mmHg for SBP, *p* < 0.001; WMD, -1.51 mmHg, 95% CI, -1.62 to -1.39 mmHg for DBP, *p* < 0.001] (Figure 3A and Figure 3B).

In terms of SBP, although there were no significant differences among subgroups with different renal function, SGLT2i-mediated blood pressure reduction seemed to be weakened when renal function got worse (WMD, -3.87 mmHg, 95% CI, -4.60 to -3.14 mmHg in normal renal function; WMD, -3.79 mmHg, 95% CI, -4.24 to -3.34 mmHg in mild renal function impairment; WMD, -3.58 mmHg, 95% CI, -4.67 to -2.50 mmHg in moderate renal function impairment; *p* = 0.67 for subgroup differences). Exceptionally, the greatest reduction of SBP was observed in individuals in the severe renal function impairment groups (WMD, -5.36 mmHg, 95% CI, -7.98 to -2.74 mmHg) (Supplementary Figure S4).

Subgroup analyses showed that there were no significant differences in SBP reduction among subgroups with different renal functions when stratified by different drug categories (Supplementary Table S3) or stratified by the follow-up period (Supplementary Table S4).

As for DBP, there was a prominent decrease in DBP in individuals with severe renal function impairment [-1.73 mmHg (-1.91 to -1.55 mmHg) in normal renal function; -1.63 mmHg (-1.83 to -1.43 mmHg) in mild renal function impairment; -1.98 mmHg (-2.69 to -1.26 mmHg) in moderate renal function impairment; -0.83 mmHg (-1.08 to -0.58 mmHg) in severe renal function impairment; *p* < 0.001 for subgroup differences] (Supplementary Figure S5).

A gradual decrease in DBP reduction was observed as renal function got worse in dapagliflozin users (WMD, -1.80 mmHg, 95% CI, -2.02 to -1.59 mmHg in normal renal function; WMD, -0.73 mmHg, 95% CI, -1.45 to -0.01 mmHg in mild renal function impairment; WMD, -0.47 mmHg, 95% CI, -2.62 to 1.68 mmHg in severe renal function impairment; *p* = 0.01 for subgroup differences). No clear changing trend pattern was found in subgroup analyses of the follow-up period (Supplementary Table S3 and Supplementary Table S4).

### Effects of SGLT2is on eGFR

Over the follow-up time ranging from 4 to 135 weeks, SGLT2i treatment failed to contribute to eGFR preservation compared with control treatment (WMD, -0.48 ml/min/1.73 m^2^, 95% CI, -0.87 to -0.08 ml/min/1.73 m^2^, *p* = 0.02). Such fluctuations of the eGFR showed no notable change pattern as renal function declined, which were comparable among subgroups with different levels of renal function [-0.45 ml/min/1.73 m^2^ (-1.39 to 0.50 ml/min/1.73 m^2^) in normal renal function; -0.46 ml/min/1.73 m^2^ (-0.93 to 0.00 ml/min/1.73 m^2^) in mild renal function impairment; -0.68 ml/min/1.73 m^2^ (-3.80 to 2.45 ml/min/1.73 m^2^) in moderate renal function impairment; -0.21 ml/min/1.73 m^2^ (-1.77 to 1.34 ml/min/1.73 m^2^) in severe renal function impairment; *p* = 0.99 for subgroup differences)] (Figure 3C and Supplementary Figure S6).

In subgroup analyses for different drug categories, greater eGFR decline was found with sotagliflozin and luseogliflozin treatment [-1.22 ml/min/1.73 m^2^ (-1.47 to -0.97 ml/min/1.73 m^2^), *p* < 0.001 for sotagliflozin; -2.09 ml/min/1.73 m^2^ (-3.54 to -0.63 ml/min/1.73 m^2^), *p* = 0.005 for luseogliflozin]. Significant eGFR decline *versus* control was observed when the follow-up duration was less than 1 year (Supplementary Table S3 and Supplementary Table S4).

### Effects of SGLT2is on adverse events

**FIGURE 4:**
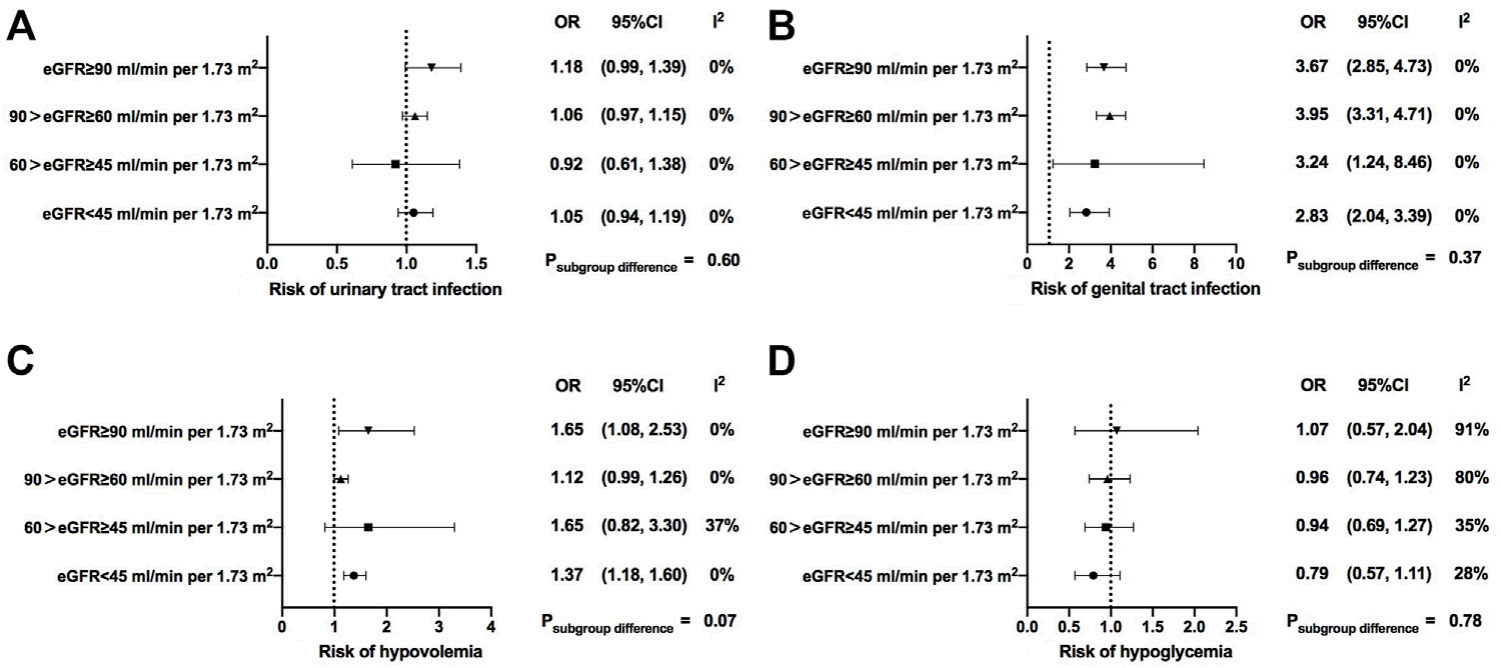
Safety of SGLT2i treatment in patients with different levels of renal function. eGFR, estimated glomerular filtrationrate; OR, odds ratio; 95% CIs, 95% confidence intervals.

In addition, we compared several major adverse events (AEs) between SGLT2i treatment and the control group. Overall, compared with the control group, the risks of urinary tract infection (OR = 1.07, 95% CI, 1.00 to 1.14, I^2^ = 0%), genital infection (OR = 3.69, 95% CI, 3.23 to 4.20, I^2^ = 0%), hypovolemia (OR = 1.24, 95% CI, 1.13 to 1.35, I^2^ = 0%), and diabetic ketoacidosis (OR = 2.23, 95% CI, 1.59 to 3.11, I^2^ = 25%) were significantly increased in SGLT2i users (Supplementary Figures S7–S14). The risk of hypovolemia was significantly increased in subgroups with normal renal function and severe renal function impairment [OR = 1.65 (95% CI, 1.08–2.53) in normal renal function, *p* = 0.02; OR = 1.12 (95% CI, 0.99–1.26) in mild renal function impairment, *p* = 0.06; OR = 1.65 (95% CI, 0.82–3.3) in moderate renal function impairment, *p* = 0.16; OR = 1.37 (95% CI, 1.18–1.6) in severe renal function impairment, *p* < 0.001; *p* = 0.07 for subgroup differences] (Figure 4). The risk of hypoglycemia was not increased in SGLT2i users across different renal function strata in the overall analysis (Figure 4). However, when compared with the placebo, the incidence of hypoglycemia in SGLT2i users was only significantly increased in patients with normal renal function and the effect sizes gradually decreased as renal function getting worse in patients treated with SGLT2is [OR = 1.57 (95% CI, 1.29–1.91) in normal renal function; OR = 1.07 (95% CI, 0.98–1.16) in mild renal function impairment; OR = 0.94 (95% CI, 0.74–1.19) in moderate renal function impairment; OR = 0.80 (95% CI, 0.62–1.05) in severe renal function impairment; *p* < 0.001 for subgroup differences] (Supplementary Table S6).

## Discussion

With the aim to compare the pleiotropic properties of SGLT2is among individuals with varying levels of renal function, we found that SGLT2is produced a substantial benefit in blood glucose improvement, weight, and blood pressure reduction even in patients with impaired renal function. We also found that the effects of SGLT2is on blood glucose, weight, and blood pressure control varied among different renal function groups, some were parallel with the renal function levels while others were not.

### Glycemic control

As it was suggested that SGLT2is exerted their glucose-lowering effect mainly through glycosuria (DeFronzo et al., 2012; Washburn and Poucher, 2013; Abdul-Ghani and DeFronzo, 2014; Hasan et al., 2014; Palmer et al., 2021), previous studies showed that SGLT2i-mediated urinary glucose excretion was renal function-dependent, reducing with progressed renal function impairment (Scheen, 2015; Hu et al., 2022). Consistent with previous studies, we suggested a greater reduction in HbA1c levels in patients treated with SGLT2is than non-SGLT2i users, which was attenuated by the decreased level of eGFR.

### Weight loss

On the basis of our studies, it was suggested that a statistically significant difference in SGLT2i-related weight loss was found among different renal functions. As we mentioned before, UGE of SGLT2is is declining with decreasing renal function. Likewise, we did observe the weight reduction effects of SGLT2is were weakened as renal function got worse. Thus, it was reasonable to speculate that urinary glucose excretion and its associated energy loss might play a predominant role in weight control for SGLT2is.

Of course, reduction in body weight with SGLT2is therapy was considered a result of the combination of several systemic factors, including increased excretion of glucose (Abdul-Ghani et al., 2012), reductions in adipose tissue mass (both visceral and subcutaneous) (Bolinder et al., 2012; Cefalu et al., 2013), preservation of lean tissue mass, and loss of extracellular fluid (Schork et al., 2019). However, we also observed a sudden increase in weight loss in patients with severe renal function impairment. Perhaps, there were other compensatory mechanisms that potentially triggered lipolysis, and in turn, led to weight loss during the treatment of SGLT2is in patients with severe renal impairment. It remains to be elucidated with further investigations.

### Reduction in systemic blood pressure

In line with previous analyses, our data suggested that SGLT2i treatment was associated with a reduction in systemic blood pressure (Foote et al., 2012; Monami et al., 2014). The initial reduction in extracellular fluid volume (Lambers Heerspink et al., 2013; Vasilakou et al., 2013; Baker et al., 2014; Oliva and Bakris, 2014; Scheen, 2015; Thomas and Cherney, 2018), a further loss in body mass, modulation of the RAAS (Burns and Cherney, 2019), and reduced plasma uric acid levels (Zhao et al., 2018) are likely to lead to reduction in blood pressure.

Interestingly, we found there was a difference in the SGLT2i effect on systolic and diastolic blood pressure. The magnitudes of SBP reduction among different subgroups were comparable. Even in patients with severe impaired renal function, SGLT2is were comparably effective in lowering systolic blood pressure. However, in terms of DBP, we observed a general trend toward more pronounced blood pressure reduction in individuals with better renal function.

Possible mechanisms for the aforementioned findings might be explained as follows. Greater use of antihypertensive medications (including diuretics) was found in patients with worsening chronic kidney disease (CKD) (Cherney et al., 2018). At the same time, patients with CKD exhibited sodium-sensitive phenotypes, leading to significant blood pressure–lowering effects from natriuretic agents (Luzardo et al., 2015). Also, natriuresis and urinary volume were increased in patients with T2D when given empagliflozin in combination with a thiazide or a loop diuretic, compared with either therapy alone (Heise et al., 2016). Thus, drug–drug interaction might partly explain the preserved effect of SGLT2is on SBP with lower eGFRs. However, as CKD developed and progressed, decreasing aortic compliance led to a consequent drop in the diastolic vascular flow and pressure (Inserra et al., 2021). Thus, it possibly made sense that less reduction in DBP with SGLT2is would occur in severe renal function impairment since baseline levels stayed low, either. As mentioned previously, the pathophysiology for BP lowering with SGLT2is has been attributed to several factors. More mechanisms on SGLT2i′ BP- lowering effects among different renal functions remained to be explored further.

### Preservation of renal function

Although SGLT2is caused an initial and small reduction in eGFR in the early stage of treatment (Perkovic et al., 2019), previous studies found the reversal of these small changes in eGFR with long-term treatment and that SGLT2is could maintain long-term renoprotective effects ultimately (Pareek et al., 2016).

Similar to existing studies, we observed a transient reduction in eGFR compared with controls when the follow-up duration was less than 1 year. However, we did not see preservation of renal function in SGLT2is compared with controls in the extended follow-up time. Since renal benefits of SGLT2is have been confirmed in several large RCT trials, we summed up the following reasons that may lead to the negative results in our data.

First, the clinical trials reflecting changes in the eGFR included in our analysis were limited, and the sample size was insufficient. Second, as we could see in the dapagliflozin and empagliflozin subgroup, patients with normal renal function or mild renal insufficiency accounted for the majority. However, changes in the eGFR in these patients were quite slight. Third, the difference in the eGFR slope between SGLT2is and control arms varied during follow-up, which was seen in CANVAS、DAPA-HF、and EMPEROR studies (Neuen et al., 2018; Jhund et al., 2021; Zannad et al., 2021).

In CANVAS, participants who received canagliflozin experienced a decline in the eGFR within the first 13 weeks. While after week 13, the annual decline in the eGFR was significantly slower in all subgroups (Neuen et al., 2018). Likewise, after day 14, the rate of decline of the eGFR was steeper in the placebo group than in the dapagliflozin group in DAPA-HF (Jhund et al., 2021). In EMPEROR-Reduced trial, at week 4, the eGFR stabilized and recovered toward baseline, whereas progressive decline was observed in the placebo group (Zannad et al., 2021). However, in our study, we compared only changes in the eGFR but not the slope. Therefore, it is very critical to select the appropriate time point for subgroup analyses. Apparently, we had already seen a significant difference in eGFR changes between SGLT2i treatment and controls when taking “1 year” as the time point. In addition, as seen in Supplementary Table S1, the number of studies with follow-up longer than 1 year was limited. Also, when followed for more than 1 year, though it was not statistically significant, we found an increase in the eGFR in patients with mild-to-moderate renal function impairment when treated with SGLT2 inhibitors compared to controls. Therefore, studies with longer follow-up are needed to further identify the optimal time point at which the gap in eGFR changes appeared between SGLT2 inhibition and controls.

### Safety

In our analyses, incidences of urinary tract infection, genital infection, hypovolemia, and diabetic ketoacidosis were higher in the SGLT2i users. At the same time, compared to the placebo, fewer episodes of hypoglycemia were reported in patients with deteriorating renal function, which made sense as urinary glucose excretion reduced with progressed renal function impairment (Scheen, 2015; Hu et al., 2022). Nevertheless, the risks of other AEs like genital infection and diabetic ketoacidosis were comparable among different renal functions. In addition, we observed a significantly increased risk of hypovolemia in patients with normal renal function and severe renal function impairment. Diuretic effects of SGLT2i led to a decrease in plasma volume and were related to a higher risk of volume depletion (Delanaye and Scheen, 2021). Meanwhile, since hypertension and edema are common comorbidities and complications in patients with CKD (Kidney Disease: Improving Global Outcomes KDIGO Blood Pressure Work Group, 2021), concomitant antihypertensive medications, including diuretics, might also be applied in these patients, which might also be associated with the increased risk of hypovolemia. Moreover, cardiovascular autonomic dysfunction in patients with advanced CKD and end-stage kidney disease (ESKD) might also be associated with the increased incidence of hypovolemia and hypotension (Soomro and Charytan, 2021; Shubrook et al., 2022). However, further analyses were limited due to insufficient information of baseline medications and cardiovascular profiles. Considering certain active comparators with an increased risk of hypoglycemia, such as sulfonylureas and insulin, might lead to a biased hypoglycemia evaluation, we conducted a further sensitivity analysis with only placebo-controlled RCTs. We found that, when compared with the placebo, the risk of hypoglycemia during SGLT2i treatment was elevated in patients with favorable renal function rather than those with renal dysfunction, which was consistent with the decreasing UGE and HbA1c reductions observed in SGLT2i users as the renal function deteriorates. Therefore, SGLT2i was generally well-tolerated in patients with renal impairments in terms of hypoglycemia.

### Strengths and limitations

To our knowledge, our study is currently the largest systematic review showing the effect of administration of SGLT2is on different renal function stratifications. Our data provide strong evidence for the clinical application of SGLT2is in patients with CKD. This study has some limitations, though. First, we did not include studies without presenting data regarding changes in HbA1c, weight, blood pressure, and eGFR, and thus data collection might be incomplete. Second, because of the current eGFR-based limitations on the use of SGLT2is, inclusion criteria bias made the sample size unevenly distributed among different stratifications. We were limited to drawing a definite conclusion concerning the efficacies of SGLT2is in patients at an advanced stage of CKD in the case of a relatively small number of participants, especially for patients with an eGFR below 30 ml/min per 1.73 m^2^. Further investigations are needed to further assess SGLT2i′s effects in the population with severe renal dysfunction. Furthermore, high urine protein levels are associated with rapid decline in kidney function (Levey et al., 2020), and research studies have shown that SGLT2is reduce albuminuria with consequent benefits on kidney outcomes in patients with diabetes (Shah et al., 2022). However, given that the RCTs included in our analyses provided quite limited data regarding changes in uACR levels, we were unable to conduct a convincing analysis focusing on the SGLT2i-induced uACR changes among groups with different renal functions in our analysis. Further explorations are needed to test SGLT2i′s effects on the uACR levels in patients with declining renal function. Moreover, since we included studies with different populations, different drug types, different dosages, and follow-up periods, the potential heterogeneity lying in our analyses might influence our results. To cope with this issue, we conducted multiple subgroup analyses to control the potential bias. In addition, we performed a sensitivity analysis with only placebo-controlled RCTs to exclude the influence of active agent comparators. It turned out that most results were generally consistent with the overall analyses (Supplementary Table S6 and Supplementary Table S7). At the same time, given that a duration of less than 12 weeks might not be sufficient to evaluate the effect of HbA1c or change of eGFR, we also performed a sensitivity analysis after deleting RCTs with follow-up periods less than 12 weeks to exclude possible influences. It turned out that most results were generally consistent with the results in the overall analyses (Supplementary Table S8). However, there is no denying that the data should still be interpreted with caution. Moreover, certain unmeasured confounding factors such as baseline cardiovascular status, concomitant medications, and diet intakes were unable to be adjusted for now. More investigations are still needed for further evaluations.

## Conclusion

In conclusion, SGLT2is contributed to an improved glycemic control, body weight, and blood pressure reduction, even in patients with renal insufficiency. The HbA1c and body weight reductions observed in SGLT2i users were generally parallel with patients’ baseline eGFR levels, while blood pressure reductions in SGLT2i users were independent of baseline eGFR levels. Consistently, when compared with the placebo, risk of hypoglycemia with SGLT2i treatment was more frequent in patients with favorable renal function, where the HbA1c reduction was profound.

## Data Availability

The original contributions presented in the study are included in the article/Supplementary Materials; further inquiries can be directed to the corresponding authors.
