# Supplementary figures and images for: Disparities in efficacy and safety of sodium-glucose cotransporter 2 inhibitor among patients with different extents of renal dysfunction: A systematic review and meta-analysis of randomized controlled trials
Source: Front Pharmacol. 2022 Nov 22;13:1018720. doi: 10.3389/fphar.2022.1018720 (PMC9723253; doi:10.3389/fphar.2022.1018720)

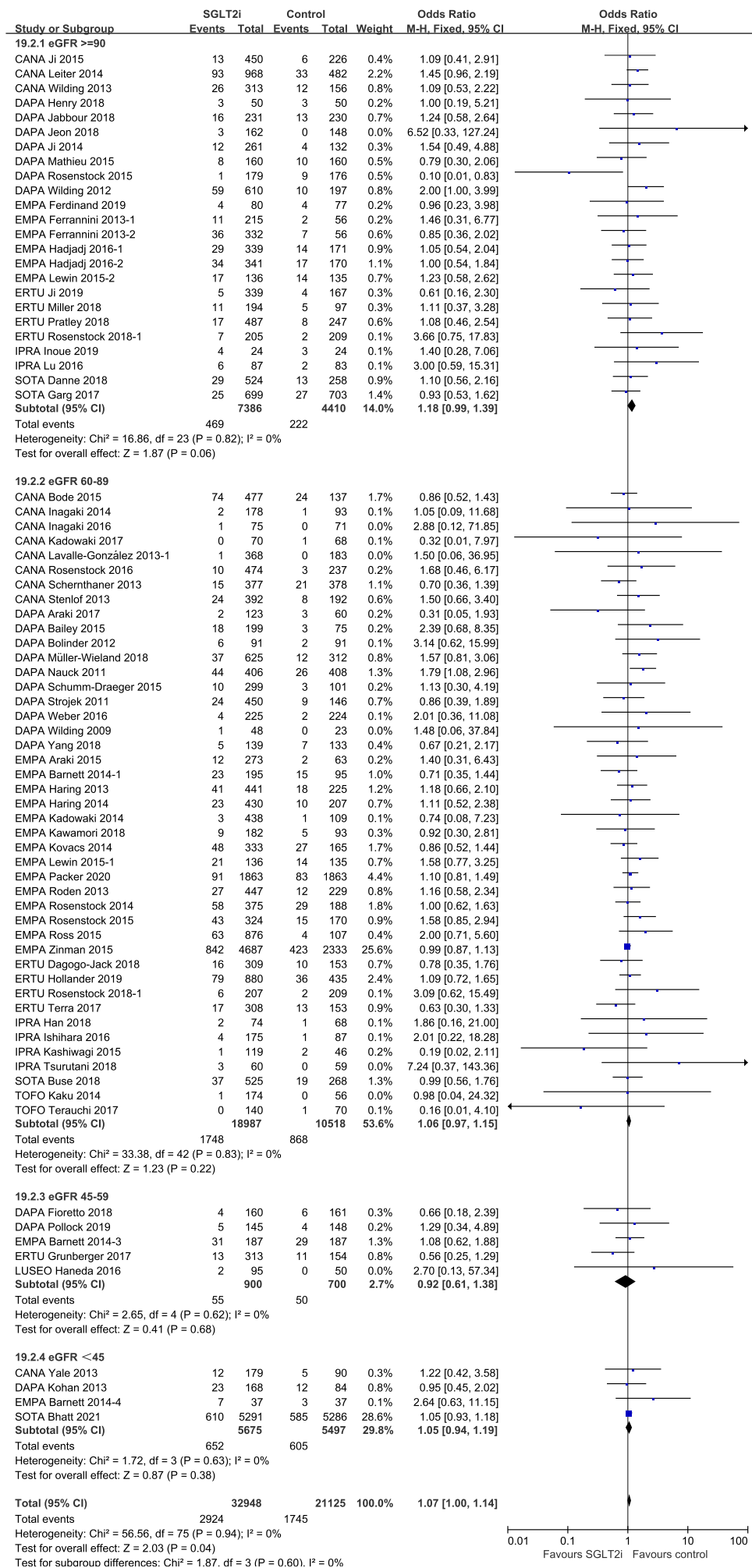

Supplement: Supplementary file 1 [file DataSheet7.PDF]

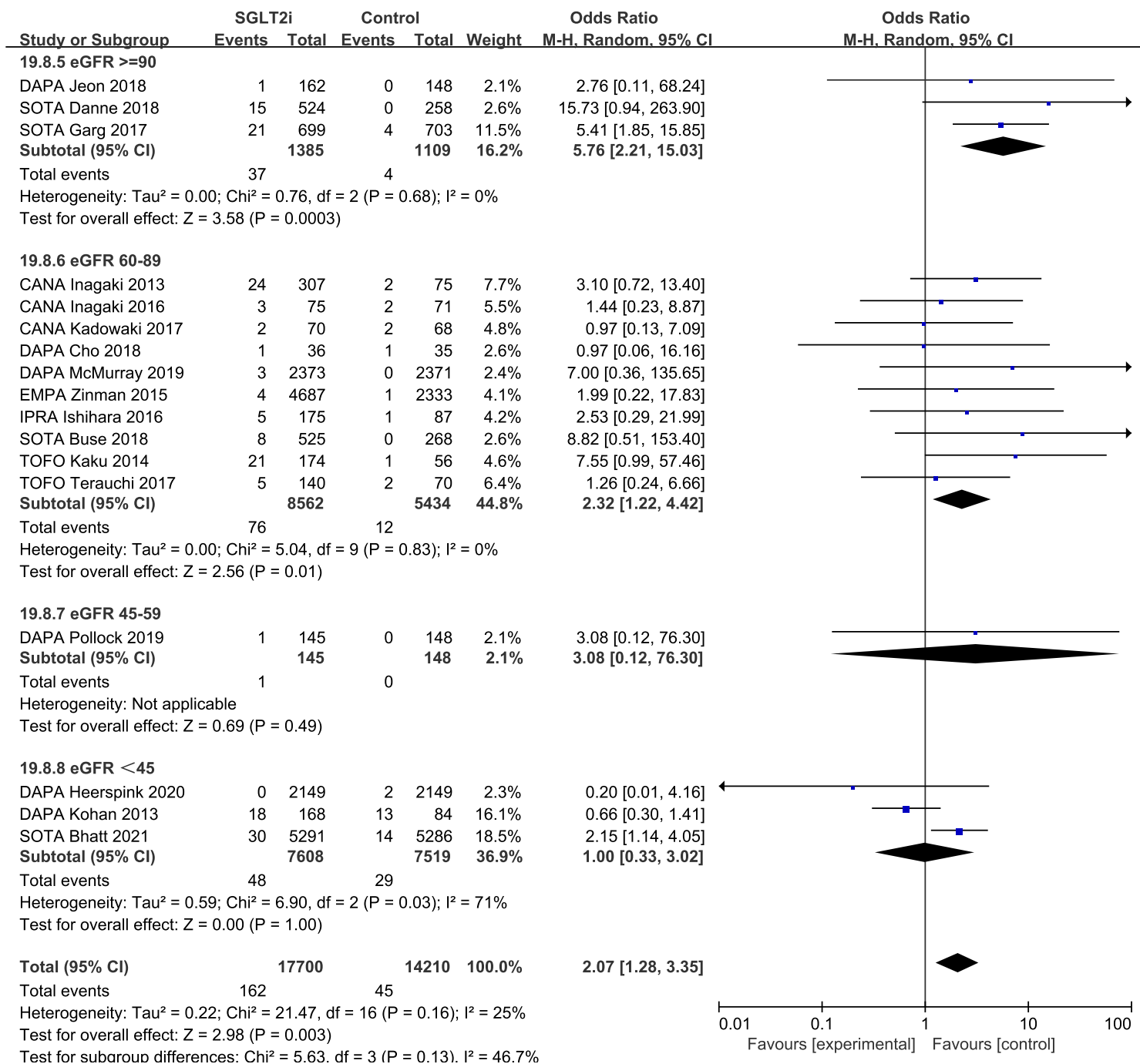

Supplement: Supplementary file 2 [file DataSheet13.PDF]

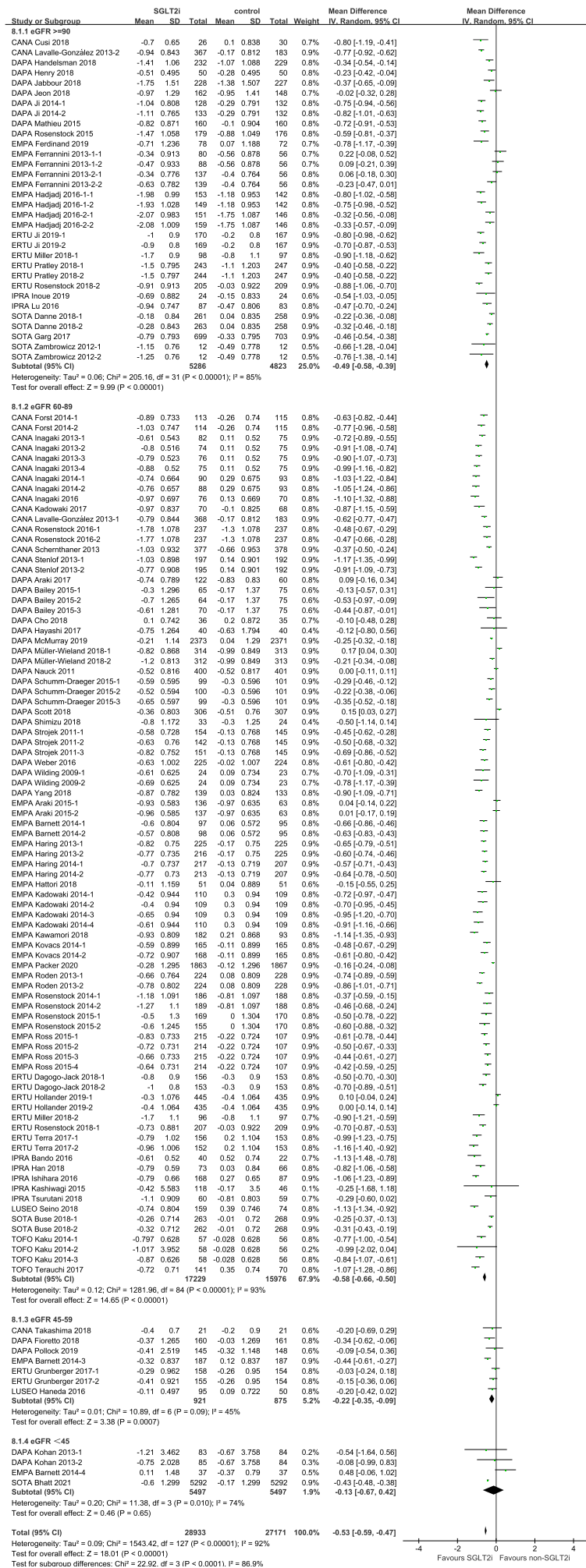

Supplement: Supplementary file 3 [file DataSheet2.PDF]

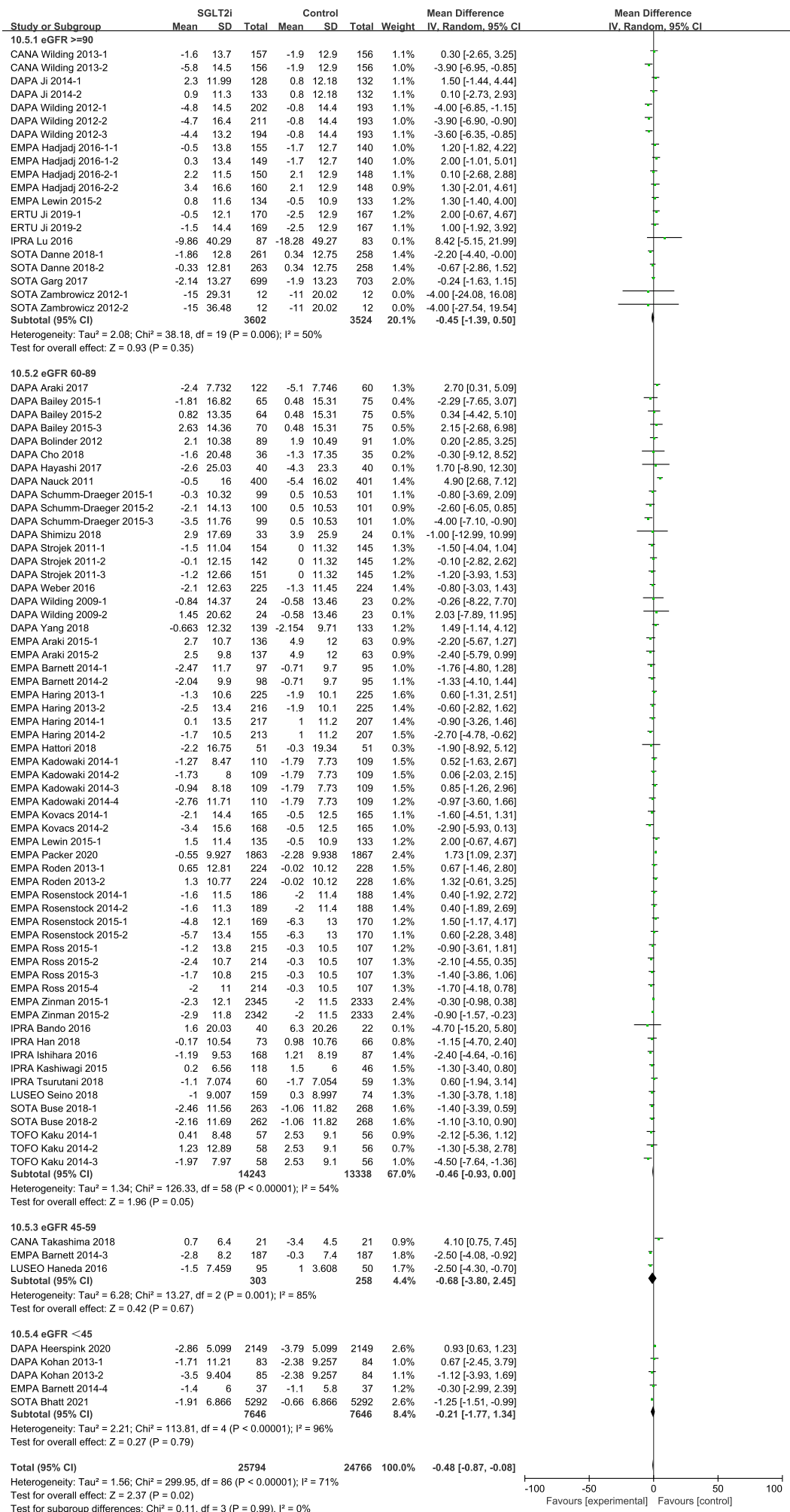

Supplement: Supplementary file 5 [file DataSheet6.PDF]

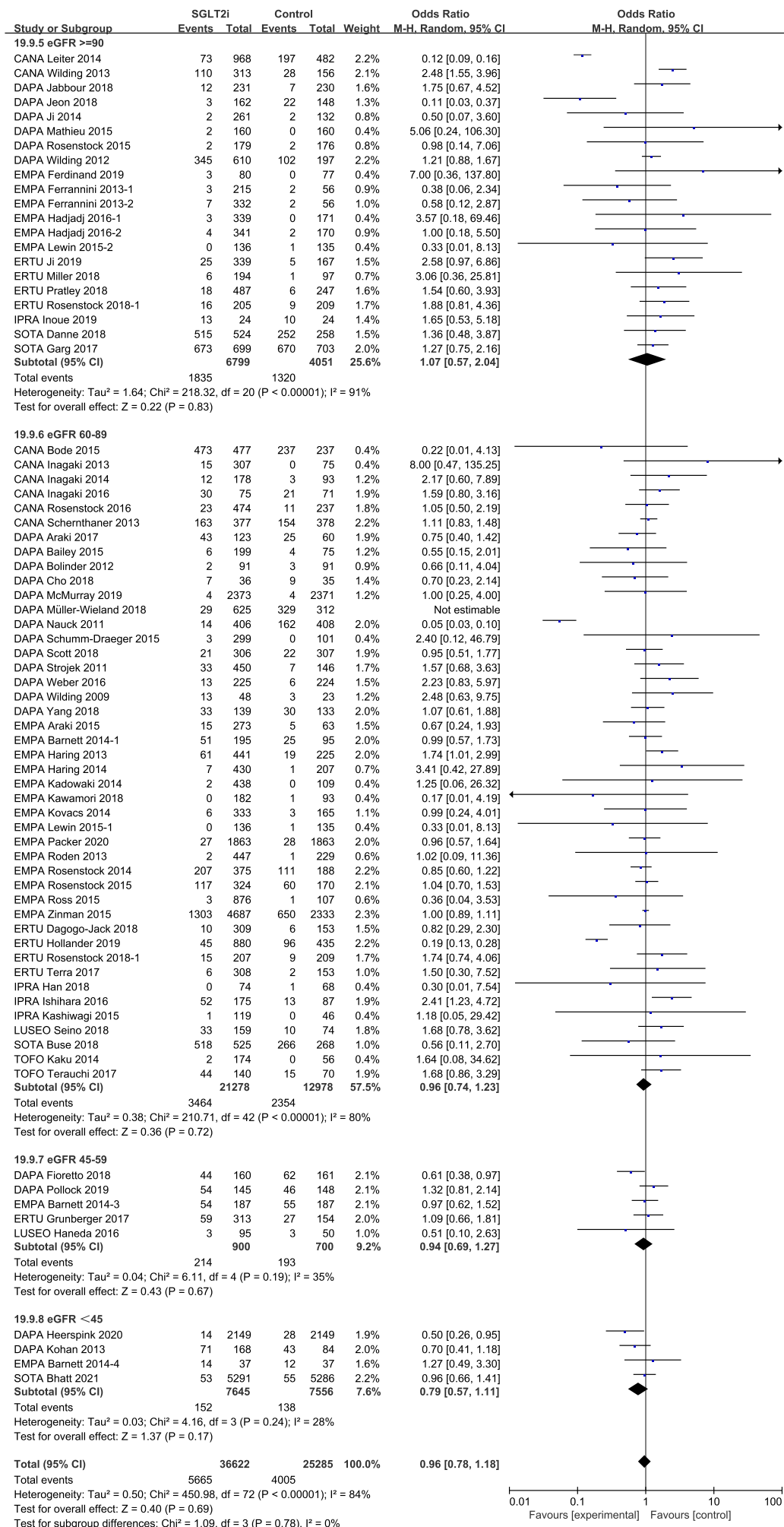

Supplement: Supplementary file 6 [file DataSheet14.PDF]

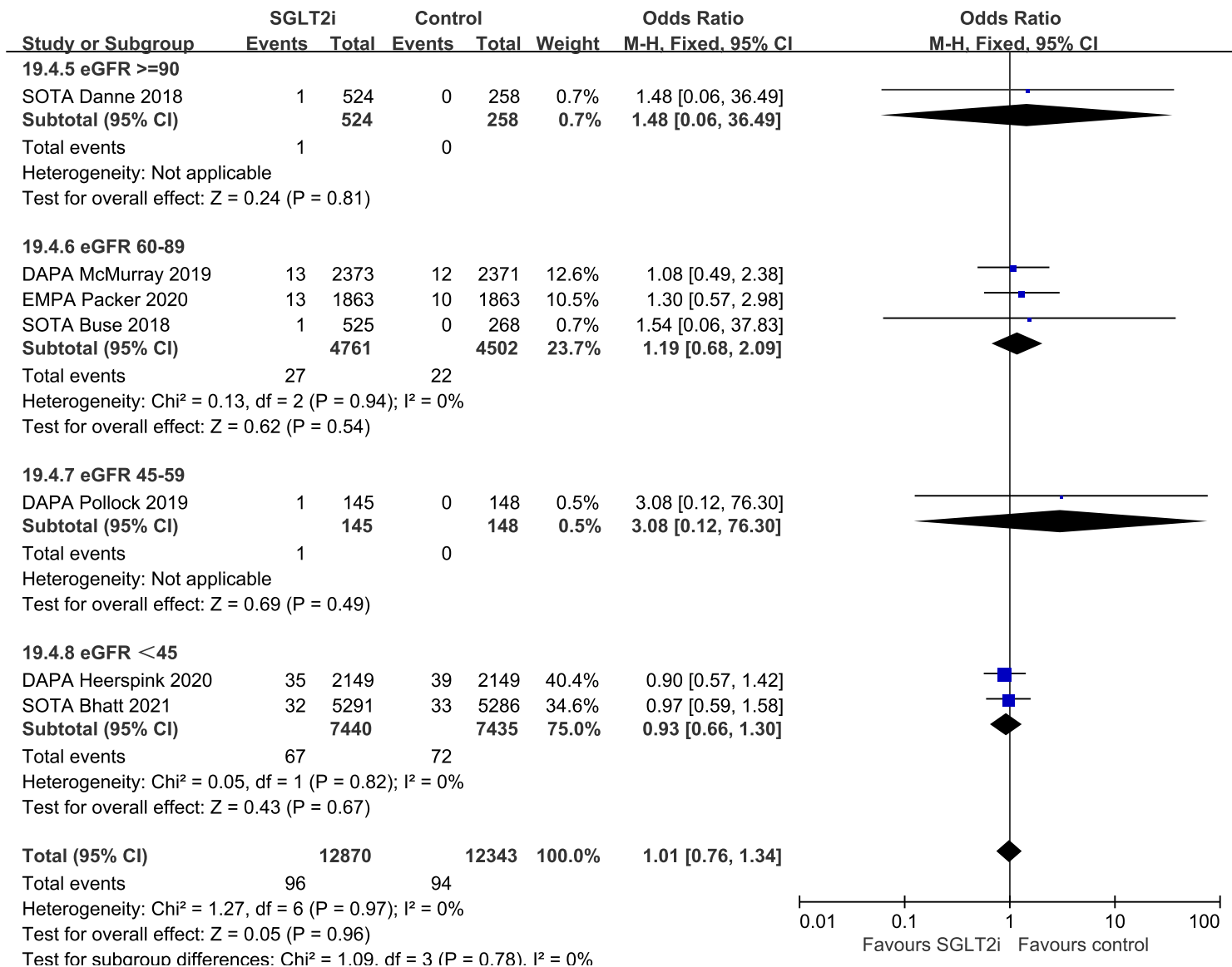

Supplement: Supplementary file 7 [file DataSheet9.PDF]

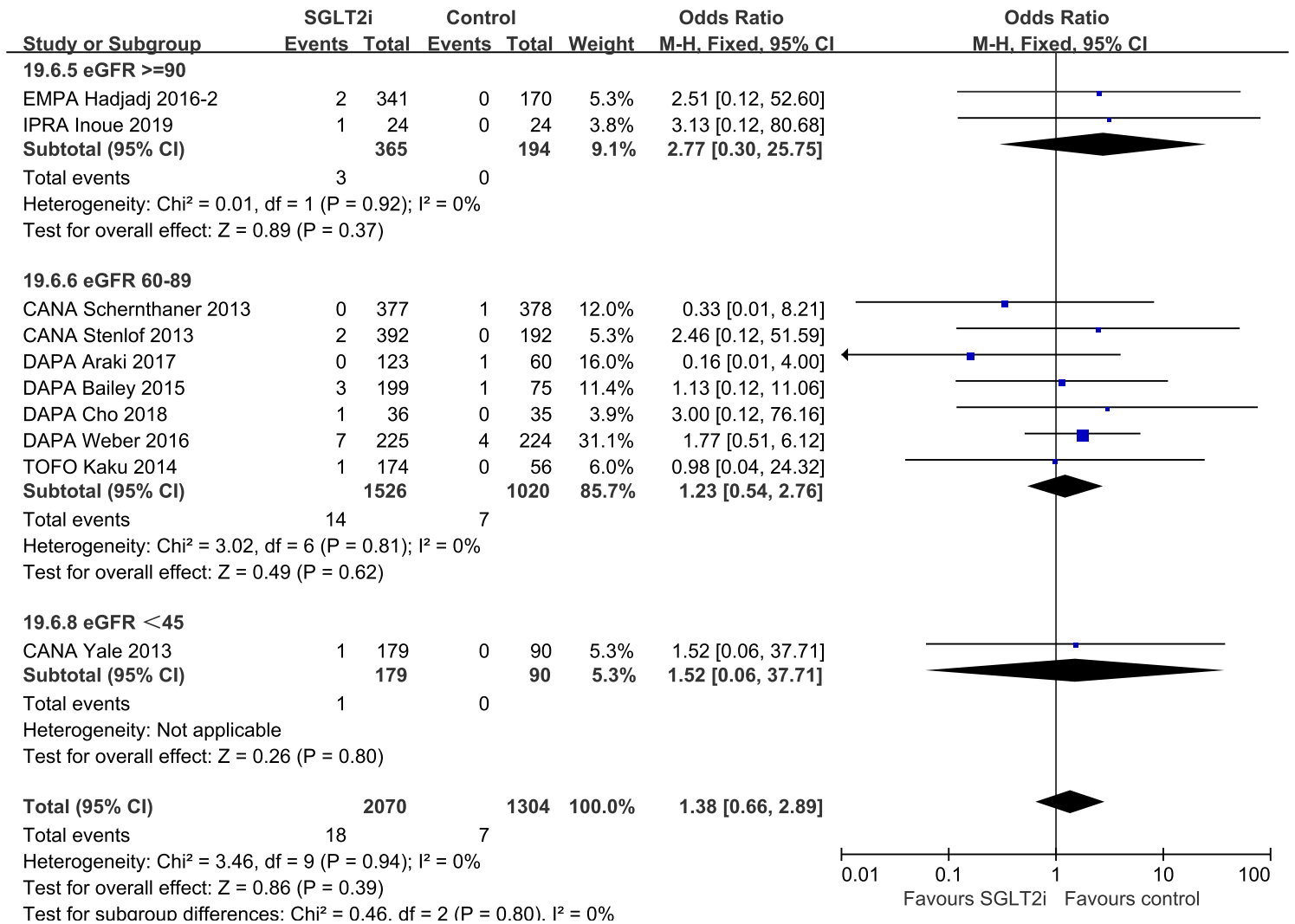

Supplement: Supplementary file 9 [file DataSheet11.PDF]

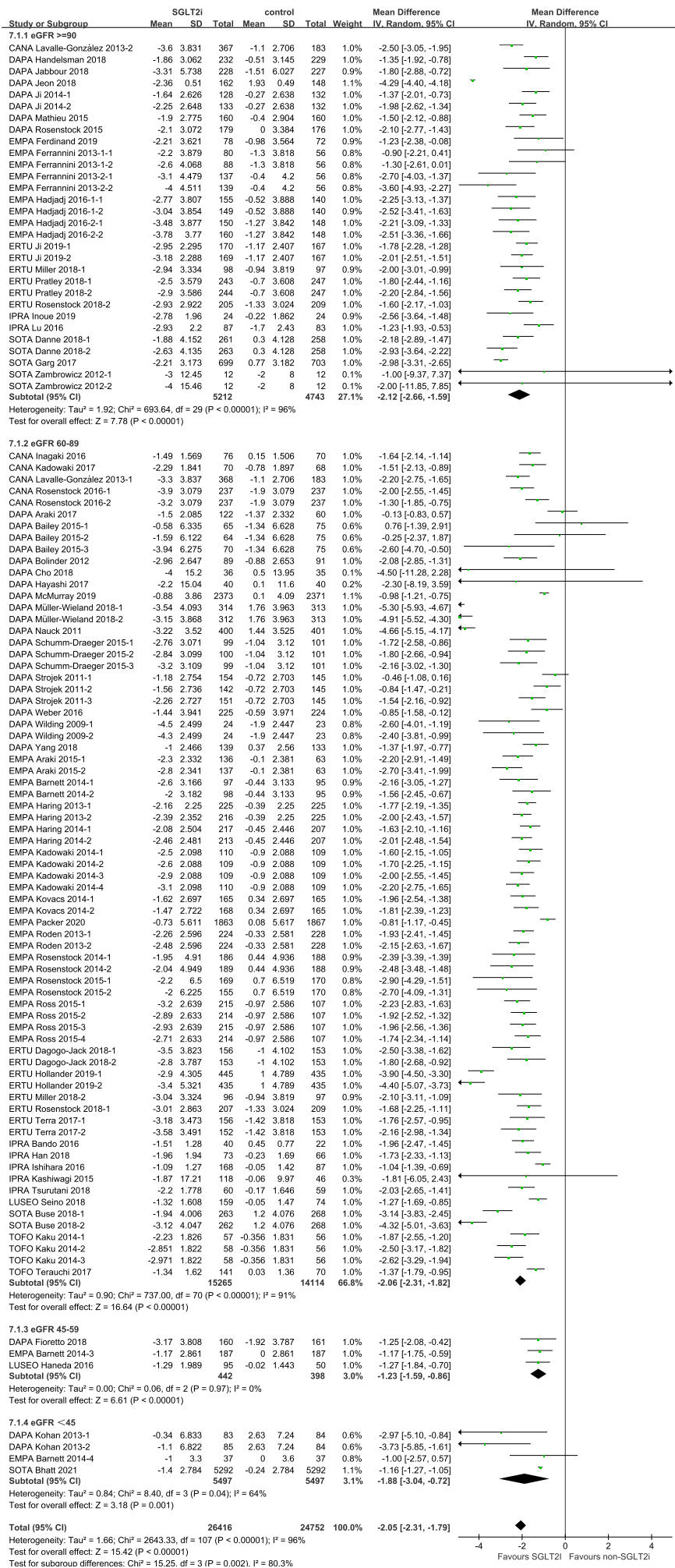

Supplement: Supplementary file 10 [file DataSheet3.PDF]

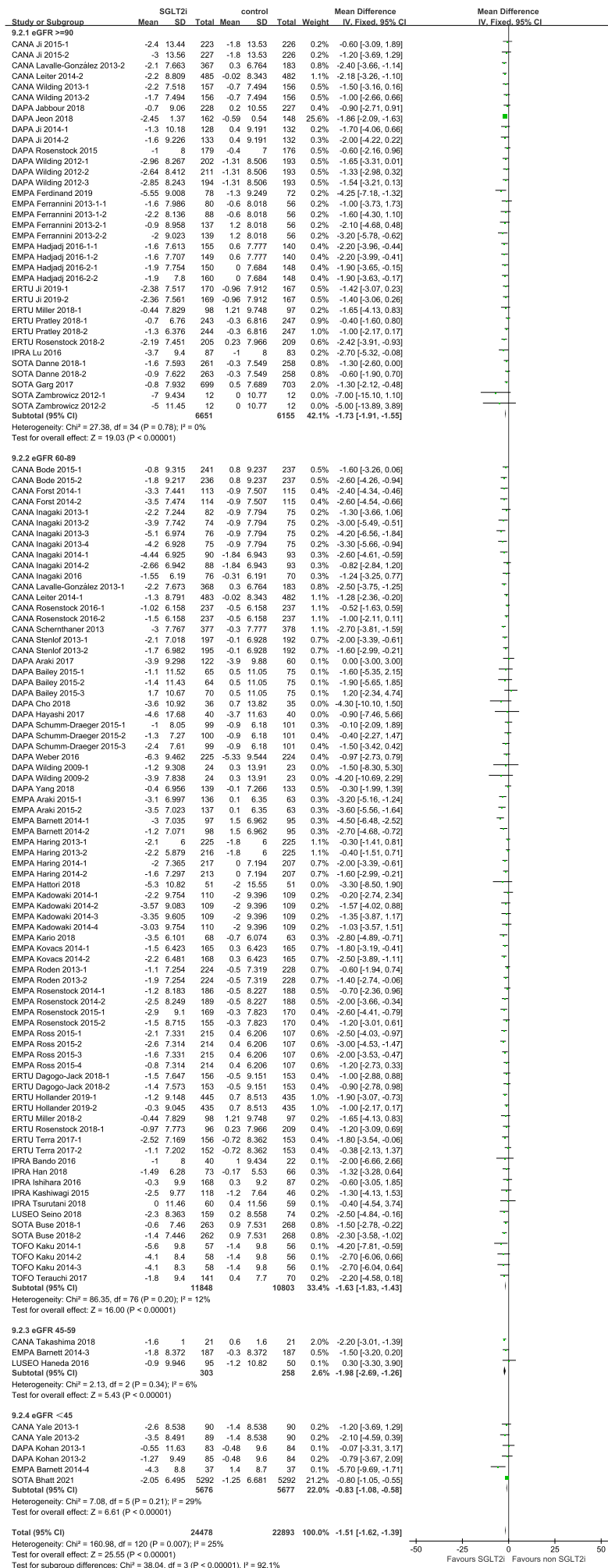

Supplement: Supplementary file 12 [file DataSheet5.PDF]

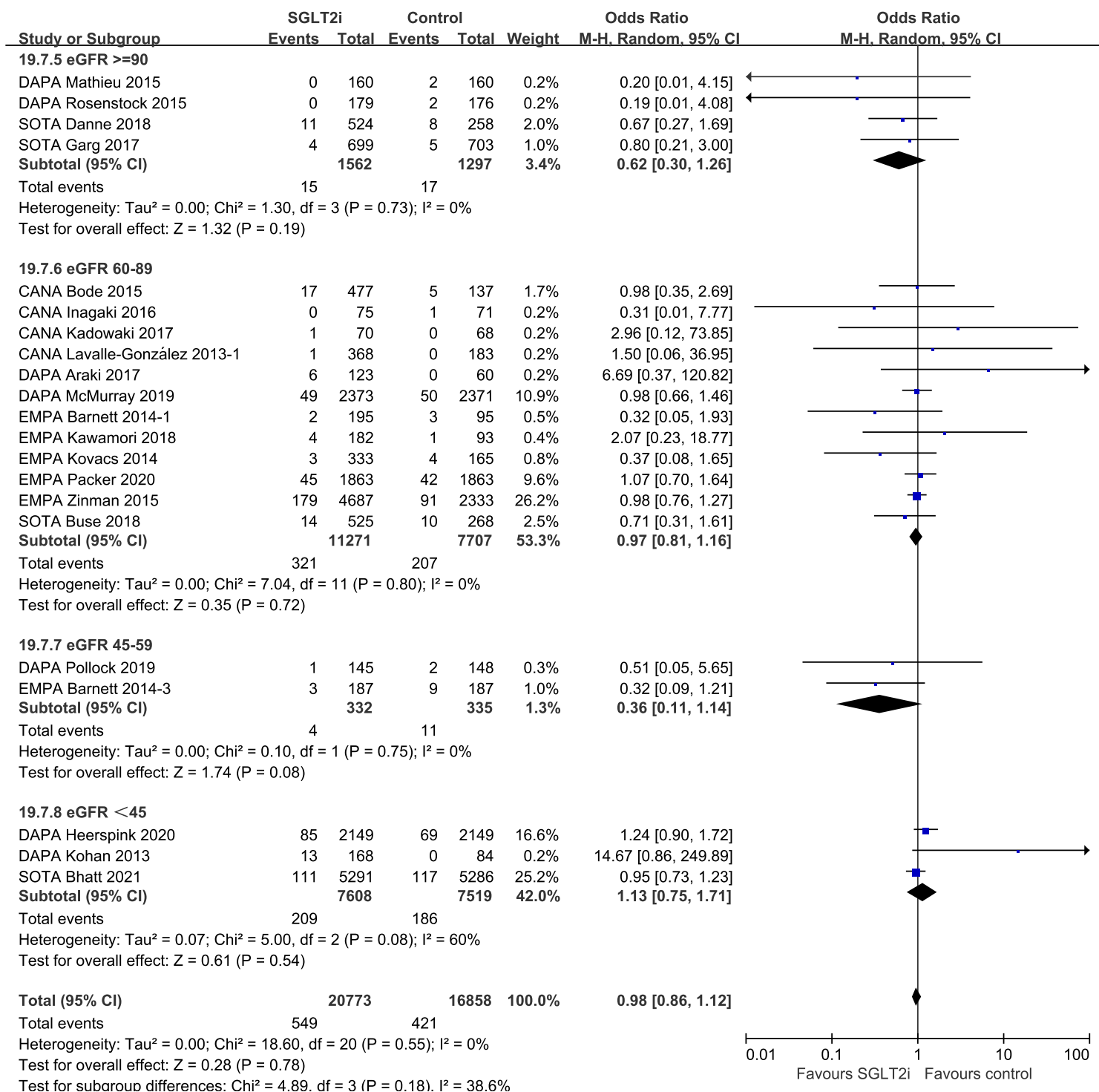

Supplement: Supplementary file 13 [file DataSheet12.PDF]

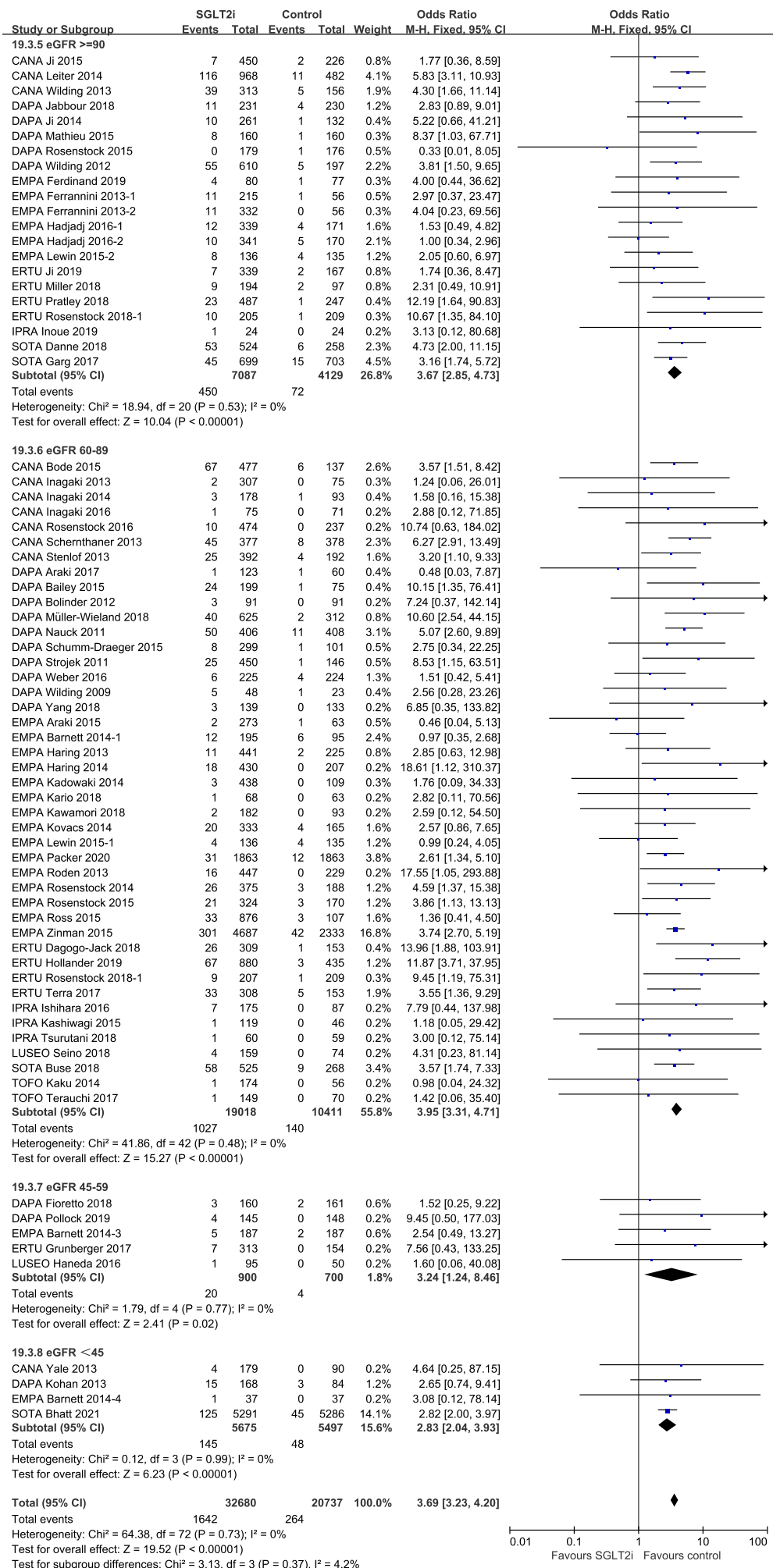

Supplement: Supplementary file 14 [file DataSheet8.PDF]

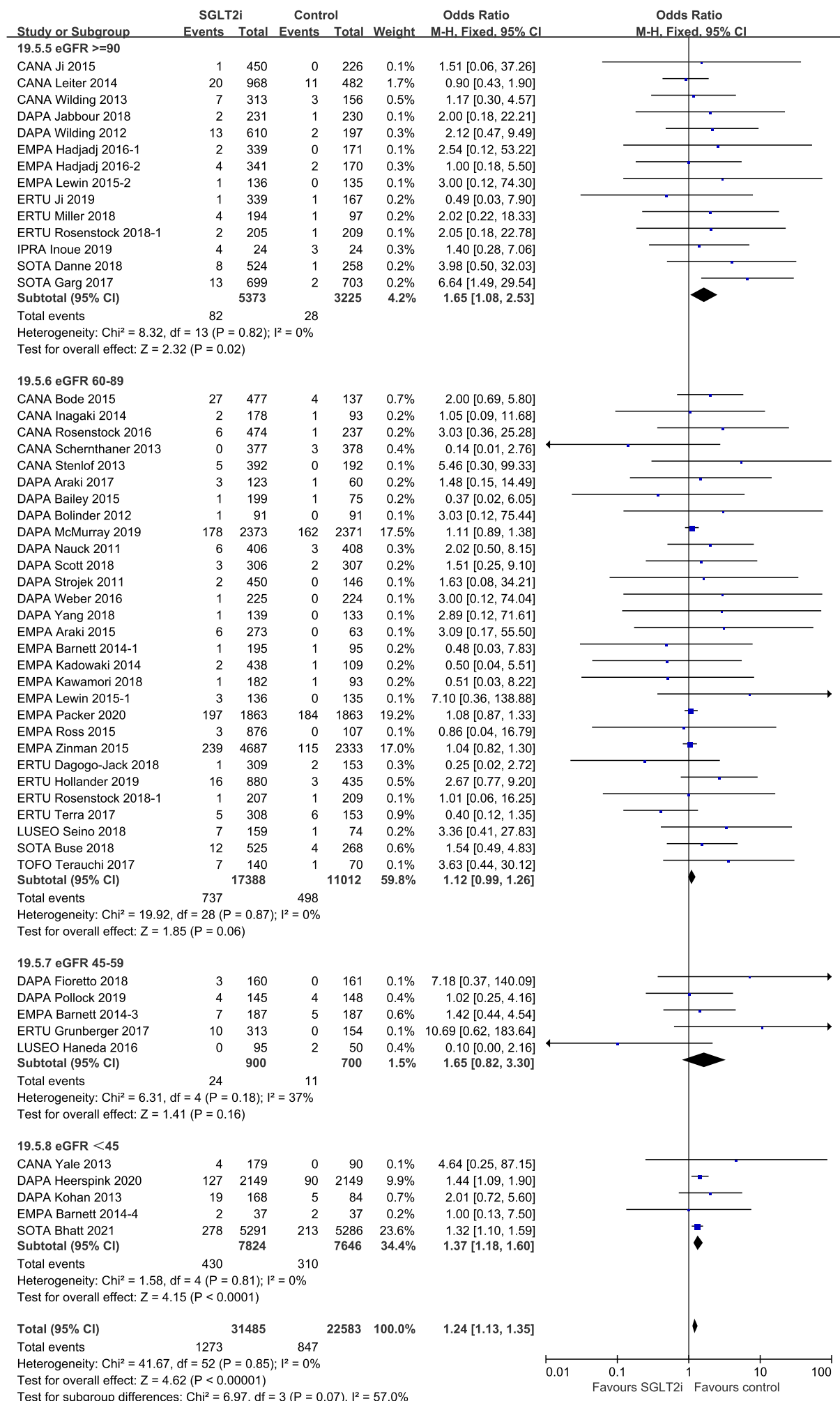

Supplement: Supplementary file 15 [file DataSheet10.PDF]
