# Supplementary material for: Disparities in efficacy and safety of sodium-glucose cotransporter 2 inhibitor among patients with different extents of renal dysfunction: A systematic review and meta-analysis of randomized controlled trials
Source: Front Pharmacol. 2022 Nov 22;13:1018720. doi: 10.3389/fphar.2022.1018720 (PMC9723253; doi:10.3389/fphar.2022.1018720)

Study or Subgroup	SGLT2i Mean	SGLT2i SD	SGLT2i Total	control Mean	control SD	control Total	Weight	Mean Difference IV, Random, 95% CI
**9.1.1 eGFR >=90**								
CANA Ji 2015-1	-5.1	22.4	223	-2	22.55	226	0.6%	-3.10 [-7.26, 1.06]
CANA Ji 2015-2	-5.5	22.6	227	-2	22.55	226	0.6%	-3.50 [-7.66, 0.66]
CANA Lavalle-González 2013-2	-5.06	11.59	367	1.52	11.21	183	1.0%	-6.58 [-8.59, -4.57]
CANA Leiter 2014-2	-3.1	13.21	485	1.7	13.17	482	1.1%	-4.80 [-6.46, -3.14]
CANA Wilding 2013-1	-3.7	12.53	157	0.1	12.49	156	0.9%	-3.80 [-6.57, -1.03]
CANA Wilding 2013-2	-2.9	12.49	156	0.1	12.49	156	0.9%	-3.00 [-5.77, -0.23]
DAPA Jabbour 2018	-4.5	12.08	228	-0.7	13.56	227	1.0%	-3.80 [-6.16, -1.44]
DAPA Jeon 2018	-4.92	1.11	162	1.36	1.01	148	1.3%	-6.28 [-6.52, -6.04]
DAPA Ji 2014-1	-1.2	13.58	128	0.8	13.79	132	0.7%	-2.00 [-5.33, 1.33]
DAPA Ji 2014-2	-2.3	12.69	133	0.8	13.79	132	0.8%	-3.10 [-6.29, 0.09]
DAPA Rosenstock 2015	-1.9	12	179	0.3	10.2	176	1.0%	-2.20 [-4.52, 0.12]
DAPA Wilding 2012-1	-5.3	14.18	202	-1.49	14.6	193	0.9%	-3.81 [-6.65, -0.97]
DAPA Wilding 2012-2	-4.33	14.45	211	-1.49	14.6	193	0.9%	-2.84 [-5.68, -0.00]
DAPA Wilding 2012-3	-4.09	14.21	194	-1.49	14.6	193	0.8%	-2.60 [-5.47, 0.27]
EMPA Ferdinand 2019	-10.26	15.01	78	-2.83	15.44	72	0.5%	-7.43 [-12.31, -2.55]
EMPA Ferrannini 2013-1-1	0.1	15.06	80	2	14.32	56	0.5%	-1.90 [-6.90, 3.10]
EMPA Ferrannini 2013-1-2	-1.7	15.55	88	2	14.32	56	0.5%	-3.70 [-8.66, 1.26]
EMPA Ferrannini 2013-2-1	-3.3	14.33	137	1.8	12.79	56	0.6%	-5.10 [-9.22, -0.98]
EMPA Ferrannini 2013-2-2	-3	14.14	139	1.8	12.79	56	0.6%	-4.80 [-8.89, -0.71]
EMPA Hadjadj 2016-1-1	-2.2	11.42	155	0.8	11.67	140	0.9%	-3.00 [-5.64, -0.36]
EMPA Hadjadj 2016-1-2	-3.2	11.56	149	0.8	11.67	140	0.9%	-4.00 [-6.68, -1.32]
EMPA Hadjadj 2016-2-1	-2.9	11.63	150	-0.2	11.53	148	0.9%	-2.70 [-5.33, -0.07]
EMPA Hadjadj 2016-2-2	-3.2	11.7	160	-0.2	11.53	148	0.9%	-3.00 [-5.60, -0.40]
ERTU Ji 2019-1	-5.09	11.24	170	0.22	11.83	167	0.9%	-5.31 [-7.77, -2.85]
ERTU Ji 2019-2	-3.87	11.34	169	0.22	11.83	167	0.9%	-4.09 [-6.57, -1.61]
ERTU Miller 2018-1	-2.04	11.09	98	2.41	13.79	97	0.7%	-4.45 [-7.96, -0.94]
ERTU Pratley 2018-1	-3.4	11.13	243	-0.7	11.63	247	1.0%	-2.70 [-4.72, -0.68]
ERTU Pratley 2018-2	-3.7	11.16	244	-0.7	11.63	247	1.0%	-3.00 [-5.02, -0.98]
ERTU Rosenstock 2018-2	-5.2	12.16	205	-0.7	12.98	209	0.9%	-4.50 [-6.92, -2.08]
IPRA Lu 2016	-6.8	14.1	87	-1.3	12.9	83	0.6%	-5.50 [-9.56, -1.44]
SOTA Danne 2018-1	-2.3	11.47	261	0.6	11.4	258	1.1%	-2.90 [-4.87, -0.93]
SOTA Danne 2018-2	-2.2	11.35	263	0.6	11.4	258	1.1%	-2.80 [-4.75, -0.85]
SOTA Garg 2017	-3.5	11.63	699	0.3	11.4	703	1.2%	-3.80 [-5.01, -2.59]
SOTA Zambrowicz 2012-1	-10	18.47	12	-4	8	12	0.1%	-6.00 [-17.39, 5.39]
SOTA Zambrowicz 2012-2	-13	15.84	12	-4	8	12	0.2%	-9.00 [-19.04, 1.04]
**Subtotal (95% CI)**			**6651**			**6155**	**28.6%**	**-3.87 [-4.60, -3.14]**
Heterogeneity: Tau² = 2.63; Chi² = 124.80, df = 34 (P < 0.00001); I² = 73%								
Test for overall effect: Z = 10.37 (P < 0.00001)								
**9.1.2 eGFR 60-89**								
CANA Bode 2015-1	-1.2	17.08	241	4.5	16.93	237	0.8%	-5.70 [-8.75, -2.65]
CANA Bode 2015-2	-3	16.9	236	4.5	16.93	237	0.8%	-7.50 [-10.55, -4.45]
CANA Forst 2014-1	-5.3	11.01	113	-1.24	11.08	115	0.8%	-4.06 [-6.93, -1.19]
CANA Forst 2014-2	-4.7	11.15	114	-1.24	11.08	115	0.8%	-3.46 [-6.34, -0.58]
CANA Inagaki 2013-1	-5.8	10.87	82	-1.2	10.39	75	0.7%	-4.60 [-7.93, -1.27]
CANA Inagaki 2013-2	-7.1	10.32	74	-1.2	10.39	75	0.7%	-5.90 [-9.23, -2.57]
CANA Inagaki 2013-3	-9.3	10.46	76	-1.2	10.39	75	0.7%	-8.10 [-11.43, -4.77]
CANA Inagaki 2013-4	-8.7	10.39	75	-1.2	10.39	75	0.7%	-7.50 [-10.83, -4.17]
CANA Inagaki 2014-1	-7.88	10.25	90	-2.72	10.22	93	0.8%	-5.16 [-8.13, -2.19]
CANA Inagaki 2014-2	-6.24	10.23	88	-2.72	10.22	93	0.8%	-3.52 [-6.50, -0.54]
CANA Inagaki 2016	-3.58	9.938	76	-0.4	9.956	70	0.8%	-3.18 [-6.41, 0.05]
CANA Lavalle-González 2013-1	-3.84	11.55	368	1.52	11.21	183	1.0%	-5.36 [-7.37, -3.35]
CANA Leiter 2014-1	-2	13.19	483	1.7	13.17	482	1.1%	-3.70 [-5.36, -2.04]
CANA Rosenstock 2016-1	-1.7	9.237	237	-0.3	9.237	237	1.1%	-1.40 [-3.06, 0.26]
CANA Rosenstock 2016-2	-2.2	9.237	237	-0.3	9.237	237	1.1%	-1.90 [-3.56, -0.24]
CANA Schernthaner 2013	-5.06	12.74	377	0.85	12.95	378	1.1%	-5.91 [-7.74, -4.08]
CANA Stenlof 2013-1	-5.04	10.79	197	0.38	10.81	192	1.0%	-5.42 [-7.57, -3.27]
CANA Stenlof 2013-2	-3.34	10.82	195	0.38	10.81	192	1.0%	-3.72 [-5.88, -1.56]
DAPA Araki 2017	-3	12.68	122	-6.8	18.38	60	0.5%	3.80 [-1.37, 8.97]
DAPA Bailey 2015-1	-1.7	13.16	65	2.1	18.56	75	0.4%	-3.80 [-9.08, 1.48]
DAPA Bailey 2015-2	1.9	16.73	64	2.1	18.56	75	0.4%	-0.20 [-6.07, 5.67]
DAPA Bailey 2015-3	3.9	14.73	70	2.1	18.56	75	0.4%	1.80 [-3.64, 7.24]
DAPA Cho 2018	-3.4	15.02	36	1.2	14.36	35	0.3%	-4.60 [-11.43, 2.23]
DAPA Hayashi 2017	-4.2	17	40	-2.2	19.41	40	0.2%	-2.00 [-10.00, 6.00]
DAPA McMurray 2019	-1.92	14.92	2373	-0.38	15.27	2371	1.3%	-1.54 [-2.40, -0.68]
DAPA Müller-Wieland 2018-1	-5.6	14.18	314	-1.6	14.15	313	1.0%	-4.00 [-6.22, -1.78]
DAPA Müller-Wieland 2018-2	-6.4	12.36	312	-1.6	14.15	313	1.0%	-4.80 [-6.88, -2.72]
DAPA Schumm-Draeger 2015-1	-3.5	12.25	99	-1.6	10.57	101	0.8%	-1.90 [-5.07, 1.27]
DAPA Schumm-Draeger 2015-2	-3	10.9	100	-1.6	10.57	101	0.8%	-1.40 [-4.37, 1.57]
DAPA Schumm-Draeger 2015-3	-3.4	11.1	99	-1.6	10.57	101	0.8%	-1.80 [-4.81, 1.21]
DAPA Weber 2016	-11.9	15.88	225	-7.62	16.02	224	0.8%	-4.28 [-7.23, -1.33]
DAPA Wilding 2009-1	-7.2	12.25	24	2.8	18.7	23	0.2%	-10.00 [-19.08, -0.92]
DAPA Wilding 2009-2	-6.1	12.25	24	2.8	18.7	23	0.2%	-8.90 [-17.98, 0.18]
DAPA Yang 2018	-4.5	12.03	139	0.5	12.57	133	0.8%	-5.00 [-7.93, -2.07]
EMPA Araki 2015-1	-6.5	11.66	136	-0.2	11.11	63	0.7%	-6.30 [-9.67, -2.93]
EMPA Araki 2015-2	-7.4	11.7	137	-0.2	11.11	63	0.7%	-7.20 [-10.57, -3.83]
EMPA Barnett 2014-1	-6.2	12.81	97	1.6	12.93	95	0.7%	-7.80 [-11.44, -4.16]
EMPA Barnett 2014-2	-1.7	13.16	98	1.6	12.93	95	0.7%	-3.30 [-6.98, 0.38]
EMPA Haring 2013-1	-4.1	10.5	225	-1.4	10.5	225	1.1%	-2.70 [-4.64, -0.76]
EMPA Haring 2013-2	-3.5	10.29	216	-1.4	10.5	225	1.1%	-2.10 [-4.04, -0.16]
EMPA Haring 2014-1	-4.5	10.31	217	-0.4	10.07	207	1.1%	-4.10 [-6.04, -2.16]
EMPA Haring 2014-2	-5.2	10.22	213	-0.4	10.07	207	1.1%	-4.80 [-6.74, -2.86]
EMPA Hattori 2018	-9.5	23.55	51	0.7	15.6	51	0.3%	-10.20 [-17.95, -2.45]
EMPA Kadowaki 2014-1	-2.85	16.15	110	-1.38	15.56	109	0.6%	-1.47 [-5.67, 2.73]
EMPA Kadowaki 2014-2	-5.57	15.03	109	-1.38	15.56	109	0.6%	-4.19 [-8.25, -0.13]
EMPA Kadowaki 2014-3	-4.6	15.87	109	-1.38	15.56	109	0.6%	-3.22 [-7.39, 0.95]
EMPA Kadowaki 2014-4	-5.21	16.15	110	-1.38	15.56	109	0.6%	-3.83 [-8.03, 0.37]
EMPA Kario 2018	-10	14.09	68	-2.4	13.97	63	0.5%	-7.60 [-12.41, -2.79]
EMPA Kovacs 2014-1	-3.1	11.56	165	0.7	11.56	165	0.9%	-3.80 [-6.29, -1.31]
EMPA Kovacs 2014-2	-4	10.37	168	0.7	11.56	165	1.0%	-4.70 [-7.06, -2.34]
EMPA Packer 2020	-2.4	17.26	1863	-1.7	17.28	1867	1.2%	-0.70 [-1.81, 0.41]
EMPA Roden 2013-1	-2.9	12.22	224	-0.3	12.33	228	1.0%	-2.60 [-4.86, -0.34]
EMPA Roden 2013-2	-3.7	12.22	224	-0.3	12.33	228	1.0%	-3.40 [-5.66, -1.14]
EMPA Rosenstock 2014-1	-3.4	13.64	186	-2.9	13.71	188	0.9%	-0.50 [-3.27, 2.27]
EMPA Rosenstock 2014-2	-3.8	13.75	189	-2.9	13.71	188	0.9%	-0.90 [-3.67, 1.87]
EMPA Rosenstock 2015-1	-4.1	13	169	0.1	13.04	170	0.9%	-4.20 [-6.97, -1.43]
EMPA Rosenstock 2015-2	-2.4	13.69	155	0.1	13.04	170	0.8%	-2.50 [-5.41, 0.41]
EMPA Ross 2015-1	-4.1	10.26	215	1.6	11.38	107	0.9%	-5.70 [-8.26, -3.14]
EMPA Ross 2015-2	-3.8	10.24	214	1.6	11.38	107	0.9%	-5.40 [-7.96, -2.84]
EMPA Ross 2015-3	-4.2	10.26	215	1.6	11.38	107	0.9%	-5.80 [-8.36, -3.24]
EMPA Ross 2015-4	-2.5	11.7	214	1.6	11.38	107	0.9%	-4.10 [-6.77, -1.43]
ERTU Dagogo-Jack 2018-1	-4.2	11.79	156	0.8	14.2	153	0.8%	-5.00 [-7.91, -2.09]
ERTU Dagogo-Jack 2018-2	-4.1	11.99	153	0.8	14.2	153	0.8%	-4.90 [-7.84, -1.96]
ERTU Hollander 2019-1	-2	13.99	445	2.1	13.3	435	1.1%	-4.10 [-5.90, -2.30]
ERTU Hollander 2019-2	-1.2	13.3	435	2.1	13.3	435	1.1%	-3.30 [-5.07, -1.53]
ERTU Miller 2018-2	-3.98	11.02	96	2.41	13.79	97	0.7%	-6.39 [-9.91, -2.87]
ERTU Rosenstock 2018-1	-4.38	11.97	207	-0.7	12.98	209	1.0%	-3.68 [-6.08, -1.28]
ERTU Terra 2017-1	-5.54	11.34	156	-2.22	13.13	153	0.9%	-3.32 [-6.06, -0.58]
ERTU Terra 2017-2	-3.93	11.39	152	-2.22	13.13	153	0.9%	-1.71 [-4.47, 1.05]
IPRA Bando 2016	-2	14.11	40	1	11	22	0.3%	-3.00 [-9.34, 3.34]
IPRA Han 2018	-2.35	10.47	73	-1.14	9.76	66	0.7%	-1.21 [-4.57, 2.15]
IPRA Ishihara 2016	-1.4	14.5	168	1.5	8.19	87	0.9%	-2.90 [-5.69, -0.11]
IPRA Kashiwagi 2015	-4.6	16.17	118	-2.7	10.08	46	0.6%	-1.90 [-6.02, 2.22]
IPRA Tsurutani 2018	-2.7	15.22	60	-0.3	15.48	59	0.4%	-2.40 [-7.92, 3.12]
LUSEO Seino 2018	-2.9	13.51	159	1.2	11.63	74	0.7%	-4.10 [-7.48, -0.72]
SOTA Buse 2018-1	-1.1	11.84	263	1.7	12.11	268	1.0%	-2.80 [-4.84, -0.76]
SOTA Buse 2018-2	-2.7	11.98	262	1.7	12.11	268	1.0%	-4.40 [-6.45, -2.35]
TOFO Kaku 2014-1	-6.8	13.1	57	-3.2	13.1	56	0.5%	-3.60 [-8.43, 1.23]
TOFO Kaku 2014-2	-7.6	11.4	58	-3.2	13.1	56	0.5%	-4.40 [-8.91, 0.11]
TOFO Kaku 2014-3	-9.4	11.1	58	-3.2	13.1	56	0.6%	-6.20 [-10.66, -1.74]
TOFO Terauchi 2017	-4.8	15	141	-2.7	11.8	70	0.7%	-2.10 [-5.81, 1.61]
**Subtotal (95% CI)**			**16819**			**15667**	**64.0%**	**-3.79 [-4.24, -3.34]**
Heterogeneity: Tau² = 1.93; Chi² = 170.70, df = 80 (P < 0.00001); I² = 53%								
Test for overall effect: Z = 16.49 (P < 0.00001)								
**9.1.3 eGFR 45-59**								
CANA Takashima 2018	-3.1	2	21	0.5	2.3	21	1.2%	-3.60 [-4.90, -2.30]
DAPA Fioretto 2018	-4.8	18.97	160	-1.7	19.03	161	0.6%	-3.10 [-7.26, 1.06]
DAPA Pollock 2019	-6.9	20.47	145	-4.1	20.68	148	0.5%	-2.80 [-7.51, 1.91]
EMPA Barnett 2014-3	-5.1	13.95	187	-0.8	13.95	187	0.9%	-4.30 [-7.13, -1.47]
LUSEO Haneda 2016	-0.5	17.4	95	2.1	14.43	50	0.4%	-2.60 [-7.91, 2.71]
**Subtotal (95% CI)**			**608**			**567**	**3.6%**	**-3.58 [-4.67, -2.50]**
Heterogeneity: Tau² = 0.00; Chi² = 0.54, df = 4 (P = 0.97); I² = 0%								
Test for overall effect: Z = 6.48 (P < 0.00001)								
**9.1.4 eGFR＜45**								
CANA Yale 2013-1	-6.1	14.23	90	-0.3	14.23	90	0.6%	-5.80 [-9.96, -1.64]
CANA Yale 2013-2	-6.4	14.15	89	-0.3	14.23	90	0.6%	-6.10 [-10.26, -1.94]
DAPA Kohan 2013-1	-0.25	18.3	83	4.14	14.07	84	0.5%	-4.39 [-9.35, 0.57]
DAPA Kohan 2013-2	-2.51	16.33	85	4.14	14.07	84	0.5%	-6.65 [-11.24, -2.06]
EMPA Barnett 2014-4	-11.2	15.7	37	1	17.4	37	0.3%	-12.20 [-19.75, -4.65]
SOTA Bhatt 2021	-3.54	10.578	5292	-1.1	10.578	5292	1.3%	-2.44 [-2.84, -2.04]
**Subtotal (95% CI)**			**5676**			**5677**	**3.8%**	**-5.36 [-7.98, -2.74]**
Heterogeneity: Tau² = 6.18; Chi² = 15.28, df = 5 (P = 0.009); I² = 67%								
Test for overall effect: Z = 4.01 (P < 0.0001)								
**Total (95% CI)**			**29754**			**28066**	**100.0%**	**-3.87 [-4.30, -3.44]**
Heterogeneity: Tau² = 3.55; Chi² = 594.18, df = 126 (P < 0.00001); I² = 79%								
Test for overall effect: Z = 17.62 (P < 0.00001)								
Test for subgroup differences: Chi² = 1.54, df = 3 (P = 0.67), I² = 0%								

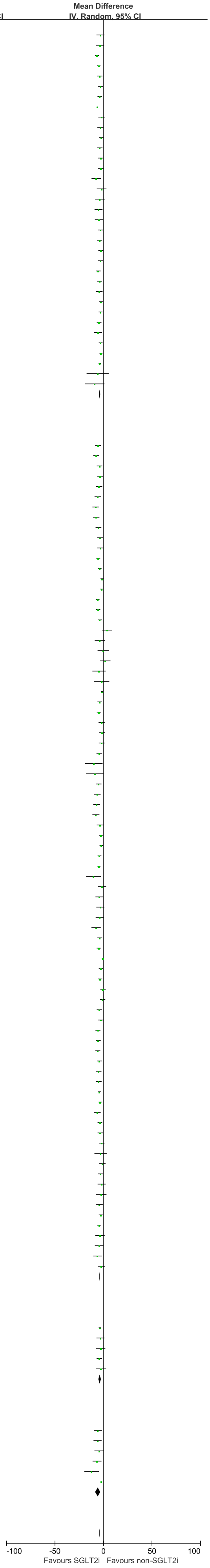

Supplement: Supplementary file 4 [file DataSheet4.PDF]
