# Supplementary material for: Disparities in efficacy and safety of sodium-glucose cotransporter 2 inhibitor among patients with different extents of renal dysfunction: A systematic review and meta-analysis of randomized controlled trials
Source: Front Pharmacol. 2022 Nov 22;13:1018720. doi: 10.3389/fphar.2022.1018720 (PMC9723253; doi:10.3389/fphar.2022.1018720)
Supplement: Supplementary file 8 [file Table1.DOCX]

Supplementary Materials

Supplementary Figure legends

Figure S1. Funnel plots of analyses for efficacy and safety of SGLT2is.

Figure S2. HbA1c changes before and after SGLT2i treatment in patients with different levels of renal function

Figure S3. Weight changes before and after SGLT2i treatment in patients with different levels of renal function

Figure S4. SBP changes before and after SGLT2i treatment in patients with different levels of renal function

Figure S5. DBP changes before and after SGLT2i treatment in patients with different levels of renal function

Figure S6. eGFR changes before and after SGLT2i treatment in patients with different levels of renal function

Figure S7. The associations between the use of SGLT2i treatment and urinary tract infection

Figure S8. The associations between the use of SGLT2i treatment and genital tract infection

Figure S9. The associations between the use of SGLT2i treatment and amputation

Figure S10. The associations between the use of SGLT2i treatment and hypovolemia

Figure S11. The associations between the use of SGLT2i treatment and orthostatic hypotension

Figure S12. The associations between the use of SGLT2i treatment and bone fracture

Figure S13. The associations between the use of SGLT2i treatment and diabetic ketoacidosis

Figure S14. The associations between the use of SGLT2i treatment and hypoglycemia

Supplementary Table legend

Table S1. Baseline characteristics of included randomized controlled trials.

Table S2. Evaluation for risk of bias in included RCTs.

Table S3. Subgroup analyses of efficacy among different drug categories.

Table S4. Subgroup analyses of efficacy among follow-up period.

Table S5. Subgroup analyses of different AEs.

Table S6. Subgroup analyses of different AEs in placebo-controlled RCTs.

Table S7. Efficacy of SGLT2i treatment in patients with different levels of renal function in placebo-controlled RCTs.

Table S8. Efficacy of SGLT2i treatment in patients with different levels of renal function (follow-up period ≥12 weeks).

Figure S1. Funnel plots of analyses for efficacy and safety of SGLT2is.

| 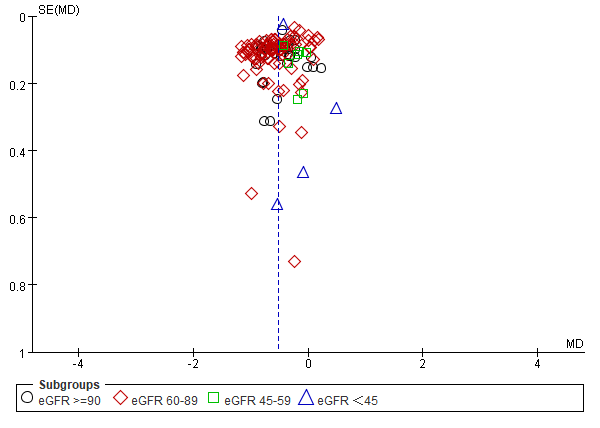 | 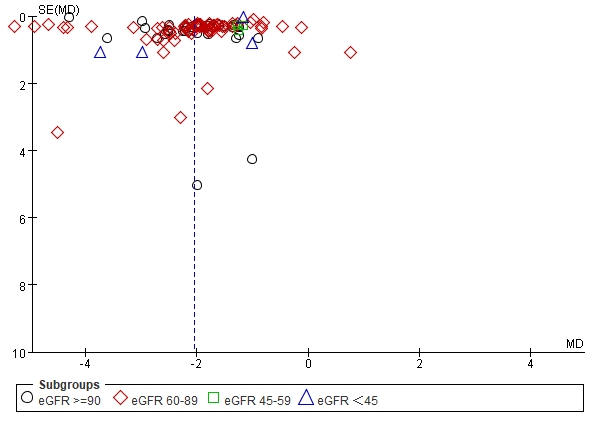 | 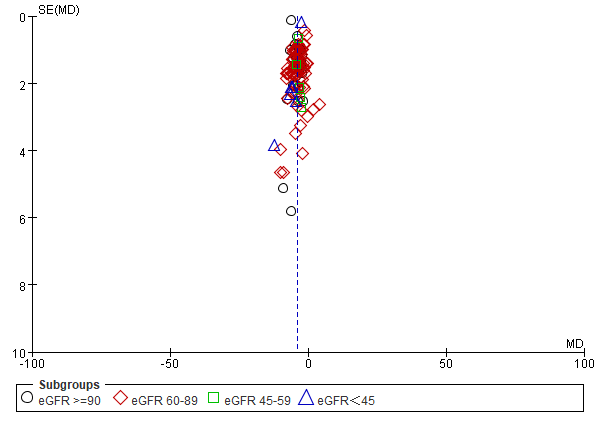 |
| --- | --- | --- |
| (a) HbA1c changes | (b) Weight changes | (c) SBP changes |
| 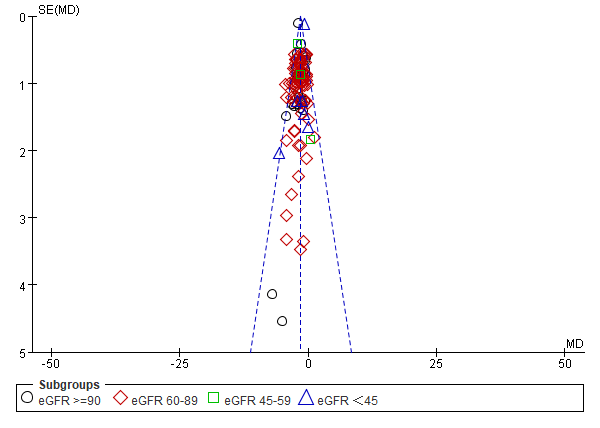 | 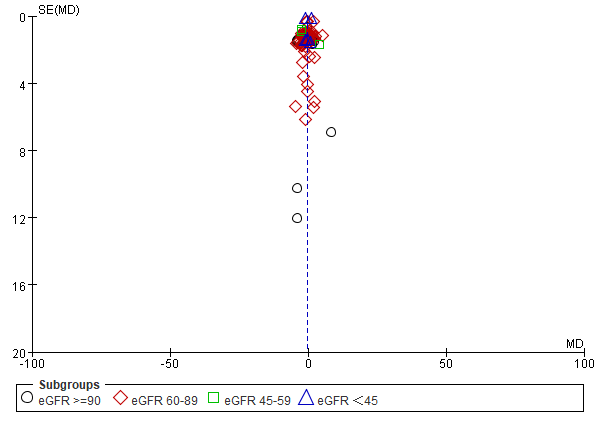 |  |
| (d) DBP changes | (e) eGFR changes |  |

| 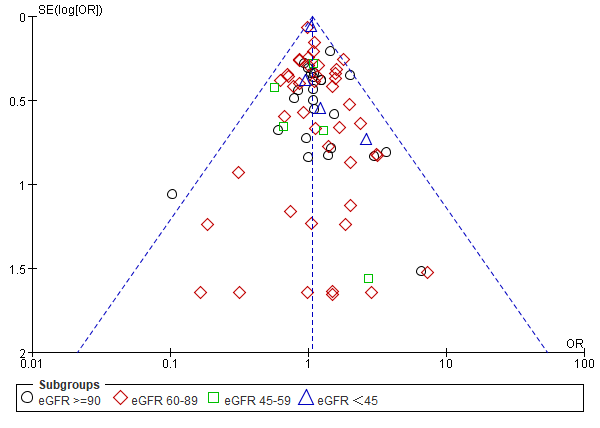 | 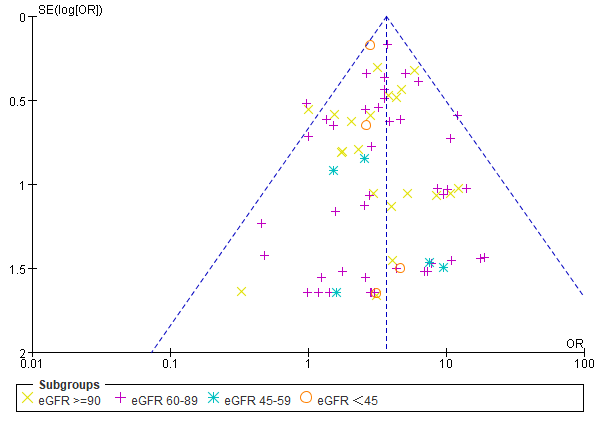 | 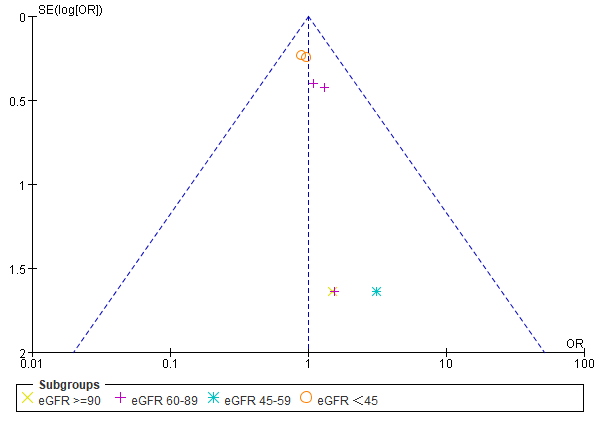 |
| --- | --- | --- |
| (f) urinary tract infection | (g) genital tract infection | (h) amputation |
| 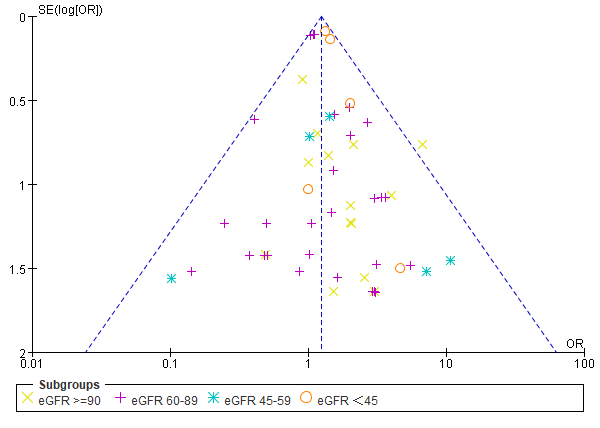 | 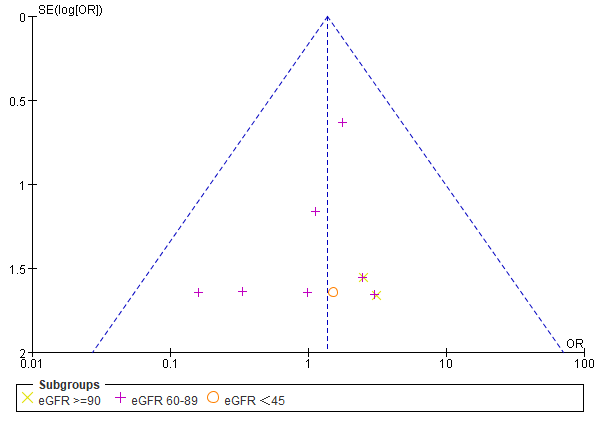 | 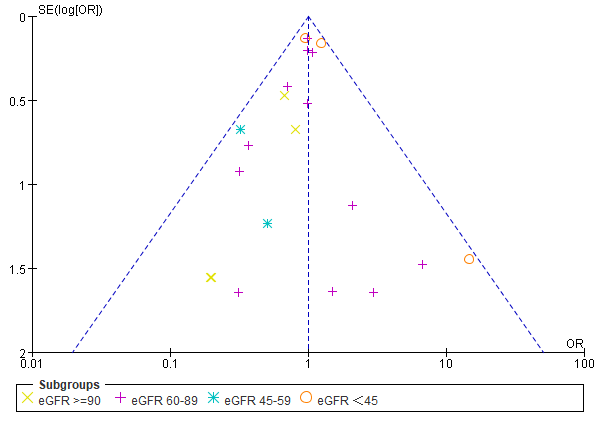 |
| (i) hypovolemia | (j) orthostatic hypotension | (k) bone fracture |
| 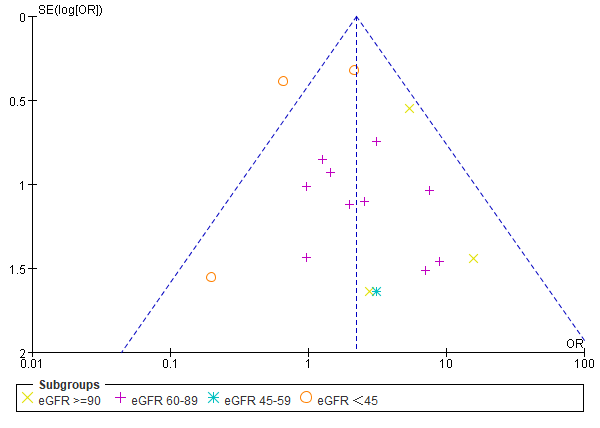 | 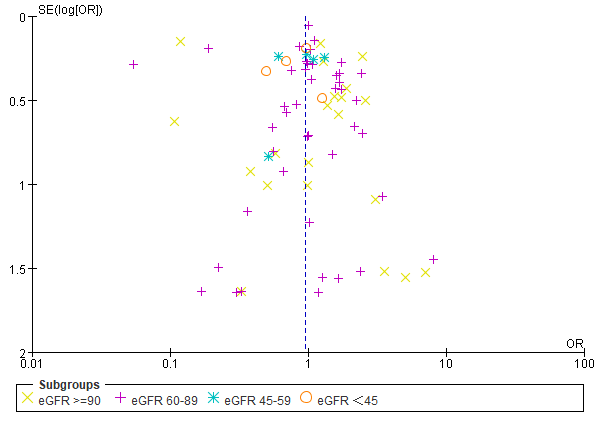 |  |
| (l) diabetic ketoacidosis(DKA) | (m) hypoglycemia |  |

Figure S2. HbA1c changes before and after SGLT2i treatment in patients with different levels of renal function.


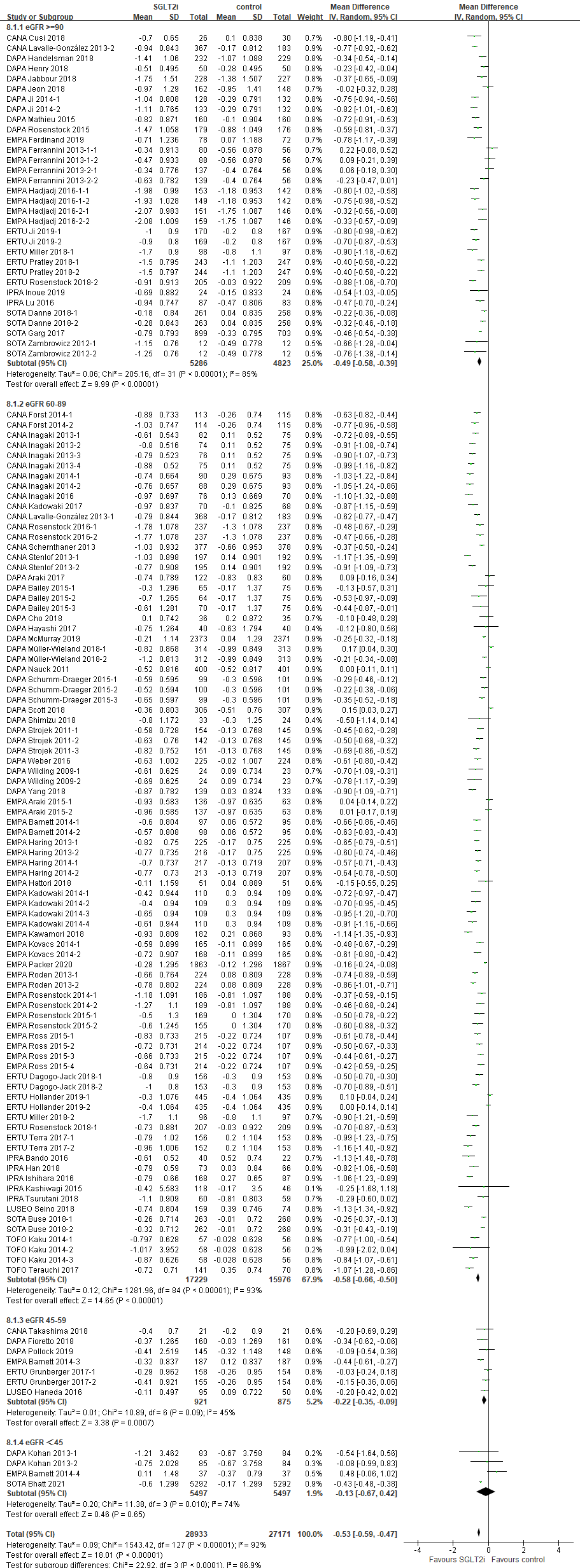


Figure S3. Weight changes before and after SGLT2i treatment in patients with different levels of renal function.


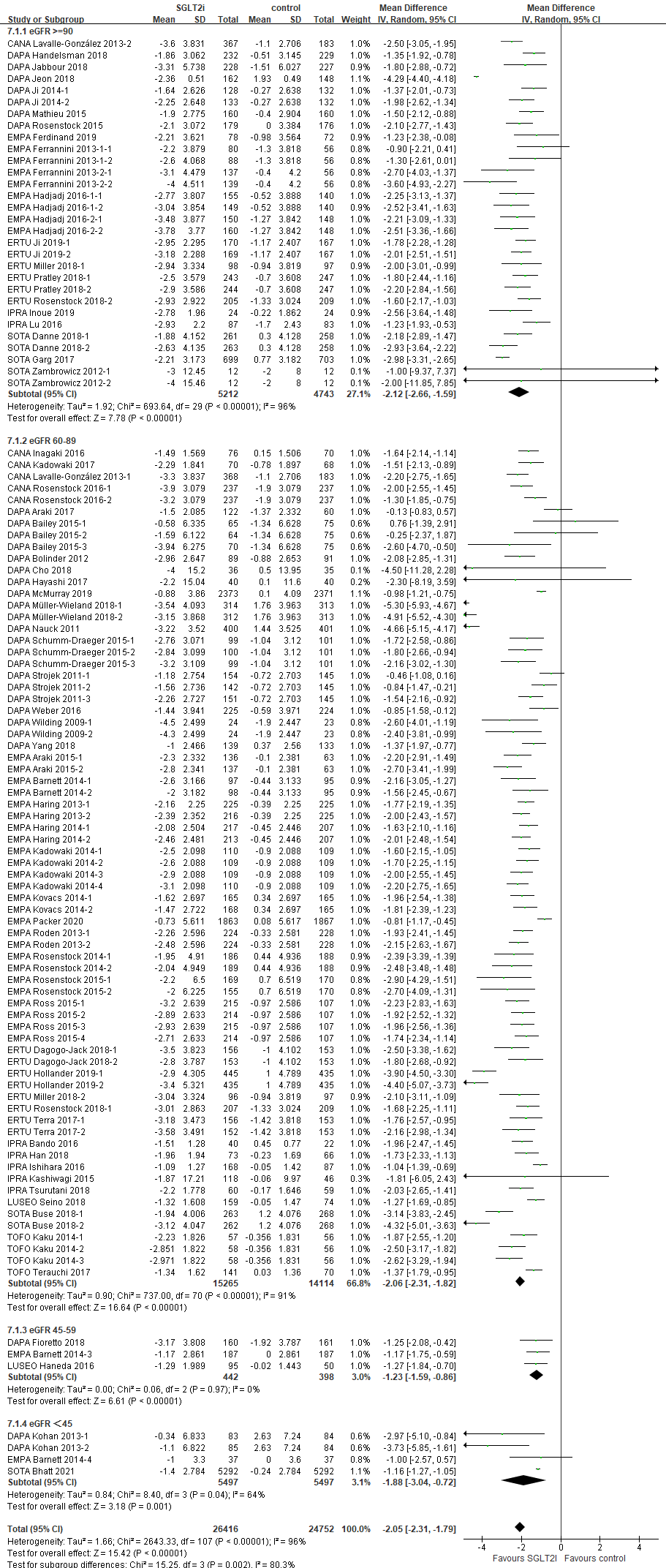


Figure S4. SBP changes before and after SGLT2i treatment in patients with different levels of renal function.


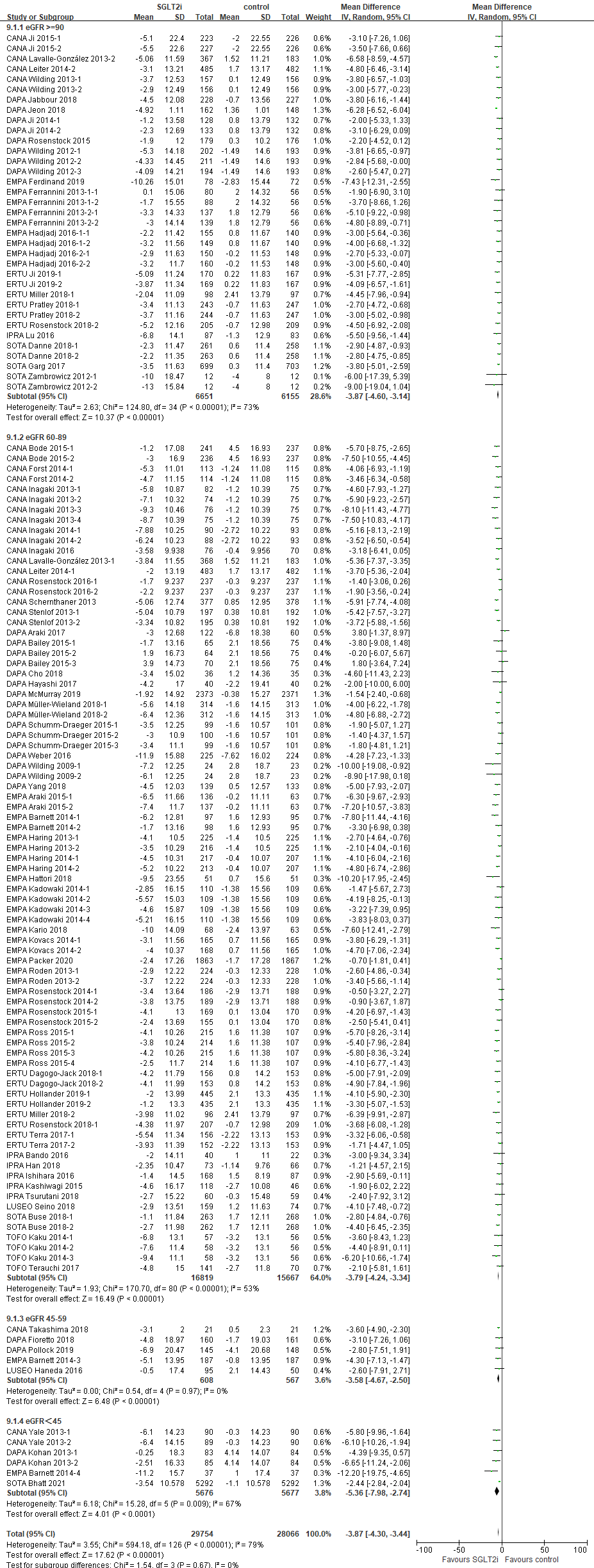


Figure S5. DBP changes before and after SGLT2i treatment in patients with different levels of renal function.


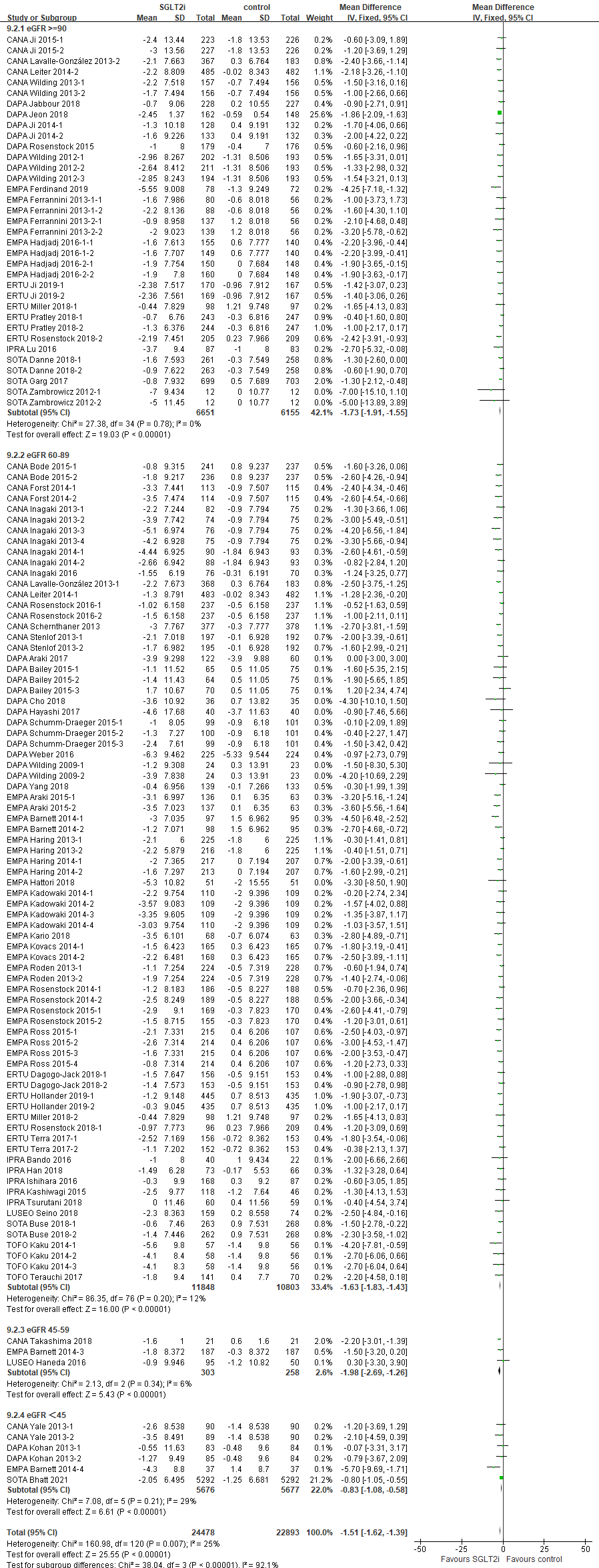


Figure S6. eGFR changes before and after SGLT2i treatment in patients with different levels of renal function.


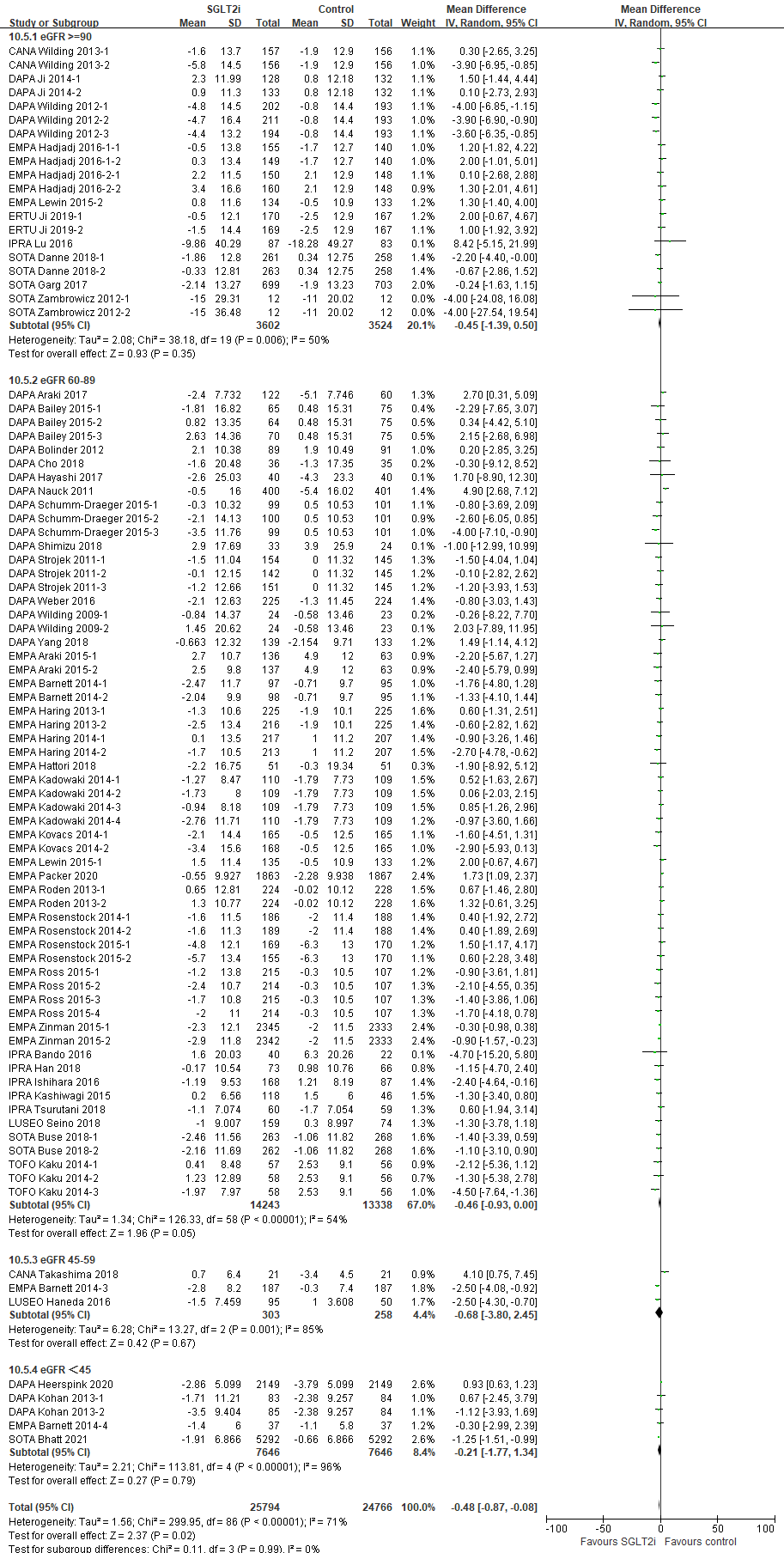


Figure S7. The associations between the use of SGLT2i treatment and urinary tract infection.


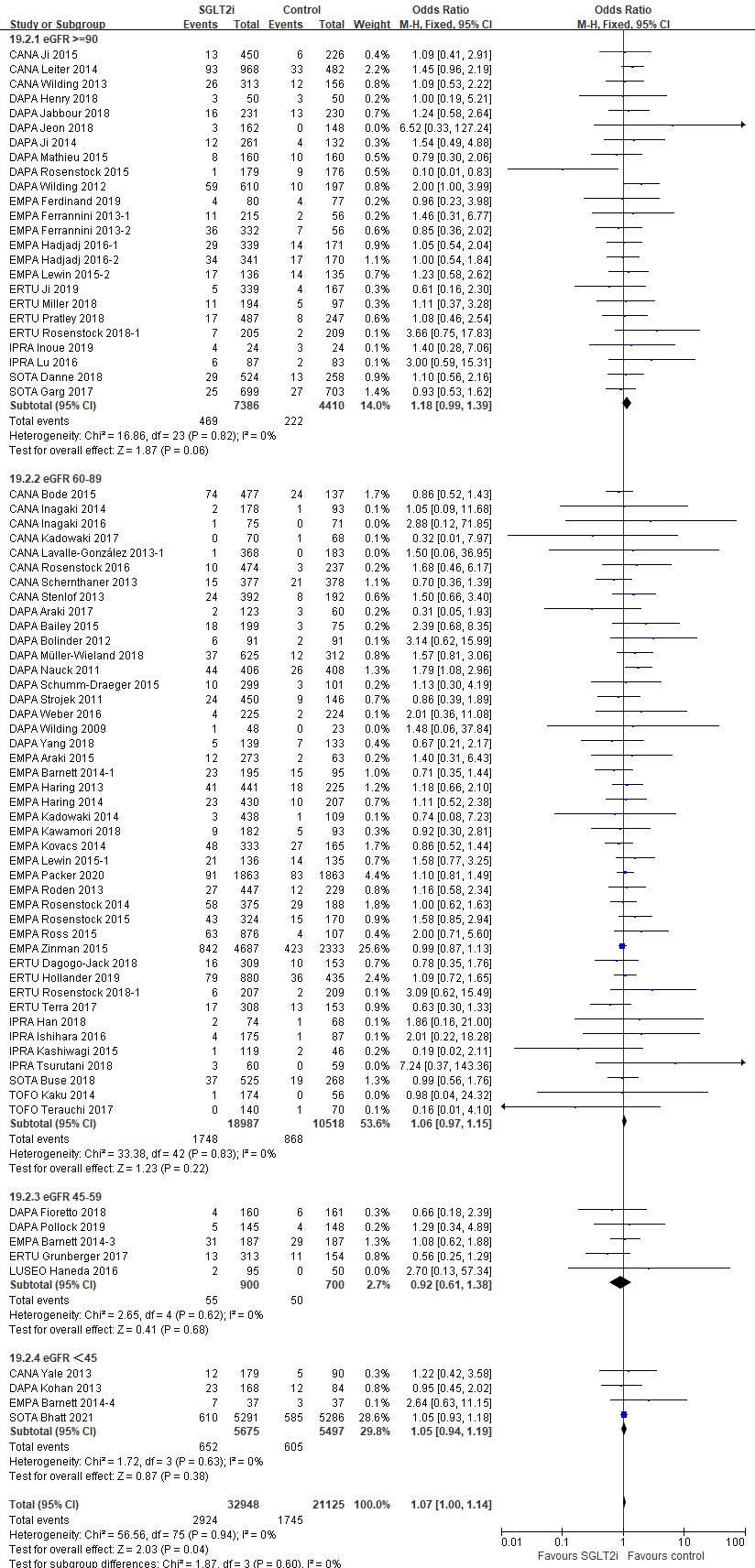


Figure S8. The associations between the use of SGLT2i treatment and genital tract infection.


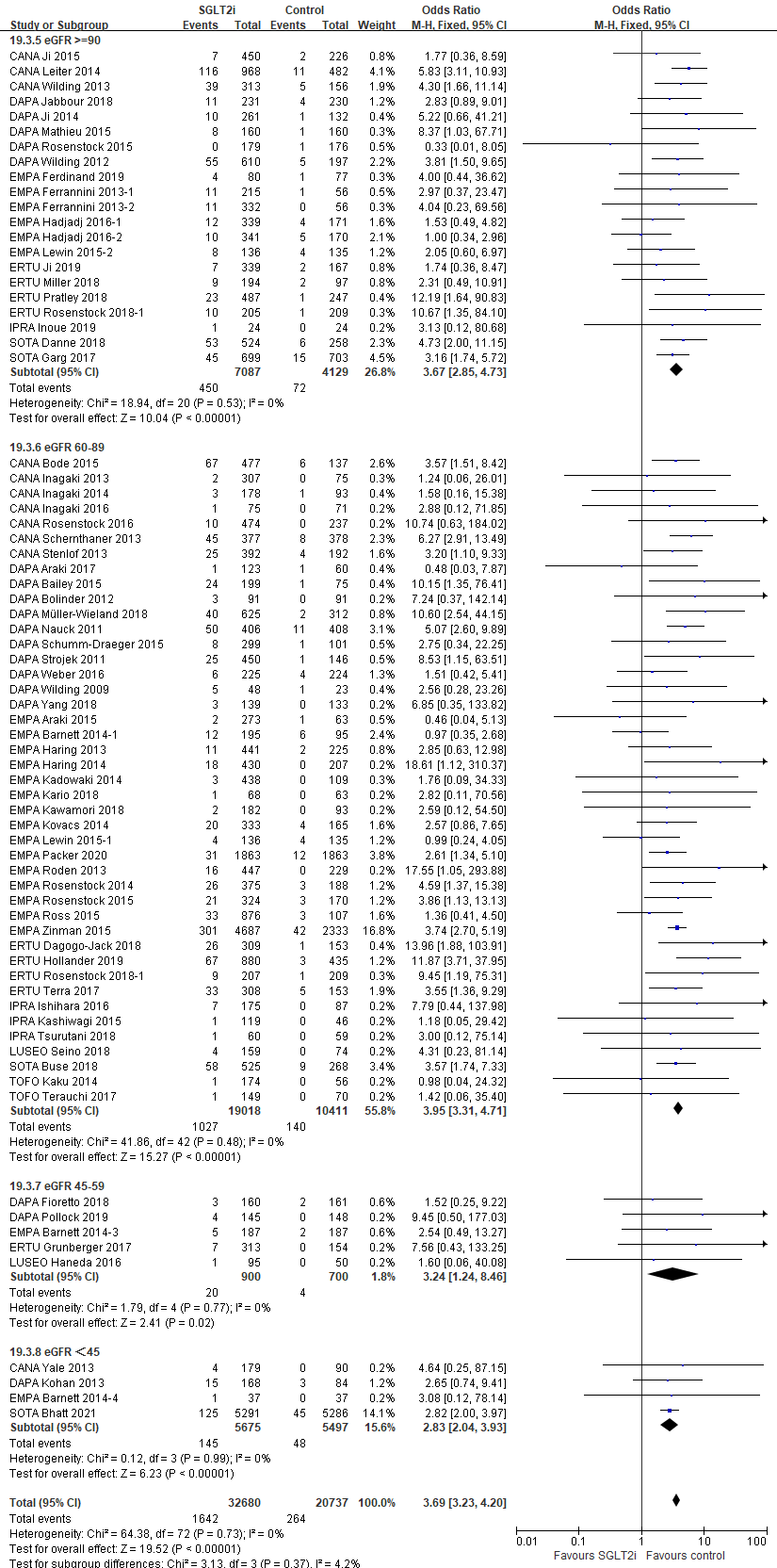


Figure S9. The associations between the use of SGLT2i treatment and amputation.


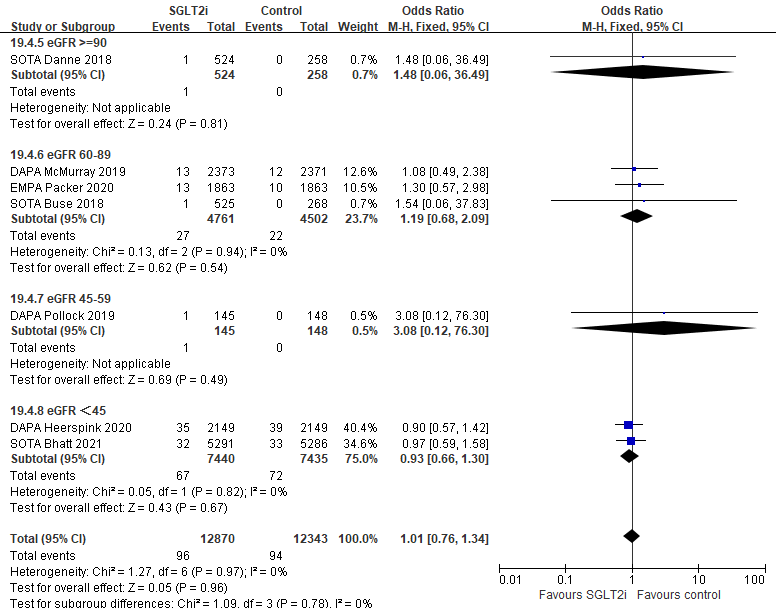


Figure S10. The associations between the use of SGLT2i treatment and hypovolemia.


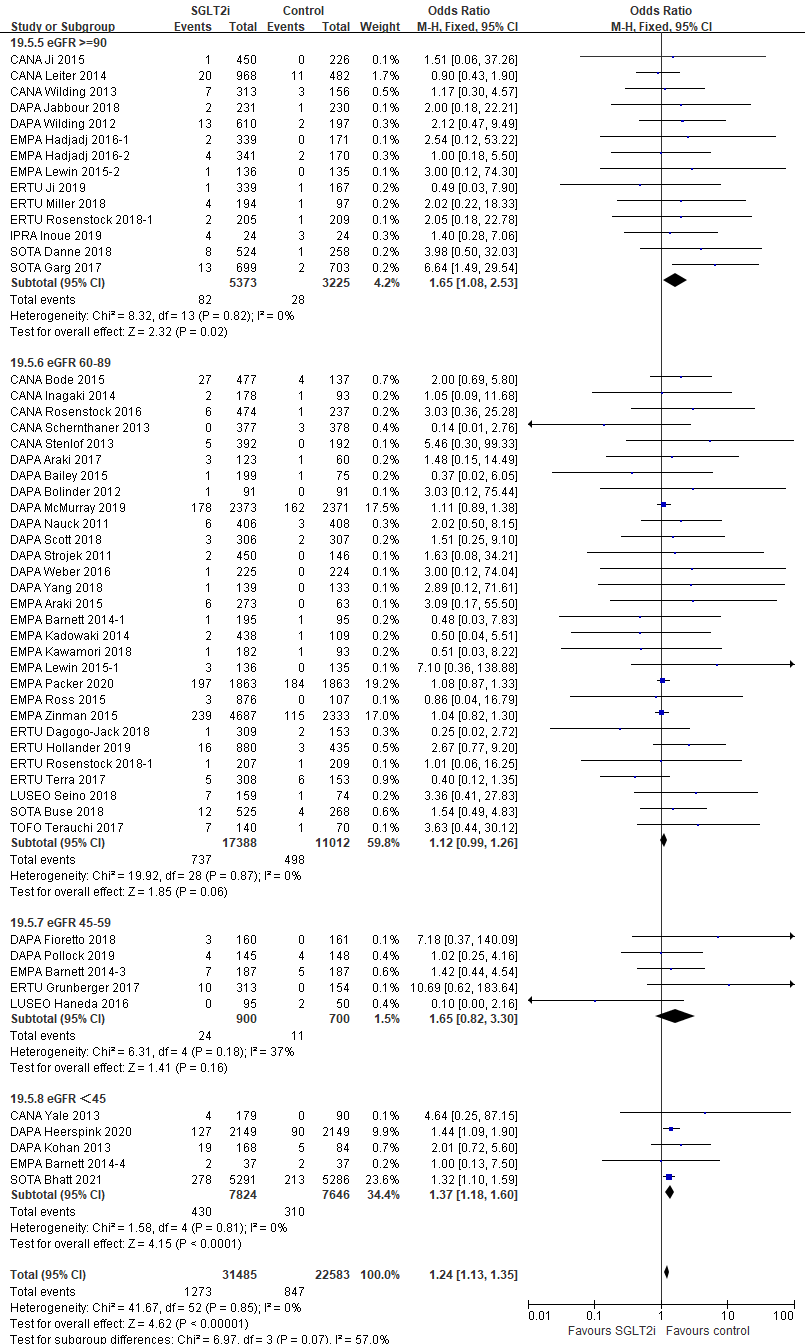


Figure S11. The associations between the use of SGLT2i treatment and orthostatic hypotension.


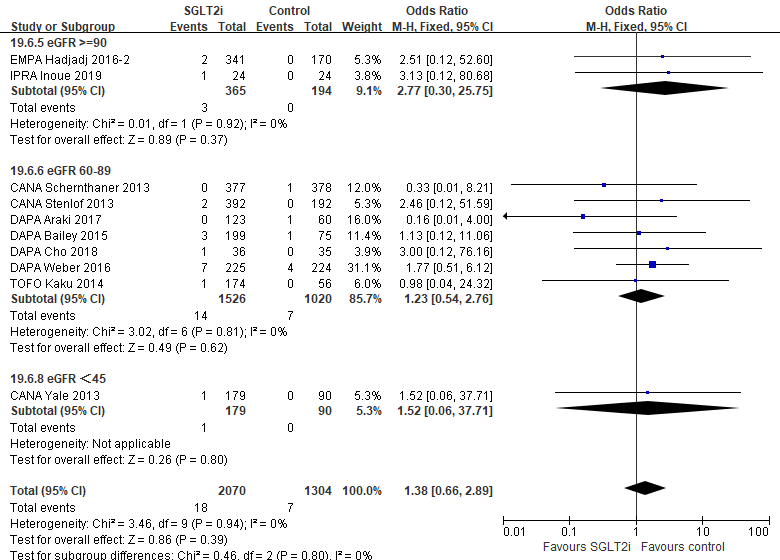


Figure S12. The associations between the use of SGLT2i treatment and bone fracture.


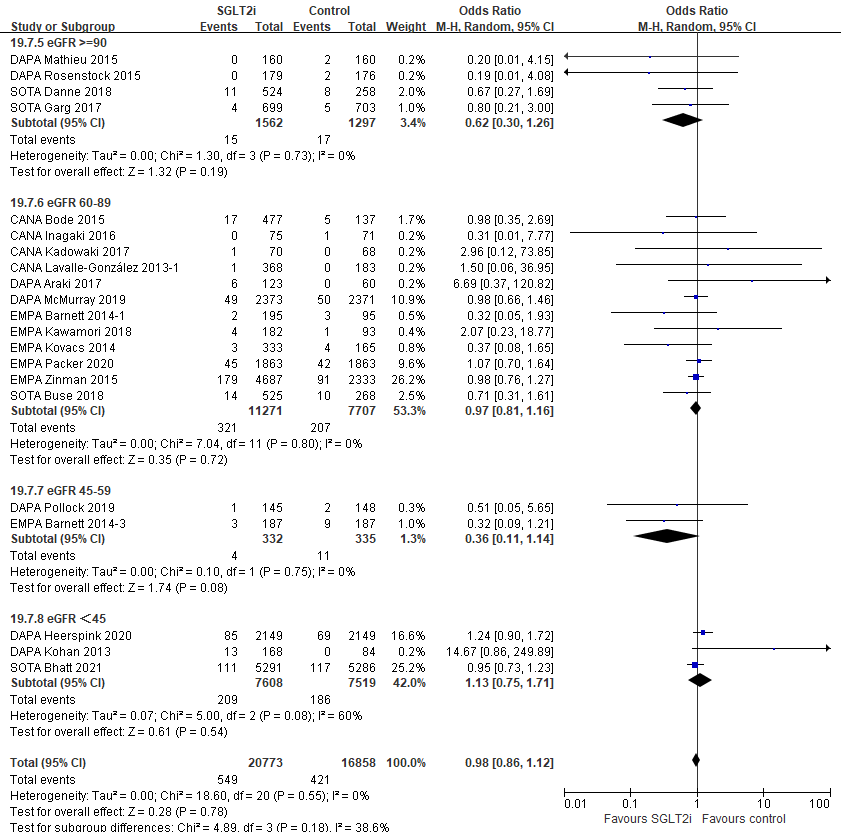


Figure S13. The associations between the use of SGLT2i treatment and diabetic ketoacidosis (DKA).


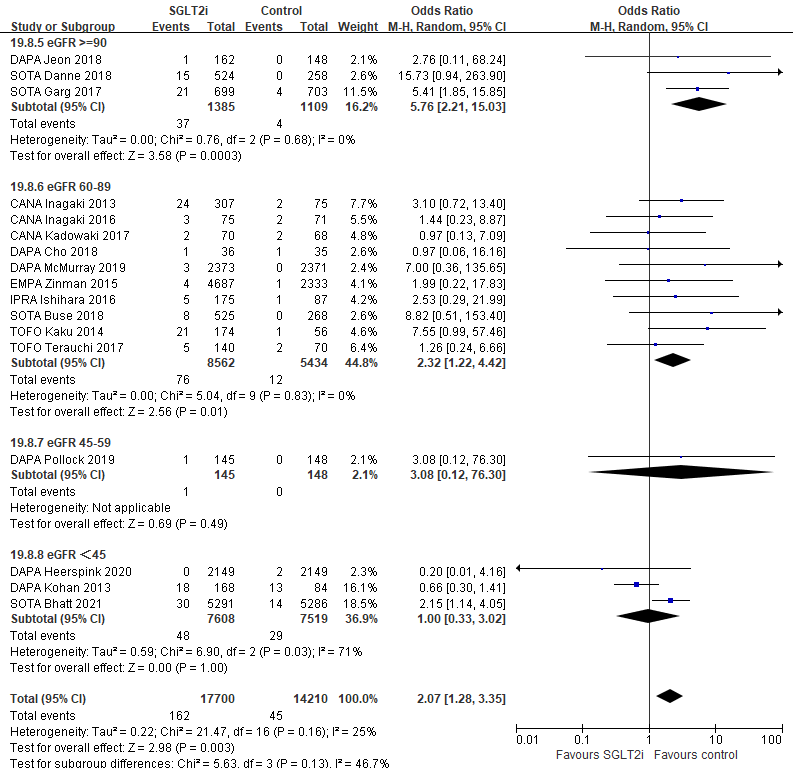


Figure S14. The associations between the use of SGLT2i treatment and hypoglycemia.


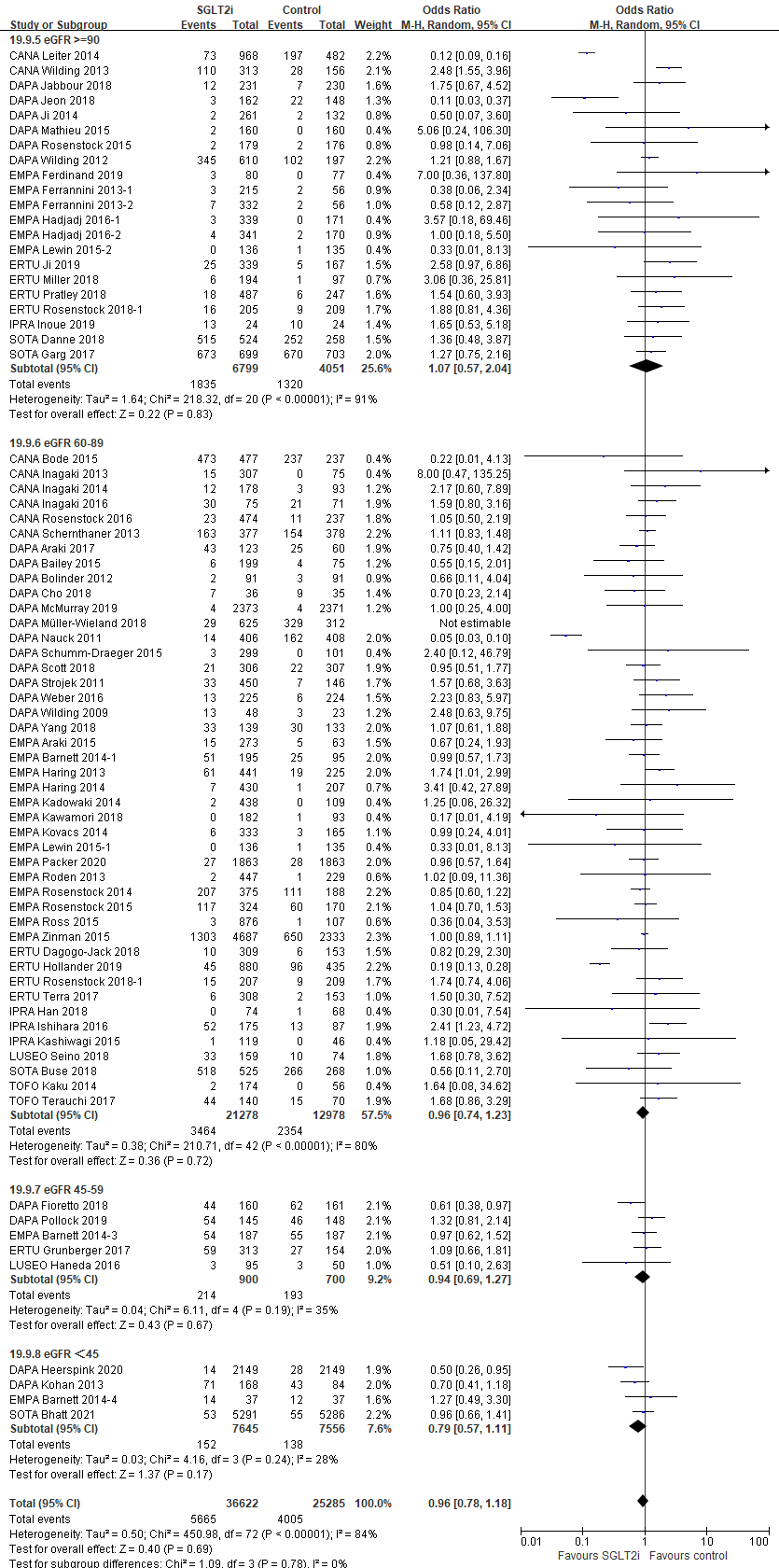


Table S1. Baseline characteristics of included RCTs.

| First author, year | Condition | Study duration(w) | Treatment group | Dosage | No. of patients | Age(year) | Male percentage (%) | Baseline eGFR(ml/min/1.73m^2^) |
| --- | --- | --- | --- | --- | --- | --- | --- | --- |
| baseline eGFR >= 90 ml/min/1.73m^2^ | | | |  |  |  |  |  |
| Cusi 2018^1^ | T2D | 24 | cana | 300mg | 26 | 58.00 | 62.00 | 92.00±16.00 |
|  |  |  | pbo |  | 30 | 58.00 | 70.00 | 89.00±17.00 |
| Ji 2015^2^ | T2D | 18 | cana | 100mg | 223 | 56.50 | 55.30 | 92.50±18.30 |
|  |  |  | cana | 300mg | 227 | 56.40 | 49.80 | 93.90±18.00 |
|  |  |  | pbo |  | 226 | 55.80 | 55.30 | 95.40±18.20 |
| Lavalle-González 2013^3^ | T2D | 26 | cana | 300mg | 367 | 55.30 | 45.00 | 90.20± / |
|  |  |  | pbo |  | 183 | 55.30 | 51.40 | 87.7± / |
| Wilding 2013^4^ | T2D | 52 | cana | 100mg qd | 157 | 57.40 | 48.40 | 91.00± / |
|  |  |  | cana | 300mg qd | 156 | 56.10 | 55.80 | 91.90± / |
|  |  |  | pbo |  | 156 | 56.80 | 48.70 | 87.40± / |
| Henry 2018^5^ | T2D | 4 | dapa | 10mg | 50 | 56.90 | 52.00 | 91.40±22.00 |
|  |  |  | pbo |  | 50 | 56.80 | 50.00 | 89.70±23.60 |
| Jabbour 2018^6^ | T2D | 52 | dapa | 10mg | 228 | 53.80 | 44.70 | 97.70±23.70 |
|  |  |  | pbo |  | 227 | 54.20 | 51.10 | 99.40±26.80 |
| Ji 2014^7^ | T2D | 24 | dapa | 5mg | 128 | 53.00 | 65.60 | 91.60±17.10 |
|  |  |  | dapa | 10mg | 133 | 51.20 | 64.70 | 91.70±20.20 |
|  |  |  | pbo |  | 132 | 49.90 | 65.90 | 94.10±17.70 |
| Mathieu 2015^8^ | T2D | 24 | dapa | 10mg | 160 | 55.20 | 43.70 | 93.50±20.80 |
|  |  |  | pbo |  | 160 | 55.00 | 47.50 | 91.60±23.20 |
| Rosenstock 2015^9^ | T2D | 24 | dapa | 10mg | 179 | 53.00 | 47.00 | 96.60±19.60 |
|  |  |  | pbo |  | 176 | 55.00 | 53.00 | 92.50±19.50 |
| Wilding 2012^10^ | T2D | 48 | dapa | 2.5mg | 202 | 59.80 | 49.50 | 111.30±36.00 |
|  |  |  | dapa | 5mg | 211 | 59.30 | 47.40 | 109.70±36.70 |
|  |  |  | dapa | 10mg | 194 | 59.30 | 44.80 | 114.30±34.90 |
|  |  |  | pbo |  | 193 | 58.80 | 49.20 | 115.90±39.70 |
| Ferdinand 2019^11^ | T2D | 24 | empa | 10mg-25mg | 78 | 56.50 | 55.10 | 91.15±18.95 |
|  |  |  | pbo |  | 72 | 57.20 | 50.00 | 91.49±20.79 |
| Hadjadj 2016^12^ | T2D | 24 | empa | 10mg | 161 | 52.20 | 60.20 | 93.60±22.30 |
|  |  |  | empa | 25mg | 165 | 51.00 | 63.60 | 95.00±20.90 |
|  |  |  | pbo |  | 168 | 53.40 | 51.20 | 90.80±19.30 |
|  |  |  | empa | 10mg | 167 | 52.30 | 59.30 | 93.30±22.00 |
|  |  |  | empa | 25mg | 169 | 53.60 | 52.10 | 92.20±19.20 |
|  |  |  | pbo |  | 164 | 51.60 | 56.10 | 93.40±20.10 |
| Lewin 2015^13^ | T2D | 52 | empa | 25mg | 134 | 54.20 | 52.20 | 90.10±19.60 |
|  |  |  | pbo |  | 133 | 53.80 | 56.40 | 89.50±20.30 |
| Ji 2019^14^ | T2D | 26 | ertu | 5mg | 170 | 56.10 | 55.90 | 97.90±19.20 |
|  |  |  | ertu | 15mg | 169 | 56.30 | 58.00 | 100.20±19.80 |
|  |  |  | pbo |  | 167 | 56.90 | 52.70 | 99.90±20.20 |
| Pratley 2018^15^ | T2D | 26 | ertu | 5mg | 243 | 55.20 | 50.60 | 91.90±20.40 |
|  |  |  | ertu | 15mg | 244 | 55.10 | 51.60 | 92.60±19.20 |
|  |  |  | pbo |  | 247 | 54.80 | 62.30 | 92.60±18.20 |
| Rosenstock 2018^16^ | T2D | 26 | ertu | 15mg | 205 | 56.90 | 45.40 | 91.00±20.60 |
|  |  |  | pbo |  | 209 | 56.50 | 46.90 | 91.60±19.80 |
| Inoue 2019^17^ | T2D | 24 | ipra | 50mg | 24 | 60.50 | 54.20 | 91.00±26.40 |
|  |  |  | pbo |  | 24 | 60.80 | 58.30 | 82.90±24.80 |
| Lu 2016^18^ | T2D | 24 | ipra | 50mg | 87 | 53.90 | 50.60 | 144.12±59.42 |
|  |  |  | pbo |  | 83 | 53.40 | 39.80 | 154.48±69.97 |
| Danne 2018^19^ | T1D | 52 | sota | 200mg | 261 | 42.30 | 53.30 | 91.56±18.78 |
|  |  |  | sota | 400mg | 263 | 41.70 | 50.60 | 91.66±17.41 |
|  |  |  | pbo |  | 258 | 39.70 | 51.90 | 92.89±18.25 |
| Garg 2017^20^ | T1D | 24 | sota | 400mg | 699 | 43.30 | 51.20 | 91.51±19.76 |
|  |  |  | pbo |  | 703 | 42.40 | 48.20 | 92.47±21.89 |
| Zambrowicz 2012^21^ | T2D | 4 | sota | 150mg qd | 12 | 53.00 | 50.00 | 129.00±28.00 |
|  |  |  | sota | 300mg qd | 12 | 52.00 | 66.70 | 139.00±36.00 |
|  |  |  | pbo |  | 12 | 55.00 | 50.00 | 114.00±18.00 |
| Leiter 2014^22^ | T2D | 104 | cana | 300mg | 485 | 55.80 | 49.70 | 91.40±19.40 |
|  |  |  | glimepiride |  | 482 | 56.30 | 54.60 | 89.50±17.50 |
| Handelsman 2018^23^ | T2D | 26 | dapa+saxagliptin | 10mg+5mg | 232 | 55.90 | 43.10 | 92.20±20.20 |
|  |  |  | sitagliptin | 100mg | 229 | 55.80 | 48.00 | 92.90±22.50 |
| Jeon 2018^24^ | T2D | 24 | dapa | 10mg qd | 162 | 59.24 | 54.32 | 99.69±10.52 |
|  |  |  | Insulin glargine |  | 148 | 56.13 | 47.30 | 98.60±12.34 |
| Ferrannini 2013^25^ | T2D | 90 | empa | 10mg | 80 | 59.00 | 46.20 | 94.40±18.90 |
|  |  |  | empa | 25mg | 88 | 59.00 | 52.30 | 92.30±19.10 |
|  |  |  | metformin |  | 56 | 58.00 | 50.00 | 88.00±19.30 |
|  | T2D | 90 | empa | 10mg | 137 | 60.00 | 50.00 | 93.60±18.10 |
|  |  |  | empa | 25mg | 139 | 60.00 | 53.00 | 92.60±21.40 |
|  |  |  | sitagliptin |  | 56 | 60.00 | 51.80 | 91.40±17.50 |
| Miller 2018^26^ | T2D | 26 | ertu+sitagliptin | 5mg | 98 | 56.40 | 58.20 | 90.00±17.20 |
|  |  |  | pbo |  | 97 | 54.30 | 58.80 | 92.60±21.60 |
| baseline eGFR [60,90) ml/min/1.73m^2^ | | | |  |  |  |  |  |
| Bode 2015^27^ | T2D | 104 | cana | 100mg | 241 | 64.30 | 51.50 | 77.60±17.00 |
|  |  |  | cana | 300mg | 236 | 63.40 | 54.70 | 78.70±16.40 |
|  |  |  | pbo |  | 237 | 63.20 | 60.30 | 76.10±16.30 |
| Forst 2014^28^ | T2D | 26 | cana | 100mg | 113 | 56.70 | 68.10 | 84.60±17.50 |
|  |  |  | cana | 300mg | 114 | 57.00 | 55.30 | 87.40±19.50 |
|  |  |  | pbo |  | 115 | 58.30 | 66.10 | 87.20±18.80 |
| Inagaki 2013^29^ | T2D | 12 | cana | 50mg | 82 | 57.40 | 61.00 | 83.50±16.10 |
|  |  |  | cana | 100mg | 74 | 57.70 | 70.30 | 86.90±15.50 |
|  |  |  | cana | 200mg | 76 | 57.00 | 64.50 | 83.80±15.00 |
|  |  |  | cana | 300mg | 75 | 57.10 | 73.30 | 86.90±15.20 |
|  |  |  | pbo |  | 75 | 57.70 | 72.00 | 83.00±16.50 |
| Inagaki 2014^30^ | T2D | 24 | cana | 100mg | 90 | 58.40 | 65.60 | 81.40±13.80 |
|  |  |  | cana | 200mg | 88 | 57.40 | 81.80 | 87.20±18.60 |
|  |  |  | pbo |  | 93 | 58.20 | 64.50 | 84.70±13.70 |
| Inagaki 2016^31^ | T2D | 16 | cana | 10mg | 76 | 59.70 | 57.90 | 83.80±18.40 |
|  |  |  | pbo |  | 70 | 56.10 | 70.00 | 86.10±21.70 |
| Kadowaki 2017^32^ | T2D | 24 | cana | 100mg | 70 | 58.40 | 77.10 | 84.70±15.60 |
|  |  |  | pbo |  | 68 | 56.00 | 77.90 | 83.90±17.10 |
| Lavalle-González 2013^3^ | T2D | 26 | cana | 100mg | 368 | 55.50 | 47.30 | 89.70± / |
|  |  |  | pbo |  | 183 | 55.30 | 51.40 | 87.70± / |
| Rosenstock 2016^33^ | T2D | 26 | cana | 300mg | 237 | 55.40 | 48.50 | 87.00±19.00 |
|  |  |  | cana | 100mg | 237 | 54.20 | 45.60 | 89.00±19.00 |
|  |  |  | pbo |  | 237 | 55.20 | 48.90 | 87.00±19.00 |
| Stenlof 2013^34^ | T2D | 26 | cana | 300mg | 197 | 55.30 | 45.20 | 86.60±19.10 |
|  |  |  | cana | 100mg | 195 | 55.10 | 41.50 | 88.50±20.20 |
|  |  |  | pbo |  | 192 | 55.70 | 45.80 | 86.00±21.50 |
| Araki 2017^35^ | T2D | 52 | dapa | 10mg | 122 | 58.30 | 73.00 | 77.30±19.10 |
|  |  |  | pbo |  | 60 | 57.60 | 66.70 | 79.60±16.40 |
| Bailey 2015^36^ | T2D | 102 | dapa | 2.5mg | 65 | 53.00 | 55.40 | 85.59±20.13 |
|  |  |  | dapa | 5mg | 64 | 52.60 | 48.40 | 83.13±18.43 |
|  |  |  | dapa | 10mg | 70 | 50.60 | 48.60 | 85.37±18.95 |
|  |  |  | pbo |  | 75 | 52.70 | 41.30 | 86.16±17.32 |
| Bolinder 2012^37^ | T2D | 24 | dapa | 10mg | 89 | 60.60 | 55.10 | 86.00±14.20 |
|  |  |  | pbo |  | 91 | 60.80 | 56.00 | 82.60±16.10 |
| McMurray 2019^38^ | HF | 35 | dapa | 10mg | 2373 | 66.20 | 76.20 | 66.00±19.60 |
|  |  |  | pbo |  | 2371 | 66.50 | 77.00 | 65.50±19.30 |
| Schumm-Draeger 2015^39^ | T2D | 16 | dapa | 10mg qd | 99 | 58.50 | 49.50 | 82.50±13.82 |
|  |  |  | dapa | 2.5mg bid | 100 | 58.30 | 37.00 | 87.70±19.62 |
|  |  |  | dapa | 5mg bid | 99 | 55.30 | 46.50 | 86.50±18.10 |
|  |  |  | pbo |  | 101 | 58.50 | 46.50 | 84.40±18.16 |
| Strojek 2011^40^ | T2D | 24 | dapa | 2.5mg | 154 | 59.90 | 50.00 | 80.90±18.60 |
|  |  |  | dapa | 5mg | 142 | 60.20 | 50.00 | 83.50±19.60 |
|  |  |  | dapa | 10mg | 151 | 58.90 | 43.70 | 82.20±17.90 |
|  |  |  | pbo |  | 145 | 60.30 | 49.00 | 80.20±19.10 |
| Weber 2016^41^ | T2D | 12 | dapa | 10mg | 225 | 56.00 | 52.00 | 84.80±19.70 |
|  |  |  | pbo |  | 224 | 57.00 | 58.00 | 87.00±19.50 |
| Wilding 2009^42^ | T2D | 12 | dapa | 10mg | 24 | 55.70 | 54.20 | 87.20±33.00 |
|  |  |  | dapa | 20mg | 24 | 56.10 | 54.20 | 89.20±16.00 |
|  |  |  | pbo |  | 23 | 58.40 | 69.60 | 86.20±21.50 |
| Yang 2018^43^ | T2D | 24 | dapa | 10mg | 139 | 56.50 | 47.50 | 88.24±19.36 |
|  |  |  | pbo |  | 133 | 58.60 | 48.10 | 88.32±19.78 |
| Barnett 2014^44^ | T2D | 52 | empa | 25mg | 97 | 62.00 | 62.90 | 72.30±11.20 |
|  |  |  | empa | 10mg | 98 | 63.20 | 61.20 | 70.80±10.30 |
|  |  |  | pbo |  | 95 | 62.60 | 58.90 | 71.80±10.20 |
| Haring 2013^45^ | T2D | 24 | empa | 10mg | 225 | 57.00 | 50.00 | 86.50±21.80 |
|  |  |  | empa | 25mg | 216 | 57.40 | 53.00 | 88.30±22.60 |
|  |  |  | pbo |  | 225 | 56.90 | 50.00 | 86.90±20.10 |
| Haring 2014^46^ | T2D | 24 | empa | 10mg | 217 | 55.50 | 58.00 | 89.50±19.60 |
|  |  |  | empa | 25mg | 213 | 55.60 | 56.00 | 87.70±19.30 |
|  |  |  | pbo |  | 207 | 56.00 | 56.00 | 89.70±21.40 |
| Hattori 2018^47^ | T2D | 52 | empa | 10mg | 51 | 57.40 | 74.50 | 74.30±16.30 |
|  |  |  | pbo |  | 51 | 58.10 | 80.40 | 71.90±18.60 |
| Kario 2018^48^ | T2D | 12 | empa | 10mg | 68 | 70.90 | 52.90 | 68.40±15.10 |
|  |  |  | pbo |  | 63 | 69.30 | 52.40 | 70.10±16.20 |
| Kadowaki 2014^49^ | T2D | 12 | empa | 5mg | 110 | 57.30 | 76.40 | 86.49±17.48 |
|  |  |  | empa | 10mg | 109 | 57.90 | 70.60 | 85.76±14.63 |
|  |  |  | empa | 25mg | 109 | 57.20 | 77.10 | 85.23±15.84 |
|  |  |  | empa | 50mg | 110 | 56.60 | 77.30 | 86.52±15.86 |
|  |  |  | pbo |  | 109 | 58.70 | 73.40 | 84.61±14.91 |
| Kawamori 2018^50^ | T2D | 24 | empa | 10mg | 182 | 60.00 | 78.00 | 89.30±18.30 |
|  |  |  | pbo |  | 93 | 59.80 | 77.40 | 86.30±15.20 |
| Kovacs 2014^51^ | T2D | 24 | empa | 10mg | 165 | 54.70 | 50.30 | 84.30±20.90 |
|  |  |  | empa | 25mg | 168 | 54.20 | 50.60 | 87.40±24.40 |
|  |  |  | pbo |  | 165 | 54.60 | 44.20 | 85.50±20.10 |
| Lewin 2015^13^ | T2D | 52 | empa | 10mg | 135 | 55.20 | 54.10 | 87.80±17.70 |
|  |  |  | pbo |  | 133 | 53.80 | 56.40 | 89.50±20.30 |
| Packer 2020^52^ | HF | 52 | empa | 10mg qd | 1863 | 67.20 | 76.50 | 61.80±21.70 |
|  |  |  | pbo |  | 1867 | 66.50 | 75.60 | 62.20±21.50 |
| Roden 2013^53^ | T2D | 24 | empa | 10mg | 224 | 56.20 | 63.00 | 87.70±19.16 |
|  |  |  | empa | 25mg | 224 | 53.80 | 65.00 | 87.54±18.29 |
|  |  |  | pbo |  | 228 | 54.90 | 54.00 | 86.83±17.94 |
| Rosenstock 2014^54^ | T2D | 52 | empa | 10mg | 186 | 56.70 | 52.00 | 84.10±17.80 |
|  |  |  | empa | 25mg | 189 | 58.00 | 44.00 | 84.40±16.60 |
|  |  |  | pbo |  | 188 | 55.30 | 40.00 | 83.40±15.40 |
| Rosenstock 2015^55^ | T2D | 78 | empa | 10mg | 169 | 58.60 | 55.00 | 85.10±22.80 |
|  |  |  | empa | 25mg | 155 | 59.90 | 60.00 | 83.30±25.10 |
|  |  |  | pbo |  | 170 | 58.10 | 53.00 | 85.00±23.00 |
| Ross 2015^56^ | T2D | 16 | empa | 12.5mg bid | 215 | 57.60 | 57.20 | 88.60±20.00 |
|  |  |  | empa | 25mg qd | 214 | 58.20 | 53.30 | 88.90±19.50 |
|  |  |  | empa | 5mg bid | 215 | 58.80 | 55.80 | 89.90±22.30 |
|  |  |  | empa | 10mg qd | 214 | 58.50 | 50.50 | 89.20±20.60 |
|  |  |  | pbo |  | 107 | 57.90 | 51.40 | 89.50±18.50 |
| Dagogo-Jack 2018^57^ | T2D | 52 | ertu | 5mg | 156 | 59.20 | 51.90 | 87.00±17.50 |
|  |  |  | ertu | 15mg | 153 | 59.70 | 53.60 | 86.90±15.60 |
|  |  |  | pbo |  | 153 | 58.30 | 65.40 | 89.90±17.50 |
| Rosenstock 2018^16^ | T2D | 26 | ertu | 5mg | 207 | 56.60 | 46.90 | 88.90±17.50 |
|  |  |  | pbo |  | 209 | 56.50 | 46.90 | 91.60±19.80 |
| Terra 2017^58^ | T2D | 26 | ertu | 5mg | 156 | 56.80 | 57.10 | 88.50±18.40 |
|  |  |  | ertu | 15mg | 152 | 56.20 | 59.20 | 88.30±18.00 |
|  |  |  | pbo |  | 153 | 56.10 | 53.60 | 86.20±19.40 |
| Bando 2016^59^ | T2D | 12 | ipra | 50mg | 40 | 54.80 | 65.00 | 79.50±14.90 |
|  |  |  | pbo |  | 22 | 55.40 | 63.60 | 76.50±13.40 |
| Han 2018^60^ | T2D | 24 | ipra | 50mg | 73 | 57.62 | 50.70 | 89.38±13.61 |
|  |  |  | pbo |  | 66 | 57.44 | 48.50 | 90.66±17.47 |
| Ishihara 2016^61^ | T2D | 16 | ipra | 50mg | 168 | 58.70 | 62.50 | 83.98±20.27 |
|  |  |  | pbo |  | 87 | 59.20 | 58.60 | 80.11±21.94 |
| Kashiwagi 2015^62^ | T2D | 24 | ipra | 50mg | 118 | 63.90 | 78.00 | 60.30±13.07 |
|  |  |  | pbo |  | 46 | 65.70 | 78.30 | 62.70±13.13 |
| Seino 2018^63^ | T2D | 16 | luseo | 2.5mg | 159 | 57.40 | 70.40 | 86.50±19.30 |
|  |  |  | pbo |  | 74 | 57.10 | 68.90 | 87.90±20.20 |
| Buse 2018^64^ | T1D | 52 | sota | 200mg | 263 | 46.60 | 47.90 | 87.04±20.18 |
|  |  |  | sota | 400mg | 262 | 46.40 | 45.80 | 86.58±18.93 |
|  |  |  | pbo |  | 268 | 45.20 | 51.10 | 87.58±18.47 |
| Kaku 2014^65^ | T2D | 24 | tofo | 10mg | 57 | 58.60 | 66.70 | 84.68±20.04 |
|  |  |  | tofo | 20mg | 58 | 56.60 | 67.20 | 86.78±19.62 |
|  |  |  | tofo | 40mg | 58 | 57.00 | 67.20 | 86.00±18.18 |
|  |  |  | pbo |  | 56 | 56.80 | 66.10 | 83.78±17.68 |
| Terauchi 2017^66^ | T2D | 16 | tofo | 20mg | 141 | 59.10 | 63.80 | 79.70±19.80 |
|  |  |  | pbo |  | 70 | 56.40 | 68.60 | 79.50±17.00 |
| Leiter 2014-2^22^ | T2D | 104 | cana | 100ng | 483 | 56.40 | 52.20 | 89.70±19.30 |
|  |  |  | glimepiride |  | 482 | 56.30 | 54.60 | 89.50±17.50 |
| Miller 2018-2^26^ | T2D | 26 | ertu+sitagliptin | 15mg | 96 | 56.10 | 55.20 | 89.50±18.10 |
|  |  |  | pbo |  | 97 | 54.30 | 58.80 | 92.60±21.60 |
| Schernthaner 2013^67^ | T2D | 52 | cana | 300mg | 377 | 56.60 | 54.90 | 88.30± / |
|  |  |  | sitagliptin | 100mg | 378 | 56.70 | 56.90 | 89.50± / |
| Nauck 2011^68^ | T2D | 52 | dapa | 2.5→5→10mg | 400 | 58.00 | 55.30 | 89.60±21.40 |
|  |  |  | glipizide |  | 401 | 59.00 | 54.90 | 90.50±22.60 |
| Müller-Wieland 2018^69^ | T2D | 52 | dapa | 10mg | 314 | 57.40 | 64.30 | 87.20±19.40 |
|  |  |  | dapa+saxagliptin | 10mg+5mg | 312 | 59.20 | 60.90 | 88.00±19.60 |
|  |  |  | glimepiride |  | 313 | 58.60 | 66.50 | 86.00±17.50 |
| Cho 2018^70^ | T2D | 24 | dapa | 5mg | 36 | 63.10 | 63.90 | 72.40±19.60 |
|  |  |  | pioglitazone |  | 35 | 63.60 | 54.30 | 67.10±16.80 |
| Scott 2018^71^ | T2D | 24 | dapa | 10mg | 306 | 66.60 | 60.80 | 76.90±12.30 |
|  |  |  | sitagliptin |  | 307 | 67.70 | 55.00 | 79.40±11.30 |
| Shimizu 2018^72^ | T2D | 24 | dapa | 5mg | 33 | 56.20 | 57.60 | 79.40±15.80 |
|  |  |  | control |  | 24 | 57.10 | 62.50 | 76.90±19.00 |
| Hayashi 2017^73^ | T2D | 12 | dapa | 5mg | 40 | 53.80 | 80.00 | 86.20±18.40 |
|  |  |  | sitagliptin | 50mg | 40 | 54.20 | 75.00 | 83.50±22.70 |
| Zinman 2015^74^ | T2D | 135 | empa | 10mg | 2345 | 63.00 | 70.50 | 74.40±21.80 |
|  |  |  | empa | 25mg | 2342 | 63.20 | 71.90 | 74.30±21.10 |
|  |  |  | pbo |  | 2333 | 63.20 | 72.00 | 74.00±21.10 |
| Araki 2015^75^ | T2D | 52 | empa | 10mg | 136 | 61.30 | 73.00 | 87.10±20.30 |
|  |  |  | empa | 25mg | 137 | 61.80 | 70.00 | 85.60±19.30 |
|  |  |  | metformin |  | 63 | 60.00 | 75.00 | 86.90±14.80 |
| Hollander 2019^76^ | T2D | 104 | ertu | 5mg | 445 | 58.70 | 51.00 | 88.30±18.70 |
|  |  |  | ertu | 15mg | 435 | 58.00 | 43.90 | 86.60±18.30 |
|  |  |  | glimepiride |  | 435 | 57.90 | 51.50 | 86.50±18.50 |
| Tsurutani 2018^77^ | T2D | 12 | ipra | 50mg | 60 | 53.50 | 68.30 | 77.20± / |
|  |  |  | sitagliptin |  | 59 | 54.00 | 67.80 | 80.40± / |
| baseline eGFR [45,60) ml/min/1.73m^2^ | | | |  |  |  |  |  |
| Takashima 2018^78^ | T2D | 52 | cana | 100mg | 21 | 64.70 | 55.00 | 57.10±16.20 |
|  |  |  | pbo |  | 21 | 65.40 | 60.00 | 55.40±12.30 |
| Fioretto 2018^79^ | T2D | 24 | dapa | 10mg | 160 | 65.30 | 56.90 | 53.48±8.76 |
|  |  |  | pbo |  | 161 | 66.20 | 56.50 | 53.68±10.67 |
| Pollock 2019^80^ | T2D | 24 | dapa | 10mg | 145 | 64.70 | 70.00 | 50.20±13.00 |
|  |  |  | pbo |  | 148 | 64.70 | 71.00 | 47.70±13.50 |
| Barnett 2014^44^ | T2D | 52 | empa | 25mg | 187 | 64.60 | 57.20 | 45.40±10.20 |
|  |  |  | pbo |  | 187 | 65.10 | 56.70 | 44.30±10.30 |
| Grunberger 2017^81^ | T2D | 26 | ertu | 5mg | 158 | 66.70 | 53.20 | 46.80±7.80 |
|  |  |  | ertu | 15mg | 155 | 67.50 | 48.40 | 46.90±9.10 |
|  |  |  | pbo |  | 154 | 67.50 | 46.80 | 46.00±9.40 |
| Haneda 2016^82^ | T2D | 24 | luseo | 2.5mg | 95 | 67.90 | 75.80 | 52.00±9.40 |
|  |  |  | pbo |  | 50 | 68.40 | 78.00 | 52.40±8.20 |
| baseline eGFR ＜45ml/min/1.73m^2^ | | | |  |  |  |  |  |
| Yale 2013^83^ | T2D | 26 | cana | 100mg | 90 | 69.50 | 64.40 | 39.70±6.90 |
|  |  |  | cana | 300mg | 89 | 67.90 | 53.90 | 38.50±6.90 |
|  |  |  | pbo |  | 90 | 68.20 | 63.30 | 40.10±6.80 |
| Heerspink 2020^84^ | CKD | 52 | dapa | 10mg qd | 2149 | 61.80 | 67.10 | 43.20±12.30 |
|  |  |  | pbo |  | 2149 | 61.90 | 66.70 | 43.00±12.40 |
| Bhatt 2021^85^ | T2D | 64 | sota | 200→400mg | 5292 | 69.00 | 55.70 | 44.40± / |
|  |  |  | pbo |  | 5292 | 69.00 | 54.50 | 44.70± / |
| Kohan 2013^86^ | T2D | 104 | dapa | 5mg qd | 83 | 66.00 | 66.30 | 44.20±8.80 |
|  |  |  | dapa | 10mg qd | 85 | 68.00 | 65.90 | 43.90±10.60 |
|  |  |  | pbo |  | 84 | 67.00 | 63.10 | 45.60±10.00 |
| Barnett 2014^44^ | T2D | 52 | empa | 25mg | 37 | 65.40 | 56.80 | 24.40±5.20 |
|  |  |  | pbo |  | 37 | 62.90 | 51.40 | 22.00±4.40 |

Table S2. Evaluation for risk of bias in included RCTs

| Author, year | Adequate randomization sequence generation | Adequate  allocation concealment | Blinding  of participants and caregivers | Blinding of  outcome assessors  and adjudicators | Free of infrequent  missing outcome data | Free of selective outcome reporting | Free of other bias |
| --- | --- | --- | --- | --- | --- | --- | --- |
| Cusi,2018^1^ | **Definitely yes**  Using a computer-generated randomization  schedule | **Definitely yes**  Randomized, double-blind | **Definitely yes**  Double-blind  (participant,  investigator) | **Definitely yes** | **Probably yes**  There were 7.7% (2/26) and 10% (3/30) patients in SGLT2i and placebo groups with missing outcome data, respectively; missing outcome data were generally balanced across treatment groups, with similar reasons for missing data across groups | **Definitely yes** | **Probably yes**  Generally balanced baseline  characteristics across groups |
| Ji, 2015^2^ | **Definitely yes**  Using a computer-generated randomization  schedule | **Definitely yes**  Randomized, double-blind | **Definitely yes**  Double-blind  (participant,  investigator) | **Definitely yes** | **Definitely yes**  There were 4.7% (21/450) and 4.0% (9/226) patients in SGLT2i and placebo groups with missing outcome data, respectively; missing outcome data were generally balanced across treatment groups, with similar reasons for missing data across groups | **Definitely yes** | **Probably yes**  Generally balanced baseline  characteristics across groups |
| Lavalle-González, 2013^3^ | **Definitely yes**  Using a computer-generated randomization  schedule | **Definitely yes**  Randomized, double-blind | **Definitely yes**  Double-blind  (participant,  investigator) | **Definitely yes** | **Probably no**  There were 12.2% (90/735) and 15.3% (28/183) patients in SGLT2i and control groups with missing outcome data, respectively; missing outcome data were generally balanced across treatment groups, with similar reasons for missing data across groups | **Definitely yes** | **Probably yes**  Generally balanced baseline  characteristics across groups |
|  |  |  |  |  |  |  |  |
| Wilding,2013^4^ | **Definitely yes**  Using a computer-generated randomization  schedule | **Definitely yes**  Randomized, double-blind | **Definitely yes**  Double-blind  (participant,  investigator) | **Definitely yes** | **Definitely no**  There were 29.7% (93/313) and 42.3% (66/156) patients in SGLT2i and placebo groups with missing outcome data, respectively; missing outcome data were generally balanced across treatment groups, with similar reasons for missing data across groups | **Definitely yes** | **Probably yes**  Generally balanced baseline  characteristics across groups |
| Henry,2018^5^ | **Definitely yes**  Using an interactive voice response system/interactive web response system | **Definitely yes**  Randomized, double-blind | **Definitely yes**  Double-blind  (participant,  investigator) | **Definitely yes** | **Definitely yes**  There were 4% (2/50)and 2% (1/50)patients in SGLT2i and placebo groups with missing outcome data, respectively; missing outcome data were generally balanced across treatment groups, with similar reasons for missing data across groups | **Definitely yes** | **Probably yes**  Generally balanced baseline  characteristics across groups |
| Jabbour, 2018^6^ | **Definitely yes**  Using an interactive voice response system and web-response system | **Definitely yes**  Randomized, double-blind | **Definitely yes**  Double-blind  (participant,  investigator) | **Definitely yes** | **Definitely yes**  There were 1.3% (3/228) and 1.3% (3/227) patients in SGLT2i and placebo groups with missing outcome data, respectively; missing outcome data were generally balanced across treatment groups, with similar reasons for missing data across groups | **Definitely yes** | **Probably yes**  Generally balanced baseline  characteristics across groups |
| Ji, 2014^7^ | **Definitely yes**  Using an interactive voice response system | **Definitely yes**  Randomized, double-blind | **Definitely yes**  Double-blind  (participant,  investigator) | **Definitely yes** | **Probably no**  There were 11.8% (31/261) and 14.4% (19/132) patients in SGLT2i and placebo groups with missing outcome data, respectively; missing outcome data were generally balanced across treatment groups, with similar reasons for missing data across groups | **Definitely yes** | **Probably yes**  Generally balanced baseline  characteristics across groups |
| Mathieu, 2015^8^ | **Definitely yes**  Using an interactive voice response system | **Definitely yes**  Randomized, double-blind | **Definitely yes**  Double-blind  (participant,  investigator) | **Definitely yes** | **Probably yes**  There were 7.5% (12/160) and 4.4% (7/160) patients in SGLT2i and placebo groups with missing outcome data, respectively; missing outcome data were generally balanced across treatment groups, with similar reasons for missing data across groups | **Definitely yes** | **Probably yes**  Generally balanced baseline  characteristics across groups |
| Rosenstock,2015^9^ | **Definitely yes**  Using a centralized blocked randomization schedule | **Definitely yes**  Randomized,  double-blind | **Definitely yes**  Double-blind  (participant,  investigator) | **Definitely yes** | **Probably yes**  There were 5.6% (10/179) and 8.5% (15/176) patients in SGLT2i and placebo groups with missing outcome data, respectively; missing outcome data were generally balanced across treatment groups, with similar reasons for missing data across groups | **Definitely yes** | **Probably yes**  Generally balanced baseline  characteristics across groups |
| Wilding, 2012^10^ | **Definitely yes**  Using a computer-generated randomization  schedule | **Definitely yes**  Randomized, double-blind | **Definitely yes**  Double-blind  (participant,  investigator) | **Definitely yes** | **Probably no**  There were 14.5% (88/607) and 18.6% (36/193) patients in SGLT2i and placebo groups with missing outcome data, respectively; missing outcome data were generally balanced across treatment groups, with similar reasons for missing data across groups | **Definitely yes** | **Probably yes**  Generally balanced baseline  characteristics across groups |
| Fedinand,2019^11^ | **Definitely yes**  Using a computer-generated random sequence and a third-party interactive telephone/web-based system | **Definitely yes**  Randomized, double-blind | **Definitely yes**  Double-blind  (participant,  investigator) | **Definitely yes** | **Probably yes**  There were 4.9% (4/82) and 8.9% (7/79) patients in SGLT2i and control groups with missing outcome data, respectively; missing outcome data were generally balanced across treatment groups, with similar reasons for missing data across groups | **Definitely yes** | **Probably yes**  Generally balanced baseline  characteristics across groups |
| Hadjadj, 2016^12^ | **Definitely yes**  Using a  computer-generated random sequence  and an interactive voice and Web response  system. | **Definitely yes**  Randomized, double-blind | **Definitely yes**  Double-blind  (participant,  investigator) | **Definitely yes** | **Probably yes**  There were 7.8% (53/680) and 11.7% (40/341) patients in SGLT2i and control groups with missing outcome data, respectively; missing outcome data were generally balanced across treatment groups, with similar reasons for missing data across groups | **Definitely yes** | **Probably yes**  Generally balanced baseline  characteristics across groups |
| Lewin, 2015^13^ | **Definitely yes**  Using a  third-party interactive voice and web response system | **Definitely yes**  Randomized,  double-blind | **Definitely yes** Double-blind  (details not reported) | **Definitely yes** | **Probably no**  There were 15.8% (43/273) and 14.1% (19/135) patients in SGLT2i and control groups with missing outcome data, respectively; missing outcome data were generally balanced across treatment groups, with similar reasons for missing data across groups | **Definitely yes** | **Probably yes**  Generally balanced baseline  characteristics across groups |
| Ji,2019^14^ | **Definitely yes**  Using a computer-generated randomization code based  on the method of random permuted blocks | **Definitely yes**  Randomized, double-blind | **Definitely yes**  Double-blind  (participant,  investigator) | **Definitely yes** | **Probably yes**  There were 7.1% (24/339) and 10.2% (17/167) patients in active agent and placebo groups with missing outcome data, respectively; missing outcome data were generally balanced across treatment groups, with similar reasons for missing data across groups | **Definitely yes** | **Probably yes**  Generally balanced baseline  characteristics across groups |
| Pratley,2018^15^ | **Definitely yes**  Using a an interactive voice response system/integrated web response system | **Definitely yes**  Randomized, double-blind | **Definitely yes**  Double-blind  (participant,  investigator) | **Definitely yes** | **Probably yes**  There were 8.2% (40/488) and 10.5% (26/247) patients in active agent and placebo groups with missing outcome data, respectively; missing outcome data were generally balanced across treatment groups, with similar reasons for missing data across groups | **Definitely yes** | **Probably yes**  Generally balanced baseline  characteristics across groups |
| Rosenstock, 2018^16^ | **Definitely yes**  Using a computer-generated randomization code based  on the method of random permuted blocks | **Definitely yes**  Randomized, double-blind | **Definitely yes**  Double-blind  (participant,  investigator) | **Definitely yes** | **Probably yes**  There were 5.1% (21/412) and 9.1% (19/209) patients in active agent and placebo groups with missing outcome data, respectively; missing outcome data were generally balanced across treatment groups, with similar reasons for missing data across groups | **Definitely yes** | **Probably yes**  Generally balanced baseline  characteristics across groups |
| Inoue,2019^17^ | **Definitely yes**  Using a computer-generated random sequence | **Definitely yes**  Open-labeled | **Definitely no**  Open-labeled  (participant,  investigator) | **Definitely no** | **Probably yes**  There were 8.3% (2/24) patients in SGLT-2 group with missing outcome data. | **Definitely yes** | **Probably yes**  Generally balanced baseline  characteristics across groups |
| Lu, 2016^18^ | **Definitely yes**  Using an interactive web randomization system with treatment codes | **Definitely yes**  Randomized,  double-blind | **Definitely yes** Double-blind  (details not reported) | **Definitely yes** | **Probably no**  There were 14.9% (13/87) and 16.9% (14/83) patients in SGLT2i and placebo groups with missing outcome data, respectively; missing outcome data were generally balanced across treatment groups, with similar reasons for missing data across groups | **Definitely yes** | **Probably yes**  Generally balanced baseline  characteristics across groups |
| Danne, 2018^19^ | **Probably yes**  Randomized,  double-blind | **Probably yes**  Randomized,  double-blind | **Definitely yes** Double-blind  (details not reported) | **Definitely yes** | **Probably no**  There were 13.5% (71/524) and 12.8% (33/258) patients in SGLT2i and control groups with missing outcome data, respectively; missing outcome data were generally balanced across treatment groups, with similar reasons for missing data across groups | **Definitely yes** | **Probably yes**  Generally balanced baseline  characteristics across groups |
| Grag, 2017^20^ | **Probably yes**  Randomized,  double-blind | **Probably yes**  Randomized,  double-blind | **Definitely yes** Double-blind  (details not reported) | **Definitely yes** | **Probably no**  There were 13.6% (95/699) and 11.5% (81/703) patients in SGLT2i and control groups with missing outcome data respectively; missing outcome data were generally balanced across treatment groups, with similar reasons for missing data across groups | **Definitely yes** | **Probably yes**  Generally balanced baseline  characteristics across groups |
| Zambrowicz,2012^21^ | **Definitely yes**  Using a SAS-generated randomization schedule | **Definitely yes**  Randomized, double-blind | **Definitely yes**  Double-blind  (participant,  investigator) | **Definitely yes** | **Probably yes**  There was 8.3% (1/12) patient in placebo group with missing outcome data. | **Definitely yes** | **Probably yes**  Generally balanced baseline  characteristics across groups |
| Leiter 2014^22^ | **Definitely yes**  Using an interactive voice or web response system | **Definitely yes**  Randomized, double-blind | **Definitely yes**  Double-blind  (participant,  investigator) | **Definitely yes** | **Definitely no**  There were 31.2% (302/968) and 34.9% (168/482) patients in SGLT2i and control groups with missing outcome data, respectively; missing outcome data were generally balanced across treatment groups, with similar reasons for missing data across groups | **Definitely yes** | **Probably yes**  Generally balanced baseline  characteristics across groups |
| Handelsman 2018^23^ | **Definitely yes**  Using an interactive voice response system | **Definitely yes**  Randomized, double-blind | **Definitely yes**  Double-blind  (participant,  investigator) | **Definitely yes** | **Probably yes**  There were 89.2% (411/468) patients in SGLT2i and control groups with missing outcome data, totally; missing outcome data were generally balanced across treatment groups, with similar reasons for missing data across groups | **Definitely yes** | **Probably yes**  Generally balanced baseline  characteristics across groups |
| Jeon 2018^24^ | **Probably yes** | **Probably yes**  Open-labeled | **Definitely no**  Open-labeled | **Definitely no** | **Probably yes** | **Definitely yes** | **Probably yes**  Generally balanced baseline  characteristics across groups |
| Ferrannini 2013^25^ | **Probably yes** | **Probably yes**  Open-labeled | **Definitely no**  Open-labeled | **Definitely no** | **Probably yes**  There were 8.03% (53/660) patients in SGLT2i and control groups with missing outcome data, totally; missing outcome data were generally balanced across treatment groups, with similar reasons for missing data across groups | **Definitely yes** | **Probably yes**  Generally balanced baseline  characteristics across groups |
| Miller 2018^26^ | **Definitely yes**  Using a central electronic randomization system (interactive voice response system/integrated web response system) | **Definitely yes**  Randomized, double-blind | **Definitely yes**  Double-blind  (participant,  investigator) | **Definitely yes** | **Probably no**  There were 8.2% (16/194) and 21.6% (21/97) patients in SGLT2i and control groups with missing outcome data, respectively; missing outcome data were generally balanced across treatment groups, with similar reasons for missing data across groups | **Definitely yes** | **Probably yes**  Generally balanced baseline  characteristics across groups |
| Bode, 2015^27^ | **Probably yes**  Randomized,  double-blind | **Probably yes**  Randomized, double-blind | **Definitely yes**  Double-blind  (participant,  investigator) | **Definitely yes** | **Definitely no**  There were 24.1% (79/477) and 33.3% (79/237) patients in SGLT2i and control groups with missing outcome data, respectively; missing outcome data were generally balanced across treatment groups, with similar reasons for missing data across groups | **Definitely yes** | **Probably yes**  Generally balanced baseline  characteristics across groups |
| Forst, 2014^28^ | **Definitely yes**  Using an interactive voice response system/interactive web response system | **Definitely yes**  Randomized, double-blind | **Definitely yes**  Double-blind  (participant,  investigator) | **Definitely yes** | **Probably no**  There were 9.7% (22/227) and 20.9% (24/115) patients in SGLT2i and placebo groups with missing outcome data, respectively; missing outcome data were generally balanced across treatment groups, with similar reasons for missing data across groups | **Definitely yes** | **Probably yes**  Generally balanced baseline  characteristics across groups |
| Inagaki, 2013^29^ | **Definitely yes**  Using a block allocation method | **Definitely yes**  Randomized, double-blind | **Definitely yes**  Double-blind  (participant,  investigator) | **Definitely yes** | **Probably no**  There were 3.9% (12/307) and 13.3% (10/75) patients in SGLT2i and placebo groups with missing outcome data, respectively; missing outcome data were generally balanced across treatment groups, with similar reasons for missing data across groups | **Definitely yes** | **Probably yes**  Generally balanced baseline  characteristics across groups |
| Inagaki, 2014^30^ | **Definitely yes**  Using a block allocation method | **Definitely yes**  Randomized, double-blind | **Definitely yes**  Double-blind  (participant,  investigator) | **Definitely yes** | **Probably no**  There were 6.7% (12/179) and 20.4% (19/93) patients in SGLT2i and placebo groups with missing outcome data, respectively; missing outcome data were generally balanced across treatment groups, with similar reasons for missing data across groups | **Definitely yes** | **Probably yes**  Generally balanced baseline  characteristics across groups |
| Inagaki, 2016^31^ | **Definitely yes**  Using a block allocation method | **Definitely yes**  Randomized, double-blind | **Definitely yes**  Double-blind  (participant,  investigator) | **Definitely yes** | **Definitely yes** There were 0% (0/76) and 0% (0/70) patients in SGLT2i and placebo groups with missing outcome data, respectively; missing outcome data were generally balanced across treatment groups, with similar reasons for missing data across groups | **Definitely yes** | **Probably yes**  Generally balanced baseline  characteristics across groups |
| Kadowaki, 2017^32^ | **Probably yes**  Randomized,  double-blind | **Probably yes**  Randomized, double-blind | **Definitely yes**  Double-blind  (participant,  investigator) | **Definitely yes** | **Probably yes**  There were 4.3% (3/70) and 10.3% (7/68) patients in SGLT2i and placebo groups with missing outcome data, respectively; missing outcome data were generally balanced across treatment groups, with similar reasons for missing data across groups | **Definitely yes** | **Probably yes**  Generally balanced baseline  characteristics across groups |
| Rosenstock, 2016^33^ | **Definitely yes**  Using a computer-generated randomization  schedule | **Definitely yes**  Randomized, double-blind | **Definitely yes**  Double-blind  (participant,  investigator) | **Definitely yes** | **Probably no**  There were 7.8% (37/474) and 13.5% (32/237) patients in SGLT2i and control groups with missing outcome data, respectively; missing outcome data were generally balanced across treatment groups, with similar reasons for missing data across groups | **Definitely yes** | **Probably yes**  Generally balanced baseline  characteristics across groups |
| Stenlöf, 2013^34^ | **Probably yes**  Randomized,  double-blind | **Probably yes**  Randomized, double-blind | **Definitely yes**  Double-blind  (participant,  investigator) | **Definitely yes** | **Probably no**  There were 11.5% (45/392) and 16.7% (32/192) patients in SGLT2i and placebo groups with missing outcome data, respectively; missing outcome data were generally balanced across treatment groups, with similar reasons for missing data across groups | **Definitely yes** | **Probably yes**  Generally balanced baseline  characteristics across groups |
| Araki, 2017^35^ | **Definitely yes**  Using an interactive web response system | **Definitely yes**  Randomized, double-blind | **Definitely yes**  Double-blind  (participant,  investigator) | **Definitely yes** | **Probably yes**  There were 9.0% (11/122) and 10% (6/60) patients in SGLT2i and placebo groups with missing outcome data, respectively; missing outcome data were generally balanced across treatment groups, with similar reasons for missing data across groups | **Definitely yes** | **Probably yes**  Generally balanced baseline  characteristics across groups |
| Bailey, 2015^36^ | **Definitely yes**  Using a computer-generated randomization by an interactive voice response system and were stratified by site in blocks of seven. | **Definitely yes**  Randomized, double-blind | **Definitely yes**  Double-blind  (participant,  investigator) | **Definitely yes** | **Definitely no**  There were 37.2% (74/199) and 44% (33/75) patients in SGLT2i and placebo groups with missing outcome data, respectively; missing outcome data were generally balanced across treatment groups, with similar reasons for missing data across groups | **Definitely yes** | **Probably yes**  Generally balanced baseline  characteristics across groups |
| Bolinder, 2012^37^ | **Definitely yes**  Using a computer-generated randomization  schedule | **Definitely yes**  Randomized, double-blind | **Definitely yes**  Double-blind  (participant,  investigator) | **Definitely yes** | **Probably yes**  There were 8.8% (8/91) and 5.5% (5/91) patients in SGLT2i and placebo groups with missing outcome data, respectively; missing outcome data were generally balanced across treatment groups, with similar reasons for missing data across groups | **Definitely yes** | **Probably yes**  Generally balanced baseline  characteristics across groups |
| McMurray, 2019^38^ | **Definitely yes**  Using an interactive voice or web response system | **Definitely yes**  Randomized | **Probably yes**  Randomized | **Probably yes** | **Probably yes**  There were 10.5% (249/2373) and 10.9% (258/2371) patients in SGLT2i and control groups with missing outcome data, respectively; missing outcome data were generally balanced across treatment groups, with similar reasons for missing data across groups | **Definitely yes** | **Probably yes**  Generally balanced baseline  characteristics across groups |
| Schumm-Draeger, 2015^39^ | **Definitely yes**  Using an interactive web response system | **Definitely yes**  Randomized,  double-blind | **Definitely yes**  Double-blind  (participant,  investigator) | **Definitely yes** | **Probably yes**  There were 7.4% (22/299) and 7.9% (8/101) patients in SGLT2i and control groups with missing outcome data, respectively; missing outcome data were generally balanced across treatment groups, with similar reasons for missing data across groups | **Definitely yes** | **Probably yes**  Generally balanced baseline  characteristics across groups |
| Strojek, 2011^40^ | **Definitely yes**  Using a computer-generated randomization  schedule | **Definitely yes**  Randomized, double-blind | **Definitely yes**  Double-blind  (participant,  investigator) | **Definitely yes** | **Probably yes**  There were 8.2% (37/450)and 8.9% (13/146)patients in SGLT2i and placebo groups with missing outcome data, respectively; missing outcome data were generally balanced across treatment groups, with similar reasons for missing data across groups | **Definitely yes** | **Probably yes**  Generally balanced baseline  characteristics across groups |
| Weber,2016^41^ | **Definitely yes**  Using a block allocation method | **Definitely yes**  Randomized, double-blind | **Definitely yes**  Double-blind  (participant,  investigator) | **Definitely yes** | **Probably yes**  There were 6.2% (14/225)and 9.8% (22/224)patients in SGLT2i and placebo groups with missing outcome data, respectively; missing outcome data were generally balanced across treatment groups, with similar reasons for missing data across groups | **Definitely yes** | **Probably yes**  Generally balanced baseline  characteristics across groups |
| Wilding, 2009^42^ | **Probably yes**  Randomized,  double-blind | **Probably yes**  Randomized, double-blind | **Definitely yes**  Double-blind  (participant,  investigator) | **Definitely yes** | **Definitely no**  There were 8.3% (4/48) and 30.4% (7/23) patients in SGLT2i and placebo groups with missing outcome data, respectively; missing outcome data were generally balanced across treatment groups, with similar reasons for missing data across groups | **Definitely yes** | **Probably yes**  Generally balanced baseline  characteristics across groups |
| Yang, 2018^43^ | **Definitely yes**  Using an interactive voice response system | **Definitely yes**  Randomized, double-blind | **Definitely yes**  Double-blind  (participant,  investigator) | **Definitely yes** | **Probably yes**  There were 3.6% (5/139) and 6.8% (9/133) patients in SGLT2i and placebo groups with missing outcome data, respectively; missing outcome data were generally balanced across treatment groups, with similar reasons for missing data across groups | **Definitely yes** | **Probably yes**  Generally balanced baseline  characteristics across groups |
| Barnett, 2014^44^ | **Definitely yes**  Using a computer-generated random sequence | **Definitely yes**  Randomized, double-blind | **Definitely yes**  Double-blind  (participant,  investigator) | **Definitely yes** | **Probably yes**  There were 9.2% (18/195) and 10.3% (10/97) patients in SGLT2i and control groups with missing outcome data, respectively; missing outcome data were generally balanced across treatment groups, with similar reasons for missing data across groups | **Definitely yes** | **Probably yes**  Generally balanced baseline  characteristics across groups |
| Haring, 2013^45^ | **Definitely yes**  Using a third-party interactive voice and web response system | **Definitely yes**  Randomized,  double-blind | **Definitely yes** Double-blind  (details not reported) | **Definitely yes** | **Probably yes**  There were 8.3% (37/444) and 10.7% (24/225) patients in SGLT2i and control groups with missing outcome data, respectively; missing outcome data were generally balanced across treatment groups, with similar reasons for missing data across groups | **Definitely yes** | **Probably yes**  Generally balanced baseline  characteristics across groups |
| Haring, 2014^46^ | **Definitely yes**  Using a third-party interactive voice and web response system | **Definitely yes**  Randomized,  double-blind | **Definitely yes** Double-blind  (details not reported) | **Definitely yes** | **Probably yes**  There were 6.0% (26/431) and 10.1% (21/207) patients in SGLT2i and control groups with missing outcome data, respectively; missing outcome data were generally balanced across treatment groups, with similar reasons for missing data across groups | **Definitely yes** | **Probably yes**  Generally balanced baseline  characteristics across groups |
| Hattori,2018^47^ | **Probably yes**  Randomized | **Probably yes**  Open-labeled | **Definitely no**  Open-labeled  (participant,  investigator) | **Definitely no** | **Probably yes** | **Definitely yes** | **Probably yes**  Generally balanced baseline  characteristics across groups |
| Kario,2018^48^ | **Definitely yes**  Using a computer-generated randomization  sequence | **Definitely yes**  Randomized, double-blind | **Definitely yes**  Double-blind  (participant,  investigator) | **Definitely yes** | **Definitely yes**  There were 1.6% (1/64) patients in placebo group with missing outcome data. | **Definitely yes** | **Probably yes**  Generally balanced baseline  characteristics across groups |
| Kadowaki, 2014^49^ | **Definitely yes**  Using a computer-generated random sequence and an interactive web response system | **Definitely yes**  Randomized, double-blind | **Definitely yes**  Double-blind  (participant,  investigator) | **Definitely yes** | **Probably yes**  There were 2.3% (10/438) and 8.3% (9/109) patients in SGLT2i and placebo groups with missing outcome data, respectively; missing outcome data were generally balanced across treatment groups, with similar reasons for missing data across groups | **Definitely yes** | **Probably yes**  Generally balanced baseline  characteristics across groups |
| Kawamori,2018^50^ | **Definitely yes**  Using a computer-generated random sequence using a web‐based interactive response system | **Definitely yes**  Randomized, double-blind | **Definitely yes**  Double-blind  (participant,  investigator) | **Definitely yes** | **Probably yes**  There were 2.7% (5/182) and 7.5% (7/93) patients in SGLT2i and control groups with missing outcome data, respectively; missing outcome data were generally balanced across treatment groups, with similar reasons for missing data across groups | **Definitely yes** | **Probably yes**  Generally balanced baseline  characteristics across groups |
| Kovacs, 2014^51^ | **Definitely yes**  Using a computer-generated random sequence and an interactive voice and web response system | **Definitely yes**  Randomized, double-blind | **Definitely yes**  Double-blind  (participant,  investigator) | **Definitely yes** | **Probably yes**  There were 6.9% (23/333)and 10.9% (18/165)patients in SGLT2i and placebo groups with missing outcome data, respectively; missing outcome data were generally balanced across treatment groups, with similar reasons for missing data across groups | **Definitely yes** | **Probably yes**  Generally balanced baseline  characteristics across groups |
| Packer, 2020^52^ | **Definitely yes**  Using an interactive-response system that used a permuted-block design | **Definitely yes**  Randomized, double-blind | **Definitely yes**  Double-blind  (participant,  investigator) | **Definitely yes** | **Definitely yes**  There were 1.2% (22/1863) and 1.1% (20/1867) patients in active agent and placebo groups with missing outcome data, respectively; missing outcome data were generally balanced across treatment groups, with similar reasons for missing data across groups | **Definitely yes** | **Probably yes**  Generally balanced baseline  characteristics across groups |
| Roden, 2013^53^ | **Definitely yes**  an interactive voice and internet-based response system, with a computer-generated random sequence, in block sizes of four | **Definitely yes**  Randomized, double-blind | **Definitely yes**  Double-blind  (participant,  investigator) | **Definitely yes** | **Probably no**  There were 8.5% (38/448)and 18.0% (41/228)patients in SGLT2i and placebo groups with missing outcome data, respectively; missing outcome data were generally balanced across treatment groups, with similar reasons for missing data across groups | **Definitely yes** | **Probably yes**  Generally balanced baseline  characteristics across groups |
| Rosenstock, 2014^54^ | **Definitely yes**  Using a third-party interactive voice-and web-response system | **Definitely yes**  Randomized, double-blind | **Definitely yes**  Double-blind  (participant,  investigator) | **Definitely yes** | **Probably no**  There were 15.6% (59/377) and 16.9% (32/189) patients in SGLT-2i and placebo groups with missing outcome data, respectively; missing outcome data were generally balanced across treatment groups, with similar reasons for missing data across groups | **Definitely yes** | **Probably yes**  Generally balanced baseline  characteristics across groups |
| Rosenstock, 2015^55^ | **Definitely yes**  Using a third-party interactive voice and web response system | **Definitely yes**  Randomized, double-blind | **Definitely yes**  Double-blind  (participant,  investigator) | **Definitely yes** | **Definitely no**  There were 25.3% (82/324) and 30.6% (52/170) patients in SGLT2i and control groups with missing outcome data, respectively; missing outcome data were generally balanced across treatment groups, with similar reasons for missing data across groups | **Definitely yes** | **Probably yes**  Generally balanced baseline  characteristics across groups |
| Ross, 2015^56^ | **Definitely yes**  Using a block allocation method | **Definitely yes**  Randomized, | **Probably yes** | **Probably yes** | **Probably yes**  There were 7.2% (63/876) and 3.7% (4/107) patients in SGLT2i and control groups with missing outcome data, respectively; missing outcome data were generally balanced across treatment groups, with similar reasons for missing data across groups | **Definitely yes** | **Probably yes**  Generally balanced baseline  characteristics across groups |
| Dagogo-Jack, 2018^57^ | **Definitely yes**  Using a computer-  generated randomization schedule | **Definitely yes**  Randomized, double-blind | **Definitely yes**  Double-blind  (participant,  investigator) | **Definitely yes** | **Probably no**  There were 11.3% (35/311) and 14.4% (22/153) patients in active agent and placebo groups with missing outcome data, respectively; missing outcome data were generally balanced across treatment groups, with similar reasons for missing data across groups | **Definitely yes** | **Probably yes**  Generally balanced baseline  characteristics across groups |
| Terra,2017^58^ | **Definitely yes**  Using a computer-generated randomization code using the method of random permuted blocks | **Definitely yes**  Randomized, double-blind | **Definitely yes**  Double-blind  (participant,  investigator) | **Definitely yes** | **Definitely no**  There were 14.0% (43/308) and 22.2% (34/153) patients in active agent and placebo groups with missing outcome data, respectively; missing outcome data were generally balanced across treatment groups, with similar reasons for missing data across groups | **Definitely yes** | **Probably yes**  Generally balanced baseline  characteristics across groups |
| Bando,2016^59^ | **Definitely yes**  Using a EDC system | **Definitely yes**  Randomized, open-label | **Definitely no**  open-label | **Definitely no** | **Definitely yes**  All patients complete the study protocol without any withdrawals | **Definitely yes** | **Probably yes**  Generally balanced baseline  characteristics across groups |
| Han,2018^60^ | **Definitely yes**  Using a computer-  generated randomization schedule | **Definitely yes**  Randomized, double-blind | **Definitely yes**  Double-blind  (participant,  investigator) | **Definitely yes** | **Definitely no**  There were 24.3% (18/74) and 17.4% (12/69) patients in SGLT-2 agent and placebo groups with missing outcome data, respectively; missing outcome data were generally balanced across treatment groups, with similar reasons for missing data across groups | **Definitely yes** | **Probably yes**  Generally balanced baseline  characteristics across groups |
| Ishihara, 2016^61^ | **Probably yes**  Randomized, double-blind | **Probably yes**  Randomized, double-blind | **Definitely yes**  Double-blind  (participant,  investigator) | **Definitely yes** | **Probably yes**  There were 3.4% (6/175) and 12.6% (11/87) patients in SGLT-2 agent and placebo groups with missing outcome data, respectively; missing outcome data were generally balanced across treatment groups, with similar reasons for missing data across groups | **Definitely yes** | **Probably yes**  Generally balanced baseline  characteristics across groups |
| Kashiwagi, 2015^62^ | **Definitely yes**  Using a block allocation method | **Definitely yes**  Randomized, double-blind | **Definitely yes**  Double-blind  (participant,  investigator) | **Definitely yes** | **Probably yes**  There were 10.1% (12/119) and 8.7% (4/46) patients in active agent and placebo groups with missing outcome data, respectively; missing outcome data were generally balanced across treatment groups, with similar reasons for missing data across groups | **Definitely yes** | **Probably yes**  Generally balanced baseline  characteristics across groups |
| Seino, 2018^63^ | **Definitely yes**  Using a computer-generated randomization schedule | **Definitely yes**  Randomized, double-blind | **Definitely yes**  Double-blind  (participant,  investigator) | **Definitely yes** | **Probably no**  There were 1.9% (3/159) and 13.5% (10/74) patients in SGLT-2 and placebo groups with missing outcome data, respectively; missing outcome data were generally balanced across treatment groups, with similar reasons for missing data across groups | **Definitely yes** | **Probably yes**  Generally balanced baseline  characteristics across groups |
| Buse, 2018^64^ | **Probably yes**  Randomized,  double-blind | **Probably yes**  Randomized,  double-blind | **Definitely yes** Double-blind  (details not reported) | **Definitely yes** | **Probably no**  There were 14.5% (76/525) and 18.7% (50/268) patients in SGLT2i and control groups with missing outcome data, respectively; missing outcome data were generally balanced across treatment groups, with similar reasons for missing data across groups | **Definitely yes** | **Probably yes**  Generally balanced baseline  characteristics across groups |
| Kaku, 2014^65^ | **Definitely yes**  Using a central web-based system | **Definitely yes**  Randomized, double-blind | **Definitely yes**  Double-blind  (participant,  investigator) | **Definitely yes** | **Probably no**  There were 7.3% (13/178) and 15.8% (9/57) patients in SGLT-2 and placebo groups with missing outcome data, respectively; missing outcome data were generally balanced across treatment groups, with similar reasons for missing data across groups | **Definitely yes** | **Probably yes**  Generally balanced baseline  characteristics across groups |
| Terauchi, 2017^66^ | **Definitely yes**  Using an interactive web response system (IWRS) | **Definitely yes**  Randomized, double-blind | **Definitely yes**  Double-blind  (participant,  investigator) | **Definitely yes** | **Definitely yes**  There were 2.8% (4/141) and 2.8% (2/70) patients in SGLT-2 and placebo groups with missing outcome data, respectively; missing outcome data were generally balanced across treatment groups, with similar reasons for missing data across groups | **Definitely yes** | **Probably yes**  Generally balanced baseline  characteristics across groups |
| Schernthaner 2013^67^ | **Definitely yes**  Using an interactive voice response system/interactive web response system | **Definitely yes**  Randomized, double-blind | **Definitely yes**  Double-blind  (participant,  investigator) | **Definitely yes** | **Definitely no**  There were 32.8% (124/378) and 44.4% (168/378) patients in SGLT2i and control groups with missing outcome data, respectively; missing outcome data were generally balanced across treatment groups, with similar reasons for missing data across groups | **Definitely yes** | **Probably yes**  Generally balanced baseline  characteristics across groups |
| Nauck 2011^68^ | **Definitely yes**  Using a predefined computer-generated randomization scheme | **Definitely yes**  Randomized, double-blind | **Definitely yes**  Double-blind  (participant,  investigator) | **Definitely yes** | **Definitely no**  There were 20.7% (84/406) and 23.0% (94/408) patients in SGLT2i and control groups with missing outcome data, respectively; missing outcome data were generally balanced across treatment groups, with similar reasons for missing data across groups | **Definitely yes** | **Probably yes**  Generally balanced baseline  characteristics across groups |
| Müller-Wieland 2018^69^ | **Definitely yes**  Using a computer-generated random sequence | **Definitely yes**  Randomized, double-blind | **Definitely yes**  Double-blind  (participant,  investigator) | **Definitely yes** | **Probably yes**  There were 7.5% (47/626) and 8.0% (25/313) patients in SGLT2i and control groups with missing outcome data, respectively; missing outcome data were generally balanced across treatment groups, with similar reasons for missing data across groups | **Definitely yes** | **Probably yes**  Generally balanced baseline  characteristics across groups |
| Cho 2018^70^ | **Probably yes**  Randomized | **Probably yes**  Open-labeled | **Definitely no**  Open-labeled  (participant,  investigator) | **Definitely no** | **Definitely yes**  There were 0% (0/36) and 0% (0/35) patients in SGLT2i and control groups with missing outcome data, respectively; missing outcome data were generally balanced across treatment groups, with similar reasons for missing data across groups | **Definitely yes** | **Probably yes**  Generally balanced baseline  characteristics across groups |
| Scott 2018^71^ | **Definitely yes**  Using an interactive voice response system | **Definitely yes**  Randomized, double-blind | **Definitely yes**  Double-blind  (details not reported) | **Definitely yes** | **Definitely yes**  There were 3.6% (11/307) and 2.6% (8/307) patients in SGLT2i and control groups with missing outcome data, respectively; missing outcome data were generally balanced across treatment groups, with similar reasons for missing data across groups | **Definitely yes** | **Probably yes**  Generally balanced baseline  characteristics across groups |
| Shimizu 2018^72^ | **Probably yes**  Randomized | **Probably yes**  Open-labeled | **Definitely no**  Open-labeled  (participant,  investigator) | **Definitely no** | **Probably no**  There were 5.7% (2/35) and 14.3% (4/28) patients in SGLT2i and control groups with missing outcome data, respectively; missing outcome data were generally balanced across treatment groups, with similar reasons for missing data across groups | **Definitely yes** | **Probably yes**  Generally balanced baseline  characteristics across groups |
| Hayashi 2017^73^ | **Probably yes**  Randomized | **Probably yes**  Open-labeled | **Definitely no**  Open-labeled  (participant,  investigator) | **Definitely no** | **Definitely yes**  There were 0% (0/40) and 0% (0/40) patients in SGLT2i and control groups with missing outcome data, respectively; missing outcome data were generally balanced across treatment groups, with similar reasons for missing data across groups | **Definitely yes** | **Probably yes**  Generally balanced baseline  characteristics across groups |
| Zinman 2015^74^ | **Definitely yes**  Using a computer-generated random-sequence and interactive voice- and Web-response system | **Definitely yes**  Randomized, double-blind | **Definitely yes**  Double-blind  (details not reported) | **Definitely yes** | **Definitely yes**  There were 3.1% (144/4687) and 2.9% (67/2333) patients in SGLT2i and control groups with missing outcome data, respectively; missing outcome data were generally balanced across treatment groups, with similar reasons for missing data across groups | **Definitely yes** | **Probably yes**  Generally balanced baseline  characteristics across groups |
| Araki 2015^75^ | **Definitely yes**  Using a computer-generated random sequence via an interactive web-based response system. | **Definitely yes**  Randomized | **Definitely no**  Open-labeled  (participant,  investigator) | **Definitely no** | **Probably yes** | **Definitely yes** | **Probably yes**  Generally balanced baseline  characteristics across groups |
| Hollander 2019^76^ | **Definitely yes**  Using a computer-generated randomization schedule. | **Definitely yes**  Randomized, double-blind | **Definitely yes**  Double-blind  (participant,  investigator) | **Definitely yes** | **Definitely no**  There were 22.9% (202/881) and 24.8% (108/435) patients in SGLT2i and control groups with missing outcome data, respectively; missing outcome data were generally balanced across treatment groups, with similar reasons for missing data across groups | **Definitely yes** | **Probably yes**  Generally balanced baseline  characteristics across groups |
| Tsurutani 2018^77^ | **Probably yes**  Randomized | **Probably yes**  Open-labeled | **Definitely no**  Open-labeled  (details not reported) | **Definitely no** | **Definitely yes**  There were 5% (3/60) and 3.4% (2/59) patients in SGLT2i and control groups with missing outcome data, respectively; missing outcome data were generally balanced across treatment groups, with similar reasons for missing data across groups | **Definitely yes** | **Probably yes**  Generally balanced baseline  characteristics across groups |
| Takashima,2018^78^ | **Probably yes**  Randomized | **Probably yes**  Open-labeled | **Definitely no**  Open-labeled  (participant,  investigator) | **Definitely no** | **Definitely yes**  There were 4.8% (1/21) and 4.8% (1/21) patients in SGLT2i and control groups with missing outcome data, respectively; missing outcome data were generally balanced across treatment groups, with similar reasons for missing data across groups | **Definitely yes** | **Probably yes**  Generally balanced baseline  characteristics across groups |
| Fioretto,2018^79^ | **Definitely yes**  Using an interactive voice response system (IVRS) or interactive web response system (IWRS) | **Definitely yes**  Randomized, double-blind | **Definitely yes**  Double-blind  (participant,  investigator) | **Definitely yes** | **Probably yes**  There were 6.9% (11/160) and 9.3% (15/161) patients in SGLT-2 and placebo groups with missing outcome data, respectively; missing outcome data were generally balanced across treatment groups, with similar reasons for missing data across groups | **Definitely yes** | **Probably yes**  Generally balanced baseline  characteristics across groups |
| Pollock, 2019^80^ | **Definitely yes**  Using the sponsor’s interactive voice–web response system | **Definitely yes**  Randomized,  double-blind | **Definitely yes**  Double-blind  (participant,  investigator) | **Definitely yes** | **Probably yes**  There were 9.3% (14/151) and 6.5% (10/153) patients in SGLT2i and control groups with missing outcome data, respectively; missing outcome data were generally balanced across treatment groups, with similar reasons for missing data across groups | **Definitely yes** | **Probably yes**  Generally balanced baseline  characteristics across groups |
| Grunberger, 2017^81^ | **Definitely yes**  Using an  interactive voice response system/integrated web  response system | **Definitely yes**  Randomized, double-blind | **Definitely yes**  Double-blind  (participant,  investigator) | **Definitely yes** | **Probably no**  There were 10.5% (33/314) and 11.0% (17/154) patients in active agent and placebo groups with missing outcome data, respectively; missing outcome data were generally balanced across treatment groups, with similar reasons for missing data across groups | **Definitely yes** | **Probably yes**  Generally balanced baseline  characteristics across groups |
| Haneda, 2016^82^ | **Probably yes**  Randomized,  double-blind | **Probably yes**  Randomized,  double-blind | **Definitely yes**  Double-blind  (participant,  investigator) | **Definitely yes** | **Probably yes**  There were 6.3% (6/95) and 4% (2/50) patients in SGLT2i and control groups with missing outcome data, respectively; missing outcome data were generally balanced across treatment groups, with similar reasons for missing data across groups | **Definitely yes** | **Probably yes**  Generally balanced baseline  characteristics across groups |
| Yale, 2013^83^ | **Definitely yes**  Using an interactive voice response system/interactive web response system. | **Definitely yes**  Randomized,  double-blind | **Definitely yes** Double-blind  (details not reported) | **Definitely yes** | **Probably no**  There were 12.3% (22/179) and 14.4% (13/90) patients in SGLT2i and control groups with missing outcome data, respectively; missing outcome data were generally balanced across treatment groups, with similar reasons for missing data across groups | **Definitely yes** | **Probably yes**  Generally balanced baseline  characteristics across groups |
| Heerspink, 2020^84^ | **Definitely yes**  Using an interactive voice-response or Web-response system | **Definitely yes**  Randomized, double-blind | **Definitely yes**  Double-blind  (participant,  investigator) | **Definitely yes** | **Definitely yes**  There were 0.47% (10/2152) and 0.23% (5/2152) patients in SGLT2i and control groups with missing outcome data, respectively; missing outcome data were generally balanced across treatment groups, with similar reasons for missing data across groups | **Definitely yes** | **Probably yes**  Generally balanced baseline  characteristics across groups |
| Bhatt 2021^85^ | **Probably yes**  Randomized,  double-blind | **Probably yes**  Randomized,  double-blind | **Definitely yes**  Double-blind  (participant,  investigator) | **Definitely yes** | **Definitely yes**  There were 1.1% (60/5292) and 1.5% (82/5292) patients in SGLT2i and control groups with missing outcome data, respectively; missing outcome data were generally balanced across treatment groups, with similar reasons for missing data across groups | **Definitely yes** | **Probably yes**  Generally balanced baseline  characteristics across groups |
| Kohan,2013^86^ | **Probably yes**  Randomized,  double-blind | **Probably yes**  Randomized,  double-blind | **Definitely yes** Double-blind  (details not reported) | **Definitely yes** | **Definitely no**  There were 42.9% (72/168) and 48.8% (41/84) patients in SGLT2i and placebo groups with missing outcome data, respectively; missing outcome data were generally balanced across treatment groups, with similar reasons for missing data across groups | **Definitely yes** | **Probably yes**  Generally balanced baseline  characteristics across groups |

Table S3. Subgroup analyses of efficacy among different drug categories.

| Drug type | Renal function | Weighted mean difference | 95%CI | P (over effect) | P (subgroup difference) |
| --- | --- | --- | --- | --- | --- |
| HbA1c (%) |  |  |  |  |  |
| CANA | ≥90 | -0.77 | -0.91, -0.64 | ＜0.001 |  |
|  | 60-89 | -0.81 | -0.93, -0.68 | ＜0.001 |  |
|  | 45-59 | -0.20 | -0.69, 0.29 | 0.42 |  |
|  | ＜45 | NA | NA | NA |  |
|  | total | -0.79 | -0.90, -0.68 | ＜0.001 | 0.06 |
| DAPA | ≥90 | -0.49 | -0.68, -0.30 | ＜0.001 |  |
|  | 60-89 | -0.32 | -0.45, -0.20 | ＜0.001 |  |
|  | 45-59 | -0.27 | -0.51, -0.04 | 0.02 |  |
|  | ＜45 | -0.27 | -0.97, 0.43 | 0.45 |  |
|  | total | -0.36 | -0.47, -0.25 | ＜0.001 | 0.42 |
| EMPA | ≥90 | -0.32 | -0.56, -0.07 | 0.01 |  |
|  | 60-89 | -0.56 | -0.65, -0.46 | ＜0.001 |  |
|  | 45-59 | -0.44 | -0.61, -0.27 | ＜0.001 |  |
|  | ＜45 | 0.48 | -0.06, 1.02 | 0.08 |  |
|  | total | -0.49 | -0.57, -0.40 | ＜0.001 | ＜0.001 |
| ERTU | ≥90 | -0.67 | -0.85, -0.49 | ＜0.001 |  |
|  | 60-89 | -0.60 | -0.93, -0.26 | ＜0.001 |  |
|  | 45-59 | -0.09 | -0.24, 0.06 | 0.23 |  |
|  | ＜45 | NA | NA | NA |  |
|  | total | -0.56 | -0.76, -0.37 | ＜0.001 | ＜0.001 |
| IPRA | ≥90 | -0.48 | -0.69, -0.27 | ＜0.001 |  |
|  | 60-89 | -0.80 | -1.13, -0.48 | ＜0.001 |  |
|  | 45-59 | NA | NA | NA |  |
|  | ＜45 | NA | NA | NA |  |
|  | total | -0.71 | -0.99, -0.44 | ＜0.001 | 0.1 |
| SOTA | ≥90 | -0.38 | -0.52, -0.24 | ＜0.001 |  |
|  | 60-89 | -0.28 | -0.37, -0.19 | ＜0.001 |  |
|  | 45-59 | NA | NA | NA |  |
|  | ＜45 | -0.43 | -0.48, -0.38 | ＜0.001 |  |
|  | total | -0.36 | -0.43, -0.28 | ＜0.001 | 0.01 |
| LUSEO | ≥90 | NA | NA | NA |  |
|  | 60-89 | -1.13 | -1.34, -0.92 | ＜0.001 |  |
|  | 45-59 | -0.2 | -0.42, 0.02 | 0.08 |  |
|  | ＜45 | NA | NA | NA |  |
|  | total | -0.67 | -1.58, 0.25 | 0.15 | ＜0.001 |
| TOFO | ≥90 | NA | NA | NA |  |
|  | 60-89 | -0.91 | -1.03, -0.78 | ＜0.001 |  |
|  | 45-59 | NA | NA | NA |  |
|  | ＜45 | NA | NA | NA |  |
|  | total | -0.91 | -1.03, -0.78 | ＜0.001 | NA |
| Weight (kg) |  |  |  |  |  |
| CANA | ≥90 | -2.5 | -3.05, -1.95 | ＜0.001 |  |
|  | 60-89 | -1.73 | -2.05, -1.42 | ＜0.001 |  |
|  | 45-59 |  | NA | NA |  |
|  | ＜45 |  | NA | NA |  |
|  | total | -1.86 | -2.22, -1.50 | ＜0.001 | 0.02 |
| DAPA | ≥90 | -2.07 | -3.43, -0.71 | 0.003 |  |
|  | 60-89 | -1.97 | -2.75, -1.19 | ＜0.001 |  |
|  | 45-59 | -1.25 | -2.08, -0.42 | 0.003 |  |
|  | ＜45 | -3.35 | -4.86, -1.85 | ＜0.001 |  |
|  | total | -2.04 | -2.74, -1.34 | ＜0.001 | 0.11 |
| EMPA | ≥90 | -2.17 | -2.64, -1.69 | ＜0.001 |  |
|  | 60-89 | -1.93 | -2.11, -1.75 | ＜0.001 |  |
|  | 45-59 | -1.17 | -1.75, -0.59 | ＜0.001 |  |
|  | ＜45 | -1.00 | -2.57, 0.57 | 0.21 |  |
|  | total | -1.94 | -2.11, -1.77 | ＜0.001 | 0.03 |
| ERTU | ≥90 | -1.88 | -2.12, -1.64 | ＜0.001 |  |
|  | 60-89 | -2.56 | -3.36, -1.75 | ＜0.001 |  |
|  | 45-59 | NA | NA | NA |  |
|  | ＜45 | NA | NA | NA |  |
|  | total | -2.27 | -2.74, -1.80 | ＜0.001 | 0.12 |
| IPRA | ≥90 | -1.83 | -3.12, | 0.006 |  |
|  | 60-89 | -1.66 | -2.16, -1.15 | ＜0.001 |  |
|  | 45-59 | NA | NA | NA |  |
|  | ＜45 | NA | NA | NA |  |
|  | total | -1.68 | -2.11, -1.25 | ＜0.001 | 0.81 |
| SOTA | ≥90 | -2.83 | -3.14, -2.51 | ＜0.001 |  |
|  | 60-89 | -3.73 | -4.89, -2.57 | ＜0.001 |  |
|  | 45-59 | NA | NA | NA |  |
|  | ＜45 | -1.16 | -1.27, -1.05 | ＜0.001 |  |
|  | total | -2.73 | -3.82, -1.65 | ＜0.001 | ＜0.001 |
| LUSEO | ≥90 | NA | NA | NA |  |
|  | 60-89 | -1.27 | -1.69, -0.85 | ＜0.001 |  |
|  | 45-59 | -1.27 | -1.84, -0.7 | ＜0.001 |  |
|  | ＜45 | NA | NA | NA |  |
|  | total | -1.27 | -1.61, -0.93 | ＜0.001 | 1.00 |
| TOFO | ≥90 | NA | NA | NA |  |
|  | 60-89 | -2.06 | -2.69, -1.42 | ＜0.001 |  |
|  | 45-59 | NA | NA | NA |  |
|  | ＜45 | NA | NA | NA |  |
|  | total | -2.06 | -2.69, -1.42 | ＜0.001 | NA |
| SBP (mmHg) |  |  |  |  |  |
| CANA | ≥90 | -4.61 | -5.77, -3.45 | ＜0.001 |  |
|  | 60-89 | -4.58 | -5.49, -3.67 | ＜0.001 |  |
|  | 45-59 | -3.60 | -4.90, -2.30 | ＜0.001 |  |
|  | ＜45 | -5.95 | -8.89, -3.01 | ＜0.001 |  |
|  | total | -4.51 | -5.18, -3.84 | ＜0.001 | 0.43 |
| DAPA | ≥90 | -3.49 | -5.24, -1.74 | ＜0.001 |  |
|  | 60-89 | -2.75 | -3.94, -1.56 | ＜0.001 |  |
|  | 45-59 | -2.97 | -6.09, 0.15 | 0.06 |  |
|  | ＜45 | -5.61 | -8.97, -2.24 | 0.001 |  |
|  | total | -3.17 | -4.37, -1.97 | ＜0.001 | 0.45 |
| EMPA | ≥90 | -3.61 | -4.72, -2.50 | ＜0.001 |  |
|  | 60-89 | -3.84 | -4.67, -3.02 | ＜0.001 |  |
|  | 45-59 | -4.30 | -7.13, -1.47 | 0.003 |  |
|  | ＜45 | -12.20 | -19.75, -4.65 | 0.002 |  |
|  | total | -3.87 | -4.55, -3.20 | ＜0.001 | 0.17 |
| ERTU | ≥90 | -3.80 | -4.77, -2.83 | ＜0.001 |  |
|  | 60-89 | -3.84 | -4.69, -2.98 | ＜0.001 |  |
|  | 45-59 | NA | NA | NA |  |
|  | ＜45 | NA | NA | NA |  |
|  | total | -3.82 | -4.46, -3.18 | ＜0.001 | 0.96 |
| IPRA | ≥90 | -5.5 | -9.56, -1.44 | 0.008 |  |
|  | 60-89 | -2.23 | -3.97, -0.50 | 0.01 |  |
|  | 45-59 | NA | NA | NA |  |
|  | ＜45 | NA | NA | NA |  |
|  | total | -2.74 | -4.33, -1.14 | ＜0.001 | 0.15 |
| SOTA | ≥90 | -3.45 | -4.36, -2.55 | ＜0.001 |  |
|  | 60-89 | -3.59 | -5.04, -2.15 | ＜0.001 |  |
|  | 45-59 | NA | NA | NA |  |
|  | ＜45 | -2.44 | -2.84, -2.04 | ＜0.001 |  |
|  | total | -2.67 | -3.02, -2.31 | ＜0.001 | 0.06 |
| LUSEO | ≥90 | NA | NA | NA |  |
|  | 60-89 | -4.1 | -7.48, -0.72 | 0.02 |  |
|  | 45-59 | -2.6 | -7.91, 2.71 | 0.34 |  |
|  | ＜45 | NA | NA | NA |  |
|  | total | -3.67 | -6.52, -0.82 | 0.01 | 0.64 |
| TOFO | ≥90 | NA | NA | NA |  |
|  | 60-89 | -3.88 | -6.04, -1.72 | ＜0.001 |  |
|  | 45-59 | NA | NA | NA |  |
|  | ＜45 | NA | NA | NA |  |
|  | total | -3.88 | -6.04, -1.72 | ＜0.001 | NA |
| DBP (mmHg) |  |  |  |  |  |
| CANA | ≥90 | -1.74 | -2.37, -1.12 | ＜0.001 |  |
|  | 60-89 | -1.83 | -2.19, -1.47 | ＜0.001 |  |
|  | 45-59 | -2.20 | -3.01, -1.39 | ＜0.001 |  |
|  | ＜45 | -1.65 | -3.41, 0.11 | 0.07 |  |
|  | total | -1.86 | -2.14, -1.57 | ＜0.001 | 0.83 |
| DAPA | ≥90 | -1.80 | -2.02, -1.59 | ＜0.001 |  |
|  | 60-89 | -0.73 | -1.45, -0.01 | 0.05 |  |
|  | 45-59 | NA | NA | NA |  |
|  | ＜45 | -0.47 | -2.62, 1.68 | 0.67 |  |
|  | total | -1.70 | -1.91, -1.50 | ＜0.001 | 0.01 |
| EMPA | ≥90 | -2.17 | -2.88, -1.46 | ＜0.001 |  |
|  | 60-89 | -1.70 | -2.02, -1.38 | ＜0.001 |  |
|  | 45-59 | -1.50 | -3.20, 0.20 | 0.08 |  |
|  | ＜45 | -5.70 | -9.69, -1.71 | 0.005 |  |
|  | total | -1.79 | -2.08, -1.51 | ＜0.001 | 0.16 |
| ERTU | ≥90 | -1.23 | -1.83, -0.63 | ＜0.001 |  |
|  | 60-89 | -1.36 | -1.91, -0.81 | ＜0.001 |  |
|  | 45-59 | NA | NA | NA |  |
|  | ＜45 | NA | NA | NA |  |
|  | total | -1.30 | -1.70, -0.89 | ＜0.001 | 0.76 |
| IPRA | ≥90 | -2.7 | -5.32, -0.08 | 0.04 |  |
|  | 60-89 | -1.10 | -2.33, 0.14 | 0.08 |  |
|  | 45-59 | NA | NA | NA |  |
|  | ＜45 | NA | NA | NA |  |
|  | total | -1.39 | -2.51, -0.27 | 0.01 | 0.28 |
| SOTA | ≥90 | -1.20 | -1.81, -0.59 | ＜0.001 |  |
|  | 60-89 | -1.90 | -2.80, -1.00 | ＜0.001 |  |
|  | 45-59 | NA | NA | NA |  |
|  | ＜45 | -0.80 | -1.05, -0.55 | ＜0.001 |  |
|  | total | -0.92 | -1.15, -0.70 | ＜0.001 | 0.04 |
| LUSEO | ≥90 | NA | NA | NA |  |
|  | 60-89 | -2.50 | -4.84, -0.16 | 0.04 |  |
|  | 45-59 | 0.30 | -3.30, 3.90 | 0.87 |  |
|  | ＜45 | NA | NA | NA |  |
|  | total | -1.67 | -3.63, 0.30 | 0.10 | 0.20 |
| TOFO | ≥90 | NA | NA | NA |  |
|  | 60-89 | -2.76 | -4.28, -1.24 | ＜0.001 |  |
|  | 45-59 | NA | NA | NA |  |
|  | ＜45 | NA | NA | NA |  |
|  | total | -2.76 | -4.28, -1.24 | ＜0.001 | NA |
| eGFR (mL/min/1.73 m^2^) | |  |  |  |  |
| CANA | ≥90 | -1.78 | -5.90, 2.33 | 0.40 |  |
|  | 60-89 | NA | NA | NA |  |
|  | 45-59 | 4.10 | 0.75, 7.45 | 0.02 |  |
|  | ＜45 | NA | NA | NA |  |
|  | total | 0.12 | -4.29, 4.54 | 0.96 | 0.03 |
| DAPA | ≥90 | -1.99 | -4.24, 0.27 | 0.08 |  |
|  | 60-89 | 0.05 | -1.16, 1.25 | 0.94 |  |
|  | 45-59 | NA | NA | NA |  |
|  | ＜45 | 0.88 | 0.47, 1.29 | ＜0.001 |  |
|  | total | -0.35 | -1.24, 0.55 | 0.45 | 0.03 |
| EMPA | ≥90 | 1.15 | -0.17, 2.46 | 0.09 |  |
|  | 60-89 | -0.33 | -0.90, 0.24 | 0.26 |  |
|  | 45-59 | -2.50 | -4.08, -0.92 | 0.002 |  |
|  | ＜45 | -0.30 | -2.99, 2.39 | 0.83 |  |
|  | total | -0.26 | -0.78, 0.26 | 0.33 | 0.007 |
| ERTU | ≥90 | 1.54 | -0.43, 3.52 | 0.12 |  |
|  | 60-89 | NA | NA | NA |  |
|  | 45-59 | NA | NA | NA |  |
|  | ＜45 | NA | NA | NA |  |
|  | total | 1.54 | -0.43, 3.52 | 0.12 | NA |
| IPRA | ≥90 | 8.42 | -5.15, 21.99 | 0.22 |  |
|  | 60-89 | -1.21 | -2.44, 0.01 | 0.05 |  |
|  | 45-59 | NA | NA | NA |  |
|  | ＜45 | NA | NA | NA |  |
|  | total | -1.14 | -2.35, 0.08 | 0.07 | 0.17 |
| SOTA | ≥90 | -0.78 | -1.82, 0.25 | 0.14 |  |
|  | 60-89 | -1.25 | -2.66, 0.16 | 0.08 |  |
|  | 45-59 | NA | NA | NA |  |
|  | ＜45 | -1.25 | -1.51, -0.99 | ＜0.001 |  |
|  | total | -1.22 | -1.47, -0.97 | ＜0.001 | 0.69 |
| LUSEO | ≥90 | NA | NA | NA |  |
|  | 60-89 | -1.30 | -3.78, 1.18 | 0.30 |  |
|  | 45-59 | -2.50 | -4.30, -0.70 | 0.007 |  |
|  | ＜45 | NA | NA | NA |  |
|  | total | -2.09 | -3.54, -0.63 | 0.005 | 0.44 |
| TOFO | ≥90 | NA | NA | NA |  |
|  | 60-89 | -2.87 | -4.84, -0.89 | 0.004 |  |
|  | 45-59 | NA | NA | NA |  |
|  | ＜45 | NA | NA | NA |  |
|  | total | -2.87 | -4.84, -0.89 | 0.004 | NA |

HbA1c, glycosylated hemoglobin; SBP, systolic blood pressure; DBP, diastolic blood pressure; eGFR, estimated glomerular filtrationrate; CANA, canagliflozin; DAPA, dapagliflozin; EMPA, empagliflozin; ERTU, ertugliflozin; IPRA, ipragliflozin; LUSEO, luseogliflozin; SOTA, sotagliflozin; TOFO, tofogliflozin; NA, not available.

Table S4. Subgroup analyses of efficacy among follow-up period.

| Follow-up period | Renal function | Weighted mean difference | 95%CI | P (over effect) | P (subgroup difference) |
| --- | --- | --- | --- | --- | --- |
| HbA1c (%) |  |  |  |  |  |
| ＜1y | ≥90 | -0.59 | -0.68, -0.50 | ＜0.001 |  |
|  | 60-89 | -0.70 | -0.79, -0.62 | ＜0.001 |  |
|  | 45-59 | -0.16 | -0.27, -0.05 | 0.005 |  |
|  | ＜45 |  |  |  |  |
|  | total | -0.65 | -0.71, -0.58 | ＜0.001 | ＜0.001 |
| ＞1y | ≥90 | -0.13 | -0.28, 0.02 | 0.08 |  |
|  | 60-89 | -0.26 | -0.36, -0.16 | ＜0.001 |  |
|  | 45-59 | -0.41 | -0.57, -0.25 | ＜0.001 |  |
|  | ＜45 | -0.13 | -0.67, 0.42 | 0.65 |  |
|  | total | -0.23 | -0.32, -0.15 | ＜0.001 | 0.09 |
| Weight (kg) |  |  |  |  |  |
| ＜1y | ≥90 | -2.09 | -2.72, -1.47 | ＜0.001 |  |
|  | 60-89 | -1.74 | -1.89, -1.60 | ＜0.001 |  |
|  | 45-59 | -1.26 | -1.73, -0.8 | ＜0.001 |  |
|  | ＜45 |  |  |  |  |
|  | total | -1.86 | -2.17, -1.56 | ＜0.001 | 0.08 |
| ＞1y | ≥90 | -2.24 | -2.86, -1.62 | ＜0.001 |  |
|  | 60-89 | -2.67 | -3.40, -1.94 | ＜0.001 |  |
|  | 45-59 | -1.17 | -1.75, -0.59 | ＜0.001 |  |
|  | ＜45 | -1.88 | -3.04, -0.72 | 0.001 |  |
|  | total | -2.47 | -3.01, -1.93 | ＜0.001 | 0.009 |
| SBP (mmHg) |  |  |  |  |  |
| ＜1y | ≥90 | -3.95 | -4.83, -3.06 | ＜0.001 |  |
|  | 60-89 | -3.73 | -4.23, -3.24 | ＜0.001 |  |
|  | 45-59 | -2.87 | -5.56, -0.19 | 0.04 |  |
|  | ＜45 | -5.95 | -8.89, -3.01 | ＜0.001 |  |
|  | total | -3.87 | -4.39, -3.36 | ＜0.001 | 0.44 |
| ＞1y | ≥90 | -3.68 | -4.49, -2.87 | ＜0.001 |  |
|  | 60-89 | -3.82 | -4.75, -2.88 | ＜0.001 |  |
|  | 45-59 | -3.72 | -4.91, -2.54 | ＜0.001 |  |
|  | ＜45 | -5.32 | -8.96, -1.67 | 0.004 |  |
|  | total | -3.79 | -4.39, -3.19 | ＜0.001 | 0.86 |
| DBP (mmHg) |  |  |  |  |  |
| ＜1y | ≥90 | -1.76 | -1.95, -1.57 | ＜0.001 |  |
|  | 60-89 | -1.52 | -1.76, -1.27 | ＜0.001 |  |
|  | 45-59 | 0.30 | -3.30, 3.90 | 0.87 |  |
|  | ＜45 | -1.65 | -3.41, 0.11 | 0.07 |  |
|  | total | -1.66 | -1.81, -1.52 | ＜0.001 | 0.33 |
| ＞1y | ≥90 | -1.46 | -1.99, -0.94 | ＜0.001 |  |
|  | 60-89 | -1.90 | -2.31, -1.49 | ＜0.001 |  |
|  | 45-59 | -2.07 | -2.80, -1.34 | ＜0.001 |  |
|  | ＜45 | -1.30 | -2.95, 0.35 | 0.12 |  |
|  | total | -1.71 | -2.06, -1.37 | ＜0.001 | 0.44 |
| eGFR (mL/min/1.73 m^2^) |  |  |  |  |  |
| ＜1y | ≥90 | -0.19 | -1.37, 0.98 | 0.75 |  |
|  | 60-89 | -0.82 | -1.27, -0.38 | ＜0.001 |  |
|  | 45-59 | -2.50 | -4.30, -0.70 | 0.007 |  |
|  | ＜45 | NA | NA | NA |  |
|  | total | -0.77 | -1.22, -0.32 | ＜0.001 | 0.11 |
| ＞1y | ≥90 | -1.02 | -2.68, 0.63 | 0.23 |  |
|  | 60-89 | 0.24 | -0.59, 1.08 | 0.57 |  |
|  | 45-59 | 0.63 | -5.83, 7.09 | 0.85 |  |
|  | ＜45 | -0.21 | -1.77, 1.34 | 0.79 |  |
|  | total | -0.06 | -0.70, 0.58 | 0.85 | 0.60 |

HbA1c, glycosylated hemoglobin; SBP, systolic blood pressure; DBP, diastolic blood pressure; eGFR, estimated glomerular filtrationrate.

Table S5. Subgroup analyses of different AEs.

| AEs | Renal function | OR | 95%CI | P (over effect) | P (subgroup difference) |
| --- | --- | --- | --- | --- | --- |
| urinary tract infection | ≥90 | 1.18 | 0.99, 1.39 | 0.06 |  |
|  | 60-89 | 1.06 | 0.97, 1.15 | 0.22 |  |
|  | 45-59 | 0.92 | 0.61, 1.38 | 0.68 |  |
|  | ＜45 | 1.05 | 0.94, 1.19 | 0.38 |  |
|  | total | 1.07 | 1.00, 1.14 | 0.04 | 0.60 |
| genital tract infection | ≥90 | 3.67 | 2.85, 4.73 | ＜0.001 |  |
|  | 60-89 | 3.95 | 3.31, 4.71 | ＜0.001 |  |
|  | 45-59 | 3.24 | 1.24, 8.46 | 0.02 |  |
|  | ＜45 | 2.83 | 2.04, 3.93 | ＜0.001 |  |
|  | total | 3.69 | 3.23, 4.20 | ＜0.001 | 0.37 |
| amputation | ≥90 | 1.48 | 0.06, 36.49 | 0.81 |  |
|  | 60-89 | 1.19 | 0.68, 2.09 | 0.54 |  |
|  | 45-59 | 3.08 | 0.12, 76.30 | 0.49 |  |
|  | ＜45 | 0.93 | 0.66, 1.30 | 0.67 |  |
|  | total | 1.01 | 0.76, 1.34 | 0.96 | 0.78 |
| hypovolemia | ≥90 | 1.65 | 1.08, 2.53 | 0.02 |  |
|  | 60-89 | 1.12 | 0.99, 1.26 | 0.06 |  |
|  | 45-59 | 1.65 | 0.82, 3.30 | 0.16 |  |
|  | ＜45 | 1.37 | 1.18, 1.60 | ＜0.001 |  |
|  | total | 1.24 | 1.13, 1.35 | ＜0.001 | 0.07 |
| orthostatic hypotension | ≥90 | 2.77 | 0.30, 25.75 | 0.37 |  |
|  | 60-89 | 1.23 | 0.54, 3.76 | 0.62 |  |
|  | 45-59 | NA | NA | NA |  |
|  | ＜45 | 1.52 | 0.06, 37.71 | 0.80 |  |
|  | total | 1.38 | 0.66, 2.89 | 0.39 | 0.80 |
| bone fracture | ≥90 | 0.59 | 0.29, 1.18 | 0.13 |  |
|  | 60-89 | 0.98 | 0.82, 1.17 | 0.81 |  |
|  | 45-59 | 0.36 | 0.11, 1.13 | 0.08 |  |
|  | ＜45 | 1.10 | 0.90, 1.35 | 0.35 |  |
|  | total | 0.99 | 0.87, 1.13 | 0.93 | 0.10 |
| DKA | ≥90 | 6.47 | 2.49,16.83 | ＜0.001 |  |
|  | 60-89 | 2.78 | 1.51, 5. 14 | 0.001 |  |
|  | 45-59 | 3.08 | 0.12, 76.30 | 0.49 |  |
|  | ＜45 | 1.27 | 0.80, 2.03 | 0.31 |  |
|  | total | 2.23 | 1.59, 3.11 | ＜0.001 | 0.01 |
| hypoglycemia | ≥90 | 1.07 | 0.57, 2.04 | 0.83 |  |
|  | 60-89 | 0.96 | 0.74, 1.23 | 0.72 |  |
|  | 45-59 | 0.94 | 0.69, 1.27 | 0.67 |  |
|  | ＜45 | 0.79 | 0.57, 1.11 | 0.17 |  |
|  | total | 0.96 | 0.78, 1.18 | 0.69 | 0.78 |

AEs, adverse effect; OR, odds ratio; DKA, diabetic ketoacidosis.

Table S6. Subgroup analyses of different AEs in placebo-controlled RCTs.

| AEs | renal function | OR | 95%CI | P (over effect) | P (subgroup difference) |
| --- | --- | --- | --- | --- | --- |
| urinary tract infection | ≥90 | 1.12 | 0.92, 1.36 | 0.25 |  |
|  | 60-89 | 1.03 | 0.94, 1.13 | 0.53 |  |
|  | 45-59 | 0.92 | 0.61, 1.38 | 0.68 |  |
|  | ＜45 | 1.05 | 0.94, 1.19 | 0.38 |  |
|  | total | 1.05 | 0.98, 1.12 | 0.20 | 0.80 |
| genital tract infection | ≥90 | 3.27 | 2.47, 4.34 | ＜0.001 |  |
|  | 60-89 | 3.50 | 2.88, 4.25 | ＜0.001 |  |
|  | 45-59 | 3.24 | 1.24, 8.46 | 0.02 |  |
|  | ＜45 | 2.83 | 2.04, 3.93 | ＜0.001 |  |
|  | total | 3.32 | 2.88, 3.82 | ＜0.001 | 0.75 |
| amputation | ≥90 | 1.48 | 0.06, 36.49 | 0.81 |  |
|  | 60-89 | 1.19 | 0.68, 2.09 | 0.54 |  |
|  | 45-59 | 3.08 | 0.12, 76.30 | 0.49 |  |
|  | ＜45 | 0.93 | 0.66, 1.30 | 0.67 |  |
|  | total | 1.01 | 0.76, 1.34 | 0.96 | 0.78 |
| hypovolemia | ≥90 | 2.15 | 1.27, 3.64 | 0.005 |  |
|  | 60-89 | 1.10 | 0.98, 1.25 | 0.11 |  |
|  | 45-59 | 1.65 | 0.82, 3.30 | 0.16 |  |
|  | ＜45 | 1.37 | 1.18, 1.60 | ＜0.001 |  |
|  | total | 1.24 | 1.13, 1.35 | ＜0.001 | 0.02 |
| orthostatic hypotension | ≥90 | 2.77 | 0.30, 25.75 | 0.37 |  |
|  | 60-89 | 1.28 | 0.52, 3.13 | 0.59 |  |
|  | 45-59 | NA | NA | NA |  |
|  | ＜45 | 1.52 | 0.06, 37.71 | 0.80 |  |
|  | total | 1.45 | 0.65, 3.23 | 0.36 | 0.82 |
| bone fracture | ≥90 | 0.62 | 0.30, 1.26 | 0.19 |  |
|  | 60-89 | 0.97 | 0.81, 1.16 | 0.72 |  |
|  | 45-59 | 0.36 | 0.11, 1.14 | 0.08 |  |
|  | ＜45 | 1.13 | 0.75, 1.71 | 0.54 |  |
|  | total | 0.98 | 0.86, 1.12 | 0.78 | 0.18 |
| DKA | ≥90 | 6.20 | 2.27, 16.91 | ＜0.001 |  |
|  | 60-89 | 2.44 | 1.26, 4.72 | 0.008 |  |
|  | 45-59 | 3.08 | 0.12, 76.30 | 0.49 |  |
|  | ＜45 | 1.00 | 0.33, 3.02 | 1.00 |  |
|  | total | 2.14 | 1.27, 3.61 | 0.004 | 0.12 |
| hypoglycemia | ≥90 | 1.57 | 1.29, 1.91 | ＜0.001 |  |
|  | 60-89 | 1.07 | 0.98, 1.16 | 0.14 |  |
|  | 45-59 | 0.94 | 0.74, 1.19 | 0.60 |  |
|  | ＜45 | 0.80 | 0.62, 1.05 | 0.11 |  |
|  | total | 1.09 | 1.02, 1.17 | 0.02 | ＜0.001 |

AEs, adverse effect; RCTs, randomized controlled trials; OR, odds ratio; DKA, diabetic ketoacidosis.

Table S7. Efficacy of SGLT2i treatment in patients with different levels of renal function in placebo-controlled RCTs.

| Efficacy outcomes | Renal function | Mean difference | 95%CI | P (over effect) | P (subgroup difference) |
| --- | --- | --- | --- | --- | --- |
| HbA1c (%) | ≥90 | -0.58 | -0.67, -0.50 | ＜0.001 |  |
|  | 60-89 | -0.67 | -0.74, -0.60 | ＜0.001 |  |
|  | 45-59 | -0.22 | -0.35, -0.09 | ＜0.001 |  |
|  | ＜45 | -0.13 | -0.67, 0.42 | 0.65 |  |
|  | total | -0.61 | -0.67, -0.56 | ＜0.001 | ＜0.001 |
| Weight (kg) | ≥90 | -2.07 | -2.31, -1.83 | ＜0.001 |  |
|  | 60-89 | -1.81 | -1.98, -1.64 | ＜0.001 |  |
|  | 45-59 | -1.23 | -1.59, -0.86 | ＜0.001 |  |
|  | ＜45 | -1.88 | -3.04, -0.72 | 0.001 |  |
|  | total | -1.85 | -1.99, -1.70 | ＜0.001 | 0.002 |
| SBP (mmHg) | ≥90 | -3.62 | -4.09, -3.15 | ＜0.001 |  |
|  | 60-89 | -3.67 | -4.16, -3.17 | ＜0.001 |  |
|  | 45-59 | -3.58 | -4.67, -2.50 | ＜0.001 |  |
|  | ＜45 | -5.36 | -7.98, -2.74 | ＜0.001 |  |
|  | total | -3.64 | -3.99, -3.30 | ＜0.001 | 0.65 |
| DBP (mmHg) | ≥90 | -1.45 | -1.75, -1.14 | ＜0.001 |  |
|  | 60-89 | -1.58 | -1.79, -1.36 | ＜0.001 |  |
|  | 45-59 | -1.98 | -2.69, -1.26 | ＜0.001 |  |
|  | ＜45 | -0.83 | -1.08, -0.58 | ＜0.001 |  |
|  | total | -1.32 | -1.46, -1.18 | ＜0.001 | ＜0.001 |
| eGFR (mL/min/1.73 m^2^) | ≥90 | -0.45 | -1.39,0.50 | 0.35 |  |
|  | 60-89 | -0.54 | -1.00, -0.09 | 0.02 |  |
|  | 45-59 | -0.68 | -3.80, 2.45 | 0.67 |  |
|  | ＜45 | -0.21 | -1.77, -1.34 | 0.79 |  |
|  | total | -0.54 | -0.94, -0.14 | 0.008 | 0.98 |

HbA1c, glycosylated hemoglobin; SBP, systolic blood pressure; DBP, diastolic blood pressure; eGFR, estimated glomerular filtration rate.

Table S8. Efficacy of SGLT2i treatment in patients with different levels of renal function (follow-up period ≥12 weeks)

| Efficacy outcomes | Renal function | Mean difference | 95%CI | P (over effect) | P(subgroup difference) |
| --- | --- | --- | --- | --- | --- |
| HbA1c (%) | ≥90 | -0.49 | -0.59,-0.39 | ＜0.001 |  |
|  | 60-89 | -0.58 | -0.66,-0.5 | ＜0.001 |  |
|  | 45-59 | -0.22 | -0.35,-0.09 | ＜0.001 |  |
|  | ＜45 | -0.13 | -0.67,0.42 | 0.65 |  |
|  | total | -0.53 | -0.59,-0.47 | ＜0.001 | ＜0.001 |
| Weight (kg) | ≥90 | -2.13 | -2.66,-1.59 | ＜0.001 |  |
|  | 60-89 | -2.06 | -2.31,-1.82 | ＜0.001 |  |
|  | 45-59 | -1.23 | -1.59,-0.86 | ＜0.001 |  |
|  | ＜45 | -1.88 | -3.04,-0.72 | 0.001 |  |
|  | total | -2.05 | -2.32,-1.79 | ＜0.001 | 0.002 |
| SBP (mmHg) | ≥90 | -3.83 | -4.57,-3.09 | ＜0.001 |  |
|  | 60-89 | -3.79 | -4.24,-3.34 | ＜0.001 |  |
|  | 45-59 | -3.58 | -4.67,-2.5 | ＜0.001 |  |
|  | ＜45 | -5.36 | -7.98,-2.74 | ＜0.001 |  |
|  | total | -3.86 | -4.29,-3.42 | ＜0.001 | 0.68 |
| DBP (mmHg) | ≥90 | -1.72 | -1.9,-1.55 | ＜0.001 |  |
|  | 60-89 | -1.63 | -1.83,-1.43 | ＜0.001 |  |
|  | 45-59 | -1.98 | -2.69,-1.26 | ＜0.001 |  |
|  | ＜45 | -0.83 | -1.08,-0.58 | ＜0.001 |  |
|  | total | -1.5 | -1.62,-1.39 | ＜0.001 | ＜0.001 |
| eGFR (mL/min/1.73 m^2^) | ≥90 | -0.43 | -1.4,0.54 | 0.38 |  |
|  | 60-89 | -0.46 | -0.93,0 | 0.05 |  |
|  | 45-59 | -0.68 | -3.8,2.45 | 0.67 |  |
|  | ＜45 | -0.21 | -1.77,1.34 | 0.79 |  |
|  | total | -0.48 | -0.87,-0.08 | 0.02 | 0.99 |

HbA1c, glycosylated hemoglobin; SBP, systolic blood pressure; DBP, diastolic blood pressure; eGFR, estimated glomerular filtration rate.

**Reference**

1. Cusi K, Bril F, Barb D, et al. Effect of canagliflozin treatment on hepatic triglyceride content and glucose metabolism in patients with type 2 diabetes[J]. Diabetes Obes Metab, 2019, 21(4): 812-821.DOI:10.1111/dom.13584.
2. Ji L, Han P, Liu Y, et al. Canagliflozin in Asian patients with type 2 diabetes on metformin alone or metformin in combination with sulphonylurea[J]. Diabetes Obes Metab, 2015, 17(1): 23-31.DOI:10.1111/dom.12385.
3. Lavalle-González FJ, Januszewicz A, Davidson J, et al. Efficacy and safety of canagliflozin compared with placebo and sitagliptin in patients with type 2 diabetes on background metformin monotherapy: a randomised trial[J]. Diabetologia, 2013, 56(12): 2582-2592.
4. Wilding JP, Charpentier G, Hollander P, et al. Efficacy and safety of canagliflozin in patients with type 2 diabetes mellitus inadequately controlled with metformin and sulphonylurea: a randomised trial[J]. Int J Clin Pract, 2013, 67(12): 1267-1282.
5. Henry RR, Strange P, Zhou R, et al. Effects of Dapagliflozin on 24-Hour Glycemic Control in Patients with Type 2 Diabetes: A Randomized Controlled Trial[J]. Diabetes Technol Ther, 2018, 20(11): 715-724
6. Jabbour SA, Frías JP, Hardy E, et al. Safety and Efficacy of Exenatide Once Weekly Plus Dapagliflozin Once Daily Versus Exenatide or Dapagliflozin Alone in Patients With Type 2 Diabetes Inadequately Controlled With Metformin Monotherapy: 52-Week Results of the DURATION-8 Randomized Controlled Trial[J]. Diabetes Care, 2018, 41(10): 2136-2146
7. Ji L, Ma J, Li H, et al. Dapagliflozin as monotherapy in drug-naive Asian patients with type 2 diabetes mellitus: a randomized, blinded, prospective phase III study[J]. Clin Ther, 2014, 36(1): 84-100 e109.DOI:10.1016/j.clinthera.2013.11.002.
8. Mathieu C, Ranetti AE, Li D, et al. Randomized, Double-Blind, Phase 3 Trial of Triple Therapy With Dapagliflozin Add-on to Saxagliptin Plus Metformin in Type 2 Diabetes[J]. Diabetes Care, 2015, 38(11): 2009-2017.DOI:10.2337/dc15-0779.
9. Rosenstock J, Hansen L, Zee P, et al. Dual add-on therapy in type 2 diabetes poorly controlled with metformin monotherapy: a randomized double-blind trial of saxagliptin plus dapagliflozin addition versus single addition of saxagliptin or dapagliflozin to metformin[J]. Diabetes Care, 2015, 38(3): 376-383.DOI:10.2337/dc14-1142.
10. Wilding JP, Woo V, Soler NG, et al. Long-term efficacy of dapagliflozin in patients with type 2 diabetes mellitus receiving high doses of insulin: a randomized trial[J]. Ann Intern Med, 2012, 156(6): 405-415.DOI:10.7326/0003-4819-156-6-201203200-00003.
11. Ferdinand KC, Izzo JL, Lee J, et al. Antihyperglycemic and Blood Pressure Effects of Empagliflozin in Black Patients With Type 2 Diabetes Mellitus and Hypertension[J]. Circulation, 2019, 139(18): 2098-2109.DOI:10.1161/circulationaha.118.036568..
12. Hadjadj S, Rosenstock J, Meinicke T, et al. Initial Combination of Empagliflozin and Metformin in Patients With Type 2 Diabetes[J]. Diabetes Care, 2016, 39(10): 1718-1728.DOI:10.2337/dc16-0522.
13. Lewin A, DeFronzo RA, Patel S, et al. Initial combination of empagliflozin and linagliptin in subjects with type 2 diabetes[J]. Diabetes Care, 2015, 38(3): 394-402.DOI:10.2337/dc14-2365.
14. Ji L, Liu Y, Miao H, et al. Safety and efficacy of ertugliflozin in Asian patients with type 2 diabetes mellitus inadequately controlled with metformin monotherapy: VERTIS Asia[J]. Diabetes Obes Metab, 2019, 21(6): 1474-1482.
15. Pratley RE, Eldor R, Raji A, et al. Ertugliflozin plus sitagliptin versus either individual agent over 52 weeks in patients with type 2 diabetes mellitus inadequately controlled with metformin: The VERTIS FACTORIAL randomized trial[J]. Diabetes Obes Metab, 2018, 20(5): 1111-1120.
16. Rosenstock J, Frias J, Páll D, et al. Effect of ertugliflozin on glucose control, body weight, blood pressure and bone density in type 2 diabetes mellitus inadequately controlled on metformin monotherapy (VERTIS MET)[J]. Diabetes Obes Metab, 2018, 20(3): 520-529.DOI:10.1111/dom.13103.
17. Inoue H, Morino K, Ugi S, et al. Ipragliflozin, a sodium-glucose cotransporter 2 inhibitor, reduces bodyweight and fat mass, but not muscle mass, in Japanese type 2 diabetes patients treated with insulin: A randomized clinical trial[J]. J Diabetes Investig, 2019, 10(4): 1012-1021.
18. Lu CH, Min KW, Chuang LM, et al. Efficacy, safety, and tolerability of ipragliflozin in Asian patients with type 2 diabetes mellitus and inadequate glycemic control with metformin: Results of a phase 3 randomized, placebo-controlled, double-blind, multicenter trial[J]. J Diabetes Investig, 2016, 7(3): 366-373.
19. Danne T, Cariou B, Banks P, et al. HbA(1c) and Hypoglycemia Reductions at 24 and 52 Weeks With Sotagliflozin in Combination With Insulin in Adults With Type 1 Diabetes: The European inTandem2 Study[J]. Diabetes Care, 2018, 41(9): 1981-1990.DOI:10.2337/dc18-0342.
20. Garg SK, Henry RR, Banks P, et al. Effects of Sotagliflozin Added to Insulin in Patients with Type 1 Diabetes[J]. N Engl J Med, 2017, 377(24): 2337-2348.DOI:10.1056/NEJMoa1708337.
21. Zambrowicz B, Freiman J, Brown PM, et al. LX4211, a dual SGLT1/SGLT2 inhibitor, improved glycemic control in patients with type 2 diabetes in a randomized, placebo-controlled trial[J]. Clin Pharmacol Ther, 2012, 92(2): 158-169.
22. Leiter LA, Yoon KH, Arias P, Langslet G, Xie J, Balis DA, et al. Canagliflozin provides durable glycemic improvements and body weight reduction over 104 weeks versus glimepiride in patients with type 2 diabetes on metformin: a randomized, double-blind, phase 3 study[J]. Diabetes Care, 2015, 38(3): 355-364.DOI:10.2337/dc13-2762.
23. Handelsman Y, Mathieu C, Del Prato S, Johnsson E, Kurlyandskaya R, Iqbal N, et al. Sustained 52-week efficacy and safety of triple therapy with dapagliflozin plus saxagliptin versus dual therapy with sitagliptin added to metformin in patients with uncontrolled type 2 diabetes[J]. Diabetes Obes Metab, 2019, 21(4): 883-892.
24. Jeon HJ, Ku EJ, Oh TK. Dapagliflozin improves blood glucose in diabetes on triple oral hypoglycemic agents having inadequate glucose control[J]. Diabetes Res Clin Pract, 2018, 142: 188-194.DOI:10.1016/j.diabres.2018.05.013.
25. Ferrannini E, Berk A, Hantel S, Pinnetti S, Hach T, Woerle HJ, et al. Long-term safety and efficacy of empagliflozin, sitagliptin, and metformin: an active-controlled, parallel-group, randomized, 78-week open-label extension study in patients with type 2 diabetes[J]. Diabetes Care, 2013, 36(12): 4015-4021.
26. Miller S, Krumins T, Zhou H, Huyck S, Johnson J, Golm G, et al. Ertugliflozin and Sitagliptin Co-initiation in Patients with Type 2 Diabetes: The VERTIS SITA Randomized Study[J]. Diabetes Ther, 2018, 9(1): 253-268.
27. Bode B, Stenlöf K, Harris S, et al. Long-term efficacy and safety of canagliflozin over 104 weeks in patients aged 55-80 years with type 2 diabetes[J]. Diabetes Obes Metab, 2015, 17(3): 294-303.DOI:10.1111/dom.12428.
28. Forst T, Guthrie R, Goldenberg R, et al. Efficacy and safety of canagliflozin over 52 weeks in patients with type 2 diabetes on background metformin and pioglitazone[J]. Diabetes Obes Metab, 2014, 16(5): 467-477.
29. Inagaki N, Kondo K, Yoshinari T, et al. Efficacy and safety of canagliflozin in Japanese patients with type 2 diabetes: a randomized, double-blind, placebo-controlled, 12-week study[J]. Diabetes Obes Metab, 2013, 15(12): 1136-1145.
30. Inagaki N, Kondo K, Yoshinari T, et al. Efficacy and safety of canagliflozin monotherapy in Japanese patients with type 2 diabetes inadequately controlled with diet and exercise: a 24-week, randomized, double-blind, placebo-controlled, Phase III study[J]. Expert Opin Pharmacother, 2014, 15(11): 1501-1515.DOI:10.1517/14656566.2014.935764.
31. Inagaki N, Harashima S, Maruyama N, et al. Efficacy and safety of canagliflozin in combination with insulin: a double-blind, randomized, placebo-controlled study in Japanese patients with type 2 diabetes mellitus[J]. Cardiovasc Diabetol, 2016, 15: 89.
32. Kadowaki T, Inagaki N, Kondo K, et al. Efficacy and safety of canagliflozin as add-on therapy to teneligliptin in Japanese patients with type 2 diabetes mellitus: Results of a 24-week, randomized, double-blind, placebo-controlled trial[J]. Diabetes Obes Metab, 2017, 19(6): 874-882.
33. Rosenstock J, Chuck L, González-Ortiz M, et al. Initial Combination Therapy With Canagliflozin Plus Metformin Versus Each Component as Monotherapy for Drug-Naïve Type 2 Diabetes[J]. Diabetes Care, 2016, 39(3): 353-362.DOI:10.2337/dc15-1736.
34. Stenlöf K, Cefalu WT, Kim KA, et al. Efficacy and safety of canagliflozin monotherapy in subjects with type 2 diabetes mellitus inadequately controlled with diet and exercise[J]. Diabetes Obes Metab, 2013, 15(4): 372-382.
35. Araki E, Onishi Y, Asano M, et al. Efficacy and safety of dapagliflozin over 1 year as add-on to insulin therapy in Japanese patients with type 2 diabetes: the DAISY (Dapagliflozin Added to patients under InSulin therapY) trial[J]. Diabetes Obes Metab, 2017, 19(4): 562-570.DOI:10.1111/dom.12853.
36. Bailey CJ, Morales Villegas EC, Woo V, et al. Efficacy and safety of dapagliflozin monotherapy in people with Type 2 diabetes: a randomized double-blind placebo-controlled 102-week trial[J]. Diabet Med, 2015, 32(4): 531-541.DOI:10.1111/dme.12624.
37. Bolinder J, Ljunggren Ö, Kullberg J, et al. Effects of dapagliflozin on body weight, total fat mass, and regional adipose tissue distribution in patients with type 2 diabetes mellitus with inadequate glycemic control on metformin[J]. J Clin Endocrinol Metab, 2012, 97(3): 1020-1031.DOI:10.1210/jc.2011-2260.
38. McMurray JJV, Solomon SD, Inzucchi SE, et al. Dapagliflozin in Patients with Heart Failure and Reduced Ejection Fraction[J]. N Engl J Med, 2019, 381(21): 1995-2008.DOI:10.1056/NEJMoa1911303.
39. Schumm-Draeger PM, Burgess L, Korányi L, et al. Twice-daily dapagliflozin co-administered with metformin in type 2 diabetes: a 16-week randomized, placebo-controlled clinical trial[J]. Diabetes Obes Metab, 2015, 17(1): 42-51.DOI:10.1111/dom.12387.
40. Strojek K, Yoon KH, Hruba V, et al. Effect of dapagliflozin in patients with type 2 diabetes who have inadequate glycaemic control with glimepiride: a randomized, 24-week, double-blind, placebo-controlled trial[J]. Diabetes Obes Metab, 2011, 13(10): 928-938.DOI:10.1111/j.1463-1326.2011.01434.x.
41. Weber MA, Mansfield TA, Cain VA, et al. Blood pressure and glycaemic effects of dapagliflozin versus placebo in patients with type 2 diabetes on combination antihypertensive therapy: a randomised, double-blind, placebo-controlled, phase 3 study[J]. Lancet Diabetes Endocrinol, 2016, 4(3): 211-220.DOI:10.1016/s2213-8587(15)00417-9.
42. Wilding JP, Norwood P, T'Joen C, et al. A study of dapagliflozin in patients with type 2 diabetes receiving high doses of insulin plus insulin sensitizers: applicability of a novel insulin-independent treatment[J]. Diabetes Care, 2009, 32(9): 1656-1662.
43. Yang W, Ma J, Li Y, et al. Dapagliflozin as add-on therapy in Asian patients with type 2 diabetes inadequately controlled on insulin with or without oral antihyperglycemic drugs: A randomized controlled trial[J]. J Diabetes, 2018, 10(7): 589-599.DOI:10.1111/1753-0407.12634.
44. Barnett AH, Mithal A, Manassie J, et al. Efficacy and safety of empagliflozin added to existing antidiabetes treatment in patients with type 2 diabetes and chronic kidney disease: a randomised, double-blind, placebo-controlled trial[J]. Lancet Diabetes Endocrinol, 2014, 2(5): 369-384.DOI:10.1016/s2213-8587(13)70208-0.
45. Häring HU, Merker L, Seewaldt-Becker E, et al. Empagliflozin as add-on to metformin plus sulfonylurea in patients with type 2 diabetes: a 24-week, randomized, double-blind, placebo-controlled trial[J]. Diabetes Care, 2013, 36(11): 3396-3404.
46. Häring HU, Merker L, Seewaldt-Becker E, et al. Empagliflozin as add-on to metformin in patients with type 2 diabetes: a 24-week, randomized, double-blind, placebo-controlled trial[J]. Diabetes Care, 2014, 37(6): 1650-1659.DOI:10.2337/dc13-2105.
47. Hattori S. Anti-inflammatory effects of empagliflozin in patients with type 2 diabetes and insulin resistance[J]. Diabetol Metab Syndr, 2018, 10: 93.
48. Kario K, Okada K, Kato M, et al. 24-Hour Blood Pressure-Lowering Effect of an SGLT-2 Inhibitor in Patients with Diabetes and Uncontrolled Nocturnal Hypertension: Results from the Randomized, Placebo-Controlled SACRA Study[J]. Circulation, 2018, 139(18): 2089-2097.
49. Kadowaki T, Haneda M, Inagaki N, et al. Empagliflozin monotherapy in Japanese patients with type 2 diabetes mellitus: a randomized, 12-week, double-blind, placebo-controlled, phase II trial[J]. Adv Ther, 2014, 31(6): 621-638.DOI:10.1007/s12325-014-0126-8.
50. Kawamori R, Haneda M, Suzaki K, et al. Empagliflozin as add-on to linagliptin in a fixed-dose combination in Japanese patients with type 2 diabetes: Glycaemic efficacy and safety profile in a 52-week, randomized, placebo-controlled trial[J]. Diabetes Obes Metab, 2018, 20(9): 2200-2209.
51. Kovacs CS, Seshiah V, Swallow R, et al. Empagliflozin improves glycaemic and weight control as add-on therapy to pioglitazone or pioglitazone plus metformin in patients with type 2 diabetes: a 24-week, randomized, placebo-controlled trial[J]. Diabetes Obes Metab, 2014, 16(2): 147-158.DOI:10.1111/dom.12188.
52. Packer M, Anker SD, Butler J, et al. Cardiovascular and Renal Outcomes with Empagliflozin in Heart Failure[J]. N Engl J Med, 2020, 383(15): 1413-1424.DOI:10.1056/NEJMoa2022190.
53. Roden M, Weng J, Eilbracht J, et al. Empagliflozin monotherapy with sitagliptin as an active comparator in patients with type 2 diabetes: a randomised, double-blind, placebo-controlled, phase 3 trial[J]. Lancet Diabetes Endocrinol, 2013, 1(3): 208-219.DOI:10.1016/s2213-8587(13)70084-6.
54. Rosenstock J, Jelaska A, Frappin G, et al. Improved glucose control with weight loss, lower insulin doses, and no increased hypoglycemia with empagliflozin added to titrated multiple daily injections of insulin in obese inadequately controlled type 2 diabetes[J]. Diabetes Care, 2014, 37(7): 1815-1823.DOI:10.2337/dc13-3055.
55. Rosenstock J, Jelaska A, Zeller C, et al. Impact of empagliflozin added on to basal insulin in type 2 diabetes inadequately controlled on basal insulin: a 78-week randomized, double-blind, placebo-controlled trial[J]. Diabetes Obes Metab, 2015, 17(10): 936-948.
56. Ross S, Thamer C, Cescutti J, et al. Efficacy and safety of empagliflozin twice daily versus once daily in patients with type 2 diabetes inadequately controlled on metformin: a 16-week, randomized, placebo-controlled trial[J]. Diabetes Obes Metab, 2015, 17(7): 699-702.DOI:10.1111/dom.12469.
57. Dagogo-Jack S, Liu J, Eldor R, et al. Efficacy and safety of the addition of ertugliflozin in patients with type 2 diabetes mellitus inadequately controlled with metformin and sitagliptin: The VERTIS SITA2 placebo-controlled randomized study[J]. Diabetes Obes Metab, 2018, 20(3): 530-540.
58. Terra SG, Focht K, Davies M, et al. Phase III, efficacy and safety study of ertugliflozin monotherapy in people with type 2 diabetes mellitus inadequately controlled with diet and exercise alone[J]. Diabetes Obes Metab, 2017, 19(5): 721-728.DOI:10.1111/dom.12888.
59. Bando Y, Tohyama H, Aoki K, et al. Ipragliflozin lowers small, dense low-density lipoprotein cholesterol levels in Japanese patients with type 2 diabetes mellitus[J]. J Clin Transl Endocrinol, 2016, 6: 1-7.
60. Han KA, Chon S, Chung CH, et al. Efficacy and safety of ipragliflozin as an add-on therapy to sitagliptin and metformin in Korean patients with inadequately controlled type 2 diabetes mellitus: A randomized controlled trial[J]. Diabetes Obes Metab, 2018, 20(10): 2408-2415.
61. Ishihara H, Yamaguchi S, Nakao I, et al. Efficacy and safety of ipragliflozin as add-on therapy to insulin in Japanese patients with type 2 diabetes mellitus (IOLITE): a multi-centre, randomized, placebo-controlled, double-blind study[J]. Diabetes Obes Metab, 2016, 18(12): 1207-1216.
62. Kashiwagi A, Takahashi H, Ishikawa H, et al. A randomized, double-blind, placebo-controlled study on long-term efficacy and safety of ipragliflozin treatment in patients with type 2 diabetes mellitus and renal impairment: results of the long-term ASP1941 safety evaluation in patients with type 2 diabetes with renal impairment (LANTERN) study[J]. Diabetes Obes Metab, 2015, 17(2): 152-160.
63. Seino Y, Sasaki T, Fukatsu A, et al. Efficacy and safety of luseogliflozin added to insulin therapy in Japanese patients with type 2 diabetes: a multicenter, 52-week, clinical study with a 16-week, double-blind period and a 36-week, open-label period[J]. Curr Med Res Opin, 2018, 34(6): 981-994.DOI:10.1080/03007995.2018.1441816.
64. Buse JB, Garg SK, Rosenstock J, et al. Sotagliflozin in Combination With Optimized Insulin Therapy in Adults With Type 1 Diabetes: The North American inTandem1 Study[J]. Diabetes Care, 2018, 41(9): 1970-1980.
65. Kaku K, Watada H, Iwamoto Y, et al. Efficacy and safety of monotherapy with the novel sodium/glucose cotransporter-2 inhibitor tofogliflozin in Japanese patients with type 2 diabetes mellitus: a combined Phase 2 and 3 randomized, placebo-controlled, double-blind, parallel-group comparative study[J]. Cardiovasc Diabetol, 2014, 13: 65.
66. Terauchi Y, Tamura M, Senda M, et al. Efficacy and safety of tofogliflozin in Japanese patients with type 2 diabetes mellitus with inadequate glycaemic control on insulin therapy (J-STEP/INS): Results of a 16-week randomized, double-blind, placebo-controlled multicentre trial[J]. Diabetes Obes Metab, 2017, 19(10): 1397-1407.
67. Schernthaner G, Gross JL, Rosenstock J, Guarisco M, Fu M, Yee J, et al. Canagliflozin compared with sitagliptin for patients with type 2 diabetes who do not have adequate glycemic control with metformin plus sulfonylurea: a 52-week randomized trial[J]. Diabetes Care, 2013, 36(9): 2508-2515
68. Nauck MA, Del Prato S, Meier JJ, Durán-García S, Rohwedder K, Elze M, et al. Dapagliflozin versus glipizide as add-on therapy in patients with type 2 diabetes who have inadequate glycemic control with metformin: a randomized, 52-week, double-blind, active-controlled noninferiority trial[J]. Diabetes Care, 2011, 34(9): 2015-2022
69. Müller-Wieland D, Kellerer M, Cypryk K, Skripova D, Rohwedder K, Johnsson E, et al. Efficacy and safety of dapagliflozin or dapagliflozin plus saxagliptin versus glimepiride as add-on to metformin in patients with type 2 diabetes[J]. Diabetes Obes Metab, 2018, 20(11): 2598-2607
70. Cho KY, Nakamura A, Omori K, Takase T, Miya A, Manda N, et al. Effect of switching from pioglitazone to the sodium glucose co-transporter-2 inhibitor dapagliflozin on body weight and metabolism-related factors in patients with type 2 diabetes mellitus: An open-label, prospective, randomized, parallel-group comparison trial[J]. Diabetes Obes Metab, 2019, 21(3): 710-714
71. Scott R, Morgan J, Zimmer Z, Lam RLH, O'Neill EA, Kaufman KD, et al. A randomized clinical trial of the efficacy and safety of sitagliptin compared with dapagliflozin in patients with type 2 diabetes mellitus and mild renal insufficiency: The CompoSIT-R study[J]. Diabetes Obes Metab, 2018, 20(12): 2876-2884
72. Shimizu M, Suzuki K, Kato K, Jojima T, Iijima T, Murohisa T, et al. Evaluation of the effects of dapagliflozin, a sodium-glucose co-transporter-2 inhibitor, on hepatic steatosis and fibrosis using transient elastography in patients with type 2 diabetes and non-alcoholic fatty liver disease[J]. Diabetes Obes Metab, 2019, 21(2): 285-292.DOI:10.1111/dom.13520.
73. Hayashi T, Fukui T, Nakanishi N, Yamamoto S, Tomoyasu M, Osamura A, et al. Dapagliflozin decreases small dense low-density lipoprotein-cholesterol and increases high-density lipoprotein 2-cholesterol in patients with type 2 diabetes: comparison with sitagliptin[J]. Cardiovasc Diabetol, 2017, 16(1): 8
74. [13] Zinman B, Lachin JM, Inzucchi SE. Empagliflozin, Cardiovascular Outcomes, and Mortality in Type 2 Diabetes[J]. N Engl J Med, 2016, 374(11): 1094.DOI:10.1056/NEJMc1600827.
75. Araki E, Tanizawa Y, Tanaka Y, Taniguchi A, Koiwai K, Kim G, et al. Long-term treatment with empagliflozin as add-on to oral antidiabetes therapy in Japanese patients with type 2 diabetes mellitus[J]. Diabetes Obes Metab, 2015, 17(7): 665-674.DOI:10.1111/dom.12464.
76. Hollander P, Hill J, Johnson J, Wei Jiang Z, Golm G, Huyck S, et al. Results of VERTIS SU extension study: safety and efficacy of ertugliflozin treatment over 104 weeks compared to glimepiride in patients with type 2 diabetes mellitus inadequately controlled on metformin[J]. Curr Med Res Opin, 2019, 35(8): 1335-1343.DOI:10.1080/03007995.2019.1583450.
77. Tsurutani Y, Nakai K, Inoue K, Azuma K, Mukai S, Maruyama S, et al. Comparative study of the effects of ipragliflozin and sitagliptin on multiple metabolic variables in Japanese patients with type 2 diabetes: A multicentre, randomized, prospective, open-label, active-controlled study[J]. Diabetes Obes Metab, 2018, 20(11): 2675-2679.DOI:10.1111/dom.13421.
78. Takashima H, Yoshida Y, Nagura C, et al. Renoprotective effects of canagliflozin, a sodium glucose cotransporter 2 inhibitor, in type 2 diabetes patients with chronic kidney disease: A randomized open-label prospective trial[J]. Diab Vasc Dis Res, 2018, 15(5): 469-472.DOI:10.1177/1479164118782872.
79. Fioretto P, Del Prato S, Buse JB, et al. Efficacy and safety of dapagliflozin in patients with type 2 diabetes and moderate renal impairment (chronic kidney disease stage 3A): The DERIVE Study[J]. Diabetes Obes Metab, 2018, 20(11): 2532-2540.
80. Pollock C, Stefánsson B, Reyner D, et al. Albuminuria-lowering effect of dapagliflozin alone and in combination with saxagliptin and effect of dapagliflozin and saxagliptin on glycaemic control in patients with type 2 diabetes and chronic kidney disease (DELIGHT): a randomised, double-blind, placebo-controlled trial[J]. Lancet Diabetes Endocrinol, 2019, 7(6): 429-441.DOI:10.1016/s2213-8587(19)30086-5.
81. Grunberger G, Camp S, Johnson J, et al. Ertugliflozin in Patients with Stage 3 Chronic Kidney Disease and Type 2 Diabetes Mellitus: The VERTIS RENAL Randomized Study[J]. Diabetes Ther, 2018, 9(1): 49-66.
82. Haneda M, Seino Y, Inagaki N, et al. Influence of Renal Function on the 52-Week Efficacy and Safety of the Sodium Glucose Cotransporter 2 Inhibitor Luseogliflozin in Japanese Patients with Type 2 Diabetes Mellitus[J]. Clin Ther, 2016, 38(1): 66-88 e20.DOI:10.1016/j.clinthera.2015.10.025.
83. Yale JF, Bakris G, Cariou B, et al. Efficacy and safety of canagliflozin in subjects with type 2 diabetes and chronic kidney disease[J]. Diabetes Obes Metab, 2013, 15(5): 463-473.
84. Heerspink HJL, Stefánsson BV, Correa-Rotter R, et al. Dapagliflozin in Patients with Chronic Kidney Disease[J]. N Engl J Med, 2020, 383(15): 1436-1446.DOI:10.1056/NEJMoa2024816.
85. Bhatt DL, Szarek M, Pitt B, et al. Sotagliflozin in Patients with Diabetes and Chronic Kidney Disease[J]. N Engl J Med, 2021, 384(2): 129-139.DOI:10.1056/NEJMoa2030186.
86. Kohan DE, Fioretto P, Tang W, et al. Long-term study of patients with type 2 diabetes and moderate renal impairment shows that dapagliflozin reduces weight and blood pressure but does not improve glycemic control[J]. Kidney Int, 2014, 85(4): 962-971.
