# Supplementary material for: Disparities in efficacy and safety of sodium-glucose cotransporter 2 inhibitor among patients with different extents of renal dysfunction: A systematic review and meta-analysis of randomized controlled trials
Source: Front Pharmacol. 2022 Nov 22;13:1018720. doi: 10.3389/fphar.2022.1018720 (PMC9723253; doi:10.3389/fphar.2022.1018720)
Supplement: Supplementary file 11 [file DataSheet1.PDF]

Supplementary Materials

Supplementary Figure legends

Figure S1. Funnel plots of analyses for efficacy and safety of SGLT2is.

Figure S2. HbA1c changes before and after SGLT2i treatment in patients with different levels of renal function

Figure S3. Weight changes before and after SGLT2i treatment in patients with different levels of renal function

Figure S4. SBP changes before and after SGLT2i treatment in patients with different levels of renal function

Figure S5. DBP changes before and after SGLT2i treatment in patients with different levels of renal function

Figure S6. eGFR changes before and after SGLT2i treatment in patients with different levels of renal function

Figure S7. The associations between the use of SGLT2i treatment and urinary tract infection

Figure S8. The associations between the use of SGLT2i treatment and genital tract infection

Figure S9. The associations between the use of SGLT2i treatment and amputation

Figure S10. The associations between the use of SGLT2i treatment and hypovolemia

Figure S11. The associations between the use of SGLT2i treatment and orthostatic hypotension

Figure S12. The associations between the use of SGLT2i treatment and bone fracture

Figure S13. The associations between the use of SGLT2i treatment and diabetic ketoacidosis

Figure S14. The associations between the use of SGLT2i treatment and hypoglycemia

Supplementary Table legend

Table S1. Baseline characteristics of included randomized controlled trials.

Table S2. Evaluation for risk of bias in included RCTs.

Table S3. Subgroup analyses of efficacy among different drug categories.

Table S4. Subgroup analyses of efficacy among follow-up period.

Table S5. Subgroup analyses of different AEs.

Table S6. Subgroup analyses of different AEs in placebo-controlled RCTs.

Table S7. Efficacy of SGLT2i treatment in patients with different levels of renal function in placebo-controlled RCTs.

Table S8. Efficacy of SGLT2i treatment in patients with different levels of renal function (follow-up period ⩾12 weeks).

Figure S1. Funnel plots of analyses for efficacy and safety of SGLT2is.

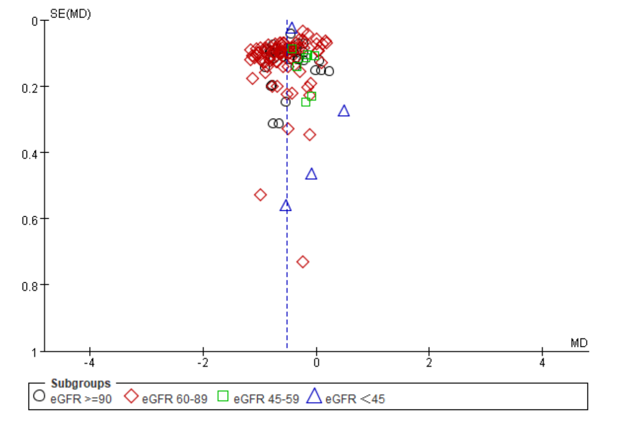

(a) HbA1c changes

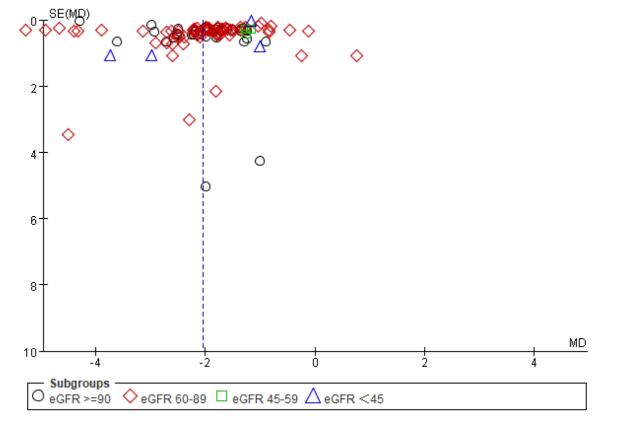

(b) Weight changes

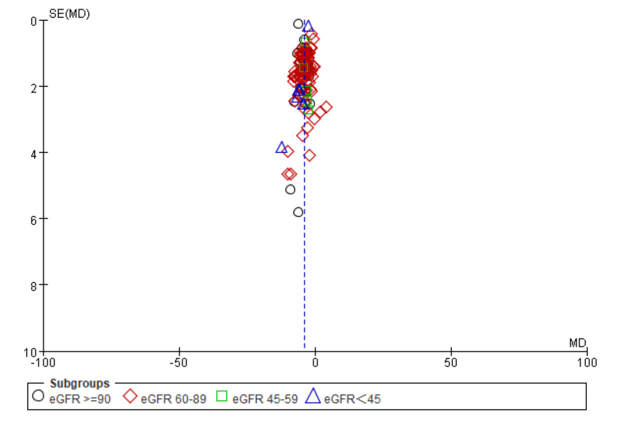

(c) SBP changes

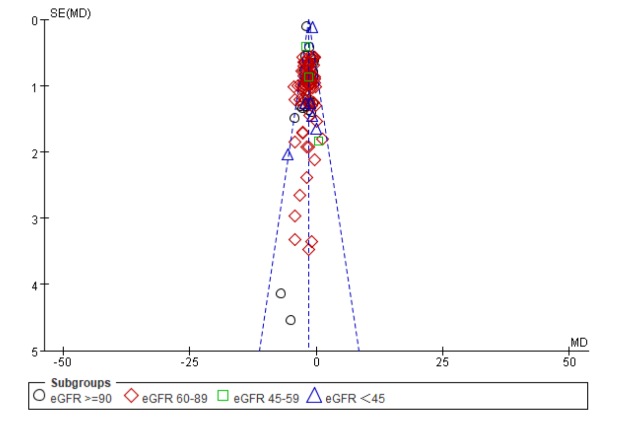

(d) DBP changes

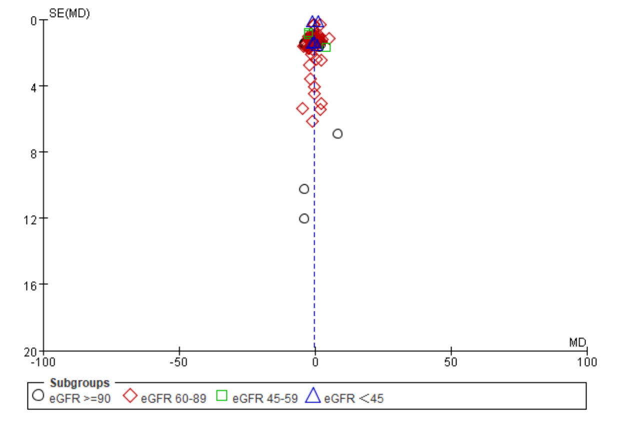

(e) eGFR changes

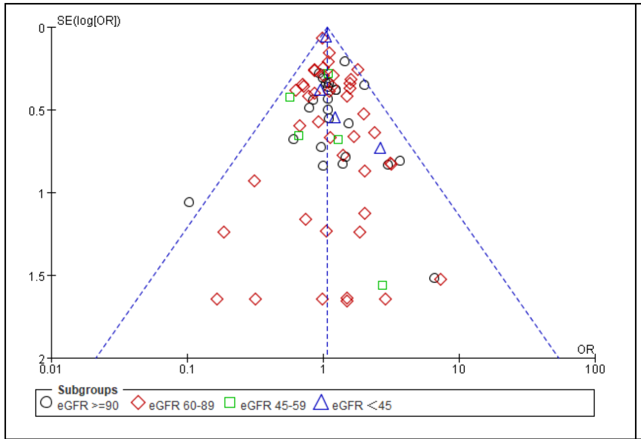

(f) urinary tract infection

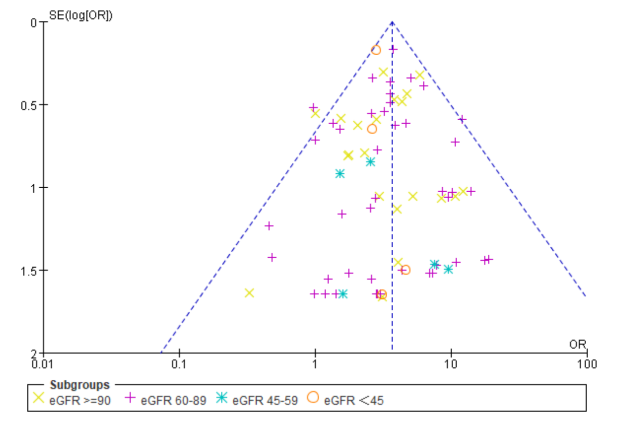

(g) genital tract infection

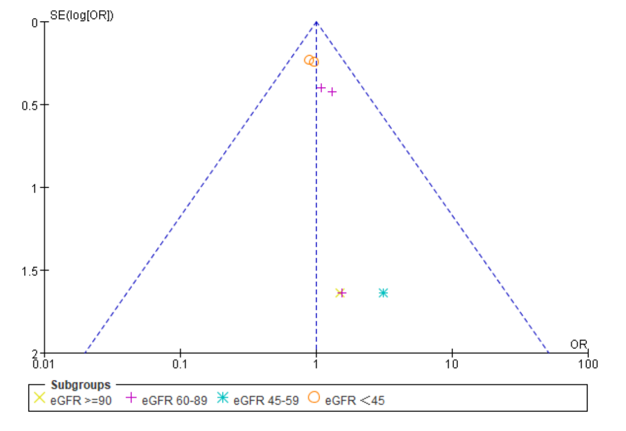

(h) amputation

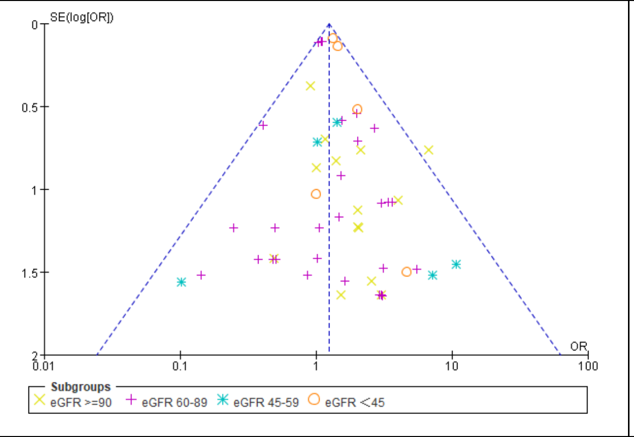

(i) hypovolemia

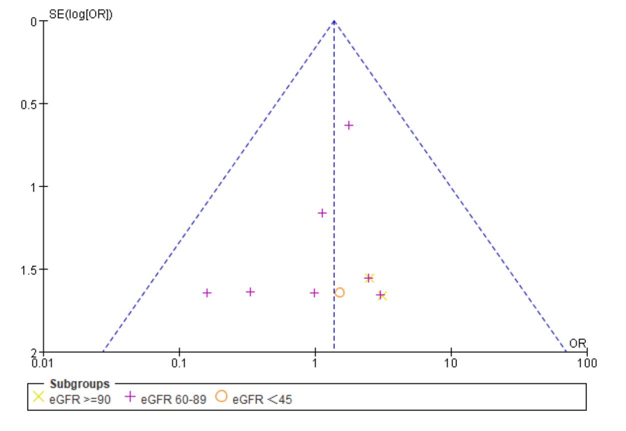

(j) orthostatic hypotension

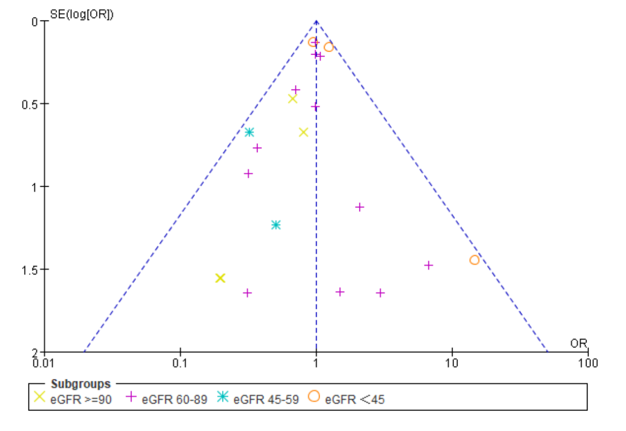

(k) bone fracture

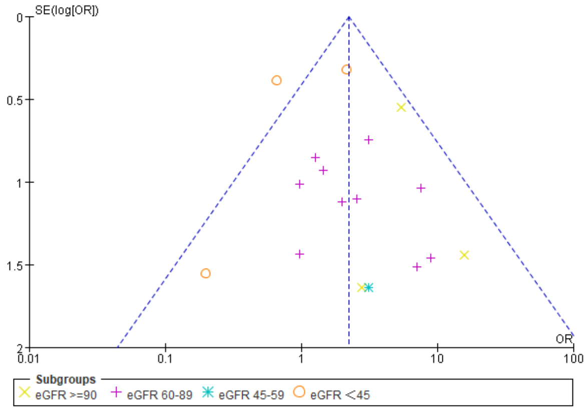

(l) diabetic ketoacidosis(DKA)

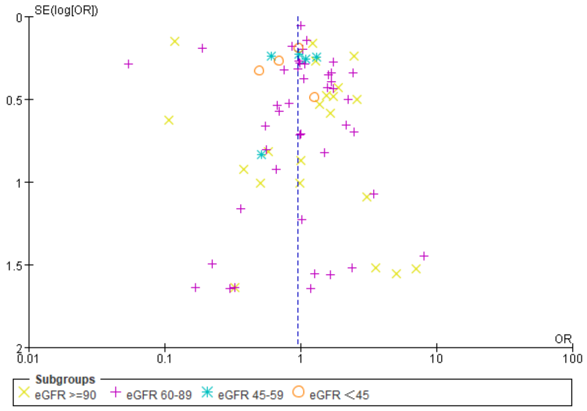

(m) hypoglycemia

Figure S2. HbA1c changes before and after SGLT2i treatment in patients with different levels of renal function.

Study or Subgroup	SGLT2i Mean	SGLT2i SD	SGLT2i Total	control Mean	control SD	control Total	Weight	Mean Difference IV, Random, 95% CI
**8.1.1 eGFR >=90**								
CANA Cusi 2018	-0.7	0.65	26	0.1	0.838	30	0.7%	-0.80 [-1.19, -0.41]
CANA Lavalle-González 2013-2	-0.94	0.843	367	-0.17	0.812	183	0.9%	-0.77 [-0.92, -0.62]
DAPA Handelsman 2018	-1.41	1.06	232	-1.07	1.088	229	0.8%	-0.34 [-0.54, -0.14]
DAPA Henry 2018	-0.51	0.495	50	-0.28	0.495	50	0.8%	-0.23 [-0.42, -0.04]
DAPA Jabbour 2018	-1.75	1.51	228	-1.38	1.507	227	0.8%	-0.37 [-0.65, -0.09]
DAPA Jeon 2018	-0.97	1.29	162	-0.95	1.41	148	0.7%	-0.02 [-0.32, 0.28]
DAPA Ji 2014-1	-1.04	0.808	128	-0.29	0.791	132	0.8%	-0.75 [-0.94, -0.56]
DAPA Ji 2014-2	-1.11	0.765	133	-0.29	0.791	132	0.8%	-0.82 [-1.01, -0.63]
DAPA Mathieu 2015	-0.82	0.871	160	-0.1	0.904	160	0.8%	-0.72 [-0.91, -0.53]
DAPA Rosenstock 2015	-1.47	1.058	179	-0.88	1.049	176	0.8%	-0.59 [-0.81, -0.37]
EMPA Ferdinand 2019	-0.71	1.236	78	0.07	1.188	72	0.7%	-0.78 [-1.17, -0.39]
EMPA Ferrannini 2013-1-1	-0.34	0.913	80	-0.56	0.878	56	0.7%	0.22 [-0.08, 0.52]
EMPA Ferrannini 2013-1-2	-0.47	0.933	88	-0.56	0.878	56	0.7%	0.09 [-0.21, 0.39]
EMPA Ferrannini 2013-2-1	-0.34	0.776	137	-0.4	0.764	56	0.8%	0.06 [-0.18, 0.30]
EMPA Ferrannini 2013-2-2	-0.63	0.782	139	-0.4	0.764	56	0.8%	-0.23 [-0.47, 0.01]
EMPA Hadjadj 2016-1-1	-1.98	0.99	153	-1.18	0.953	142	0.8%	-0.80 [-1.02, -0.58]
EMPA Hadjadj 2016-1-2	-1.93	1.028	149	-1.18	0.953	142	0.8%	-0.75 [-0.98, -0.52]
EMPA Hadjadj 2016-2-1	-2.07	0.983	151	-1.75	1.087	146	0.8%	-0.32 [-0.56, -0.08]
EMPA Hadjadj 2016-2-2	-2.08	1.009	159	-1.75	1.087	146	0.8%	-0.33 [-0.57, -0.09]
ERTU Ji 2019-1	-1	0.9	170	-0.2	0.8	167	0.9%	-0.80 [-0.98, -0.62]
ERTU Ji 2019-2	-0.9	0.8	169	-0.2	0.8	167	0.9%	-0.70 [-0.87, -0.53]
ERTU Miller 2018-1	-1.7	0.9	98	-0.8	1.1	97	0.8%	-0.90 [-1.18, -0.62]
ERTU Pratley 2018-1	-1.5	0.795	243	-1.1	1.203	247	0.9%	-0.40 [-0.58, -0.22]
ERTU Pratley 2018-2	-1.5	0.797	244	-1.1	1.203	247	0.9%	-0.40 [-0.58, -0.22]
ERTU Rosenstock 2018-2	-0.91	0.913	205	-0.03	0.922	209	0.9%	-0.88 [-1.06, -0.70]
IPRA Inoue 2019	-0.69	0.882	24	-0.15	0.833	24	0.6%	-0.54 [-1.03, -0.05]
IPRA Lu 2016	-0.94	0.747	87	-0.47	0.806	83	0.8%	-0.47 [-0.70, -0.24]
SOTA Danne 2018-1	-0.18	0.84	261	0.04	0.835	258	0.9%	-0.22 [-0.36, -0.08]
SOTA Danne 2018-2	-0.28	0.843	263	0.04	0.835	258	0.9%	-0.32 [-0.46, -0.18]
SOTA Garg 2017	-0.79	0.793	699	-0.33	0.795	703	0.9%	-0.46 [-0.54, -0.38]
SOTA Zambrowicz 2012-1	-1.15	0.76	12	-0.49	0.778	12	0.5%	-0.66 [-1.28, -0.04]
SOTA Zambrowicz 2012-2	-1.25	0.76	12	-0.49	0.778	12	0.5%	-0.76 [-1.38, -0.14]
**Subtotal (95% CI)**			**5286**			**4823**	**25.0%**	**-0.49 [-0.58, -0.39]**
Heterogeneity: Tau² = 0.06; Chi² = 205.16, df = 31 (P < 0.00001); I² = 85%								
Test for overall effect: Z = 9.99 (P < 0.00001)								
**8.1.2 eGFR 60-89**								
CANA Forst 2014-1	-0.89	0.733	113	-0.26	0.74	115	0.8%	-0.63 [-0.82, -0.44]
CANA Forst 2014-2	-1.03	0.747	114	-0.26	0.74	115	0.8%	-0.77 [-0.96, -0.58]
CANA Inagaki 2013-1	-0.61	0.543	82	0.11	0.52	75	0.9%	-0.72 [-0.89, -0.55]
CANA Inagaki 2013-2	-0.8	0.516	74	0.11	0.52	75	0.9%	-0.91 [-1.08, -0.74]
CANA Inagaki 2013-3	-0.79	0.523	76	0.11	0.52	75	0.9%	-0.90 [-1.07, -0.73]
CANA Inagaki 2013-4	-0.88	0.52	75	0.11	0.52	75	0.9%	-0.99 [-1.16, -0.82]
CANA Inagaki 2014-1	-0.74	0.664	90	0.29	0.675	93	0.8%	-1.03 [-1.22, -0.84]
CANA Inagaki 2014-2	-0.76	0.657	88	0.29	0.675	93	0.8%	-1.05 [-1.24, -0.86]
CANA Inagaki 2016	-0.97	0.697	76	0.13	0.669	70	0.8%	-1.10 [-1.32, -0.88]
CANA Kadowaki 2017	-0.97	0.837	70	-0.1	0.825	68	0.8%	-0.87 [-1.15, -0.59]
CANA Lavalle-González 2013-1	-0.79	0.844	368	-0.17	0.812	183	0.9%	-0.62 [-0.77, -0.47]
CANA Rosenstock 2016-1	-1.78	1.078	237	-1.3	1.078	237	0.8%	-0.48 [-0.67, -0.29]
CANA Rosenstock 2016-2	-1.77	1.078	237	-1.3	1.078	237	0.8%	-0.47 [-0.66, -0.28]
CANA Schernthaner 2013	-1.03	0.932	377	-0.66	0.953	378	0.9%	-0.37 [-0.50, -0.24]
CANA Stenlof 2013-1	-1.03	0.898	197	0.14	0.901	192	0.9%	-1.17 [-1.35, -0.99]
CANA Stenlof 2013-2	-0.77	0.908	195	0.14	0.901	192	0.9%	-0.91 [-1.09, -0.73]
DAPA Araki 2017	-0.74	0.789	122	-0.83	0.83	60	0.8%	0.09 [-0.16, 0.34]
DAPA Bailey 2015-1	-0.3	1.296	65	-0.17	1.37	75	0.6%	-0.13 [-0.57, 0.31]
DAPA Bailey 2015-2	-0.7	1.265	64	-0.17	1.37	75	0.6%	-0.53 [-0.97, -0.09]
DAPA Bailey 2015-3	-0.61	1.281	70	-0.17	1.37	75	0.6%	-0.44 [-0.87, -0.01]
DAPA Cho 2018	0.1	0.742	36	0.2	0.872	35	0.7%	-0.10 [-0.48, 0.28]
DAPA Hayashi 2017	-0.75	1.264	40	-0.63	1.794	40	0.4%	-0.12 [-0.80, 0.56]
DAPA McMurray 2019	-0.21	1.14	2373	0.04	1.29	2371	0.9%	-0.25 [-0.32, -0.18]
DAPA Müller-Wieland 2018-1	-0.82	0.868	314	-0.99	0.849	313	0.9%	0.17 [0.04, 0.30]
DAPA Müller-Wieland 2018-2	-1.2	0.813	312	-0.99	0.849	313	0.9%	-0.21 [-0.34, -0.08]
DAPA Nauck 2011	-0.52	0.816	400	-0.52	0.817	401	0.9%	0.00 [-0.11, 0.11]
DAPA Schumm-Draeger 2015-1	-0.59	0.595	99	-0.3	0.596	101	0.9%	-0.29 [-0.46, -0.12]
DAPA Schumm-Draeger 2015-2	-0.52	0.594	100	-0.3	0.596	101	0.9%	-0.22 [-0.38, -0.06]
DAPA Schumm-Draeger 2015-3	-0.65	0.597	99	-0.3	0.596	101	0.9%	-0.35 [-0.52, -0.18]
DAPA Scott 2018	-0.36	0.803	306	-0.51	0.76	307	0.9%	0.15 [0.03, 0.27]
DAPA Shimizu 2018	-0.8	1.172	33	-0.3	1.25	24	0.4%	-0.50 [-1.14, 0.14]
DAPA Strojek 2011-1	-0.58	0.728	154	-0.13	0.768	145	0.9%	-0.45 [-0.62, -0.28]
DAPA Strojek 2011-2	-0.63	0.76	142	-0.13	0.768	145	0.9%	-0.50 [-0.68, -0.32]
DAPA Strojek 2011-3	-0.82	0.752	151	-0.13	0.768	145	0.9%	-0.69 [-0.86, -0.52]
DAPA Weber 2016	-0.63	1.002	225	-0.02	1.007	224	0.8%	-0.61 [-0.80, -0.42]
DAPA Wilding 2009-1	-0.61	0.625	24	0.09	0.734	23	0.7%	-0.70 [-1.09, -0.31]
DAPA Wilding 2009-2	-0.69	0.625	24	0.09	0.734	23	0.7%	-0.78 [-1.17, -0.39]
DAPA Yang 2018	-0.87	0.782	139	0.03	0.824	133	0.8%	-0.90 [-1.09, -0.71]
EMPA Araki 2015-1	-0.93	0.583	136	-0.97	0.635	63	0.8%	0.04 [-0.14, 0.22]
EMPA Araki 2015-2	-0.96	0.585	137	-0.97	0.635	63	0.8%	0.01 [-0.17, 0.19]
EMPA Barnett 2014-1	-0.6	0.804	97	0.06	0.572	95	0.8%	-0.66 [-0.86, -0.46]
EMPA Barnett 2014-2	-0.57	0.808	98	0.06	0.572	95	0.8%	-0.63 [-0.83, -0.43]
EMPA Haring 2013-1	-0.82	0.75	225	-0.17	0.75	225	0.9%	-0.65 [-0.79, -0.51]
EMPA Haring 2013-2	-0.77	0.735	216	-0.17	0.75	225	0.9%	-0.60 [-0.74, -0.46]
EMPA Haring 2014-1	-0.7	0.737	217	-0.13	0.719	207	0.9%	-0.57 [-0.71, -0.43]
EMPA Haring 2014-2	-0.77	0.73	213	-0.13	0.719	207	0.9%	-0.64 [-0.78, -0.50]
EMPA Hattori 2018	-0.11	1.159	51	0.04	0.889	51	0.6%	-0.15 [-0.55, 0.25]
EMPA Kadowaki 2014-1	-0.42	0.944	110	0.3	0.94	109	0.8%	-0.72 [-0.97, -0.47]
EMPA Kadowaki 2014-2	-0.4	0.94	109	0.3	0.94	109	0.8%	-0.70 [-0.95, -0.45]
EMPA Kadowaki 2014-3	-0.65	0.94	109	0.3	0.94	109	0.8%	-0.95 [-1.20, -0.70]
EMPA Kadowaki 2014-4	-0.61	0.944	110	0.3	0.94	109	0.8%	-0.91 [-1.16, -0.66]
EMPA Kawamori 2018	-0.93	0.809	182	0.21	0.868	93	0.8%	-1.14 [-1.35, -0.93]
EMPA Kovacs 2014-1	-0.59	0.899	165	-0.11	0.899	165	0.8%	-0.48 [-0.67, -0.29]
EMPA Kovacs 2014-2	-0.72	0.907	168	-0.11	0.899	165	0.8%	-0.61 [-0.80, -0.42]
EMPA Packer 2020	-0.28	1.295	1863	-0.12	1.296	1867	0.9%	-0.16 [-0.24, -0.08]
EMPA Roden 2013-1	-0.66	0.764	224	0.08	0.809	228	0.9%	-0.74 [-0.89, -0.59]
EMPA Roden 2013-2	-0.78	0.802	224	0.08	0.809	228	0.9%	-0.86 [-1.01, -0.71]
EMPA Rosenstock 2014-1	-1.18	1.091	186	-0.81	1.097	188	0.8%	-0.37 [-0.59, -0.15]
EMPA Rosenstock 2014-2	-1.27	1.1	189	-0.81	1.097	188	0.8%	-0.46 [-0.68, -0.24]
EMPA Rosenstock 2015-1	-0.5	1.3	169	0	1.304	170	0.8%	-0.50 [-0.78, -0.22]
EMPA Rosenstock 2015-2	-0.6	1.245	155	0	1.304	170	0.8%	-0.60 [-0.88, -0.32]
EMPA Ross 2015-1	-0.83	0.733	215	-0.22	0.724	107	0.9%	-0.61 [-0.78, -0.44]
EMPA Ross 2015-2	-0.72	0.731	214	-0.22	0.724	107	0.9%	-0.50 [-0.67, -0.33]
EMPA Ross 2015-3	-0.66	0.733	215	-0.22	0.724	107	0.9%	-0.44 [-0.61, -0.27]
EMPA Ross 2015-4	-0.64	0.731	214	-0.22	0.724	107	0.9%	-0.42 [-0.59, -0.25]
ERTU Dagogo-Jack 2018-1	-0.8	0.9	156	-0.3	0.9	153	0.8%	-0.50 [-0.70, -0.30]
ERTU Dagogo-Jack 2018-2	-1	0.8	153	-0.3	0.9	153	0.8%	-0.70 [-0.89, -0.51]
ERTU Hollander 2019-1	-0.3	1.076	445	-0.4	1.064	435	0.9%	0.10 [-0.04, 0.24]
ERTU Hollander 2019-2	-0.4	1.064	435	-0.4	1.064	435	0.9%	0.00 [-0.14, 0.14]
ERTU Miller 2018-2	-1.7	1.1	96	-0.8	1.1	97	0.7%	-0.90 [-1.21, -0.59]
ERTU Rosenstock 2018-1	-0.73	0.881	207	-0.03	0.922	209	0.9%	-0.70 [-0.87, -0.53]
ERTU Terra 2017-1	-0.79	1.02	156	0.2	1.104	153	0.8%	-0.99 [-1.23, -0.75]
ERTU Terra 2017-2	-0.96	1.006	152	0.2	1.104	153	0.8%	-1.16 [-1.40, -0.92]
IPRA Bando 2016	-0.61	0.52	40	0.52	0.74	22	0.7%	-1.13 [-1.48, -0.78]
IPRA Han 2018	-0.79	0.59	73	0.03	0.84	66	0.8%	-0.82 [-1.06, -0.58]
IPRA Ishihara 2016	-0.79	0.66	168	0.27	0.65	87	0.9%	-1.06 [-1.23, -0.89]
IPRA Kashiwagi 2015	-0.42	5.583	118	-0.17	3.5	46	0.1%	-0.25 [-1.68, 1.18]
IPRA Tsurutani 2018	-1.1	0.909	60	-0.81	0.803	59	0.7%	-0.29 [-0.60, 0.02]
LUSEO Seino 2018	-0.74	0.804	159	0.39	0.746	74	0.8%	-1.13 [-1.34, -0.92]
SOTA Buse 2018-1	-0.26	0.714	263	-0.01	0.72	268	0.9%	-0.25 [-0.37, -0.13]
SOTA Buse 2018-2	-0.32	0.712	262	-0.01	0.72	268	0.9%	-0.31 [-0.43, -0.19]
TOFO Kaku 2014-1	-0.797	0.628	57	-0.028	0.628	56	0.8%	-0.77 [-1.00, -0.54]
TOFO Kaku 2014-2	-1.017	3.952	58	-0.028	0.628	56	0.2%	-0.99 [-2.02, 0.04]
TOFO Kaku 2014-3	-0.87	0.626	58	-0.028	0.628	56	0.8%	-0.84 [-1.07, -0.61]
TOFO Terauchi 2017	-0.72	0.71	141	0.35	0.74	70	0.8%	-1.07 [-1.28, -0.86]
**Subtotal (95% CI)**			**17229**			**15976**	**67.9%**	**-0.58 [-0.66, -0.50]**
Heterogeneity: Tau² = 0.12; Chi² = 1281.96, df = 84 (P < 0.00001); I² = 93%								
Test for overall effect: Z = 14.65 (P < 0.00001)								
**8.1.3 eGFR 45-59**								
CANA Takashima 2018	-0.4	0.7	21	-0.2	0.9	21	0.6%	-0.20 [-0.69, 0.29]
DAPA Fioretto 2018	-0.37	1.265	160	-0.03	1.269	161	0.8%	-0.34 [-0.62, -0.06]
DAPA Pollock 2019	-0.41	2.519	145	-0.32	1.148	148	0.6%	-0.09 [-0.54, 0.36]
EMPA Barnett 2014-3	-0.32	0.837	187	0.12	0.837	187	0.9%	-0.44 [-0.61, -0.27]
ERTU Grunberger 2017-1	-0.29	0.962	158	-0.26	0.95	154	0.8%	-0.03 [-0.24, 0.18]
ERTU Grunberger 2017-2	-0.41	0.921	155	-0.26	0.95	154	0.8%	-0.15 [-0.36, 0.06]
LUSEO Haneda 2016	-0.11	0.497	95	0.09	0.722	50	0.8%	-0.20 [-0.42, 0.02]
**Subtotal (95% CI)**			**921**			**875**	**5.2%**	**-0.22 [-0.35, -0.09]**
Heterogeneity: Tau² = 0.01; Chi² = 10.89, df = 6 (P = 0.09); I² = 45%								
Test for overall effect: Z = 3.38 (P = 0.0007)								
**8.1.4 eGFR <45**								
DAPA Kohan 2013-1	-1.21	3.462	83	-0.67	3.758	84	0.2%	-0.54 [-1.64, 0.56]
DAPA Kohan 2013-2	-0.75	2.028	85	-0.67	3.758	84	0.3%	-0.08 [-0.99, 0.83]
EMPA Barnett 2014-4	0.11	1.48	37	-0.37	0.79	37	0.5%	0.48 [-0.06, 1.02]
SOTA Bhatt 2021	-0.6	1.299	5292	-0.17	1.299	5292	0.9%	-0.43 [-0.48, -0.38]
**Subtotal (95% CI)**			**5497**			**5497**	**1.9%**	**-0.13 [-0.67, 0.42]**
Heterogeneity: Tau² = 0.20; Chi² = 11.38, df = 3 (P = 0.010); I² = 74%								
Test for overall effect: Z = 0.46 (P = 0.65)								
**Total (95% CI)**			**28933**			**27171**	**100.0%**	**-0.53 [-0.59, -0.47]**
Heterogeneity: Tau² = 0.09; Chi² = 1543.42, df = 127 (P < 0.00001); I² = 92%								
Test for overall effect: Z = 18.01 (P < 0.00001)								
Test for subgroup differences: Chi² = 22.92, df = 3 (P < 0.0001), I² = 86.9%								

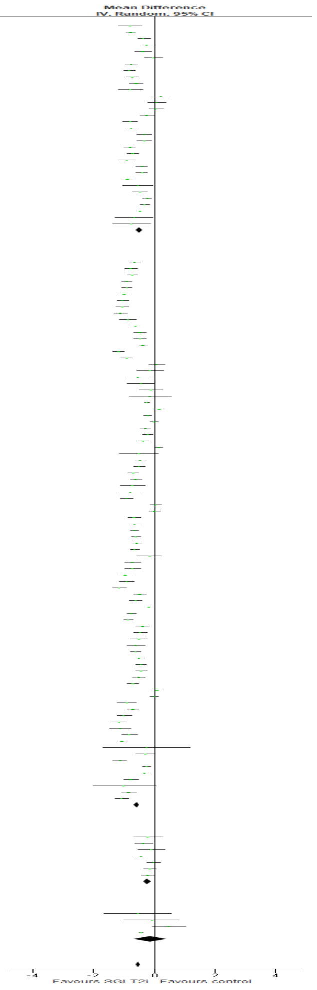

Figure S3. Weight changes before and after SGLT2i treatment in patients with different levels of renal function.

Study or Subgroup	SGLT2i Mean	SGLT2i SD	SGLT2i Total	control Mean	control SD	control Total	Weight	Mean Difference IV, Random, 95% CI
**7.1.1 eGFR >=90**								
CANA Lavalle-González 2013-2	-3.6	3.831	367	-1.1	2.706	183	1.0%	-2.50 [-3.05, -1.95]
DAPA Handelsman 2018	-1.86	3.062	232	-0.51	3.145	229	1.0%	-1.35 [-1.92, -0.78]
DAPA Jabbour 2018	-3.31	5.738	228	-1.51	6.027	227	0.9%	-1.80 [-2.88, -0.72]
DAPA Jeon 2018	-2.36	0.51	162	1.93	0.49	148	1.1%	-4.29 [-4.40, -4.18]
DAPA Ji 2014-1	-1.64	2.626	128	-0.27	2.638	132	1.0%	-1.37 [-2.01, -0.73]
DAPA Ji 2014-2	-2.25	2.648	133	-0.27	2.638	132	1.0%	-1.98 [-2.62, -1.34]
DAPA Mathieu 2015	-1.9	2.775	160	-0.4	2.904	160	1.0%	-1.50 [-2.12, -0.88]
DAPA Rosenstock 2015	-2.1	3.072	179	0	3.384	176	1.0%	-2.10 [-2.77, -1.43]
EMPA Ferdinand 2019	-2.21	3.621	78	-0.98	3.564	72	0.9%	-1.23 [-2.38, -0.08]
EMPA Ferrannini 2013-1-1	-2.2	3.879	80	-1.3	3.818	56	0.8%	-0.90 [-2.21, 0.41]
EMPA Ferrannini 2013-1-2	-2.6	4.068	88	-1.3	3.818	56	0.8%	-1.30 [-2.61, 0.01]
EMPA Ferrannini 2013-2-1	-3.1	4.479	137	-0.4	4.2	56	0.8%	-2.70 [-4.03, -1.37]
EMPA Ferrannini 2013-2-2	-4	4.511	139	-0.4	4.2	56	0.8%	-3.60 [-4.93, -2.27]
EMPA Hadjadj 2016-1-1	-2.77	3.807	155	-0.52	3.888	140	1.0%	-2.25 [-3.13, -1.37]
EMPA Hadjadj 2016-1-2	-3.04	3.854	149	-0.52	3.888	140	0.9%	-2.52 [-3.41, -1.63]
EMPA Hadjadj 2016-2-1	-3.48	3.877	150	-1.27	3.842	148	1.0%	-2.21 [-3.09, -1.33]
EMPA Hadjadj 2016-2-2	-3.78	3.77	160	-1.27	3.842	148	1.0%	-2.51 [-3.36, -1.66]
ERTU Ji 2019-1	-2.95	2.295	170	-1.17	2.407	167	1.0%	-1.78 [-2.28, -1.28]
ERTU Ji 2019-2	-3.18	2.288	169	-1.17	2.407	167	1.0%	-2.01 [-2.51, -1.51]
ERTU Miller 2018-1	-2.94	3.334	98	-0.94	3.819	97	0.9%	-2.00 [-3.01, -0.99]
ERTU Pratley 2018-1	-2.5	3.579	243	-0.7	3.608	247	1.0%	-1.80 [-2.44, -1.16]
ERTU Pratley 2018-2	-2.9	3.586	244	-0.7	3.608	247	1.0%	-2.20 [-2.84, -1.56]
ERTU Rosenstock 2018-2	-2.93	2.922	205	-1.33	3.024	209	1.0%	-1.60 [-2.17, -1.03]
IPRA Inoue 2019	-2.78	1.96	24	-0.22	1.862	24	0.9%	-2.56 [-3.64, -1.48]
IPRA Lu 2016	-2.93	2.2	87	-1.7	2.43	83	1.0%	-1.23 [-1.93, -0.53]
SOTA Danne 2018-1	-1.88	4.152	261	0.3	4.128	258	1.0%	-2.18 [-2.89, -1.47]
SOTA Danne 2018-2	-2.63	4.135	263	0.3	4.128	258	1.0%	-2.93 [-3.64, -2.22]
SOTA Garg 2017	-2.21	3.173	699	0.77	3.182	703	1.0%	-2.98 [-3.31, -2.65]
SOTA Zambrowicz 2012-1	-3	12.45	12	-2	8	12	0.1%	-1.00 [-9.37, 7.37]
SOTA Zambrowicz 2012-2	-4	15.46	12	-2	8	12	0.1%	-2.00 [-11.85, 7.85]
**Subtotal (95% CI)**			**5212**			**4743**	**27.1%**	**-2.12 [-2.66, -1.59]**
Heterogeneity: Tau² = 1.92; Chi² = 693.64, df = 29 (P < 0.00001); I² = 96%								
Test for overall effect: Z = 7.78 (P < 0.00001)								
**7.1.2 eGFR 60-89**								
CANA Inagaki 2016	-1.49	1.569	76	0.15	1.506	70	1.0%	-1.64 [-2.14, -1.14]
CANA Kadowaki 2017	-2.29	1.841	70	-0.78	1.897	68	1.0%	-1.51 [-2.13, -0.89]
CANA Lavalle-González 2013-1	-3.3	3.837	368	-1.1	2.706	183	1.0%	-2.20 [-2.75, -1.65]
CANA Rosenstock 2016-1	-3.9	3.079	237	-1.9	3.079	237	1.0%	-2.00 [-2.55, -1.45]
CANA Rosenstock 2016-2	-3.2	3.079	237	-1.9	3.079	237	1.0%	-1.30 [-1.85, -0.75]
DAPA Araki 2017	-1.5	2.085	122	-1.37	2.332	60	1.0%	-0.13 [-0.83, 0.57]
DAPA Bailey 2015-1	-0.58	6.335	65	-1.34	6.628	75	0.6%	0.76 [-1.39, 2.91]
DAPA Bailey 2015-2	-1.59	6.122	64	-1.34	6.628	75	0.6%	-0.25 [-2.37, 1.87]
DAPA Bailey 2015-3	-3.94	6.275	70	-1.34	6.628	75	0.6%	-2.60 [-4.70, -0.50]
DAPA Bolinder 2012	-2.96	2.647	89	-0.88	2.653	91	1.0%	-2.08 [-2.85, -1.31]
DAPA Cho 2018	-4	15.2	36	0.5	13.95	35	0.1%	-4.50 [-11.28, 2.28]
DAPA Hayashi 2017	-2.2	15.04	40	0.1	11.6	40	0.2%	-2.30 [-8.19, 3.59]
DAPA McMurray 2019	-0.88	3.86	2373	0.1	4.09	2371	1.1%	-0.98 [-1.21, -0.75]
DAPA Müller-Wieland 2018-1	-3.54	4.093	314	1.76	3.963	313	1.0%	-5.30 [-5.93, -4.67]
DAPA Müller-Wieland 2018-2	-3.15	3.868	312	1.76	3.963	313	1.0%	-4.91 [-5.52, -4.30]
DAPA Nauck 2011	-3.22	3.52	400	1.44	3.525	401	1.0%	-4.66 [-5.15, -4.17]
DAPA Schumm-Draeger 2015-1	-2.76	3.071	99	-1.04	3.12	101	1.0%	-1.72 [-2.58, -0.86]
DAPA Schumm-Draeger 2015-2	-2.84	3.099	100	-1.04	3.12	101	1.0%	-1.80 [-2.66, -0.94]
DAPA Schumm-Draeger 2015-3	-3.2	3.109	99	-1.04	3.12	101	1.0%	-2.16 [-3.02, -1.30]
DAPA Strojek 2011-1	-1.18	2.754	154	-0.72	2.703	145	1.0%	-0.46 [-1.08, 0.16]
DAPA Strojek 2011-2	-1.56	2.736	142	-0.72	2.703	145	1.0%	-0.84 [-1.47, -0.21]
DAPA Strojek 2011-3	-2.26	2.727	151	-0.72	2.703	145	1.0%	-1.54 [-2.16, -0.92]
DAPA Weber 2016	-1.44	3.941	225	-0.59	3.971	224	1.0%	-0.85 [-1.58, -0.12]
DAPA Wilding 2009-1	-4.5	2.499	24	-1.9	2.447	23	0.8%	-2.60 [-4.01, -1.19]
DAPA Wilding 2009-2	-4.3	2.499	24	-1.9	2.447	23	0.8%	-2.40 [-3.81, -0.99]
DAPA Yang 2018	-1	2.466	139	0.37	2.56	133	1.0%	-1.37 [-1.97, -0.77]
EMPA Araki 2015-1	-2.3	2.332	136	-0.1	2.381	63	1.0%	-2.20 [-2.91, -1.49]
EMPA Araki 2015-2	-2.8	2.341	137	-0.1	2.381	63	1.0%	-2.70 [-3.41, -1.99]
EMPA Barnett 2014-1	-2.6	3.166	97	-0.44	3.133	95	0.9%	-2.16 [-3.05, -1.27]
EMPA Barnett 2014-2	-2	3.182	98	-0.44	3.133	95	0.9%	-1.56 [-2.45, -0.67]
EMPA Haring 2013-1	-2.16	2.25	225	-0.39	2.25	225	1.0%	-1.77 [-2.19, -1.35]
EMPA Haring 2013-2	-2.39	2.352	216	-0.39	2.25	225	1.0%	-2.00 [-2.43, -1.57]
EMPA Haring 2014-1	-2.08	2.504	217	-0.45	2.446	207	1.0%	-1.63 [-2.10, -1.16]
EMPA Haring 2014-2	-2.46	2.481	213	-0.45	2.446	207	1.0%	-2.01 [-2.48, -1.54]
EMPA Kadowaki 2014-1	-2.5	2.098	110	-0.9	2.088	109	1.0%	-1.60 [-2.15, -1.05]
EMPA Kadowaki 2014-2	-2.6	2.088	109	-0.9	2.088	109	1.0%	-1.70 [-2.25, -1.15]
EMPA Kadowaki 2014-3	-2.9	2.088	109	-0.9	2.088	109	1.0%	-2.00 [-2.55, -1.45]
EMPA Kadowaki 2014-4	-3.1	2.098	110	-0.9	2.088	109	1.0%	-2.20 [-2.75, -1.65]
EMPA Kovacs 2014-1	-1.62	2.697	165	0.34	2.697	165	1.0%	-1.96 [-2.54, -1.38]
EMPA Kovacs 2014-2	-1.47	2.722	168	0.34	2.697	165	1.0%	-1.81 [-2.39, -1.23]
EMPA Packer 2020	-0.73	5.611	1863	0.08	5.617	1867	1.0%	-0.81 [-1.17, -0.45]
EMPA Roden 2013-1	-2.26	2.596	224	-0.33	2.581	228	1.0%	-1.93 [-2.41, -1.45]
EMPA Roden 2013-2	-2.48	2.596	224	-0.33	2.581	228	1.0%	-2.15 [-2.63, -1.67]
EMPA Rosenstock 2014-1	-1.95	4.91	186	0.44	4.936	188	0.9%	-2.39 [-3.39, -1.39]
EMPA Rosenstock 2014-2	-2.04	4.949	189	0.44	4.936	188	0.9%	-2.48 [-3.48, -1.48]
EMPA Rosenstock 2015-1	-2.2	6.5	169	0.7	6.519	170	0.8%	-2.90 [-4.29, -1.51]
EMPA Rosenstock 2015-2	-2	6.225	155	0.7	6.519	170	0.8%	-2.70 [-4.09, -1.31]
EMPA Ross 2015-1	-3.2	2.639	215	-0.97	2.586	107	1.0%	-2.23 [-2.83, -1.63]
EMPA Ross 2015-2	-2.89	2.633	214	-0.97	2.586	107	1.0%	-1.92 [-2.52, -1.32]
EMPA Ross 2015-3	-2.93	2.639	215	-0.97	2.586	107	1.0%	-1.96 [-2.56, -1.36]
EMPA Ross 2015-4	-2.71	2.633	214	-0.97	2.586	107	1.0%	-1.74 [-2.34, -1.14]
ERTU Dagogo-Jack 2018-1	-3.5	3.823	156	-1	4.102	153	1.0%	-2.50 [-3.38, -1.62]
ERTU Dagogo-Jack 2018-2	-2.8	3.787	153	-1	4.102	153	1.0%	-1.80 [-2.68, -0.92]
ERTU Hollander 2019-1	-2.9	4.305	445	1	4.789	435	1.0%	-3.90 [-4.50, -3.30]
ERTU Hollander 2019-2	-3.4	5.321	435	1	4.789	435	1.0%	-4.40 [-5.07, -3.73]
ERTU Miller 2018-2	-3.04	3.324	96	-0.94	3.819	97	0.9%	-2.10 [-3.11, -1.09]
ERTU Rosenstock 2018-1	-3.01	2.863	207	-1.33	3.024	209	1.0%	-1.68 [-2.25, -1.11]
ERTU Terra 2017-1	-3.18	3.473	156	-1.42	3.818	153	1.0%	-1.76 [-2.57, -0.95]
ERTU Terra 2017-2	-3.58	3.491	152	-1.42	3.818	153	1.0%	-2.16 [-2.98, -1.34]
IPRA Bando 2016	-1.51	1.28	40	0.45	0.77	22	1.0%	-1.96 [-2.47, -1.45]
IPRA Han 2018	-1.96	1.94	73	-0.23	1.69	66	1.0%	-1.73 [-2.33, -1.13]
IPRA Ishihara 2016	-1.09	1.27	168	-0.05	1.42	87	1.0%	-1.04 [-1.39, -0.69]
IPRA Kashiwagi 2015	-1.87	17.21	118	-0.06	9.97	46	0.3%	-1.81 [-6.05, 2.43]
IPRA Tsurutani 2018	-2.2	1.778	60	-0.17	1.646	59	1.0%	-2.03 [-2.65, -1.41]
LUSEO Seino 2018	-1.32	1.608	159	-0.05	1.47	74	1.0%	-1.27 [-1.69, -0.85]
SOTA Buse 2018-1	-1.94	4.006	263	1.2	4.076	268	1.0%	-3.14 [-3.83, -2.45]
SOTA Buse 2018-2	-3.12	4.047	262	1.2	4.076	268	1.0%	-4.32 [-5.01, -3.63]
TOFO Kaku 2014-1	-2.23	1.826	57	-0.356	1.831	56	1.0%	-1.87 [-2.55, -1.20]
TOFO Kaku 2014-2	-2.851	1.822	58	-0.356	1.831	56	1.0%	-2.50 [-3.17, -1.82]
TOFO Kaku 2014-3	-2.971	1.822	58	-0.356	1.831	56	1.0%	-2.62 [-3.29, -1.94]
TOFO Terauchi 2017	-1.34	1.62	141	0.03	1.36	70	1.0%	-1.37 [-1.79, -0.95]
**Subtotal (95% CI)**			**15265**			**14114**	**66.8%**	**-2.06 [-2.31, -1.82]**
Heterogeneity: Tau² = 0.90; Chi² = 737.00, df = 70 (P < 0.00001); I² = 91%								
Test for overall effect: Z = 16.64 (P < 0.00001)								
**7.1.3 eGFR 45-59**								
DAPA Fioretto 2018	-3.17	3.808	160	-1.92	3.787	161	1.0%	-1.25 [-2.08, -0.42]
EMPA Barnett 2014-3	-1.17	2.861	187	0	2.861	187	1.0%	-1.17 [-1.75, -0.59]
LUSEO Haneda 2016	-1.29	1.989	95	-0.02	1.443	50	1.0%	-1.27 [-1.84, -0.70]
**Subtotal (95% CI)**			**442**			**398**	**3.0%**	**-1.23 [-1.59, -0.86]**
Heterogeneity: Tau² = 0.00; Chi² = 0.06, df = 2 (P = 0.97); I² = 0%								
Test for overall effect: Z = 6.61 (P < 0.00001)								
**7.1.4 eGFR <45**								
DAPA Kohan 2013-1	-0.34	6.833	83	2.63	7.24	84	0.6%	-2.97 [-5.10, -0.84]
DAPA Kohan 2013-2	-1.1	6.822	85	2.63	7.24	84	0.6%	-3.73 [-5.85, -1.61]
EMPA Barnett 2014-4	-1	3.3	37	0	3.6	37	0.8%	-1.00 [-2.57, 0.57]
SOTA Bhatt 2021	-1.4	2.784	5292	-0.24	2.784	5292	1.1%	-1.16 [-1.27, -1.05]
**Subtotal (95% CI)**			**5497**			**5497**	**3.1%**	**-1.88 [-3.04, -0.72]**
Heterogeneity: Tau² = 0.84; Chi² = 8.40, df = 3 (P = 0.04); I² = 64%								
Test for overall effect: Z = 3.18 (P = 0.001)								
**Total (95% CI)**			**26416**			**24752**	**100.0%**	**-2.05 [-2.31, -1.79]**
Heterogeneity: Tau² = 1.66; Chi² = 2643.33, df = 107 (P < 0.00001); I² = 96%								
Test for overall effect: Z = 15.42 (P < 0.00001)								
Test for subgroup differences: Chi² = 15.25, df = 3 (P = 0.002), I² = 80.3%								

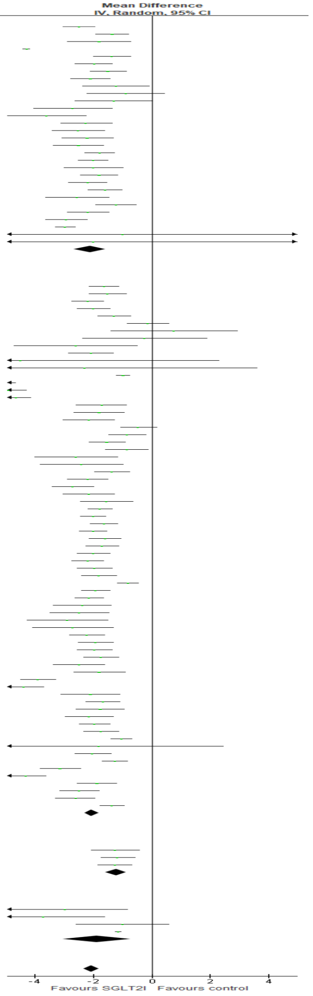

Figure S4. SBP changes before and after SGLT2i treatment in patients with different levels of renal function.

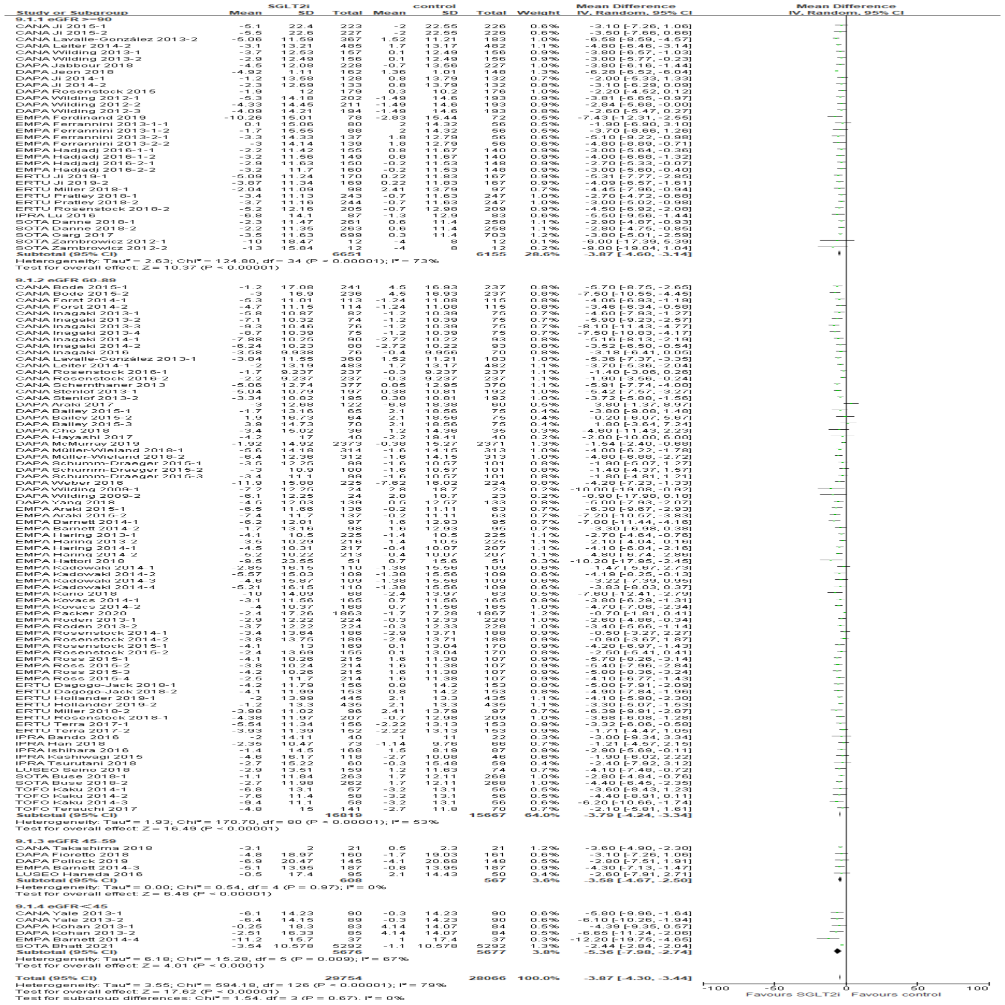

Figure S5. DBP changes before and after SGLT2i treatment in patients with different levels of renal function.

Study or Subgroup	SGLT2i Mean	SGLT2i SD	SGLT2i Total	control Mean	control SD	control Total	Weight	Mean Difference IV, Fixed, 95% CI
**9.2.1 eGFR >=90**								
CANA Ji 2015-1	-2.4	13.44	223	-1.8	13.53	226	0.2%	-0.60 [-3.09, 1.89]
CANA Ji 2015-2	-3	13.56	227	-1.8	13.53	226	0.2%	-1.20 [-3.69, 1.29]
CANA Lavalle-González 2013-2	-2.1	7.663	367	0.3	6.764	183	0.8%	-2.40 [-3.66, -1.14]
CANA Leiter 2014-2	-2.2	8.809	485	-0.02	8.343	482	1.1%	-2.18 [-3.26, -1.10]
CANA Wilding 2013-1	-2.2	7.518	157	-0.7	7.494	156	0.5%	-1.50 [-3.16, 0.16]
CANA Wilding 2013-2	-1.7	7.494	156	-0.7	7.494	156	0.5%	-1.00 [-2.66, 0.66]
DAPA Jabbour 2018	-0.7	9.06	228	0.2	10.55	227	0.4%	-0.90 [-2.71, 0.91]
DAPA Jeon 2018	-2.45	1.37	162	-0.59	0.54	148	25.6%	-1.86 [-2.09, -1.63]
DAPA Ji 2014-1	-1.3	10.18	128	0.4	9.191	132	0.2%	-1.70 [-4.06, 0.66]
DAPA Ji 2014-2	-1.6	9.226	133	0.4	9.191	132	0.3%	-2.00 [-4.22, 0.22]
DAPA Rosenstock 2015	-1	8	179	-0.4	7	176	0.5%	-0.60 [-2.16, 0.96]
DAPA Wilding 2012-1	-2.96	8.267	202	-1.31	8.506	193	0.5%	-1.65 [-3.31, 0.01]
DAPA Wilding 2012-2	-2.64	8.412	211	-1.31	8.506	193	0.5%	-1.33 [-2.98, 0.32]
DAPA Wilding 2012-3	-2.85	8.243	194	-1.31	8.506	193	0.5%	-1.54 [-3.21, 0.13]
EMPA Ferdinand 2019	-5.55	9.008	78	-1.3	9.249	72	0.2%	-4.25 [-7.18, -1.32]
EMPA Ferrannini 2013-1-1	-1.6	7.986	80	-0.6	8.018	56	0.2%	-1.00 [-3.73, 1.73]
EMPA Ferrannini 2013-1-2	-2.2	8.136	88	-0.6	8.018	56	0.2%	-1.60 [-4.30, 1.10]
EMPA Ferrannini 2013-2-1	-0.9	8.958	137	1.2	8.018	56	0.2%	-2.10 [-4.68, 0.48]
EMPA Ferrannini 2013-2-2	-2	9.023	139	1.2	8.018	56	0.2%	-3.20 [-5.78, -0.62]
EMPA Hadjadj 2016-1-1	-1.6	7.613	155	0.6	7.777	140	0.4%	-2.20 [-3.96, -0.44]
EMPA Hadjadj 2016-1-2	-1.6	7.707	149	0.6	7.777	140	0.4%	-2.20 [-3.99, -0.41]
EMPA Hadjadj 2016-2-1	-1.9	7.754	150	0	7.684	148	0.4%	-1.90 [-3.65, -0.15]
EMPA Hadjadj 2016-2-2	-1.9	7.8	160	0	7.684	148	0.4%	-1.90 [-3.63, -0.17]
ERTU Ji 2019-1	-2.38	7.517	170	-0.96	7.912	167	0.5%	-1.42 [-3.07, 0.23]
ERTU Ji 2019-2	-2.36	7.561	169	-0.96	7.912	167	0.5%	-1.40 [-3.06, 0.26]
ERTU Miller 2018-1	-0.44	7.829	98	1.21	9.748	97	0.2%	-1.65 [-4.13, 0.83]
ERTU Pratley 2018-1	-0.7	6.76	243	-0.3	6.816	247	0.9%	-0.40 [-1.60, 0.80]
ERTU Pratley 2018-2	-1.3	6.376	244	-0.3	6.816	247	1.0%	-1.00 [-2.17, 0.17]
ERTU Rosenstock 2018-2	-2.19	7.451	205	0.23	7.966	209	0.6%	-2.42 [-3.91, -0.93]
IPRA Lu 2016	-3.7	9.4	87	-1	8	83	0.2%	-2.70 [-5.32, -0.08]
SOTA Danne 2018-1	-1.6	7.593	261	-0.3	7.549	258	0.8%	-1.30 [-2.60, 0.00]
SOTA Danne 2018-2	-0.9	7.622	263	-0.3	7.549	258	0.8%	-0.60 [-1.90, 0.70]
SOTA Garg 2017	-0.8	7.932	699	0.5	7.689	703	2.0%	-1.30 [-2.12, -0.48]
SOTA Zambrowicz 2012-1	-7	9.434	12	0	10.77	12	0.0%	-7.00 [-15.10, 1.10]
SOTA Zambrowicz 2012-2	-5	11.45	12	0	10.77	12	0.0%	-5.00 [-13.89, 3.89]
**Subtotal (95% CI)**			**6651**			**6155**	**42.1%**	**-1.73 [-1.91, -1.55]**
Heterogeneity: Chi² = 27.38, df = 34 (P = 0.78); I² = 0%								
Test for overall effect: Z = 19.03 (P < 0.00001)								
**9.2.2 eGFR 60-89**								
CANA Bode 2015-1	-0.8	9.315	241	0.8	9.237	237	0.5%	-1.60 [-3.26, 0.06]
CANA Bode 2015-2	-1.8	9.217	236	0.8	9.237	237	0.5%	-2.60 [-4.26, -0.94]
CANA Forst 2014-1	-3.3	7.441	113	-0.9	7.507	115	0.4%	-2.40 [-4.34, -0.46]
CANA Forst 2014-2	-3.5	7.474	114	-0.9	7.507	115	0.4%	-2.60 [-4.54, -0.66]
CANA Inagaki 2013-1	-2.2	7.244	82	-0.9	7.794	75	0.2%	-1.30 [-3.66, 1.06]
CANA Inagaki 2013-2	-3.9	7.742	74	-0.9	7.794	75	0.2%	-3.00 [-5.49, -0.51]
CANA Inagaki 2013-3	-5.1	6.974	76	-0.9	7.794	75	0.2%	-4.20 [-6.56, -1.84]
CANA Inagaki 2013-4	-4.2	6.928	75	-0.9	7.794	75	0.2%	-3.30 [-5.66, -0.94]
CANA Inagaki 2014-1	-4.44	6.925	90	-1.84	6.943	93	0.3%	-2.60 [-4.61, -0.59]
CANA Inagaki 2014-2	-2.66	6.942	88	-1.84	6.943	93	0.3%	-0.82 [-2.84, 1.20]
CANA Inagaki 2016	-1.55	6.19	76	-0.31	6.191	70	0.3%	-1.24 [-3.25, 0.77]
CANA Lavalle-González 2013-1	-2.2	7.673	368	0.3	6.764	183	0.8%	-2.50 [-3.75, -1.25]
CANA Leiter 2014-1	-1.3	8.791	483	-0.02	8.343	482	1.1%	-1.28 [-2.36, -0.20]
CANA Rosenstock 2016-1	-1.02	6.158	237	-0.5	6.158	237	1.1%	-0.52 [-1.63, 0.59]
CANA Rosenstock 2016-2	-1.5	6.158	237	-0.5	6.158	237	1.1%	-1.00 [-2.11, 0.11]
CANA Schernthaner 2013	-3	7.767	377	-0.3	7.777	378	1.1%	-2.70 [-3.81, -1.59]
CANA Stenlof 2013-1	-2.1	7.018	197	-0.1	6.928	192	0.7%	-2.00 [-3.39, -0.61]
CANA Stenlof 2013-2	-1.7	6.982	195	-0.1	6.928	192	0.7%	-1.60 [-2.99, -0.21]
DAPA Araki 2017	-3.9	9.298	122	-3.9	9.88	60	0.1%	0.00 [-3.00, 3.00]
DAPA Bailey 2015-1	-1.1	11.52	65	0.5	11.05	75	0.1%	-1.60 [-5.35, 2.15]
DAPA Bailey 2015-2	-1.4	11.43	64	0.5	11.05	75	0.1%	-1.90 [-5.65, 1.85]
DAPA Bailey 2015-3	1.7	10.67	70	0.5	11.05	75	0.1%	1.20 [-2.34, 4.74]
DAPA Cho 2018	-3.6	10.92	36	0.7	13.82	35	0.0%	-4.30 [-10.10, 1.50]
DAPA Hayashi 2017	-4.6	17.68	40	-3.7	11.63	40	0.0%	-0.90 [-7.46, 5.66]
DAPA Schumm-Draeger 2015-1	-1	8.05	99	-0.9	6.18	101	0.3%	-0.10 [-2.09, 1.89]
DAPA Schumm-Draeger 2015-2	-1.3	7.27	100	-0.9	6.18	101	0.4%	-0.40 [-2.27, 1.47]
DAPA Schumm-Draeger 2015-3	-2.4	7.61	99	-0.9	6.18	101	0.4%	-1.50 [-3.42, 0.42]
DAPA Weber 2016	-6.3	9.462	225	-5.33	9.544	224	0.4%	-0.97 [-2.73, 0.79]
DAPA Wilding 2009-1	-1.2	9.308	24	0.3	13.91	23	0.0%	-1.50 [-8.30, 5.30]
DAPA Wilding 2009-2	-3.9	7.838	24	0.3	13.91	23	0.0%	-4.20 [-10.69, 2.29]
DAPA Yang 2018	-0.4	6.956	139	-0.1	7.266	133	0.5%	-0.30 [-1.99, 1.39]
EMPA Araki 2015-1	-3.1	6.997	136	0.1	6.35	63	0.3%	-3.20 [-5.16, -1.24]
EMPA Araki 2015-2	-3.5	7.023	137	0.1	6.35	63	0.3%	-3.60 [-5.56, -1.64]
EMPA Barnett 2014-1	-3	7.035	97	1.5	6.962	95	0.3%	-4.50 [-6.48, -2.52]
EMPA Barnett 2014-2	-1.2	7.071	98	1.5	6.962	95	0.3%	-2.70 [-4.68, -0.72]
EMPA Haring 2013-1	-2.1	6	225	-1.8	6	225	1.1%	-0.30 [-1.41, 0.81]
EMPA Haring 2013-2	-2.2	5.879	216	-1.8	6	225	1.1%	-0.40 [-1.51, 0.71]
EMPA Haring 2014-1	-2	7.365	217	0	7.194	207	0.7%	-2.00 [-3.39, -0.61]
EMPA Haring 2014-2	-1.6	7.297	213	0	7.194	207	0.7%	-1.60 [-2.99, -0.21]
EMPA Hattori 2018	-5.3	10.82	51	-2	15.55	51	0.0%	-3.30 [-8.50, 1.90]
EMPA Kadowaki 2014-1	-2.2	9.754	110	-2	9.396	109	0.2%	-0.20 [-2.74, 2.34]
EMPA Kadowaki 2014-2	-3.57	9.083	109	-2	9.396	109	0.2%	-1.57 [-4.02, 0.88]
EMPA Kadowaki 2014-3	-3.35	9.605	109	-2	9.396	109	0.2%	-1.35 [-3.87, 1.17]
EMPA Kadowaki 2014-4	-3.03	9.754	110	-2	9.396	109	0.2%	-1.03 [-3.57, 1.51]
EMPA Kario 2018	-3.5	6.101	68	-0.7	6.074	63	0.3%	-2.80 [-4.89, -0.71]
EMPA Kovacs 2014-1	-1.5	6.423	165	0.3	6.423	165	0.7%	-1.80 [-3.19, -0.41]
EMPA Kovacs 2014-2	-2.2	6.481	168	0.3	6.423	165	0.7%	-2.50 [-3.89, -1.11]
EMPA Roden 2013-1	-1.1	7.254	224	-0.5	7.319	228	0.7%	-0.60 [-1.94, 0.74]
EMPA Roden 2013-2	-1.9	7.254	224	-0.5	7.319	228	0.7%	-1.40 [-2.74, -0.06]
EMPA Rosenstock 2014-1	-1.2	8.183	186	-0.5	8.227	188	0.5%	-0.70 [-2.36, 0.96]
EMPA Rosenstock 2014-2	-2.5	8.249	189	-0.5	8.227	188	0.5%	-2.00 [-3.66, -0.34]
EMPA Rosenstock 2015-1	-2.9	9.1	169	-0.3	7.823	170	0.4%	-2.60 [-4.41, -0.79]
EMPA Rosenstock 2015-2	-1.5	8.715	155	-0.3	7.823	170	0.4%	-1.20 [-3.01, 0.61]
EMPA Ross 2015-1	-2.1	7.331	215	0.4	6.206	107	0.6%	-2.50 [-4.03, -0.97]
EMPA Ross 2015-2	-2.6	7.314	214	0.4	6.206	107	0.6%	-3.00 [-4.53, -1.47]
EMPA Ross 2015-3	-1.6	7.331	215	0.4	6.206	107	0.6%	-2.00 [-3.53, -0.47]
EMPA Ross 2015-4	-0.8	7.314	214	0.4	6.206	107	0.6%	-1.20 [-2.73, 0.33]
ERTU Dagogo-Jack 2018-1	-1.5	7.647	156	-0.5	9.151	153	0.4%	-1.00 [-2.88, 0.88]
ERTU Dagogo-Jack 2018-2	-1.4	7.573	153	-0.5	9.151	153	0.4%	-0.90 [-2.78, 0.98]
ERTU Hollander 2019-1	-1.2	9.148	445	0.7	8.513	435	1.0%	-1.90 [-3.07, -0.73]
ERTU Hollander 2019-2	-0.3	9.045	435	0.7	8.513	435	1.0%	-1.00 [-2.17, 0.17]
ERTU Miller 2018-2	-0.44	7.829	98	1.21	9.748	97	0.2%	-1.65 [-4.13, 0.83]
ERTU Rosenstock 2018-1	-0.97	7.773	96	0.23	7.966	209	0.4%	-1.20 [-3.09, 0.69]
ERTU Terra 2017-1	-2.52	7.169	156	-0.72	8.362	153	0.4%	-1.80 [-3.54, -0.06]
ERTU Terra 2017-2	-1.1	7.202	152	-0.72	8.362	153	0.4%	-0.38 [-2.13, 1.37]
IPRA Bando 2016	-1	8	40	1	9.434	22	0.1%	-2.00 [-6.66, 2.66]
IPRA Han 2018	-1.49	6.28	73	-0.17	5.53	66	0.3%	-1.32 [-3.28, 0.64]
IPRA Ishihara 2016	-0.3	9.9	168	0.3	9.2	87	0.2%	-0.60 [-3.05, 1.85]
IPRA Kashiwagi 2015	-2.5	9.77	118	-1.2	7.64	46	0.2%	-1.30 [-4.13, 1.53]
IPRA Tsurutani 2018	0	11.46	60	0.4	11.56	59	0.1%	-0.40 [-4.54, 3.74]
LUSEO Seino 2018	-2.3	8.363	159	0.2	8.558	74	0.2%	-2.50 [-4.84, -0.16]
SOTA Buse 2018-1	-0.6	7.46	263	0.9	7.531	268	0.8%	-1.50 [-2.78, -0.22]
SOTA Buse 2018-2	-1.4	7.446	262	0.9	7.531	268	0.8%	-2.30 [-3.58, -1.02]
TOFO Kaku 2014-1	-5.6	9.8	57	-1.4	9.8	56	0.1%	-4.20 [-7.81, -0.59]
TOFO Kaku 2014-2	-4.1	8.4	58	-1.4	9.8	56	0.1%	-2.70 [-6.06, 0.66]
TOFO Kaku 2014-3	-4.1	8.3	58	-1.4	9.8	56	0.1%	-2.70 [-6.04, 0.64]
TOFO Terauchi 2017	-1.8	9.4	141	0.4	7.7	70	0.2%	-2.20 [-4.58, 0.18]
**Subtotal (95% CI)**			**11848**			**10803**	**33.4%**	**-1.63 [-1.83, -1.43]**
Heterogeneity: Chi² = 86.35, df = 76 (P = 0.20); I² = 12%								
Test for overall effect: Z = 16.00 (P < 0.00001)								
**9.2.3 eGFR 45-59**								
CANA Takashima 2018	-1.6	1	21	0.6	1.6	21	2.0%	-2.20 [-3.01, -1.39]
EMPA Barnett 2014-3	-1.8	8.372	187	-0.3	8.372	187	0.5%	-1.50 [-3.20, 0.20]
LUSEO Haneda 2016	-0.9	9.946	95	-1.2	10.82	50	0.1%	0.30 [-3.30, 3.90]
**Subtotal (95% CI)**			**303**			**258**	**2.6%**	**-1.98 [-2.69, -1.26]**
Heterogeneity: Chi² = 2.13, df = 2 (P = 0.34); I² = 6%								
Test for overall effect: Z = 5.43 (P < 0.00001)								
**9.2.4 eGFR <45**								
CANA Yale 2013-1	-2.6	8.538	90	-1.4	8.538	90	0.2%	-1.20 [-3.69, 1.29]
CANA Yale 2013-2	-3.5	8.491	89	-1.4	8.538	90	0.2%	-2.10 [-4.59, 0.39]
DAPA Kohan 2013-1	-0.55	11.63	83	-0.48	9.6	84	0.1%	-0.07 [-3.31, 3.17]
DAPA Kohan 2013-2	-1.27	9.49	85	-0.48	9.6	84	0.2%	-0.79 [-3.67, 2.09]
EMPA Barnett 2014-4	-4.3	8.8	37	1.4	8.7	37	0.1%	-5.70 [-9.69, -1.71]
SOTA Bhatt 2021	-2.05	6.495	5292	-1.25	6.681	5292	21.2%	-0.80 [-1.05, -0.55]
**Subtotal (95% CI)**			**5676**			**5677**	**22.0%**	**-0.83 [-1.08, -0.58]**
Heterogeneity: Chi² = 7.08, df = 5 (P = 0.21); I² = 29%								
Test for overall effect: Z = 6.61 (P < 0.00001)								
**Total (95% CI)**			**24478**			**22893**	**100.0%**	**-1.51 [-1.62, -1.39]**
Heterogeneity: Chi² = 160.98, df = 120 (P = 0.007); I² = 25%								
Test for overall effect: Z = 25.55 (P < 0.00001)								
Test for subgroup differences: Chi² = 38.04, df = 3 (P < 0.00001), I² = 92.1%								

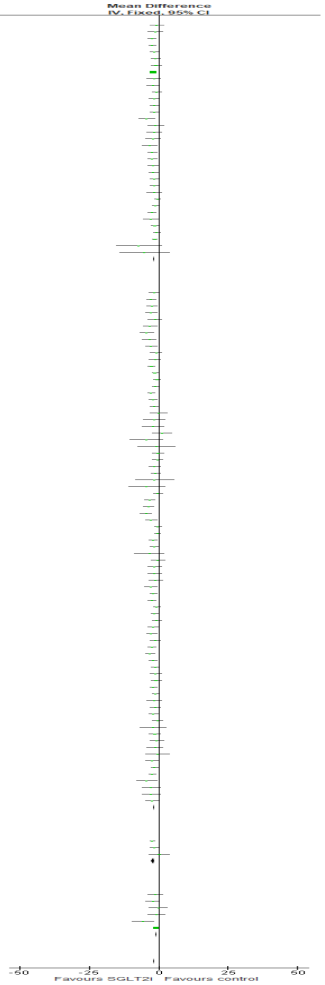

Figure S6. eGFR changes before and after SGLT2i treatment in patients with different levels of renal function.

Study or Subgroup	SGLT2i Mean	SGLT2i SD	SGLT2i Total	Control Mean	Control SD	Control Total	Weight	Mean Difference IV, Random, 95% CI
**10.5.1 eGFR >=90**								
CANA Wilding 2013-1	-1.6	13.7	157	-1.9	12.9	156	1.1%	0.30 [-2.65, 3.25]
CANA Wilding 2013-2	-5.8	14.5	156	-1.9	12.9	156	1.0%	-3.90 [-6.95, -0.85]
DAPA Ji 2014-1	2.3	11.99	128	0.8	12.18	132	1.1%	1.50 [-1.44, 4.44]
DAPA Ji 2014-2	0.9	11.3	133	0.8	12.18	132	1.1%	0.10 [-2.73, 2.93]
DAPA Wilding 2012-1	-4.8	14.5	202	-0.8	14.4	193	1.1%	-4.00 [-6.85, -1.15]
DAPA Wilding 2012-2	-4.7	16.4	211	-0.8	14.4	193	1.0%	-3.90 [-6.90, -0.90]
DAPA Wilding 2012-3	-4.4	13.2	194	-0.8	14.4	193	1.1%	-3.60 [-6.35, -0.85]
EMPA Hadjadj 2016-1-1	-0.5	13.8	155	-1.7	12.7	140	1.0%	1.20 [-1.82, 4.22]
EMPA Hadjadj 2016-1-2	0.3	13.4	149	-1.7	12.7	140	1.0%	2.00 [-1.01, 5.01]
EMPA Hadjadj 2016-2-1	2.2	11.5	150	2.1	12.9	148	1.1%	0.10 [-2.68, 2.88]
EMPA Hadjadj 2016-2-2	3.4	16.6	160	2.1	12.9	148	0.9%	1.30 [-2.01, 4.61]
EMPA Lewin 2015-2	0.8	11.6	134	-0.5	10.9	133	1.2%	1.30 [-1.40, 4.00]
ERTU Ji 2019-1	-0.5	12.1	170	-2.5	12.9	167	1.2%	2.00 [-0.67, 4.67]
ERTU Ji 2019-2	-1.5	14.4	169	-2.5	12.9	167	1.1%	1.00 [-1.92, 3.92]
IPRA Lu 2016	-9.86	40.29	87	-18.28	49.27	83	0.1%	8.42 [-5.15, 21.99]
SOTA Danne 2018-1	-1.86	12.8	261	0.34	12.75	258	1.4%	-2.20 [-4.40, -0.00]
SOTA Danne 2018-2	-0.33	12.81	263	0.34	12.75	258	1.4%	-0.67 [-2.86, 1.52]
SOTA Garg 2017	-2.14	13.27	699	-1.9	13.23	703	2.0%	-0.24 [-1.63, 1.15]
SOTA Zambrowicz 2012-1	-15	29.31	12	-11	20.02	12	0.0%	-4.00 [-24.08, 16.08]
SOTA Zambrowicz 2012-2	-15	36.48	12	-11	20.02	12	0.0%	-4.00 [-27.54, 19.54]
**Subtotal (95% CI)**			**3602**			**3524**	**20.1%**	**-0.45 [-1.39, 0.50]**
Heterogeneity: Tau² = 2.08; Chi² = 38.18, df = 19 (P = 0.006); I² = 50%								
Test for overall effect: Z = 0.93 (P = 0.35)								
**10.5.2 eGFR 60-89**								
DAPA Araki 2017	-2.4	7.732	122	-5.1	7.746	60	1.3%	2.70 [0.31, 5.09]
DAPA Bailey 2015-1	-1.81	16.82	65	0.48	15.31	75	0.4%	-2.29 [-7.65, 3.07]
DAPA Bailey 2015-2	0.82	13.35	64	0.48	15.31	75	0.5%	0.34 [-4.42, 5.10]
DAPA Bailey 2015-3	2.63	14.36	70	0.48	15.31	75	0.5%	2.15 [-2.68, 6.98]
DAPA Bolinder 2012	2.1	10.38	89	1.9	10.49	91	1.0%	0.20 [-2.85, 3.25]
DAPA Cho 2018	-1.6	20.48	36	-1.3	17.35	35	0.2%	-0.30 [-9.12, 8.52]
DAPA Hayashi 2017	-2.6	25.03	40	-4.3	23.3	40	0.1%	1.70 [-8.90, 12.30]
DAPA Nauck 2011	-0.5	16	400	-5.4	16.02	401	1.4%	4.90 [2.68, 7.12]
DAPA Schumm-Draeger 2015-1	-0.3	10.32	99	0.5	10.53	101	1.1%	-0.80 [-3.69, 2.09]
DAPA Schumm-Draeger 2015-2	-2.1	14.13	100	0.5	10.53	101	0.9%	-2.60 [-6.05, 0.85]
DAPA Schumm-Draeger 2015-3	-3.5	11.76	99	0.5	10.53	101	1.0%	-4.00 [-7.10, -0.90]
DAPA Shimizu 2018	2.9	17.69	33	3.9	25.9	24	0.1%	-1.00 [-12.99, 10.99]
DAPA Strojek 2011-1	-1.5	11.04	154	0	11.32	145	1.3%	-1.50 [-4.04, 1.04]
DAPA Strojek 2011-2	-0.1	12.15	142	0	11.32	145	1.2%	-0.10 [-2.82, 2.62]
DAPA Strojek 2011-3	-1.2	12.66	151	0	11.32	145	1.2%	-1.20 [-3.93, 1.53]
DAPA Weber 2016	-2.1	12.63	225	-1.3	11.45	224	1.4%	-0.80 [-3.03, 1.43]
DAPA Wilding 2009-1	-0.84	14.37	24	-0.58	13.46	23	0.2%	-0.26 [-8.22, 7.70]
DAPA Wilding 2009-2	1.45	20.62	24	-0.58	13.46	23	0.1%	2.03 [-7.89, 11.95]
DAPA Yang 2018	-0.663	12.32	139	-2.154	9.71	133	1.2%	1.49 [-1.14, 4.12]
EMPA Araki 2015-1	2.7	10.7	136	4.9	12	63	0.9%	-2.20 [-5.67, 1.27]
EMPA Araki 2015-2	2.5	9.8	137	4.9	12	63	0.9%	-2.40 [-5.79, 0.99]
EMPA Barnett 2014-1	-2.47	11.7	97	-0.71	9.7	95	1.0%	-1.76 [-4.80, 1.28]
EMPA Barnett 2014-2	-2.04	9.9	98	-0.71	9.7	95	1.1%	-1.33 [-4.10, 1.44]
EMPA Haring 2013-1	-1.3	10.6	225	-1.9	10.1	225	1.6%	0.60 [-1.31, 2.51]
EMPA Haring 2013-2	-2.5	13.4	216	-1.9	10.1	225	1.4%	-0.60 [-2.82, 1.62]
EMPA Haring 2014-1	0.1	13.5	217	1	11.2	207	1.4%	-0.90 [-3.26, 1.46]
EMPA Haring 2014-2	-1.7	10.5	213	1	11.2	207	1.5%	-2.70 [-4.78, -0.62]
EMPA Hattori 2018	-2.2	16.75	51	-0.3	19.34	51	0.3%	-1.90 [-8.92, 5.12]
EMPA Kadowaki 2014-1	-1.27	8.47	110	-1.79	7.73	109	1.5%	0.52 [-1.63, 2.67]
EMPA Kadowaki 2014-2	-1.73	8	109	-1.79	7.73	109	1.5%	0.06 [-2.03, 2.15]
EMPA Kadowaki 2014-3	-0.94	8.18	109	-1.79	7.73	109	1.5%	0.85 [-1.26, 2.96]
EMPA Kadowaki 2014-4	-2.76	11.71	110	-1.79	7.73	109	1.2%	-0.97 [-3.60, 1.66]
EMPA Kovacs 2014-1	-2.1	14.4	165	-0.5	12.5	165	1.1%	-1.60 [-4.51, 1.31]
EMPA Kovacs 2014-2	-3.4	15.6	168	-0.5	12.5	165	1.0%	-2.90 [-5.93, 0.13]
EMPA Lewin 2015-1	1.5	11.4	135	-0.5	10.9	133	1.2%	2.00 [-0.67, 4.67]
EMPA Packer 2020	-0.55	9.927	1863	-2.28	9.938	1867	2.4%	1.73 [1.09, 2.37]
EMPA Roden 2013-1	0.65	12.81	224	-0.02	10.12	228	1.5%	0.67 [-1.46, 2.80]
EMPA Roden 2013-2	1.3	10.77	224	-0.02	10.12	228	1.6%	1.32 [-0.61, 3.25]
EMPA Rosenstock 2014-1	-1.6	11.5	186	-2	11.4	188	1.4%	0.40 [-1.92, 2.72]
EMPA Rosenstock 2014-2	-1.6	11.3	189	-2	11.4	188	1.4%	0.40 [-1.89, 2.69]
EMPA Rosenstock 2015-1	-4.8	12.1	169	-6.3	13	170	1.2%	1.50 [-1.17, 4.17]
EMPA Rosenstock 2015-2	-5.7	13.4	155	-6.3	13	170	1.1%	0.60 [-2.28, 3.48]
EMPA Ross 2015-1	-1.2	13.8	215	-0.3	10.5	107	1.2%	-0.90 [-3.61, 1.81]
EMPA Ross 2015-2	-2.4	10.7	214	-0.3	10.5	107	1.3%	-2.10 [-4.55, 0.35]
EMPA Ross 2015-3	-1.7	10.8	215	-0.3	10.5	107	1.3%	-1.40 [-3.86, 1.06]
EMPA Ross 2015-4	-2	11	214	-0.3	10.5	107	1.3%	-1.70 [-4.18, 0.78]
EMPA Zinman 2015-1	-2.3	12.1	2345	-2	11.5	2333	2.4%	-0.30 [-0.98, 0.38]
EMPA Zinman 2015-2	-2.9	11.8	2342	-2	11.5	2333	2.4%	-0.90 [-1.57, -0.23]
IPRA Bando 2016	1.6	20.03	40	6.3	20.26	22	0.1%	-4.70 [-15.20, 5.80]
IPRA Han 2018	-0.17	10.54	73	0.98	10.76	66	0.8%	-1.15 [-4.70, 2.40]
IPRA Ishihara 2016	-1.19	9.53	168	1.21	8.19	87	1.4%	-2.40 [-4.64, -0.16]
IPRA Kashiwagi 2015	0.2	6.56	118	1.5	6	46	1.5%	-1.30 [-3.40, 0.80]
IPRA Tsurutani 2018	-1.1	7.074	60	-1.7	7.054	59	1.3%	0.60 [-1.94, 3.14]
LUSEO Seino 2018	-1	9.007	159	0.3	8.997	74	1.3%	-1.30 [-3.78, 1.18]
SOTA Buse 2018-1	-2.46	11.56	263	-1.06	11.82	268	1.6%	-1.40 [-3.39, 0.59]
SOTA Buse 2018-2	-2.16	11.69	262	-1.06	11.82	268	1.6%	-1.10 [-3.10, 0.90]
TOFO Kaku 2014-1	0.41	8.48	57	2.53	9.1	56	0.9%	-2.12 [-5.36, 1.12]
TOFO Kaku 2014-2	1.23	12.89	58	2.53	9.1	56	0.7%	-1.30 [-5.38, 2.78]
TOFO Kaku 2014-3	-1.97	7.97	58	2.53	9.1	56	1.0%	-4.50 [-7.64, -1.36]
**Subtotal (95% CI)**			**14243**			**13338**	**67.0%**	**-0.46 [-0.93, 0.00]**
Heterogeneity: Tau² = 1.34; Chi² = 126.33, df = 58 (P < 0.00001); I² = 54%								
Test for overall effect: Z = 1.96 (P = 0.05)								
**10.5.3 eGFR 45-59**								
CANA Takashima 2018	0.7	6.4	21	-3.4	4.5	21	0.9%	4.10 [0.75, 7.45]
EMPA Barnett 2014-3	-2.8	8.2	187	-0.3	7.4	187	1.8%	-2.50 [-4.08, -0.92]
LUSEO Haneda 2016	-1.5	7.459	95	1	3.608	50	1.7%	-2.50 [-4.30, -0.70]
**Subtotal (95% CI)**			**303**			**258**	**4.4%**	**-0.68 [-3.80, 2.45]**
Heterogeneity: Tau² = 6.28; Chi² = 13.27, df = 2 (P = 0.001); I² = 85%								
Test for overall effect: Z = 0.42 (P = 0.67)								
**10.5.4 eGFR <45**								
DAPA Heerspink 2020	-2.86	5.099	2149	-3.79	5.099	2149	2.6%	0.93 [0.63, 1.23]
DAPA Kohan 2013-1	-1.71	11.21	83	-2.38	9.257	84	1.0%	0.67 [-2.45, 3.79]
DAPA Kohan 2013-2	-3.5	9.404	85	-2.38	9.257	84	1.1%	-1.12 [-3.93, 1.69]
EMPA Barnett 2014-4	-1.4	6	37	-1.1	5.8	37	1.2%	-0.30 [-2.99, 2.39]
SOTA Bhatt 2021	-1.91	6.866	5292	-0.66	6.866	5292	2.6%	-1.25 [-1.51, -0.99]
**Subtotal (95% CI)**			**7646**			**7646**	**8.4%**	**-0.21 [-1.77, 1.34]**
Heterogeneity: Tau² = 2.21; Chi² = 113.81, df = 4 (P < 0.00001); I² = 96%								
Test for overall effect: Z = 0.27 (P = 0.79)								
**Total (95% CI)**			**25794**			**24766**	**100.0%**	**-0.48 [-0.87, -0.08]**
Heterogeneity: Tau² = 1.56; Chi² = 299.95, df = 86 (P < 0.00001); I² = 71%								
Test for overall effect: Z = 2.37 (P = 0.02)								
Test for subgroup differences: Chi² = 0.11, df = 3 (P = 0.99), I² = 0%								

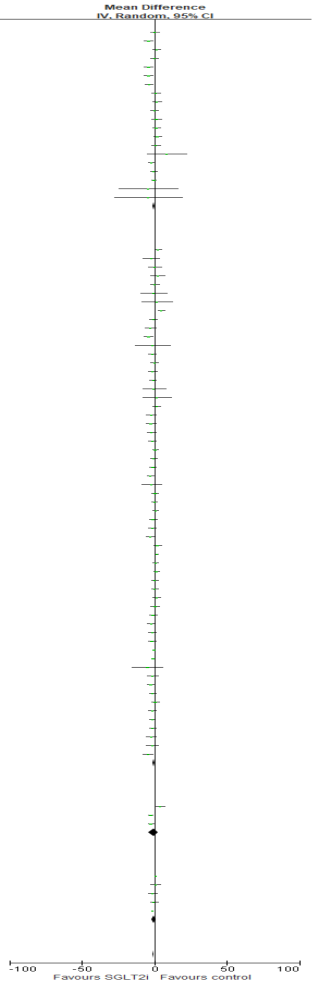

Figure S7. The associations between the use of SGLT2i treatment and urinary tract infection.

Study or Subgroup	SGLT2i Events	SGLT2i Total	Control Events	Control Total	Weight	Odds Ratio M-H, Fixed, 95% CI
**19.2.1 eGFR >=90**						
CANA Ji 2015	13	450	6	226	0.4%	1.09 [0.41, 2.91]
CANA Leiter 2014	93	968	33	482	2.2%	1.45 [0.96, 2.19]
CANA Wilding 2013	26	313	12	156	0.8%	1.09 [0.53, 2.22]
DAPA Henry 2018	3	50	3	50	0.2%	1.00 [0.19, 5.21]
DAPA Jabbour 2018	16	231	13	230	0.7%	1.24 [0.58, 2.64]
DAPA Jeon 2018	3	162	0	148	0.0%	6.52 [0.33, 127.24]
DAPA Ji 2014	12	261	4	132	0.3%	1.54 [0.49, 4.88]
DAPA Mathieu 2015	8	160	10	160	0.5%	0.79 [0.30, 2.06]
DAPA Rosenstock 2015	1	179	9	176	0.5%	0.10 [0.01, 0.83]
DAPA Wilding 2012	59	610	10	197	0.8%	2.00 [1.00, 3.99]
EMPA Ferdinand 2019	4	80	4	77	0.2%	0.96 [0.23, 3.98]
EMPA Ferrannini 2013-1	11	215	2	56	0.2%	1.46 [0.31, 6.77]
EMPA Ferrannini 2013-2	36	332	7	56	0.6%	0.85 [0.36, 2.02]
EMPA Hadjadj 2016-1	29	339	14	171	0.9%	1.05 [0.54, 2.04]
EMPA Hadjadj 2016-2	34	341	17	170	1.1%	1.00 [0.54, 1.84]
EMPA Lewin 2015-2	17	136	14	135	0.7%	1.23 [0.58, 2.62]
ERTU Ji 2019	5	339	4	167	0.3%	0.61 [0.16, 2.30]
ERTU Miller 2018	11	194	5	97	0.3%	1.11 [0.37, 3.28]
ERTU Pratley 2018	17	487	8	247	0.6%	1.08 [0.46, 2.54]
ERTU Rosenstock 2018-1	7	205	2	209	0.1%	3.66 [0.75, 17.83]
IPRA Inoue 2019	4	24	3	24	0.1%	1.40 [0.28, 7.06]
IPRA Lu 2016	6	87	2	83	0.1%	3.00 [0.59, 15.31]
SOTA Danne 2018	29	524	13	258	0.9%	1.10 [0.56, 2.16]
SOTA Garg 2017	25	699	27	703	1.4%	0.93 [0.53, 1.62]
**Subtotal (95% CI)**		**7386**		**4410**	**14.0%**	**1.18 [0.99, 1.39]**
Total events	469		222			
Heterogeneity: Chi² = 16.86, df = 23 (P = 0.82); I² = 0%						
Test for overall effect: Z = 1.87 (P = 0.06)						
**19.2.2 eGFR 60-89**						
CANA Bode 2015	74	477	24	137	1.7%	0.86 [0.52, 1.43]
CANA Inagaki 2014	2	178	1	93	0.1%	1.05 [0.09, 11.68]
CANA Inagaki 2016	1	75	0	71	0.0%	2.88 [0.12, 71.85]
CANA Kadowaki 2017	0	70	1	68	0.1%	0.32 [0.01, 7.97]
CANA Lavalle-González 2013-1	1	368	0	183	0.0%	1.50 [0.06, 36.95]
CANA Rosenstock 2016	10	474	3	237	0.2%	1.68 [0.46, 6.17]
CANA Schernthaner 2013	15	377	21	378	1.1%	0.70 [0.36, 1.39]
CANA Stenlof 2013	24	392	8	192	0.6%	1.50 [0.66, 3.40]
DAPA Araki 2017	2	123	3	60	0.2%	0.31 [0.05, 1.93]
DAPA Bailey 2015	18	199	3	75	0.2%	2.39 [0.68, 8.35]
DAPA Bolinder 2012	6	91	2	91	0.1%	3.14 [0.62, 15.99]
DAPA Müller-Wieland 2018	37	625	12	312	0.8%	1.57 [0.81, 3.06]
DAPA Nauck 2011	44	406	26	408	1.3%	1.79 [1.08, 2.96]
DAPA Schumm-Draeger 2015	10	299	3	101	0.2%	1.13 [0.30, 4.19]
DAPA Strojek 2011	24	450	9	146	0.7%	0.86 [0.39, 1.89]
DAPA Weber 2016	4	225	2	224	0.1%	2.01 [0.36, 11.08]
DAPA Wilding 2009	1	48	0	23	0.0%	1.48 [0.06, 37.84]
DAPA Yang 2018	5	139	7	133	0.4%	0.67 [0.21, 2.17]
EMPA Araki 2015	12	273	2	63	0.2%	1.40 [0.31, 6.43]
EMPA Barnett 2014-1	23	195	15	95	1.0%	0.71 [0.35, 1.44]
EMPA Haring 2013	41	441	18	225	1.2%	1.18 [0.66, 2.10]
EMPA Haring 2014	23	430	10	207	0.7%	1.11 [0.52, 2.38]
EMPA Kadowaki 2014	3	438	1	109	0.1%	0.74 [0.08, 7.23]
EMPA Kawamori 2018	9	182	5	93	0.3%	0.92 [0.30, 2.81]
EMPA Kovacs 2014	48	333	27	165	1.7%	0.86 [0.52, 1.44]
EMPA Lewin 2015-1	21	136	14	135	0.7%	1.58 [0.77, 3.25]
EMPA Packer 2020	91	1863	83	1863	4.4%	1.10 [0.81, 1.49]
EMPA Roden 2013	27	447	12	229	0.8%	1.16 [0.58, 2.34]
EMPA Rosenstock 2014	58	375	29	188	1.8%	1.00 [0.62, 1.63]
EMPA Rosenstock 2015	43	324	15	170	0.9%	1.58 [0.85, 2.94]
EMPA Ross 2015	63	876	4	107	0.4%	2.00 [0.71, 5.60]
EMPA Zinman 2015	842	4687	423	2333	25.6%	0.99 [0.87, 1.13]
ERTU Dagogo-Jack 2018	16	309	10	153	0.7%	0.78 [0.35, 1.76]
ERTU Hollander 2019	79	880	36	435	2.4%	1.09 [0.72, 1.65]
ERTU Rosenstock 2018-1	6	207	2	209	0.1%	3.09 [0.62, 15.49]
ERTU Terra 2017	17	308	13	153	0.9%	0.63 [0.30, 1.33]
IPRA Han 2018	2	74	1	68	0.1%	1.86 [0.16, 21.00]
IPRA Ishihara 2016	4	175	1	87	0.1%	2.01 [0.22, 18.28]
IPRA Kashiwagi 2015	1	119	2	46	0.2%	0.19 [0.02, 2.11]
IPRA Tsurutani 2018	3	60	0	59	0.0%	7.24 [0.37, 143.36]
SOTA Buse 2018	37	525	19	268	1.3%	0.99 [0.56, 1.76]
TOFO Kaku 2014	1	174	0	56	0.0%	0.98 [0.04, 24.32]
TOFO Terauchi 2017	0	140	1	70	0.1%	0.16 [0.01, 4.10]
**Subtotal (95% CI)**		**18987**		**10518**	**53.6%**	**1.06 [0.97, 1.15]**
Total events	1748		868			
Heterogeneity: Chi² = 33.38, df = 42 (P = 0.83); I² = 0%						
Test for overall effect: Z = 1.23 (P = 0.22)						
**19.2.3 eGFR 45-59**						
DAPA Fioretto 2018	4	160	6	161	0.3%	0.66 [0.18, 2.39]
DAPA Pollock 2019	5	145	4	148	0.2%	1.29 [0.34, 4.89]
EMPA Barnett 2014-3	31	187	29	187	1.3%	1.08 [0.62, 1.88]
ERTU Grunberger 2017	13	313	11	154	0.8%	0.56 [0.25, 1.29]
LUSEO Haneda 2016	2	95	0	50	0.0%	2.70 [0.13, 57.34]
**Subtotal (95% CI)**		**900**		**700**	**2.7%**	**0.92 [0.61, 1.38]**
Total events	55		50			
Heterogeneity: Chi² = 2.65, df = 4 (P = 0.62); I² = 0%						
Test for overall effect: Z = 0.41 (P = 0.68)						
**19.2.4 eGFR <45**						
CANA Yale 2013	12	179	5	90	0.3%	1.22 [0.42, 3.58]
DAPA Kohan 2013	23	168	12	84	0.8%	0.95 [0.45, 2.02]
EMPA Barnett 2014-4	7	37	3	37	0.1%	2.64 [0.63, 11.15]
SOTA Bhatt 2021	610	5291	585	5286	28.6%	1.05 [0.93, 1.18]
**Subtotal (95% CI)**		**5675**		**5497**	**29.8%**	**1.05 [0.94, 1.19]**
Total events	652		605			
Heterogeneity: Chi² = 1.72, df = 3 (P = 0.63); I² = 0%						
Test for overall effect: Z = 0.87 (P = 0.38)						
**Total (95% CI)**		**32948**		**21125**	**100.0%**	**1.07 [1.00, 1.14]**
Total events	2924		1745			
Heterogeneity: Chi² = 56.56, df = 75 (P = 0.94); I² = 0%						
Test for overall effect: Z = 2.03 (P = 0.04)						
Test for subgroup differences: Chi² = 1.87, df = 3 (P = 0.60), I² = 0%						

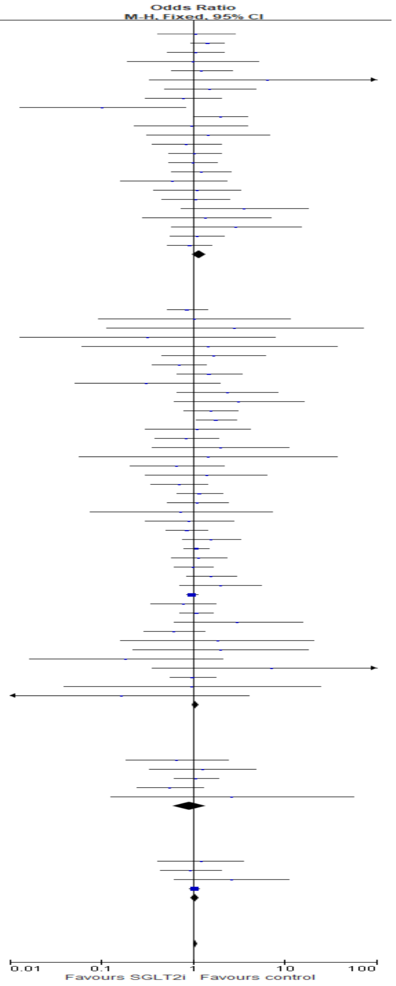

Figure S8. The associations between the use of SGLT2i treatment and genital tract infection.

Study or Subgroup	SGLT2i Events	SGLT2i Total	Control Events	Control Total	Weight	Odds Ratio M-H, Fixed, 95% CI
**19.3.5 eGFR >=90**						
CANA Ji 2015	7	450	2	226	0.8%	1.77 [0.36, 8.59]
CANA Leiter 2014	116	968	11	482	4.1%	5.83 [3.11, 10.93]
CANA Wilding 2013	39	313	5	156	1.9%	4.30 [1.66, 11.14]
DAPA Jabbour 2018	11	231	4	230	1.2%	2.83 [0.89, 9.01]
DAPA Ji 2014	10	261	1	132	0.4%	5.22 [0.66, 41.21]
DAPA Mathieu 2015	8	160	1	160	0.3%	8.37 [1.03, 67.71]
DAPA Rosenstock 2015	0	179	1	176	0.5%	0.33 [0.01, 8.05]
DAPA Wilding 2012	55	610	5	197	2.2%	3.81 [1.50, 9.65]
EMPA Ferdinand 2019	4	80	1	77	0.3%	4.00 [0.44, 36.62]
EMPA Ferrannini 2013-1	11	215	1	56	0.5%	2.97 [0.37, 23.47]
EMPA Ferrannini 2013-2	11	332	0	56	0.3%	4.04 [0.23, 69.56]
EMPA Hadjadj 2016-1	12	339	4	171	1.6%	1.53 [0.49, 4.82]
EMPA Hadjadj 2016-2	10	341	5	170	2.1%	1.00 [0.34, 2.96]
EMPA Lewin 2015-2	8	136	4	135	1.2%	2.05 [0.60, 6.97]
ERTU Ji 2019	7	339	2	167	0.8%	1.74 [0.36, 8.47]
ERTU Miller 2018	9	194	2	97	0.8%	2.31 [0.49, 10.91]
ERTU Pratley 2018	23	487	1	247	0.4%	12.19 [1.64, 90.83]
ERTU Rosenstock 2018-1	10	205	1	209	0.3%	10.67 [1.35, 84.10]
IPRA Inoue 2019	1	24	0	24	0.2%	3.13 [0.12, 80.68]
SOTA Danne 2018	53	524	6	258	2.3%	4.73 [2.00, 11.15]
SOTA Garg 2017	45	699	15	703	4.5%	3.16 [1.74, 5.72]
**Subtotal (95% CI)**		**7087**		**4129**	**26.8%**	**3.67 [2.85, 4.73]**
Total events	450		72			
Heterogeneity: Chi^2 = 18.94, df = 20 (P = 0.53); I^2 = 0%						
Test for overall effect: Z = 10.04 (P < 0.00001)						
**19.3.6 eGFR 60-89**						
CANA Bode 2015	67	477	6	137	2.6%	3.57 [1.51, 8.42]
CANA Inagaki 2013	2	307	0	75	0.3%	1.24 [0.06, 26.01]
CANA Inagaki 2014	3	178	1	93	0.4%	1.58 [0.16, 15.38]
CANA Inagaki 2016	1	75	0	71	0.2%	2.88 [0.12, 71.85]
CANA Rosenstock 2016	10	474	0	237	0.2%	10.74 [0.63, 184.02]
CANA Schernthaner 2013	45	377	8	378	2.3%	6.27 [2.91, 13.49]
CANA Stenlof 2013	25	392	4	192	1.6%	3.20 [1.10, 9.33]
DAPA Araki 2017	1	123	1	60	0.4%	0.48 [0.03, 7.87]
DAPA Bailey 2015	24	199	1	75	0.4%	10.15 [1.35, 76.41]
DAPA Bolinder 2012	3	91	0	91	0.2%	7.24 [0.37, 142.14]
DAPA Müller-Wieland 2018	40	625	2	312	0.8%	10.60 [2.54, 44.15]
DAPA Nauck 2011	50	406	11	408	3.1%	5.07 [2.60, 9.89]
DAPA Schumm-Draeger 2015	8	299	1	101	0.5%	2.75 [0.34, 22.25]
DAPA Strojek 2011	25	450	1	146	0.5%	8.53 [1.15, 63.51]
DAPA Weber 2016	6	225	4	224	1.3%	1.51 [0.42, 5.41]
DAPA Wilding 2009	5	48	1	23	0.4%	2.56 [0.28, 23.26]
DAPA Yang 2018	3	139	0	133	0.2%	6.85 [0.35, 133.82]
EMPA Araki 2015	2	273	1	63	0.5%	0.46 [0.04, 5.13]
EMPA Barnett 2014-1	12	195	6	95	2.4%	0.97 [0.35, 2.68]
EMPA Haring 2013	11	441	2	225	0.8%	2.85 [0.63, 12.98]
EMPA Haring 2014	18	430	0	207	0.2%	18.61 [1.12, 310.37]
EMPA Kadowaki 2014	3	438	0	109	0.3%	1.76 [0.09, 34.33]
EMPA Kario 2018	1	68	0	63	0.2%	2.82 [0.11, 70.56]
EMPA Kawamori 2018	2	182	0	93	0.2%	2.59 [0.12, 54.50]
EMPA Kovacs 2014	20	333	4	165	1.6%	2.57 [0.86, 7.65]
EMPA Lewin 2015-1	4	136	4	135	1.2%	0.99 [0.24, 4.05]
EMPA Packer 2020	31	1863	12	1863	3.8%	2.61 [1.34, 5.10]
EMPA Roden 2013	16	447	0	229	0.2%	17.55 [1.05, 293.88]
EMPA Rosenstock 2014	26	375	3	188	1.2%	4.59 [1.37, 15.38]
EMPA Rosenstock 2015	21	324	3	170	1.2%	3.86 [1.13, 13.13]
EMPA Ross 2015	33	876	3	107	1.6%	1.36 [0.41, 4.50]
EMPA Zinman 2015	301	4687	42	2333	16.8%	3.74 [2.70, 5.19]
ERTU Dagogo-Jack 2018	26	309	1	153	0.4%	13.96 [1.88, 103.91]
ERTU Hollander 2019	67	880	3	435	1.2%	11.87 [3.71, 37.95]
ERTU Rosenstock 2018-1	9	207	1	209	0.3%	9.45 [1.19, 75.31]
ERTU Terra 2017	33	308	5	153	1.9%	3.55 [1.36, 9.29]
IPRA Ishihara 2016	7	175	0	87	0.2%	7.79 [0.44, 137.98]
IPRA Kashiwagi 2015	1	119	0	46	0.2%	1.18 [0.05, 29.42]
IPRA Tsurutani 2018	1	60	0	59	0.2%	3.00 [0.12, 75.14]
LUSEO Seino 2018	4	159	0	74	0.2%	4.31 [0.23, 81.14]
SOTA Buse 2018	58	525	9	268	3.4%	3.57 [1.74, 7.33]
TOFO Kaku 2014	1	174	0	56	0.2%	0.98 [0.04, 24.32]
TOFO Terauchi 2017	1	149	0	70	0.2%	1.42 [0.06, 35.40]
**Subtotal (95% CI)**		**19018**		**10411**	**55.8%**	**3.95 [3.31, 4.71]**
Total events	1027		140			
Heterogeneity: Chi^2 = 41.86, df = 42 (P = 0.48); I^2 = 0%						
Test for overall effect: Z = 15.27 (P < 0.00001)						
**19.3.7 eGFR 45-59**						
DAPA Fioretto 2018	3	160	2	161	0.6%	1.52 [0.25, 9.22]
DAPA Pollock 2019	4	145	0	148	0.2%	9.45 [0.50, 177.03]
EMPA Barnett 2014-3	5	187	2	187	0.6%	2.54 [0.49, 13.27]
ERTU Grunberger 2017	7	313	0	154	0.2%	7.56 [0.43, 133.25]
LUSEO Haneda 2016	1	95	0	50	0.2%	1.60 [0.06, 40.08]
**Subtotal (95% CI)**		**900**		**700**	**1.8%**	**3.24 [1.24, 8.46]**
Total events	20		4			
Heterogeneity: Chi^2 = 1.79, df = 4 (P = 0.77); I^2 = 0%						
Test for overall effect: Z = 2.41 (P = 0.02)						
**19.3.8 eGFR <45**						
CANA Yale 2013	4	179	0	90	0.2%	4.64 [0.25, 87.15]
DAPA Kohan 2013	15	168	3	84	1.2%	2.65 [0.74, 9.41]
EMPA Barnett 2014-4	1	37	0	37	0.2%	3.08 [0.12, 78.14]
SOTA Bhatt 2021	125	5291	45	5286	14.1%	2.82 [2.00, 3.97]
**Subtotal (95% CI)**		**5675**		**5497**	**15.6%**	**2.83 [2.04, 3.93]**
Total events	145		48			
Heterogeneity: Chi^2 = 0.12, df = 3 (P = 0.99); I^2 = 0%						
Test for overall effect: Z = 6.23 (P < 0.00001)						
**Total (95% CI)**		**32680**		**20737**	**100.0%**	**3.69 [3.23, 4.20]**
Total events	1642		264			
Heterogeneity: Chi^2 = 64.38, df = 72 (P = 0.73); I^2 = 0%						
Test for overall effect: Z = 19.52 (P < 0.00001)						
Test for subgroup differences: Chi^2 = 3.13, df = 3 (P = 0.37), I^2 = 4.2%						

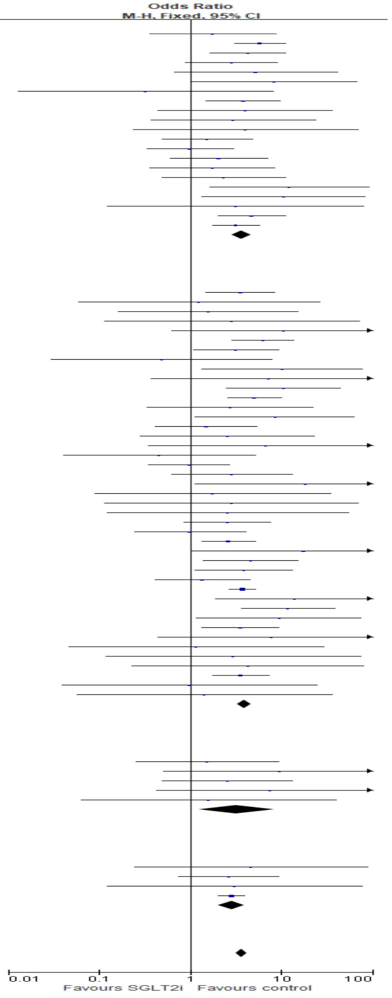

Figure S9. The associations between the use of SGLT2i treatment and amputation.

Study or Subgroup	SGLT2i Events	SGLT2i Total	Control Events	Control Total	Weight	Odds Ratio M-H, Fixed, 95% CI
**19.4.5 eGFR >=90**						
SOTA Danne 2018	1	524	0	258	0.7%	1.48 [0.06, 36.49]
**Subtotal (95% CI)**		**524**		**258**	**0.7%**	**1.48 [0.06, 36.49]**
Total events	1		0			
Heterogeneity: Not applicable						
Test for overall effect: Z = 0.24 (P = 0.81)						
**19.4.6 eGFR 60-89**						
DAPA McMurray 2019	13	2373	12	2371	12.6%	1.08 [0.49, 2.38]
EMPA Packer 2020	13	1863	10	1863	10.5%	1.30 [0.57, 2.98]
SOTA Buse 2018	1	525	0	268	0.7%	1.54 [0.06, 37.83]
**Subtotal (95% CI)**		**4761**		**4502**	**23.7%**	**1.19 [0.68, 2.09]**
Total events	27		22			
Heterogeneity: Chi² = 0.13, df = 2 (P = 0.94); I² = 0%						
Test for overall effect: Z = 0.62 (P = 0.54)						
**19.4.7 eGFR 45-59**						
DAPA Pollock 2019	1	145	0	148	0.5%	3.08 [0.12, 76.30]
**Subtotal (95% CI)**		**145**		**148**	**0.5%**	**3.08 [0.12, 76.30]**
Total events	1		0			
Heterogeneity: Not applicable						
Test for overall effect: Z = 0.69 (P = 0.49)						
**19.4.8 eGFR <45**						
DAPA Heerspink 2020	35	2149	39	2149	40.4%	0.90 [0.57, 1.42]
SOTA Bhatt 2021	32	5291	33	5286	34.6%	0.97 [0.59, 1.58]
**Subtotal (95% CI)**		**7440**		**7435**	**75.0%**	**0.93 [0.66, 1.30]**
Total events	67		72			
Heterogeneity: Chi² = 0.05, df = 1 (P = 0.82); I² = 0%						
Test for overall effect: Z = 0.43 (P = 0.67)						
**Total (95% CI)**		**12870**		**12343**	**100.0%**	**1.01 [0.76, 1.34]**
Total events	96		94			
Heterogeneity: Chi² = 1.27, df = 6 (P = 0.97); I² = 0%						
Test for overall effect: Z = 0.05 (P = 0.96)						
Test for subgroup differences: Chi² = 1.09, df = 3 (P = 0.78), I² = 0%						

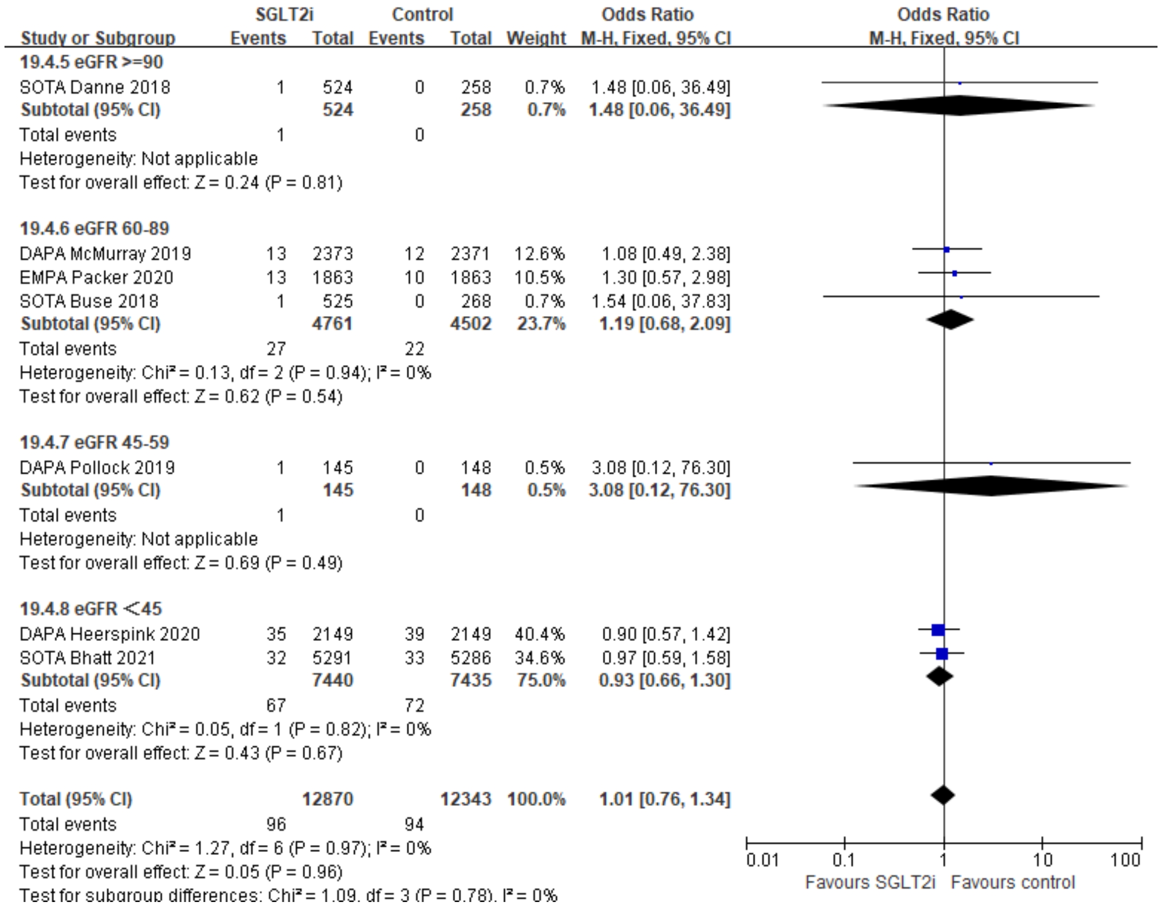

Figure S10. The associations between the use of SGLT2i treatment and hypovolemia.

Study or Subgroup	SGLT2i Events	SGLT2i Total	Control Events	Control Total	Weight	Odds Ratio M-H, Fixed, 95% CI
**19.5.5 eGFR >=90**						
CANA Ji 2015	1	450	0	226	0.1%	1.51 [0.06, 37.26]
CANA Leiter 2014	20	968	11	482	1.7%	0.90 [0.43, 1.90]
CANA Wilding 2013	7	313	3	156	0.5%	1.17 [0.30, 4.57]
DAPA Jabbour 2018	2	231	1	230	0.1%	2.00 [0.18, 22.21]
DAPA Wilding 2012	13	610	2	197	0.3%	2.12 [0.47, 9.49]
EMPA Hadjadj 2016-1	2	339	0	171	0.1%	2.54 [0.12, 53.22]
EMPA Hadjadj 2016-2	4	341	2	170	0.3%	1.00 [0.18, 5.50]
EMPA Lewin 2015-2	1	136	0	135	0.1%	3.00 [0.12, 74.30]
ERTU Ji 2019	1	339	1	167	0.2%	0.49 [0.03, 7.90]
ERTU Miller 2018	4	194	1	97	0.2%	2.02 [0.22, 18.33]
ERTU Rosenstock 2018-1	2	205	1	209	0.1%	2.05 [0.18, 22.78]
IPRA Inoue 2019	4	24	3	24	0.3%	1.40 [0.28, 7.06]
SOTA Danne 2018	8	524	1	258	0.2%	3.98 [0.50, 32.03]
SOTA Garg 2017	13	699	2	703	0.2%	6.64 [1.49, 29.54]
**Subtotal (95% CI)**		**5373**		**3225**	**4.2%**	**1.65 [1.08, 2.53]**
Total events	82		28			
Heterogeneity: Chi^2 = 8.32, df = 13 (P = 0.82); I^2 = 0%						
Test for overall effect: Z = 2.32 (P = 0.02)						
**19.5.6 eGFR 60-89**						
CANA Bode 2015	27	477	4	137	0.7%	2.00 [0.69, 5.80]
CANA Inagaki 2014	2	178	1	93	0.2%	1.05 [0.09, 11.68]
CANA Rosenstock 2016	6	474	1	237	0.2%	3.03 [0.36, 25.28]
CANA Schernthaner 2013	0	377	3	378	0.4%	0.14 [0.01, 2.76]
CANA Stenlof 2013	5	392	0	192	0.1%	5.46 [0.30, 99.33]
DAPA Araki 2017	3	123	1	60	0.2%	1.48 [0.15, 14.49]
DAPA Bailey 2015	1	199	1	75	0.2%	0.37 [0.02, 6.05]
DAPA Bolinder 2012	1	91	0	91	0.1%	3.03 [0.12, 75.44]
DAPA McMurray 2019	178	2373	162	2371	17.5%	1.11 [0.89, 1.38]
DAPA Nauck 2011	6	406	3	408	0.3%	2.02 [0.50, 8.15]
DAPA Scott 2018	3	306	2	307	0.2%	1.51 [0.25, 9.10]
DAPA Strojek 2011	2	450	0	146	0.1%	1.63 [0.08, 34.21]
DAPA Weber 2016	1	225	0	224	0.1%	3.00 [0.12, 74.04]
DAPA Yang 2018	1	139	0	133	0.1%	2.89 [0.12, 71.61]
EMPA Araki 2015	6	273	0	63	0.1%	3.09 [0.17, 55.50]
EMPA Barnett 2014-1	1	195	1	95	0.2%	0.48 [0.03, 7.83]
EMPA Kadowaki 2014	2	438	1	109	0.2%	0.50 [0.04, 5.51]
EMPA Kawamori 2018	1	182	1	93	0.2%	0.51 [0.03, 8.22]
EMPA Lewin 2015-1	3	136	0	135	0.1%	7.10 [0.36, 138.88]
EMPA Packer 2020	197	1863	184	1863	19.2%	1.08 [0.87, 1.33]
EMPA Ross 2015	3	876	0	107	0.1%	0.86 [0.04, 16.79]
EMPA Zinman 2015	239	4687	115	2333	17.0%	1.04 [0.82, 1.30]
ERTU Dagogo-Jack 2018	1	309	2	153	0.3%	0.25 [0.02, 2.72]
ERTU Hollander 2019	16	880	3	435	0.5%	2.67 [0.77, 9.20]
ERTU Rosenstock 2018-1	1	207	1	209	0.1%	1.01 [0.06, 16.25]
ERTU Terra 2017	5	308	6	153	0.9%	0.40 [0.12, 1.35]
LUSEO Seino 2018	7	159	1	74	0.2%	3.36 [0.41, 27.83]
SOTA Buse 2018	12	525	4	268	0.6%	1.54 [0.49, 4.83]
TOFO Terauchi 2017	7	140	1	70	0.1%	3.63 [0.44, 30.12]
**Subtotal (95% CI)**		**17388**		**11012**	**59.8%**	**1.12 [0.99, 1.26]**
Total events	737		498			
Heterogeneity: Chi^2 = 19.92, df = 28 (P = 0.87); I^2 = 0%						
Test for overall effect: Z = 1.85 (P = 0.06)						
**19.5.7 eGFR 45-59**						
DAPA Fioretto 2018	3	160	0	161	0.1%	7.18 [0.37, 140.09]
DAPA Pollock 2019	4	145	4	148	0.4%	1.02 [0.25, 4.16]
EMPA Barnett 2014-3	7	187	5	187	0.6%	1.42 [0.44, 4.54]
ERTU Grunberger 2017	10	313	0	154	0.1%	10.69 [0.62, 183.64]
LUSEO Haneda 2016	0	95	2	50	0.4%	0.10 [0.00, 2.16]
**Subtotal (95% CI)**		**900**		**700**	**1.5%**	**1.65 [0.82, 3.30]**
Total events	24		11			
Heterogeneity: Chi^2 = 6.31, df = 4 (P = 0.18); I^2 = 37%						
Test for overall effect: Z = 1.41 (P = 0.16)						
**19.5.8 eGFR <45**						
CANA Yale 2013	4	179	0	90	0.1%	4.64 [0.25, 87.15]
DAPA Heerspink 2020	127	2149	90	2149	9.9%	1.44 [1.09, 1.90]
DAPA Kohan 2013	19	168	5	84	0.7%	2.01 [0.72, 5.60]
EMPA Barnett 2014-4	2	37	2	37	0.2%	1.00 [0.13, 7.50]
SOTA Bhatt 2021	278	5291	213	5286	23.6%	1.32 [1.10, 1.59]
**Subtotal (95% CI)**		**7824**		**7646**	**34.4%**	**1.37 [1.18, 1.60]**
Total events	430		310			
Heterogeneity: Chi^2 = 1.58, df = 4 (P = 0.81); I^2 = 0%						
Test for overall effect: Z = 4.15 (P < 0.0001)						
**Total (95% CI)**		**31485**		**22583**	**100.0%**	**1.24 [1.13, 1.35]**
Total events	1273		847			
Heterogeneity: Chi^2 = 41.67, df = 52 (P = 0.85); I^2 = 0%						
Test for overall effect: Z = 4.62 (P < 0.00001)						
Test for subgroup differences: Chi^2 = 6.97, df = 3 (P = 0.07), I^2 = 57.0%						

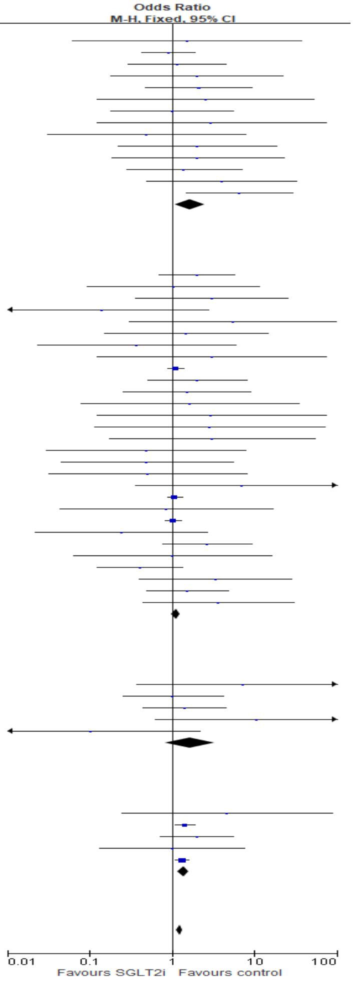

Figure S11. The associations between the use of SGLT2i treatment and orthostatic hypotension.

Study or Subgroup	SGLT2i Events	SGLT2i Total	Control Events	Control Total	Weight	Odds Ratio M-H, Fixed, 95% CI
**19.6.5 eGFR >=90**						
EMPA Hadjadj 2016-2	2	341	0	170	5.3%	2.51 [0.12, 52.60]
IPRA Inoue 2019	1	24	0	24	3.8%	3.13 [0.12, 80.68]
**Subtotal (95% CI)**		**365**		**194**	**9.1%**	**2.77 [0.30, 25.75]**
Total events	3		0			
Heterogeneity: Chi² = 0.01, df = 1 (P = 0.92); I² = 0%						
Test for overall effect: Z = 0.89 (P = 0.37)						
**19.6.6 eGFR 60-89**						
CANA Schernthaner 2013	0	377	1	378	12.0%	0.33 [0.01, 8.21]
CANA Stenlof 2013	2	392	0	192	5.3%	2.46 [0.12, 51.59]
DAPA Araki 2017	0	123	1	60	16.0%	0.16 [0.01, 4.00]
DAPA Bailey 2015	3	199	1	75	11.4%	1.13 [0.12, 11.06]
DAPA Cho 2018	1	36	0	35	3.9%	3.00 [0.12, 76.16]
DAPA Weber 2016	7	225	4	224	31.1%	1.77 [0.51, 6.12]
TOFO Kaku 2014	1	174	0	56	6.0%	0.98 [0.04, 24.32]
**Subtotal (95% CI)**		**1526**		**1020**	**85.7%**	**1.23 [0.54, 2.76]**
Total events	14		7			
Heterogeneity: Chi² = 3.02, df = 6 (P = 0.81); I² = 0%						
Test for overall effect: Z = 0.49 (P = 0.62)						
**19.6.8 eGFR <45**						
CANA Yale 2013	1	179	0	90	5.3%	1.52 [0.06, 37.71]
**Subtotal (95% CI)**		**179**		**90**	**5.3%**	**1.52 [0.06, 37.71]**
Total events	1		0			
Heterogeneity: Not applicable						
Test for overall effect: Z = 0.26 (P = 0.80)						
**Total (95% CI)**		**2070**		**1304**	**100.0%**	**1.38 [0.66, 2.89]**
Total events	18		7			
Heterogeneity: Chi² = 3.46, df = 9 (P = 0.94); I² = 0%						
Test for overall effect: Z = 0.86 (P = 0.39)						
Test for subgroup differences: Chi² = 0.46, df = 2 (P = 0.80), I² = 0%						

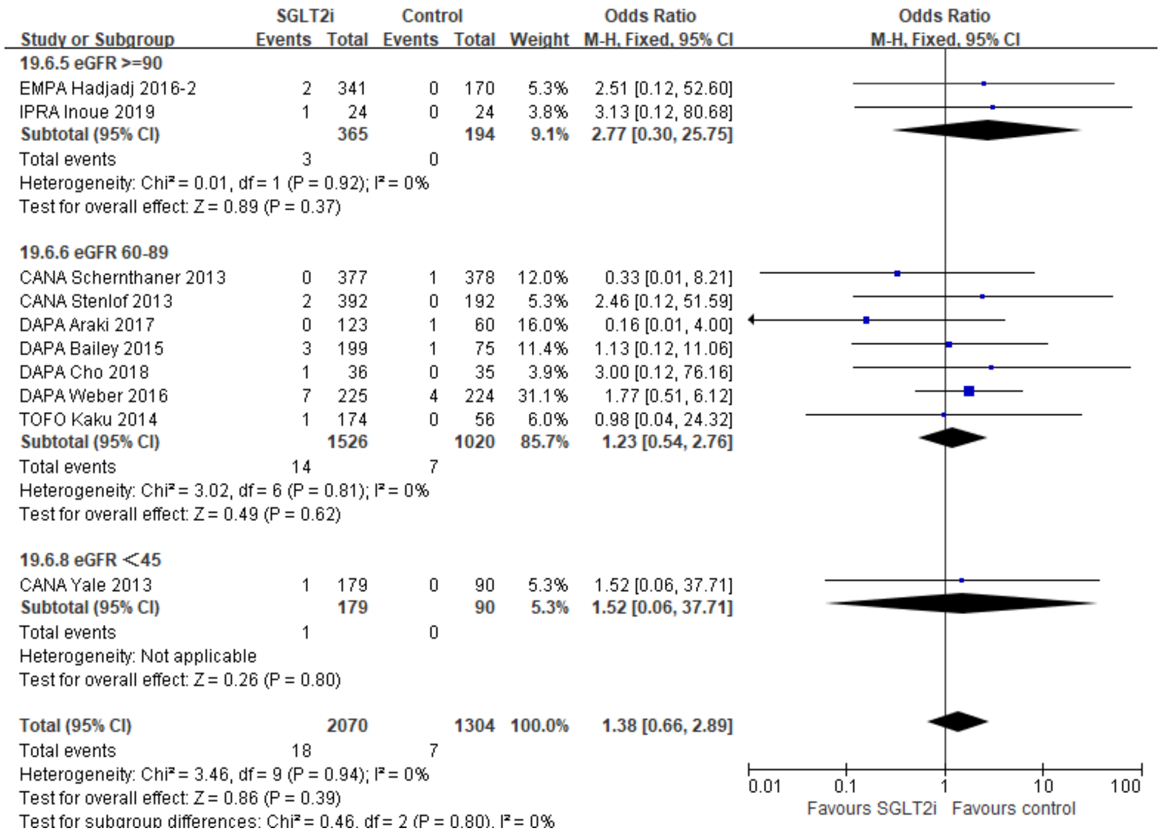

Figure S12. The associations between the use of SGLT2i treatment and bone fracture.

Study or Subgroup	SGLT2i Events	SGLT2i Total	Control Events	Control Total	Weight	Odds Ratio M-H, Random, 95% CI
**19.7.5 eGFR >=90**						
DAPA Mathieu 2015	0	160	2	160	0.2%	0.20 [0.01, 4.15]
DAPA Rosenstock 2015	0	179	2	176	0.2%	0.19 [0.01, 4.08]
SOTA Danne 2018	11	524	8	258	2.0%	0.67 [0.27, 1.69]
SOTA Garg 2017	4	699	5	703	1.0%	0.80 [0.21, 3.00]
**Subtotal (95% CI)**		**1562**		**1297**	**3.4%**	**0.62 [0.30, 1.26]**
Total events	15		17			
Heterogeneity: Tau² = 0.00; Chi² = 1.30, df = 3 (P = 0.73); I² = 0%						
Test for overall effect: Z = 1.32 (P = 0.19)						
**19.7.6 eGFR 60-89**						
CANA Bode 2015	17	477	5	137	1.7%	0.98 [0.35, 2.69]
CANA Inagaki 2016	0	75	1	71	0.2%	0.31 [0.01, 7.77]
CANA Kadowaki 2017	1	70	0	68	0.2%	2.96 [0.12, 73.85]
CANA Lavalle-González 2013-1	1	368	0	183	0.2%	1.50 [0.06, 36.95]
DAPA Araki 2017	6	123	0	60	0.2%	6.69 [0.37, 120.82]
DAPA McMurray 2019	49	2373	50	2371	10.9%	0.98 [0.66, 1.46]
EMPA Barnett 2014-1	2	195	3	95	0.5%	0.32 [0.05, 1.93]
EMPA Kawamori 2018	4	182	1	93	0.4%	2.07 [0.23, 18.77]
EMPA Kovacs 2014	3	333	4	165	0.8%	0.37 [0.08, 1.65]
EMPA Packer 2020	45	1863	42	1863	9.6%	1.07 [0.70, 1.64]
EMPA Zinman 2015	179	4687	91	2333	26.2%	0.98 [0.76, 1.27]
SOTA Buse 2018	14	525	10	268	2.5%	0.71 [0.31, 1.61]
**Subtotal (95% CI)**		**11271**		**7707**	**53.3%**	**0.97 [0.81, 1.16]**
Total events	321		207			
Heterogeneity: Tau² = 0.00; Chi² = 7.04, df = 11 (P = 0.80); I² = 0%						
Test for overall effect: Z = 0.35 (P = 0.72)						
**19.7.7 eGFR 45-59**						
DAPA Pollock 2019	1	145	2	148	0.3%	0.51 [0.05, 5.65]
EMPA Barnett 2014-3	3	187	9	187	1.0%	0.32 [0.09, 1.21]
**Subtotal (95% CI)**		**332**		**335**	**1.3%**	**0.36 [0.11, 1.14]**
Total events	4		11			
Heterogeneity: Tau² = 0.00; Chi² = 0.10, df = 1 (P = 0.75); I² = 0%						
Test for overall effect: Z = 1.74 (P = 0.08)						
**19.7.8 eGFR <45**						
DAPA Heerspink 2020	85	2149	69	2149	16.6%	1.24 [0.90, 1.72]
DAPA Kohan 2013	13	168	0	84	0.2%	14.67 [0.86, 249.89]
SOTA Bhatt 2021	111	5291	117	5286	25.2%	0.95 [0.73, 1.23]
**Subtotal (95% CI)**		**7608**		**7519**	**42.0%**	**1.13 [0.75, 1.71]**
Total events	209		186			
Heterogeneity: Tau² = 0.07; Chi² = 5.00, df = 2 (P = 0.08); I² = 60%						
Test for overall effect: Z = 0.61 (P = 0.54)						
**Total (95% CI)**		**20773**		**16858**	**100.0%**	**0.98 [0.86, 1.12]**
Total events	549		421			
Heterogeneity: Tau² = 0.00; Chi² = 18.60, df = 20 (P = 0.55); I² = 0%						
Test for overall effect: Z = 0.28 (P = 0.78)						
Test for subgroup differences: Chi² = 4.89, df = 3 (P = 0.18), I² = 38.6%						

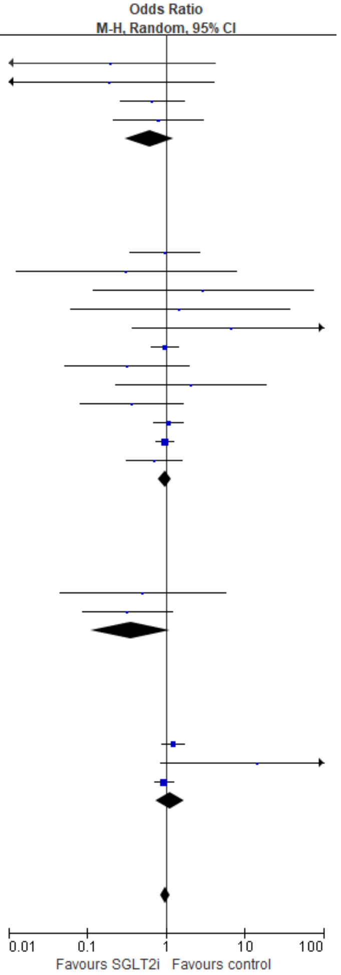

Figure S13. The associations between the use of SGLT2i treatment and diabetic ketoacidosis (DKA).

Study or Subgroup	SGLT2i Events	SGLT2i Total	Control Events	Control Total	Weight	Odds Ratio M-H, Random, 95% CI
**19.8.5 eGFR >=90**						
DAPA Jeon 2018	1	162	0	148	2.1%	2.76 [0.11, 68.24]
SOTA Danne 2018	15	524	0	258	2.6%	15.73 [0.94, 263.90]
SOTA Garg 2017	21	699	4	703	11.5%	5.41 [1.85, 15.85]
**Subtotal (95% CI)**		**1385**		**1109**	**16.2%**	**5.76 [2.21, 15.03]**
Total events	37		4			
Heterogeneity: Tau² = 0.00; Chi² = 0.76, df = 2 (P = 0.68); I² = 0%						
Test for overall effect: Z = 3.58 (P = 0.0003)						
**19.8.6 eGFR 60-89**						
CANA Inagaki 2013	24	307	2	75	7.7%	3.10 [0.72, 13.40]
CANA Inagaki 2016	3	75	2	71	5.5%	1.44 [0.23, 8.87]
CANA Kadowaki 2017	2	70	2	68	4.8%	0.97 [0.13, 7.09]
DAPA Cho 2018	1	36	1	35	2.6%	0.97 [0.06, 16.16]
DAPA McMurray 2019	3	2373	0	2371	2.4%	7.00 [0.36, 135.65]
EMPA Zinman 2015	4	4687	1	2333	4.1%	1.99 [0.22, 17.83]
IPRA Ishihara 2016	5	175	1	87	4.2%	2.53 [0.29, 21.99]
SOTA Buse 2018	8	525	0	268	2.6%	8.82 [0.51, 153.40]
TOFO Kaku 2014	21	174	1	56	4.6%	7.55 [0.99, 57.46]
TOFO Terauchi 2017	5	140	2	70	6.4%	1.26 [0.24, 6.66]
**Subtotal (95% CI)**		**8562**		**5434**	**44.8%**	**2.32 [1.22, 4.42]**
Total events	76		12			
Heterogeneity: Tau² = 0.00; Chi² = 5.04, df = 9 (P = 0.83); I² = 0%						
Test for overall effect: Z = 2.56 (P = 0.01)						
**19.8.7 eGFR 45-59**						
DAPA Pollock 2019	1	145	0	148	2.1%	3.08 [0.12, 76.30]
**Subtotal (95% CI)**		**145**		**148**	**2.1%**	**3.08 [0.12, 76.30]**
Total events	1		0			
Heterogeneity: Not applicable						
Test for overall effect: Z = 0.69 (P = 0.49)						
**19.8.8 eGFR <45**						
DAPA Heerspink 2020	0	2149	2	2149	2.3%	0.20 [0.01, 4.16]
DAPA Kohan 2013	18	168	13	84	16.1%	0.66 [0.30, 1.41]
SOTA Bhatt 2021	30	5291	14	5286	18.5%	2.15 [1.14, 4.05]
**Subtotal (95% CI)**		**7608**		**7519**	**36.9%**	**1.00 [0.33, 3.02]**
Total events	48		29			
Heterogeneity: Tau² = 0.59; Chi² = 6.90, df = 2 (P = 0.03); I² = 71%						
Test for overall effect: Z = 0.00 (P = 1.00)						
**Total (95% CI)**		**17700**		**14210**	**100.0%**	**2.07 [1.28, 3.35]**
Total events	162		45			
Heterogeneity: Tau² = 0.22; Chi² = 21.47, df = 16 (P = 0.16); I² = 25%						
Test for overall effect: Z = 2.98 (P = 0.003)						
Test for subgroup differences: Chi² = 5.63, df = 3 (P = 0.13), I² = 46.7%						

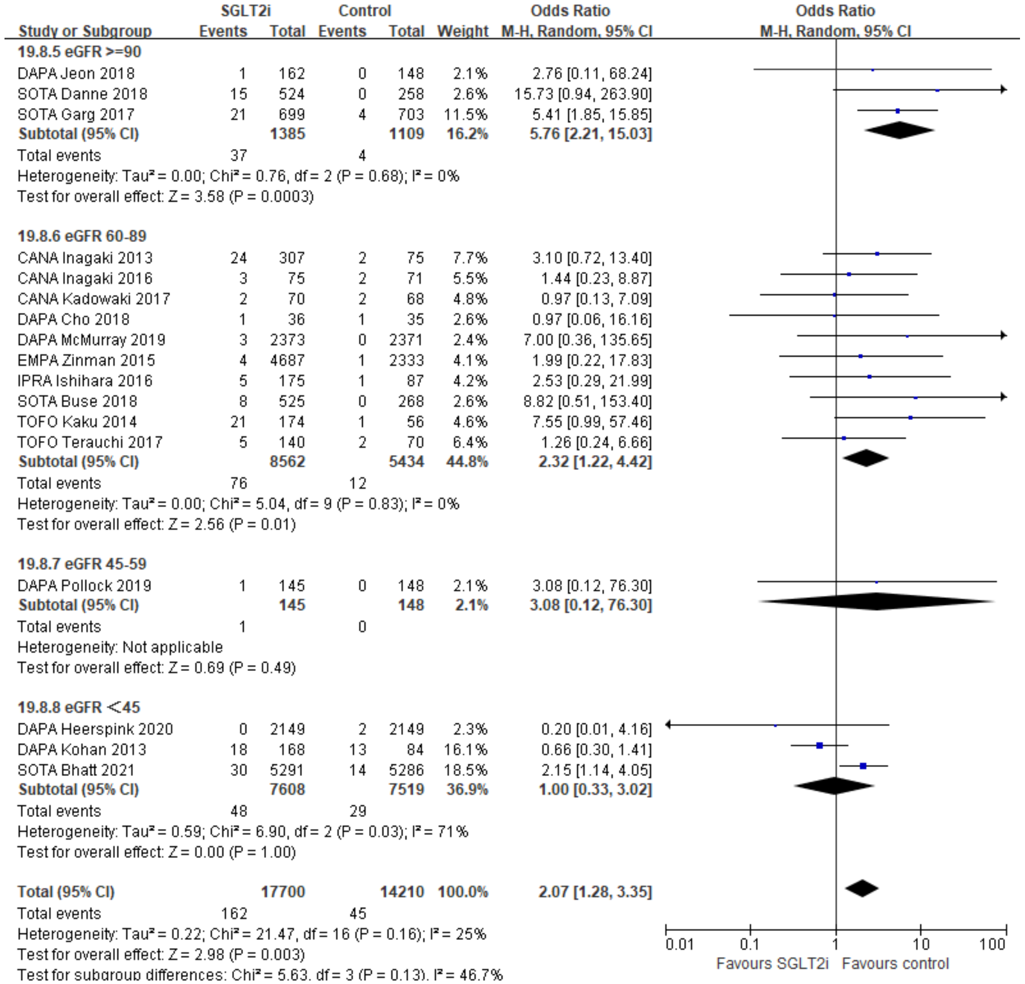

Figure S14. The associations between the use of SGLT2i treatment and hypoglycemia.

Study or Subgroup	SGLT2i Events	SGLT2i Total	Control Events	Control Total	Weight	Odds Ratio M-H, Random, 95% CI
**19.9.5 eGFR >=90**						
CANA Leiter 2014	73	968	197	482	2.2%	0.12 [0.09, 0.16]
CANA Wilding 2013	110	313	28	156	2.1%	2.48 [1.55, 3.96]
DAPA Jabbour 2018	12	231	7	230	1.6%	1.75 [0.67, 4.52]
DAPA Jeon 2018	3	162	22	148	1.3%	0.11 [0.03, 0.37]
DAPA Ji 2014	2	261	2	132	0.8%	0.50 [0.07, 3.60]
DAPA Mathieu 2015	2	160	0	160	0.4%	5.06 [0.24, 106.30]
DAPA Rosenstock 2015	2	179	2	176	0.8%	0.98 [0.14, 7.06]
DAPA Wilding 2012	345	610	102	197	2.2%	1.21 [0.88, 1.67]
EMPA Ferdinand 2019	3	80	0	77	0.4%	7.00 [0.36, 137.80]
EMPA Ferrannini 2013-1	3	215	2	56	0.9%	0.38 [0.06, 2.34]
EMPA Ferrannini 2013-2	7	332	2	56	1.0%	0.58 [0.12, 2.87]
EMPA Hadjadj 2016-1	3	339	0	171	0.4%	3.57 [0.18, 69.46]
EMPA Hadjadj 2016-2	4	341	2	170	0.9%	1.00 [0.18, 5.50]
EMPA Lewin 2015-2	0	136	1	135	0.4%	0.33 [0.01, 8.13]
ERTU Ji 2019	25	339	5	167	1.5%	2.58 [0.97, 6.86]
ERTU Miller 2018	6	194	1	97	0.7%	3.06 [0.36, 25.81]
ERTU Pratley 2018	18	487	6	247	1.6%	1.54 [0.60, 3.93]
ERTU Rosenstock 2018-1	16	205	9	209	1.7%	1.88 [0.81, 4.36]
IPRA Inoue 2019	13	24	10	24	1.4%	1.65 [0.53, 5.18]
SOTA Danne 2018	515	524	252	258	1.5%	1.36 [0.48, 3.87]
SOTA Garg 2017	673	699	670	703	2.0%	1.27 [0.75, 2.16]
**Subtotal (95% CI)**		**6799**		**4051**	**25.6%**	**1.07 [0.57, 2.04]**
Total events	1835		1320			
Heterogeneity: Tau² = 1.64; Chi² = 218.32, df = 20 (P < 0.00001); I² = 91%						
Test for overall effect: Z = 0.22 (P = 0.83)						
**19.9.6 eGFR 60-89**						
CANA Bode 2015	473	477	237	237	0.4%	0.22 [0.01, 4.13]
CANA Inagaki 2013	15	307	0	75	0.4%	8.00 [0.47, 135.25]
CANA Inagaki 2014	12	178	3	93	1.2%	2.17 [0.60, 7.89]
CANA Inagaki 2016	30	75	21	71	1.9%	1.59 [0.80, 3.16]
CANA Rosenstock 2016	23	474	11	237	1.8%	1.05 [0.50, 2.19]
CANA Schernthaner 2013	163	377	154	378	2.2%	1.11 [0.83, 1.48]
DAPA Araki 2017	43	123	25	60	1.9%	0.75 [0.40, 1.42]
DAPA Bailey 2015	6	199	4	75	1.2%	0.55 [0.15, 2.01]
DAPA Bolinder 2012	2	91	3	91	0.9%	0.66 [0.11, 4.04]
DAPA Cho 2018	7	36	9	35	1.4%	0.70 [0.23, 2.14]
DAPA McMurray 2019	4	2373	4	2371	1.2%	1.00 [0.25, 4.00]
DAPA Müller-Wieland 2018	29	625	329	312		Not estimable
DAPA Nauck 2011	14	406	162	408	2.0%	0.05 [0.03, 0.10]
DAPA Schumm-Draeger 2015	3	299	0	101	0.4%	2.40 [0.12, 46.79]
DAPA Scott 2018	21	306	22	307	1.9%	0.95 [0.51, 1.77]
DAPA Strojek 2011	33	450	7	146	1.7%	1.57 [0.68, 3.63]
DAPA Weber 2016	13	225	6	224	1.5%	2.23 [0.83, 5.97]
DAPA Wilding 2009	13	48	3	23	1.2%	2.48 [0.63, 9.75]
DAPA Yang 2018	33	139	30	133	2.0%	1.07 [0.61, 1.88]
EMPA Araki 2015	15	273	5	63	1.5%	0.67 [0.24, 1.93]
EMPA Barnett 2014-1	51	195	25	95	2.0%	0.99 [0.57, 1.73]
EMPA Haring 2013	61	441	19	225	2.0%	1.74 [1.01, 2.99]
EMPA Haring 2014	7	430	1	207	0.7%	3.41 [0.42, 27.89]
EMPA Kadowaki 2014	2	438	0	109	0.4%	1.25 [0.06, 26.32]
EMPA Kawamori 2018	0	182	1	93	0.4%	0.17 [0.01, 4.19]
EMPA Kovacs 2014	6	333	3	165	1.1%	0.99 [0.24, 4.01]
EMPA Lewin 2015-1	0	136	1	135	0.4%	0.33 [0.01, 8.13]
EMPA Packer 2020	27	1863	28	1863	2.0%	0.96 [0.57, 1.64]
EMPA Roden 2013	2	447	1	229	0.6%	1.02 [0.09, 11.36]
EMPA Rosenstock 2014	207	375	111	188	2.2%	0.85 [0.60, 1.22]
EMPA Rosenstock 2015	117	324	60	170	2.1%	1.04 [0.70, 1.53]
EMPA Ross 2015	3	876	1	107	0.6%	0.36 [0.04, 3.53]
EMPA Zinman 2015	1303	4687	650	2333	2.3%	1.00 [0.89, 1.11]
ERTU Dagogo-Jack 2018	10	309	6	153	1.5%	0.82 [0.29, 2.30]
ERTU Hollander 2019	45	880	96	435	2.2%	0.19 [0.13, 0.28]
ERTU Rosenstock 2018-1	15	207	9	209	1.7%	1.74 [0.74, 4.06]
ERTU Terra 2017	6	308	2	153	1.0%	1.50 [0.30, 7.52]
IPRA Han 2018	0	74	1	68	0.4%	0.30 [0.01, 7.54]
IPRA Ishihara 2016	52	175	13	87	1.9%	2.41 [1.23, 4.72]
IPRA Kashiwagi 2015	1	119	0	46	0.4%	1.18 [0.05, 29.42]
LUSEO Seino 2018	33	159	10	74	1.8%	1.68 [0.78, 3.62]
SOTA Buse 2018	518	525	266	268	1.0%	0.56 [0.11, 2.70]
TOFO Kaku 2014	2	174	0	56	0.4%	1.64 [0.08, 34.62]
TOFO Terauchi 2017	44	140	15	70	1.9%	1.68 [0.86, 3.29]
**Subtotal (95% CI)**		**21278**		**12978**	**57.5%**	**0.96 [0.74, 1.23]**
Total events	3464		2354			
Heterogeneity: Tau² = 0.38; Chi² = 210.71, df = 42 (P < 0.00001); I² = 80%						
Test for overall effect: Z = 0.36 (P = 0.72)						
**19.9.7 eGFR 45-59**						
DAPA Fioretto 2018	44	160	62	161	2.1%	0.61 [0.38, 0.97]
DAPA Pollock 2019	54	145	46	148	2.1%	1.32 [0.81, 2.14]
EMPA Barnett 2014-3	54	187	55	187	2.1%	0.97 [0.62, 1.52]
ERTU Grunberger 2017	59	313	27	154	2.0%	1.09 [0.66, 1.81]
LUSEO Haneda 2016	3	95	3	50	1.0%	0.51 [0.10, 2.63]
**Subtotal (95% CI)**		**900**		**700**	**9.2%**	**0.94 [0.69, 1.27]**
Total events	214		193			
Heterogeneity: Tau² = 0.04; Chi² = 6.11, df = 4 (P = 0.19); I² = 35%						
Test for overall effect: Z = 0.43 (P = 0.67)						
**19.9.8 eGFR <45**						
DAPA Heerspink 2020	14	2149	28	2149	1.9%	0.50 [0.26, 0.95]
DAPA Kohan 2013	71	168	43	84	2.0%	0.70 [0.41, 1.18]
EMPA Barnett 2014-4	14	37	12	37	1.6%	1.27 [0.49, 3.30]
SOTA Bhatt 2021	53	5291	55	5286	2.2%	0.96 [0.66, 1.41]
**Subtotal (95% CI)**		**7645**		**7556**	**7.6%**	**0.79 [0.57, 1.11]**
Total events	152		138			
Heterogeneity: Tau² = 0.03; Chi² = 4.16, df = 3 (P = 0.24); I² = 28%						
Test for overall effect: Z = 1.37 (P = 0.17)						
**Total (95% CI)**		**36622**		**25285**	**100.0%**	**0.96 [0.78, 1.18]**
Total events	5665		4005			
Heterogeneity: Tau² = 0.50; Chi² = 450.98, df = 72 (P < 0.00001); I² = 84%						
Test for overall effect: Z = 0.40 (P = 0.69)						
Test for subgroup differences: Chi² = 1.09, df = 3 (P = 0.78), I² = 0%						

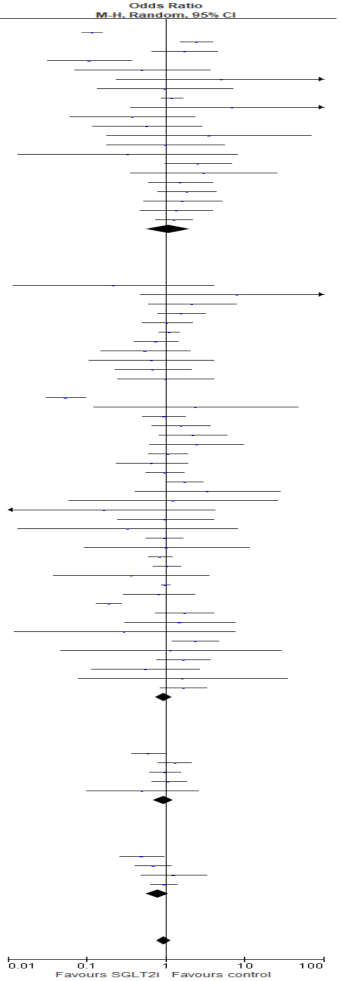

Table S1. Baseline characteristics of included RCTs.

First author, year	Condition	Study duration(w)	Treatment group	Dosage	No. of patients	Age(year)	Male percentage (%)	Baseline eGFR(ml/min/1.73m^2)
**baseline eGFR >= 90 ml/min/1.73m^2**								
**Cusi 2018[1]**	T2D	24	cana	300mg	26	58.00	62.00	92.00±16.00
			pbo		30	58.00	70.00	89.00±17.00
**Ji 2015[2]**	T2D	18	cana	100mg	223	56.50	55.30	92.50±18.30
			cana	300mg	227	56.40	49.80	93.90±18.00
			pbo		226	55.80	55.30	95.40±18.20
**Lavalle-González 2013[3]**	T2D	26	cana	300mg	367	55.30	45.00	90.20± /
			pbo		183	55.30	51.40	87.7± /
**Wilding 2013[4]**	T2D	52	cana	100mg qd	157	57.40	48.40	91.00± /
			cana	300mg qd	156	56.10	55.80	91.90± /
			pbo		156	56.80	48.70	87.40± /
**Henry 2018[5]**	T2D	4	dapa	10mg	50	56.90	52.00	91.40±22.00
			pbo		50	56.80	50.00	89.70±23.60
**Jabbour 2018[6]**	T2D	52	dapa	10mg	228	53.80	44.70	97.70±23.70
			pbo		227	54.20	51.10	99.40±26.80
**Ji 2014[7]**	T2D	24	dapa	5mg	128	53.00	65.60	91.60±17.10
			dapa	10mg	133	51.20	64.70	91.70±20.20
			pbo		132	49.90	65.90	94.10±17.70
**Mathieu 2015[8]**	T2D	24	dapa	10mg	160	55.20	43.70	93.50±20.80
			pbo		160	55.00	47.50	91.60±23.20
**Rosenstock 2015[9]**	T2D	24	dapa	10mg	179	53.00	47.00	96.60±19.60

			pbo		176	55.00	53.00	92.50±19.50
**Wilding 2012[10]**	T2D	48	dapa	2.5mg	202	59.80	49.50	111.30±36.00
			dapa	5mg	211	59.30	47.40	109.70±36.70
			dapa	10mg	194	59.30	44.80	114.30±34.90
			pbo		193	58.80	49.20	115.90±39.70
**Ferdinand 2019[11]**	T2D	24	empa	10mg-25mg	78	56.50	55.10	91.15±18.95
			pbo		72	57.20	50.00	91.49±20.79
**Hadjadj 2016[12]**	T2D	24	empa	10mg	161	52.20	60.20	93.60±22.30
			empa	25mg	165	51.00	63.60	95.00±20.90
			pbo		168	53.40	51.20	90.80±19.30
			empa	10mg	167	52.30	59.30	93.30±22.00
			empa	25mg	169	53.60	52.10	92.20±19.20
			pbo		164	51.60	56.10	93.40±20.10
**Lewin 2015[13]**	T2D	52	empa	25mg	134	54.20	52.20	90.10±19.60
			pbo		133	53.80	56.40	89.50±20.30
**Ji 2019[14]**	T2D	26	ertu	5mg	170	56.10	55.90	97.90±19.20
			ertu	15mg	169	56.30	58.00	100.20±19.80
			pbo		167	56.90	52.70	99.90±20.20
**Pratley 2018[15]**	T2D	26	ertu	5mg	243	55.20	50.60	91.90±20.40
			ertu	15mg	244	55.10	51.60	92.60±19.20
			pbo		247	54.80	62.30	92.60±18.20
**Rosenstock 2018[16]**	T2D	26	ertu	15mg	205	56.90	45.40	91.00±20.60
			pbo		209	56.50	46.90	91.60±19.80
**Inoue 2019[17]**	T2D	24	ipra	50mg	24	60.50	54.20	91.00±26.40

			pbo		24	60.80	58.30	82.90±24.80
**Lu 2016[18]**	T2D	24	ipra	50mg	87	53.90	50.60	144.12±59.42
			pbo		83	53.40	39.80	154.48±69.97
**Danne 2018[19]**	T1D	52	sota	200mg	261	42.30	53.30	91.56±18.78
			sota	400mg	263	41.70	50.60	91.66±17.41
			pbo		258	39.70	51.90	92.89±18.25
**Garg 2017[20]**	T1D	24	sota	400mg	699	43.30	51.20	91.51±19.76
			pbo		703	42.40	48.20	92.47±21.89
**Zambrowicz 2012[21]**	T2D	4	sota	150mg qd	12	53.00	50.00	129.00±28.00
			sota	300mg qd	12	52.00	66.70	139.00±36.00
			pbo		12	55.00	50.00	114.00±18.00
**Leiter 2014[22]**	T2D	104	cana	300mg	485	55.80	49.70	91.40±19.40
			glimepiride		482	56.30	54.60	89.50±17.50
**Handelsman 2018[23]**	T2D	26	dapa+saxagliptin	10mg+5mg	232	55.90	43.10	92.20±20.20
			sitagliptin	100mg	229	55.80	48.00	92.90±22.50
**Jeon 2018[24]**	T2D	24	dapa	10mg qd	162	59.24	54.32	99.69±10.52
			Insulin glargine		148	56.13	47.30	98.60±12.34
**Ferrannini 2013[25]**	T2D	90	empa	10mg	80	59.00	46.20	94.40±18.90
			empa	25mg	88	59.00	52.30	92.30±19.10
			metformin		56	58.00	50.00	88.00±19.30
	T2D	90	empa	10mg	137	60.00	50.00	93.60±18.10
			empa	25mg	139	60.00	53.00	92.60±21.40

			sitagliptin		56	60.00	51.80	91.40±17.50
**Miller 2018[26]**	T2D	26	ertu+sitagliptin	5mg	98	56.40	58.20	90.00±17.20
			pbo		97	54.30	58.80	92.60±21.60
**baseline eGFR [60,90) ml/min/1.73m²**								
**Bode 2015[27]**	T2D	104	cana	100mg	241	64.30	51.50	77.60±17.00
			cana	300mg	236	63.40	54.70	78.70±16.40
			pbo		237	63.20	60.30	76.10±16.30
**Forst 2014[28]**	T2D	26	cana	100mg	113	56.70	68.10	84.60±17.50
			cana	300mg	114	57.00	55.30	87.40±19.50
			pbo		115	58.30	66.10	87.20±18.80
**Inagaki 2013[29]**	T2D	12	cana	50mg	82	57.40	61.00	83.50±16.10
			cana	100mg	74	57.70	70.30	86.90±15.50
			cana	200mg	76	57.00	64.50	83.80±15.00
			cana	300mg	75	57.10	73.30	86.90±15.20
			pbo		75	57.70	72.00	83.00±16.50
**Inagaki 2014[30]**	T2D	24	cana	100mg	90	58.40	65.60	81.40±13.80
			cana	200mg	88	57.40	81.80	87.20±18.60
			pbo		93	58.20	64.50	84.70±13.70
**Inagaki 2016[31]**	T2D	16	cana	10mg	76	59.70	57.90	83.80±18.40
			pbo		70	56.10	70.00	86.10±21.70
**Kadowaki 2017[32]**	T2D	24	cana	100mg	70	58.40	77.10	84.70±15.60
			pbo		68	56.00	77.90	83.90±17.10
**Lavalle-González 2013[3]**	T2D	26	cana	100mg	368	55.50	47.30	89.70± /

			pbo		183	55.30	51.40	87.70± /
**Rosenstock 2016[33]**	T2D	26	cana	300mg	237	55.40	48.50	87.00±19.00
			cana	100mg	237	54.20	45.60	89.00±19.00
			pbo		237	55.20	48.90	87.00±19.00
**Stenlof 2013[34]**	T2D	26	cana	300mg	197	55.30	45.20	86.60±19.10
			cana	100mg	195	55.10	41.50	88.50±20.20
			pbo		192	55.70	45.80	86.00±21.50
**Araki 2017[35]**	T2D	52	dapa	10mg	122	58.30	73.00	77.30±19.10
			pbo		60	57.60	66.70	79.60±16.40
**Bailey 2015[36]**	T2D	102	dapa	2.5mg	65	53.00	55.40	85.59±20.13
			dapa	5mg	64	52.60	48.40	83.13±18.43
			dapa	10mg	70	50.60	48.60	85.37±18.95
			pbo		75	52.70	41.30	86.16±17.32
**Bolinder 2012[37]**	T2D	24	dapa	10mg	89	60.60	55.10	86.00±14.20
			pbo		91	60.80	56.00	82.60±16.10
**McMurray 2019[38]**	HF	35	dapa	10mg	2373	66.20	76.20	66.00±19.60
			pbo		2371	66.50	77.00	65.50±19.30
**Schumm-Draeger 2015[39]**	T2D	16	dapa	10mg qd	99	58.50	49.50	82.50±13.82
			dapa	2.5mg bid	100	58.30	37.00	87.70±19.62
			dapa	5mg bid	99	55.30	46.50	86.50±18.10
			pbo		101	58.50	46.50	84.40±18.16
**Strojek 2011[40]**	T2D	24	dapa	2.5mg	154	59.90	50.00	80.90±18.60
			dapa	5mg	142	60.20	50.00	83.50±19.60
			dapa	10mg	151	58.90	43.70	82.20±17.90

			pbo		145	60.30	49.00	80.20±19.10
**Weber 2016[41]**	T2D	12	dapa	10mg	225	56.00	52.00	84.80±19.70
			pbo		224	57.00	58.00	87.00±19.50
**Wilding 2009[42]**	T2D	12	dapa	10mg	24	55.70	54.20	87.20±33.00
			dapa	20mg	24	56.10	54.20	89.20±16.00
			pbo		23	58.40	69.60	86.20±21.50
**Yang 2018[43]**	T2D	24	dapa	10mg	139	56.50	47.50	88.24±19.36
			pbo		133	58.60	48.10	88.32±19.78
**Barnett 2014[44]**	T2D	52	empa	25mg	97	62.00	62.90	72.30±11.20
			empa	10mg	98	63.20	61.20	70.80±10.30
			pbo		95	62.60	58.90	71.80±10.20
**Haring 2013[45]**	T2D	24	empa	10mg	225	57.00	50.00	86.50±21.80
			empa	25mg	216	57.40	53.00	88.30±22.60
			pbo		225	56.90	50.00	86.90±20.10
**Haring 2014[46]**	T2D	24	empa	10mg	217	55.50	58.00	89.50±19.60
			empa	25mg	213	55.60	56.00	87.70±19.30
			pbo		207	56.00	56.00	89.70±21.40
**Hattori 2018[47]**	T2D	52	empa	10mg	51	57.40	74.50	74.30±16.30
			pbo		51	58.10	80.40	71.90±18.60
**Kario 2018[48]**	T2D	12	empa	10mg	68	70.90	52.90	68.40±15.10
			pbo		63	69.30	52.40	70.10±16.20
**Kadowaki 2014[49]**	T2D	12	empa	5mg	110	57.30	76.40	86.49±17.48
			empa	10mg	109	57.90	70.60	85.76±14.63
			empa	25mg	109	57.20	77.10	85.23±15.84
			empa	50mg	110	56.60	77.30	86.52±15.86

			pbo		109	58.70	73.40	84.61±14.91
**Kawamori 2018[50]**	T2D	24	empa	10mg	182	60.00	78.00	89.30±18.30
			pbo		93	59.80	77.40	86.30±15.20
**Kovacs 2014[51]**	T2D	24	empa	10mg	165	54.70	50.30	84.30±20.90
			empa	25mg	168	54.20	50.60	87.40±24.40
			pbo		165	54.60	44.20	85.50±20.10
**Lewin 2015[13]**	T2D	52	empa	10mg	135	55.20	54.10	87.80±17.70
			pbo		133	53.80	56.40	89.50±20.30
**Packer 2020[52]**	HF	52	empa	10mg qd	1863	67.20	76.50	61.80±21.70
			pbo		1867	66.50	75.60	62.20±21.50
**Roden 2013[53]**	T2D	24	empa	10mg	224	56.20	63.00	87.70±19.16
			empa	25mg	224	53.80	65.00	87.54±18.29
			pbo		228	54.90	54.00	86.83±17.94
**Rosenstock 2014[54]**	T2D	52	empa	10mg	186	56.70	52.00	84.10±17.80
			empa	25mg	189	58.00	44.00	84.40±16.60
			pbo		188	55.30	40.00	83.40±15.40
**Rosenstock 2015[55]**	T2D	78	empa	10mg	169	58.60	55.00	85.10±22.80
			empa	25mg	155	59.90	60.00	83.30±25.10
			pbo		170	58.10	53.00	85.00±23.00
**Ross 2015[56]**	T2D	16	empa	12.5mg bid	215	57.60	57.20	88.60±20.00
			empa	25mg qd	214	58.20	53.30	88.90±19.50
			empa	5mg bid	215	58.80	55.80	89.90±22.30
			empa	10mg qd	214	58.50	50.50	89.20±20.60
			pbo		107	57.90	51.40	89.50±18.50

**Dagogo-Jack 2018**[57]	T2D	52	ertu	5mg	156	59.20	51.90	87.00±17.50
			ertu	15mg	153	59.70	53.60	86.90±15.60
			pbo		153	58.30	65.40	89.90±17.50
**Rosenstock 2018**[16]	T2D	26	ertu	5mg	207	56.60	46.90	88.90±17.50
			pbo		209	56.50	46.90	91.60±19.80
**Terra 2017**[58]	T2D	26	ertu	5mg	156	56.80	57.10	88.50±18.40
			ertu	15mg	152	56.20	59.20	88.30±18.00
			pbo		153	56.10	53.60	86.20±19.40
**Bando 2016**[59]	T2D	12	ipra	50mg	40	54.80	65.00	79.50±14.90
			pbo		22	55.40	63.60	76.50±13.40
**Han 2018**[60]	T2D	24	ipra	50mg	73	57.62	50.70	89.38±13.61
			pbo		66	57.44	48.50	90.66±17.47
**Ishihara 2016**[61]	T2D	16	ipra	50mg	168	58.70	62.50	83.98±20.27
			pbo		87	59.20	58.60	80.11±21.94
**Kashiwagi 2015**[62]	T2D	24	ipra	50mg	118	63.90	78.00	60.30±13.07
			pbo		46	65.70	78.30	62.70±13.13
**Seino 2018**[63]	T2D	16	luseo	2.5mg	159	57.40	70.40	86.50±19.30
			pbo		74	57.10	68.90	87.90±20.20
**Buse 2018**[64]	T1D	52	sota	200mg	263	46.60	47.90	87.04±20.18
			sota	400mg	262	46.40	45.80	86.58±18.93
			pbo		268	45.20	51.10	87.58±18.47
**Kaku 2014**[65]	T2D	24	tofo	10mg	57	58.60	66.70	84.68±20.04
			tofo	20mg	58	56.60	67.20	86.78±19.62
			tofo	40mg	58	57.00	67.20	86.00±18.18

			pbo		56	56.80	66.10	83.78±17.68
**Terauchi 2017[66]**	T2D	16	tofo	20mg	141	59.10	63.80	79.70±19.80
			pbo		70	56.40	68.60	79.50±17.00
**Leiter 2014-2[22]**	T2D	104	cana	100ng	483	56.40	52.20	89.70±19.30
			glimepiride		482	56.30	54.60	89.50±17.50
**Miller 2018-2[26]**	T2D	26	ertu+sitagliptin	15mg	96	56.10	55.20	89.50±18.10
			pbo		97	54.30	58.80	92.60±21.60
**Schernthaner 2013[67]**	T2D	52	cana	300mg	377	56.60	54.90	88.30± /
			sitagliptin	100mg	378	56.70	56.90	89.50± /
**Nauck 2011[68]**	T2D	52	dapa	2.5→5→10mg	400	58.00	55.30	89.60±21.40
			glipizide		401	59.00	54.90	90.50±22.60
**Müller-Wieland 2018[69]**	T2D	52	dapa	10mg	314	57.40	64.30	87.20±19.40
			dapa+saxagliptin	10mg+5mg	312	59.20	60.90	88.00±19.60
			glimepiride		313	58.60	66.50	86.00±17.50
**Cho 2018[70]**	T2D	24	dapa	5mg	36	63.10	63.90	72.40±19.60
			pioglitazone		35	63.60	54.30	67.10±16.80
**Scott 2018[71]**	T2D	24	dapa	10mg	306	66.60	60.80	76.90±12.30
			sitagliptin		307	67.70	55.00	79.40±11.30
**Shimizu 2018[72]**	T2D	24	dapa	5mg	33	56.20	57.60	79.40±15.80
			control		24	57.10	62.50	76.90±19.00

**Hayashi 2017[73]**	T2D	12	dapa	5mg	40	53.80	80.00	86.20±18.40
			sitagliptin	50mg	40	54.20	75.00	83.50±22.70
**Zinman 2015[74]**	T2D	135	empa	10mg	2345	63.00	70.50	74.40±21.80
			empa	25mg	2342	63.20	71.90	74.30±21.10
			pbo		2333	63.20	72.00	74.00±21.10
**Araki 2015[75]**	T2D	52	empa	10mg	136	61.30	73.00	87.10±20.30
			empa	25mg	137	61.80	70.00	85.60±19.30
			metformin		63	60.00	75.00	86.90±14.80
**Hollander 2019[76]**	T2D	104	ertu	5mg	445	58.70	51.00	88.30±18.70
			ertu	15mg	435	58.00	43.90	86.60±18.30
			glimepiride		435	57.90	51.50	86.50±18.50
**Tsurutani 2018[77]**	T2D	12	ipra	50mg	60	53.50	68.30	77.20± /
			sitagliptin		59	54.00	67.80	80.40± /
**baseline eGFR [45,60) ml/min/1.73m^2**								
**Takashima 2018[78]**	T2D	52	cana	100mg	21	64.70	55.00	57.10±16.20
			pbo		21	65.40	60.00	55.40±12.30
**Fioretto 2018[79]**	T2D	24	dapa	10mg	160	65.30	56.90	53.48±8.76
			pbo		161	66.20	56.50	53.68±10.67
**Pollock 2019[80]**	T2D	24	dapa	10mg	145	64.70	70.00	50.20±13.00
			pbo		148	64.70	71.00	47.70±13.50
**Barnett 2014[44]**	T2D	52	empa	25mg	187	64.60	57.20	45.40±10.20
			pbo		187	65.10	56.70	44.30±10.30
**Grunberger 2017[81]**	T2D	26	ertu	5mg	158	66.70	53.20	46.80±7.80
			ertu	15mg	155	67.50	48.40	46.90±9.10

			pbo		154	67.50	46.80	46.00±9.40
**Haneda 2016[82]**	T2D	24	luseo	2.5mg	95	67.90	75.80	52.00±9.40
			pbo		50	68.40	78.00	52.40±8.20
**baseline eGFR ＜45ml/min/1.73m^2**								
**Yale 2013[83]**	T2D	26	cana	100mg	90	69.50	64.40	39.70±6.90
			cana	300mg	89	67.90	53.90	38.50±6.90
			pbo		90	68.20	63.30	40.10±6.80
**Heerspink 2020[84]**	CKD	52	dapa	10mg qd	2149	61.80	67.10	43.20±12.30
			pbo		2149	61.90	66.70	43.00±12.40
**Bhatt 2021[85]**	T2D	64	sota	200→400 mg	5292	69.00	55.70	44.40± /
			pbo		5292	69.00	54.50	44.70± /
**Kohan 2013[86]**	T2D	104	dapa	5mg qd	83	66.00	66.30	44.20±8.80
			dapa	10mg qd	85	68.00	65.90	43.90±10.60
			pbo		84	67.00	63.10	45.60±10.00
**Barnett 2014[44]**	T2D	52	empa	25mg	37	65.40	56.80	24.40±5.20
			pbo		37	62.90	51.40	22.00±4.40

Table S2. Evaluation for risk of bias in included RCTs

Author, year	Adequate randomization sequence generation	Adequate allocation concealment	Blinding of participants and caregivers	Blinding of outcome assessors and adjudicators	Free of infrequent missing outcome data	Free of selective outcome reporting	Free of other bias
Cusi,2018[1]	**Definitely yes** Using a computer-generated randomization schedule	**Definitely yes** Randomized, double-blind	**Definitely yes** Double-blind (participant, investigator)	**Definitely yes**	**Probably yes** There were 7.7% (2/26) and 10% (3/30) patients in SGLT2i and placebo groups with missing outcome data, respectively; missing outcome data were generally balanced across treatment groups, with similar reasons for missing data across groups	**Definitely yes**	**Probably yes** Generally balanced baseline characteristics across groups

Ji, 2015[2]	**Definitely yes** Using a computer-generated randomization schedule	**Definitely yes** Randomized, double-blind	**Definitely yes** Double-blind (participant, investigator)	**Definitely yes**	**Definitely yes** There were 4.7% (21/450) and 4.0% (9/226) patients in SGLT2i and placebo groups with missing outcome data, respectively; missing outcome data were generally balanced across treatment groups, with similar reasons for missing data across groups	**Definitely yes**	**Probably yes** Generally balanced baseline characteristics across groups
Lavalle-González, 2013[3]	**Definitely yes** Using a computer-generated randomization schedule	**Definitely yes** Randomized, double-blind	**Definitely yes** Double-blind (participant, investigator)	**Definitely yes**	**Probably no** There were 12.2% (90/735) and 15.3% (28/183) patients in SGLT2i and	**Definitely yes**	**Probably yes** Generally balanced baseline characteristics across groups

					control groups with missing outcome data, respectively; missing outcome data were generally balanced across treatment groups, with similar reasons for missing data across groups		
Wilding,2013[4]	**Definitely yes** Using a computer-generated randomization schedule	**Definitely yes** Randomized, double-blind	**Definitely yes** Double-blind (participant, investigator)	**Definitely yes**	**Definitely no** There were 29.7% (93/313) and 42.3% (66/156) patients in SGLT2i and placebo groups with missing outcome data, respectively; missing outcome	**Definitely yes**	**Probably yes** Generally balanced baseline characteristics across groups

					data were generally balanced across treatment groups, with similar reasons for missing data across groups		
Henry,2018[5]	**Definitely yes** Using an interactive voice response system/interactive web response system	**Definitely yes** Randomized, double-blind	**Definitely yes** Double-blind (participant, investigator)	**Definitely yes**	**Definitely yes** There were 4% (2/50)and 2% (1/50)patients in SGLT2i and placebo groups with missing outcome data, respectively; missing outcome data were generally balanced across treatment groups, with similar reasons for missing data	**Definitely yes**	**Probably yes** Generally balanced baseline characteristics across groups

					across groups		
Jabbour, 2018[6]	**Definitely yes** Using an interactive voice response system and web-response system	**Definitely yes** Randomized, double-blind	**Definitely yes** Double-blind (participant, investigator)	**Definitely yes**	**Definitely yes** There were 1.3% (3/228) and 1.3% (3/227) patients in SGLT2i and placebo groups with missing outcome data, respectively; missing outcome data were generally balanced across treatment groups, with similar reasons for missing data across groups	**Definitely yes**	**Probably yes** Generally balanced baseline characteristics across groups
Ji, 2014[7]	**Definitely yes** Using an interactive voice response system	**Definitely yes** Randomized, double-blind	**Definitely yes** Double-blind (participant, investigator)	**Definitely yes**	**Probably no** There were 11.8% (31/261) and 14.4% (19/132) patients in SGLT2i and	**Definitely yes**	**Probably yes** Generally balanced baseline characteristics across groups

					placebo groups with missing outcome data, respectively; missing outcome data were generally balanced across treatment groups, with similar reasons for missing data across groups		
Mathieu, 2015[8]	**Definitely yes** Using an interactive voice response system	**Definitely yes** Randomized, double-blind	**Definitely yes** Double-blind (participant, investigator)	**Definitely yes**	**Probably yes** There were 7.5% (12/160) and 4.4% (7/160) patients in SGLT2i and placebo groups with missing outcome data, respectively; missing outcome data were	**Definitely yes**	**Probably yes** Generally balanced baseline characteristics across groups

					generally balanced across treatment groups, with similar reasons for missing data across groups		
Rosenstock,2015[9]	**Definitely yes** Using a centralized blocked randomization schedule	**Definitely yes** Randomized, double-blind	**Definitely yes** Double-blind (participant, investigator)	**Definitely yes**	**Probably yes** There were 5.6% (10/179) and 8.5% (15/176) patients in SGLT2i and placebo groups with missing outcome data, respectively; missing outcome data were generally balanced across treatment groups, with similar reasons for missing data	**Definitely yes**	**Probably yes** Generally balanced baseline characteristics across groups

					across groups		
Wilding, 2012[10]	**Definitely yes** Using a computer-generated randomization schedule	**Definitely yes** Randomized, double-blind	**Definitely yes** Double-blind (participant, investigator)	**Definitely yes**	**Probably no** There were 14.5% (88/607) and 18.6% (36/193) patients in SGLT2i and placebo groups with missing outcome data, respectively; missing outcome data were generally balanced across treatment groups, with similar reasons for missing data across groups	**Definitely yes**	**Probably yes** Generally balanced baseline characteristics across groups
Fedinand,2019[11]	**Definitely yes** Using a computer-generated random sequence and a third-party	**Definitely yes** Randomized, double-blind	**Definitely yes** Double-blind (participant, investigator)	**Definitely yes**	**Probably yes** There were 4.9% (4/82) and 8.9% (7/79) patients in SGLT2i and	**Definitely yes**	**Probably yes** Generally balanced baseline characteristics

	interactive telephone/web-based system				control groups with missing outcome data, respectively; missing outcome data were generally balanced across treatment groups, with similar reasons for missing data across groups		across groups
Hadjadj, 2016[12]	**Definitely yes** Using a computer-generated random sequence and an interactive voice and Web response system.	**Definitely yes** Randomized, double-blind	**Definitely yes** Double-blind (participant, investigator)	**Definitely yes**	**Probably yes** There were 7.8% (53/680) and 11.7% (40/341) patients in SGLT2i and control groups with missing outcome data, respectively; missing outcome data were	**Definitely yes**	**Probably yes** Generally balanced baseline characteristics across groups

					generally balanced across treatment groups, with similar reasons for missing data across groups		
Lewin, 2015[13]	**Definitely yes** Using a third-party interactive voice and web response system	**Definitely yes** Randomized, double-blind	**Definitely yes** Double-blind (details not reported)	**Definitely yes**	**Probably no** There were 15.8% (43/273) and 14.1% (19/135) patients in SGLT2i and control groups with missing outcome data, respectively; missing outcome data were generally balanced across treatment groups, with similar reasons for missing data	**Definitely yes**	**Probably yes** Generally balanced baseline characteristics across groups

					across groups		
Ji,2019[14]	**Definitely yes** Using a computer-generated randomization code based on the method of random permuted blocks	**Definitely yes** Randomized, double-blind	**Definitely yes** Double-blind (participant, investigator)	**Definitely yes**	**Probably yes** There were 7.1% (24/339) and 10.2% (17/167) patients in active agent and placebo groups with missing outcome data, respectively; missing outcome data were generally balanced across treatment groups, with similar reasons for missing data across groups	**Definitely yes**	**Probably yes** Generally balanced baseline characteristics across groups
Pratley,2018[15]	**Definitely yes** Using a an interactive voice response system/integrated	**Definitely yes** Randomized, double-blind	**Definitely yes** Double-blind (participant, investigator)	**Definitely yes**	**Probably yes** There were 8.2% (40/488) and 10.5% (26/247) patients in active	**Definitely yes**	**Probably yes** Generally balanced baseline characteristics

	web response system				agent and placebo groups with missing outcome data, respectively; missing outcome data were generally balanced across treatment groups, with similar reasons for missing data across groups		across groups
Rosenstock, 2018[16]	**Definitely yes** Using a computer-generated randomization code based on the method of random permuted blocks	**Definitely yes** Randomized, double-blind	**Definitely yes** Double-blind (participant, investigator)	**Definitely yes**	**Probably yes** There were 5.1% (21/412) and 9.1% (19/209) patients in active agent and placebo groups with missing outcome data, respectively; missing outcome	**Definitely yes**	**Probably yes** Generally balanced baseline characteristics across groups

					data were generally balanced across treatment groups, with similar reasons for missing data across groups		
Inoue,2019[17]	**Definitely yes** Using a computer-generated random sequence	**Definitely yes** Open-labeled	**Definitely no** Open-labeled (participant, investigator)	**Definitely no**	**Probably yes** There were 8.3% (2/24) patients in SGLT-2 group with missing outcome data.	**Definitely yes**	**Probably yes** Generally balanced baseline characteristics across groups
Lu, 2016[18]	**Definitely yes** Using an interactive web randomization system with treatment codes	**Definitely yes** Randomized, double-blind	**Definitely yes** Double-blind (details not reported)	**Definitely yes**	**Probably no** There were 14.9% (13/87) and 16.9% (14/83) patients in SGLT2i and placebo groups with missing outcome data, respectively; missing outcome	**Definitely yes**	**Probably yes** Generally balanced baseline characteristics across groups

					data were generally balanced across treatment groups, with similar reasons for missing data across groups		
Danne, 2018[19]	**Probably yes** Randomized, double-blind	**Probably yes** Randomized, double-blind	**Definitely yes** Double-blind (details not reported)	**Definitely yes**	**Probably no** There were 13.5% (71/524) and 12.8% (33/258) patients in SGLT2i and control groups with missing outcome data, respectively; missing outcome data were generally balanced across treatment groups, with similar reasons for	**Definitely yes**	**Probably yes** Generally balanced baseline characteristics across groups

					missing data across groups		
Grag, 2017[20]	**Probably yes** Randomized, double-blind	**Probably yes** Randomized, double-blind	**Definitely yes** Double-blind (details not reported)	**Definitely yes**	**Probably no** There were 13.6% (95/699) and 11.5% (81/703) patients in SGLT2i and control groups with missing outcome data respectively; missing outcome data were generally balanced across treatment groups, with similar reasons for missing data across groups	**Definitely yes**	**Probably yes** Generally balanced baseline characteristics across groups
Zambrowicz,2012[21]	**Definitely yes** Using a SAS-generated randomization	**Definitely yes** Randomized, double-blind	**Definitely yes** Double-blind (participant,	**Definitely yes**	**Probably yes** There was 8.3% (1/12) patient in placebo group	**Definitely yes**	**Probably yes** Generally balanced baseline

	schedule		investigator)		with missing outcome data.		characteristics across groups
Leiter 2014[22]	**Definitely yes** Using an interactive voice or web response system	**Definitely yes** Randomized, double-blind	**Definitely yes** Double-blind (participant, investigator)	**Definitely yes**	**Definitely no** There were 31.2% (302/968) and 34.9% (168/482) patients in SGLT2i and control groups with missing outcome data, respectively; missing outcome data were generally balanced across treatment groups, with similar reasons for missing data across groups	**Definitely yes**	**Probably yes** Generally balanced baseline characteristics across groups
Handelsman 2018[23]	**Definitely yes** Using an interactive voice response	**Definitely yes** Randomized, double-blind	**Definitely yes** Double-blind	**Definitely yes**	**Probably yes** There were 89.2% (411/468)	**Definitely yes**	**Probably yes** Generally balanced

	system		(participant, investigator)		patients in SGLT2i and control groups with missing outcome data, totally; missing outcome data were generally balanced across treatment groups, with similar reasons for missing data across groups		baseline characteristics across groups
Jeon 2018[24]	**Probably yes**	**Probably yes** Open-labeled	**Definitely no** Open-labeled	**Definitely no**	**Probably yes**	**Definitely yes**	**Probably yes** Generally balanced baseline characteristics across groups
Ferrannini 2013[25]	**Probably yes**	**Probably yes** Open-labeled	**Definitely no** Open-labeled	**Definitely no**	**Probably yes** There were 8.03% (53/660) patients in SGLT2i and	**Definitely yes**	**Probably yes** Generally balanced baseline characteristics

					control groups with missing outcome data, totally; missing outcome data were generally balanced across treatment groups, with similar reasons for missing data across groups		across groups
Miller 2018[26]	**Definitely yes** Using a central electronic randomization system (interactive voice response system/integrated web response system)	**Definitely yes** Randomized, double-blind	**Definitely yes** Double-blind (participant, investigator)	**Definitely yes**	**Probably no** There were 8.2% (16/194) and 21.6% (21/97) patients in SGLT2i and control groups with missing outcome data, respectively; missing outcome data were generally	**Definitely yes**	**Probably yes** Generally balanced baseline characteristics across groups

					balanced across treatment groups, with similar reasons for missing data across groups		
Bode, 2015[27]	**Probably yes** Randomized, double-blind	**Probably yes** Randomized, double-blind	**Definitely yes** Double-blind (participant, investigator)	**Definitely yes**	**Definitely no** There were 24.1% (79/477) and 33.3% (79/237) patients in SGLT2i and control groups with missing outcome data, respectively; missing outcome data were generally balanced across treatment groups, with similar reasons for missing data across groups	**Definitely yes**	**Probably yes** Generally balanced baseline characteristics across groups

Forst, 2014[28]	**Definitely yes** Using an interactive voice response system/interactive web response system	**Definitely yes** Randomized, double-blind	**Definitely yes** Double-blind (participant, investigator)	**Definitely yes**	**Probably no** There were 9.7% (22/227) and 20.9% (24/115) patients in SGLT2i and placebo groups with missing outcome data, respectively; missing outcome data were generally balanced across treatment groups, with similar reasons for missing data across groups	**Definitely yes**	**Probably yes** Generally balanced baseline characteristics across groups
Inagaki, 2013[29]	**Definitely yes** Using a block allocation method	**Definitely yes** Randomized, double-blind	**Definitely yes** Double-blind (participant, investigator)	**Definitely yes**	**Probably no** There were 3.9% (12/307) and 13.3% (10/75) patients in SGLT2i and	**Definitely yes**	**Probably yes** Generally balanced baseline characteristics across groups

					placebo groups with missing outcome data, respectively; missing outcome data were generally balanced across treatment groups, with similar reasons for missing data across groups		
Inagaki, 2014[30]	**Definitely yes** Using a block allocation method	**Definitely yes** Randomized, double-blind	**Definitely yes** Double-blind (participant, investigator)	**Definitely yes**	**Probably no** There were 6.7% (12/179) and 20.4% (19/93) patients in SGLT2i and placebo groups with missing outcome data, respectively; missing outcome data were	**Definitely yes**	**Probably yes** Generally balanced baseline characteristics across groups

					generally balanced across treatment groups, with similar reasons for missing data across groups		
Inagaki, 2016[31]	**Definitely yes** Using a block allocation method	**Definitely yes** Randomized, double-blind	**Definitely yes** Double-blind (participant, investigator)	**Definitely yes**	**Definitely yes** There were 0% (0/76) and 0% (0/70) patients in SGLT2i and placebo groups with missing outcome data, respectively; missing outcome data were generally balanced across treatment groups, with similar reasons for missing data across groups	**Definitely yes**	**Probably yes** Generally balanced baseline characteristics across groups

Kadowaki, 2017[32]	**Probably yes** Randomized, double-blind	**Probably yes** Randomized, double-blind	**Definitely yes** Double-blind (participant, investigator)	**Definitely yes**	**Probably yes** There were 4.3% (3/70) and 10.3% (7/68) patients in SGLT2i and placebo groups with missing outcome data, respectively; missing outcome data were generally balanced across treatment groups, with similar reasons for missing data across groups	**Definitely yes**	**Probably yes** Generally balanced baseline characteristics across groups
Rosenstock, 2016[33]	**Definitely yes** Using a computer-generated randomization schedule	**Definitely yes** Randomized, double-blind	**Definitely yes** Double-blind (participant, investigator)	**Definitely yes**	**Probably no** There were 7.8% (37/474) and 13.5% (32/237) patients in SGLT2i and control groups	**Definitely yes**	**Probably yes** Generally balanced baseline characteristics across groups

					with missing outcome data, respectively; missing outcome data were generally balanced across treatment groups, with similar reasons for missing data across groups		
Stenlöf, 2013[34]	**Probably yes** Randomized, double-blind	**Probably yes** Randomized, double-blind	**Definitely yes** Double-blind (participant, investigator)	**Definitely yes**	**Probably no** There were 11.5% (45/392) and 16.7% (32/192) patients in SGLT2i and placebo groups with missing outcome data, respectively; missing outcome data were generally	**Definitely yes**	**Probably yes** Generally balanced baseline characteristics across groups

					balanced across treatment groups, with similar reasons for missing data across groups		
Araki, 2017[35]	**Definitely yes** Using an interactive web response system	**Definitely yes** Randomized, double-blind	**Definitely yes** Double-blind (participant, investigator)	**Definitely yes**	**Probably yes** There were 9.0% (11/122) and 10% (6/60) patients in SGLT2i and placebo groups with missing outcome data, respectively; missing outcome data were generally balanced across treatment groups, with similar reasons for missing data across groups	**Definitely yes**	**Probably yes** Generally balanced baseline characteristics across groups
Bailey, 2015[36]	**Definitely yes**	**Definitely yes**	**Definitely**	**Definitely**	**Definitely no**	**Definitely**	**Probably yes**

	Using a computer-generated randomization by an interactive voice response system and were stratified by site in blocks of seven.	Randomized, double-blind	**yes** Double-blind (participant, investigator)	**yes**	There were 37.2% (74/199) and 44% (33/75) patients in SGLT2i and placebo groups with missing outcome data, respectively; missing outcome data were generally balanced across treatment groups, with similar reasons for missing data across groups	**yes**	Generally balanced baseline characteristics across groups
Bolinder, 2012[37]	**Definitely yes** Using a computer-generated randomization schedule	**Definitely yes** Randomized, double-blind	**Definitely yes** Double-blind (participant, investigator)	**Definitely yes**	**Probably yes** There were 8.8% (8/91) and 5.5% (5/91) patients in SGLT2i and placebo groups with missing	**Definitely yes**	**Probably yes** Generally balanced baseline characteristics across groups

					outcome data, respectively; missing outcome data were generally balanced across treatment groups, with similar reasons for missing data across groups		
McMurray, 2019[38]	**Definitely yes** Using an interactive voice or web response system	**Definitely yes** Randomized	**Probably yes** Randomized	**Probably yes**	**Probably yes** There were 10.5% (249/2373) and 10.9% (258/2371) patients in SGLT2i and control groups with missing outcome data, respectively; missing outcome data were	**Definitely yes**	**Probably yes** Generally balanced baseline characteristics across groups

					generally balanced across treatment groups, with similar reasons for missing data across groups		
Schumm-Draeger, 2015[39]	**Definitely yes** Using an interactive web response system	**Definitely yes** Randomized, double-blind	**Definitely yes** Double-blind (participant, investigator)	**Definitely yes**	**Probably yes** There were 7.4% (22/299) and 7.9% (8/101) patients in SGLT2i and control groups with missing outcome data, respectively; missing outcome data were generally balanced across treatment groups, with similar reasons for missing data	**Definitely yes**	**Probably yes** Generally balanced baseline characteristics across groups

					across groups		
Strojek, 2011[40]	**Definitely yes** Using a computer-generated randomization schedule	**Definitely yes** Randomized, double-blind	**Definitely yes** Double-blind (participant, investigator)	**Definitely yes**	**Probably yes** There were 8.2% (37/450)and 8.9% (13/146)patients in SGLT2i and placebo groups with missing outcome data, respectively; missing outcome data were generally balanced across treatment groups, with similar reasons for missing data across groups	**Definitely yes**	**Probably yes** Generally balanced baseline characteristics across groups
Weber,2016[41]	**Definitely yes** Using a block allocation method	**Definitely yes** Randomized, double-blind	**Definitely yes** Double-blind (participant, investigator)	**Definitely yes**	**Probably yes** There were 6.2% (14/225)and 9.8% (22/224)patients in SGLT2i and placebo groups	**Definitely yes**	**Probably yes** Generally balanced baseline characteristics across groups

					with missing outcome data, respectively; missing outcome data were generally balanced across treatment groups, with similar reasons for missing data across groups		
Wilding, 2009[42]	**Probably yes** Randomized, double-blind	**Probably yes** Randomized, double-blind	**Definitely yes** Double-blind (participant, investigator)	**Definitely yes**	**Definitely no** There were 8.3% (4/48) and 30.4% (7/23) patients in SGLT2i and placebo groups with missing outcome data, respectively; missing outcome data were generally balanced across	**Definitely yes**	**Probably yes** Generally balanced baseline characteristics across groups

					treatment groups, with similar reasons for missing data across groups		
Yang, 2018[43]	**Definitely yes** Using an interactive voice response system	**Definitely yes** Randomized, double-blind	**Definitely yes** Double-blind (participant, investigator)	**Definitely yes**	**Probably yes** There were 3.6% (5/139) and 6.8% (9/133) patients in SGLT2i and placebo groups with missing outcome data, respectively; missing outcome data were generally balanced across treatment groups, with similar reasons for missing data across groups	**Definitely yes**	**Probably yes** Generally balanced baseline characteristics across groups
Barnett, 2014[44]	**Definitely yes** Using a	**Definitely yes** Randomized,	**Definitely yes**	**Definitely yes**	**Probably yes** There were 9.2%	**Definitely yes**	**Probably yes** Generally

	computer-generated random sequence	double-blind	Double-blind (participant, investigator)		(18/195) and 10.3% (10/97) patients in SGLT2i and control groups with missing outcome data, respectively; missing outcome data were generally balanced across treatment groups, with similar reasons for missing data across groups		balanced baseline characteristics across groups
Haring, 2013[45]	**Definitely yes** Using a third-party interactive voice and web response system	**Definitely yes** Randomized, double-blind	**Definitely yes** Double-blind (details not reported)	**Definitely yes**	**Probably yes** There were 8.3% (37/444) and 10.7% (24/225) patients in SGLT2i and control groups with missing	**Definitely yes**	**Probably yes** Generally balanced baseline characteristics across groups

					outcome data, respectively; missing outcome data were generally balanced across treatment groups, with similar reasons for missing data across groups		
Haring, 2014[46]	**Definitely yes** Using a third-party interactive voice and web response system	**Definitely yes** Randomized, double-blind	**Definitely yes** Double-blind (details not reported)	**Definitely yes**	**Probably yes** There were 6.0% (26/431) and 10.1% (21/207) patients in SGLT2i and control groups with missing outcome data, respectively; missing outcome data were generally balanced across	**Definitely yes**	**Probably yes** Generally balanced baseline characteristics across groups

					treatment groups, with similar reasons for missing data across groups		
Hattori,2018[47]	**Probably yes** Randomized	**Probably yes** Open-labeled	**Definitely no** Open-labeled (participant, investigator)	**Definitely no**	**Probably yes**	**Definitely yes**	**Probably yes** Generally balanced baseline characteristics across groups
Kario,2018[48]	**Definitely yes** Using a computer-generated randomization sequence	**Definitely yes** Randomized, double-blind	**Definitely yes** Double-blind (participant, investigator)	**Definitely yes**	**Definitely yes** There were 1.6% (1/64) patients in placebo group with missing outcome data.	**Definitely yes**	**Probably yes** Generally balanced baseline characteristics across groups
Kadowaki, 2014[49]	**Definitely yes** Using a computer-generated random sequence and an interactive web response system	**Definitely yes** Randomized, double-blind	**Definitely yes** Double-blind (participant, investigator)	**Definitely yes**	**Probably yes** There were 2.3% (10/438) and 8.3% (9/109) patients in SGLT2i and placebo groups with missing	**Definitely yes**	**Probably yes** Generally balanced baseline characteristics across groups

					outcome data, respectively; missing outcome data were generally balanced across treatment groups, with similar reasons for missing data across groups		
Kawamori,2018[50]	**Definitely yes** Using a computer-generated random sequence using a web-based interactive response system	**Definitely yes** Randomized, double-blind	**Definitely yes** Double-blind (participant, investigator)	**Definitely yes**	**Probably yes** There were 2.7% (5/182) and 7.5% (7/93) patients in SGLT2i and control groups with missing outcome data, respectively; missing outcome data were generally balanced across treatment groups,	**Definitely yes**	**Probably yes** Generally balanced baseline characteristics across groups

					with similar reasons for missing data across groups		
Kovacs, 2014[51]	**Definitely yes** Using a computer-generated random sequence and an interactive voice and web response system	**Definitely yes** Randomized, double-blind	**Definitely yes** Double-blind (participant, investigator)	**Definitely yes**	**Probably yes** There were 6.9% (23/333)and 10.9% (18/165)patients in SGLT2i and placebo groups with missing outcome data, respectively; missing outcome data were generally balanced across treatment groups, with similar reasons for missing data across groups	**Definitely yes**	**Probably yes** Generally balanced baseline characteristics across groups
Packer, 2020[52]	**Definitely yes** Using an	**Definitely yes** Randomized,	**Definitely yes**	**Definitely yes**	**Definitely yes** There were 1.2%	**Definitely yes**	**Probably yes** Generally

	interactive-response system that used a permuted-block design	double-blind	Double-blind (participant, investigator)		(22/1863) and 1.1% (20/1867) patients in active agent and placebo groups with missing outcome data, respectively; missing outcome data were generally balanced across treatment groups, with similar reasons for missing data across groups		balanced baseline characteristics across groups
Roden, 2013[53]	**Definitely yes** an interactive voice and internet-based response system, with a computer-generated random sequence, in block sizes of four	**Definitely yes** Randomized, double-blind	**Definitely yes** Double-blind (participant, investigator)	**Definitely yes**	**Probably no** There were 8.5% (38/448)and 18.0% (41/228)patients in SGLT2i and placebo groups with missing	**Definitely yes**	**Probably yes** Generally balanced baseline characteristics across groups

					outcome data, respectively; missing outcome data were generally balanced across treatment groups, with similar reasons for missing data across groups		
Rosenstock, 2014[54]	**Definitely yes** Using a third-party interactive voice-and web-response system	**Definitely yes** Randomized, double-blind	**Definitely yes** Double-blind (participant, investigator)	**Definitely yes**	**Probably no** There were 15.6% (59/377) and 16.9% (32/189) patients in SGLT-2i and placebo groups with missing outcome data, respectively; missing outcome data were generally balanced across	**Definitely yes**	**Probably yes** Generally balanced baseline characteristics across groups

					treatment groups, with similar reasons for missing data across groups		
Rosenstock, 2015[55]	**Definitely yes** Using a third-party interactive voice and web response system	**Definitely yes** Randomized, double-blind	**Definitely yes** Double-blind (participant, investigator)	**Definitely yes**	**Definitely no** There were 25.3% (82/324) and 30.6% (52/170) patients in SGLT2i and control groups with missing outcome data, respectively; missing outcome data were generally balanced across treatment groups, with similar reasons for missing data across groups	**Definitely yes**	**Probably yes** Generally balanced baseline characteristics across groups
Ross, 2015[56]	**Definitely yes**	**Definitely yes**	**Probably yes**	**Probably yes**	**Probably yes**	**Definitely**	**Probably yes**

	Using a block allocation method	Randomized,			There were 7.2% (63/876) and 3.7% (4/107) patients in SGLT2i and control groups with missing outcome data, respectively; missing outcome data were generally balanced across treatment groups, with similar reasons for missing data across groups	**yes**	Generally balanced baseline characteristics across groups
Dagogo-Jack, 2018[57]	**Definitely yes** Using a computer-generated randomization schedule	**Definitely yes** Randomized, double-blind	**Definitely yes** Double-blind (participant, investigator)	**Definitely yes**	**Probably no** There were 11.3% (35/311) and 14.4% (22/153) patients in active agent and placebo	**Definitely yes**	**Probably yes** Generally balanced baseline characteristics across groups

					groups with missing outcome data, respectively; missing outcome data were generally balanced across treatment groups, with similar reasons for missing data across groups		
Terra,2017[58]	**Definitely yes** Using a computer-generated randomization code using the method of random permuted blocks	**Definitely yes** Randomized, double-blind	**Definitely yes** Double-blind (participant, investigator)	**Definitely yes**	**Definitely no** There were 14.0% (43/308) and 22.2% (34/153) patients in active agent and placebo groups with missing outcome data, respectively; missing outcome	**Definitely yes**	**Probably yes** Generally balanced baseline characteristics across groups

					data were generally balanced across treatment groups, with similar reasons for missing data across groups		
Bando,2016[59]	**Definitely yes**<br>Using a EDC system	**Definitely yes**<br>Randomized, open-label	**Definitely no**<br>open-label	**Definitely no**	**Definitely yes**<br>All patients complete the study protocol without any withdrawals	**Definitely yes**	**Probably yes**<br>Generally balanced baseline characteristics across groups
Han,2018[60]	**Definitely yes**<br>Using a computer-generated randomization schedule	**Definitely yes**<br>Randomized, double-blind	**Definitely yes**<br>Double-blind (participant, investigator)	**Definitely yes**	**Definitely no**<br>There were 24.3% (18/74) and 17.4% (12/69) patients in SGLT-2 agent and placebo groups with missing outcome data, respectively;	**Definitely yes**	**Probably yes**<br>Generally balanced baseline characteristics across groups

					missing outcome data were generally balanced across treatment groups, with similar reasons for missing data across groups		
Ishihara, 2016[61]	**Probably yes** Randomized, double-blind	**Probably yes** Randomized, double-blind	**Definitely yes** Double-blind (participant, investigator)	**Definitely yes**	**Probably yes** There were 3.4% (6/175) and 12.6% (11/87) patients in SGLT-2 agent and placebo groups with missing outcome data, respectively; missing outcome data were generally balanced across treatment groups,	**Definitely yes**	**Probably yes** Generally balanced baseline characteristics across groups

					with similar reasons for missing data across groups		
Kashiwagi, 2015[62]	**Definitely yes** Using a block allocation method	**Definitely yes** Randomized, double-blind	**Definitely yes** Double-blind (participant, investigator)	**Definitely yes**	**Probably yes** There were 10.1% (12/119) and 8.7% (4/46) patients in active agent and placebo groups with missing outcome data, respectively; missing outcome data were generally balanced across treatment groups, with similar reasons for missing data across groups	**Definitely yes**	**Probably yes** Generally balanced baseline characteristics across groups
Seino, 2018[63]	**Definitely yes** Using a	**Definitely yes** Randomized,	**Definitely yes**	**Definitely yes**	**Probably no** There were 1.9%	**Definitely yes**	**Probably yes** Generally

	computer-generated randomization schedule	double-blind	Double-blind (participant, investigator)		(3/159) and 13.5% (10/74) patients in SGLT-2 and placebo groups with missing outcome data, respectively; missing outcome data were generally balanced across treatment groups, with similar reasons for missing data across groups		balanced baseline characteristics across groups
Buse, 2018[64]	**Probably yes** Randomized, double-blind	**Probably yes** Randomized, double-blind	**Definitely yes** Double-blind (details not reported)	**Definitely yes**	**Probably no** There were 14.5% (76/525) and 18.7% (50/268) patients in SGLT2i and control groups with missing	**Definitely yes**	**Probably yes** Generally balanced baseline characteristics across groups

					outcome data, respectively; missing outcome data were generally balanced across treatment groups, with similar reasons for missing data across groups		
Kaku, 2014[65]	**Definitely yes** Using a central web-based system	**Definitely yes** Randomized, double-blind	**Definitely yes** Double-blind (participant, investigator)	**Definitely yes**	**Probably no** There were 7.3% (13/178) and 15.8% (9/57) patients in SGLT-2 and placebo groups with missing outcome data, respectively; missing outcome data were generally balanced across	**Definitely yes**	**Probably yes** Generally balanced baseline characteristics across groups

					treatment groups, with similar reasons for missing data across groups		
Terauchi, 2017[66]	**Definitely yes** Using an interactive web response system (IWRS)	**Definitely yes** Randomized, double-blind	**Definitely yes** Double-blind (participant, investigator)	**Definitely yes**	**Definitely yes** There were 2.8% (4/141) and 2.8% (2/70) patients in SGLT-2 and placebo groups with missing outcome data, respectively; missing outcome data were generally balanced across treatment groups, with similar reasons for missing data across groups	**Definitely yes**	**Probably yes** Generally balanced baseline characteristics across groups
Schernthaner 2013[67]	**Definitely yes** Using an interactive	**Definitely yes** Randomized,	**Definitely yes**	**Definitely yes**	**Definitely no** There were	**Definitely yes**	**Probably yes** Generally

	voice response system/interactive web response system	double-blind	Double-blind (participant, investigator)		32.8% (124/378) and 44.4% (168/378) patients in SGLT2i and control groups with missing outcome data, respectively; missing outcome data were generally balanced across treatment groups, with similar reasons for missing data across groups		balanced baseline characteristics across groups
Nauck 2011[68]	**Definitely yes** Using a predefined computer-generated randomization scheme	**Definitely yes** Randomized, double-blind	**Definitely yes** Double-blind (participant, investigator)	**Definitely yes**	**Definitely no** There were 20.7% (84/406) and 23.0% (94/408) patients in SGLT2i and control groups	**Definitely yes**	**Probably yes** Generally balanced baseline characteristics across groups

					with missing outcome data, respectively; missing outcome data were generally balanced across treatment groups, with similar reasons for missing data across groups		
Müller-Wieland 2018[69]	**Definitely yes** Using a computer-generated random sequence	**Definitely yes** Randomized, double-blind	**Definitely yes** Double-blind (participant, investigator)	**Definitely yes**	**Probably yes** There were 7.5% (47/626) and 8.0% (25/313) patients in SGLT2i and control groups with missing outcome data, respectively; missing outcome data were generally	**Definitely yes**	**Probably yes** Generally balanced baseline characteristics across groups

					balanced across treatment groups, with similar reasons for missing data across groups		
Cho 2018[70]	**Probably yes** Randomized	**Probably yes** Open-labeled	**Definitely no** Open-labeled (participant, investigator)	**Definitely no**	**Definitely yes** There were 0% (0/36) and 0% (0/35) patients in SGLT2i and control groups with missing outcome data, respectively; missing outcome data were generally balanced across treatment groups, with similar reasons for missing data across groups	**Definitely yes**	**Probably yes** Generally balanced baseline characteristics across groups
Scott 2018[71]	**Definitely yes**	**Definitely yes**	**Definitely**	**Definitely**	**Definitely yes**	**Definitely**	**Probably yes**

	Using an interactive voice response system	Randomized, double-blind	**yes** Double-blind (details not reported)	**yes**	There were 3.6% (11/307) and 2.6% (8/307) patients in SGLT2i and control groups with missing outcome data, respectively; missing outcome data were generally balanced across treatment groups, with similar reasons for missing data across groups	**yes**	Generally balanced baseline characteristics across groups
Shimizu 2018[72]	**Probably yes** Randomized	**Probably yes** Open-labeled	**Definitely no** Open-labeled (participant, investigator)	**Definitely no**	**Probably no** There were 5.7% (2/35) and 14.3% (4/28) patients in SGLT2i and control groups with missing	**Definitely yes**	**Probably yes** Generally balanced baseline characteristics across groups

					outcome data, respectively; missing outcome data were generally balanced across treatment groups, with similar reasons for missing data across groups		
Hayashi 2017[73]	**Probably yes** Randomized	**Probably yes** Open-labeled	**Definitely no** Open-labeled (participant, investigator)	**Definitely no**	**Definitely yes** There were 0% (0/40) and 0% (0/40) patients in SGLT2i and control groups with missing outcome data, respectively; missing outcome data were generally balanced across treatment groups,	**Definitely yes**	**Probably yes** Generally balanced baseline characteristics across groups

					with similar reasons for missing data across groups		
Zinman 2015[74]	**Definitely yes** Using a computer-generated random-sequence and interactive voice- and Web-response system	**Definitely yes** Randomized, double-blind	**Definitely yes** Double-blind (details not reported)	**Definitely yes**	**Definitely yes** There were 3.1% (144/4687) and 2.9% (67/2333) patients in SGLT2i and control groups with missing outcome data, respectively; missing outcome data were generally balanced across treatment groups, with similar reasons for missing data across groups	**Definitely yes**	**Probably yes** Generally balanced baseline characteristics across groups
Araki 2015[75]	**Definitely yes** Using a	**Definitely yes** Randomized	**Definitely no** Open-labeled	**Definitely no**	**Probably yes**	**Definitely yes**	**Probably yes** Generally

	computer-generated random sequence via an interactive web-based response system.		(participant, investigator)				balanced baseline characteristics across groups
Hollander 2019[76]	**Definitely yes** Using a computer-generated randomization schedule.	**Definitely yes** Randomized, double-blind	**Definitely yes** Double-blind (participant, investigator)	**Definitely yes**	**Definitely no** There were 22.9% (202/881) and 24.8% (108/435) patients in SGLT2i and control groups with missing outcome data, respectively; missing outcome data were generally balanced across treatment groups, with similar reasons for missing data across groups	**Definitely yes**	**Probably yes** Generally balanced baseline characteristics across groups

Tsurutani 2018[77]	**Probably yes** Randomized	**Probably yes** Open-labeled	**Definitely no** Open-labeled (details not reported)	**Definitely no**	**Definitely yes** There were 5% (3/60) and 3.4% (2/59) patients in SGLT2i and control groups with missing outcome data, respectively; missing outcome data were generally balanced across treatment groups, with similar reasons for missing data across groups	**Definitely yes**	**Probably yes** Generally balanced baseline characteristics across groups
Takashima,2018[78]	**Probably yes** Randomized	**Probably yes** Open-labeled	**Definitely no** Open-labeled (participant, investigator)	**Definitely no**	**Definitely yes** There were 4.8% (1/21) and 4.8% (1/21) patients in SGLT2i and control groups	**Definitely yes**	**Probably yes** Generally balanced baseline characteristics across groups

					with missing outcome data, respectively; missing outcome data were generally balanced across treatment groups, with similar reasons for missing data across groups		
Fioretto,2018[79]	**Definitely yes** Using an interactive voice response system (IVRS) or interactive web response system (IWRS)	**Definitely yes** Randomized, double-blind	**Definitely yes** Double-blind (participant, investigator)	**Definitely yes**	**Probably yes** There were 6.9% (11/160) and 9.3% (15/161) patients in SGLT-2 and placebo groups with missing outcome data, respectively; missing outcome data were generally	**Definitely yes**	**Probably yes** Generally balanced baseline characteristics across groups

					balanced across treatment groups, with similar reasons for missing data across groups		
Pollock, 2019[80]	**Definitely yes** Using the sponsor's interactive voice–web response system	**Definitely yes** Randomized, double-blind	**Definitely yes** Double-blind (participant, investigator)	**Definitely yes**	**Probably yes** There were 9.3% (14/151) and 6.5% (10/153) patients in SGLT2i and control groups with missing outcome data, respectively; missing outcome data were generally balanced across treatment groups, with similar reasons for missing data across groups	**Definitely yes**	**Probably yes** Generally balanced baseline characteristics across groups

Grunberger, 2017[81]	**Definitely yes** Using an interactive voice response system/integrated web response system	**Definitely yes** Randomized, double-blind	**Definitely yes** Double-blind (participant, investigator)	**Definitely yes**	**Probably no** There were 10.5% (33/314) and 11.0% (17/154) patients in active agent and placebo groups with missing outcome data, respectively; missing outcome data were generally balanced across treatment groups, with similar reasons for missing data across groups	**Definitely yes**	**Probably yes** Generally balanced baseline characteristics across groups
Haneda, 2016[82]	**Probably yes** Randomized, double-blind	**Probably yes** Randomized, double-blind	**Definitely yes** Double-blind (participant, investigator)	**Definitely yes**	**Probably yes** There were 6.3% (6/95) and 4% (2/50) patients in	**Definitely yes**	**Probably yes** Generally balanced baseline characteristics

					SGLT2i and control groups with missing outcome data, respectively; missing outcome data were generally balanced across treatment groups, with similar reasons for missing data across groups		across groups
Yale, 2013[83]	**Definitely yes** Using an interactive voice response system/interactive web response system.	**Definitely yes** Randomized, double-blind	**Definitely yes** Double-blind (details not reported)	**Definitely yes**	**Probably no** There were 12.3% (22/179) and 14.4% (13/90) patients in SGLT2i and control groups with missing outcome data, respectively; missing outcome	**Definitely yes**	**Probably yes** Generally balanced baseline characteristics across groups

					data were generally balanced across treatment groups, with similar reasons for missing data across groups		
Heerspink, 2020[84]	**Definitely yes** Using an interactive voice-response or Web-response system	**Definitely yes** Randomized, double-blind	**Definitely yes** Double-blind (participant, investigator)	**Definitely yes**	**Definitely yes** There were 0.47% (10/2152) and 0.23% (5/2152) patients in SGLT2i and control groups with missing outcome data, respectively; missing outcome data were generally balanced across treatment groups, with similar reasons for	**Definitely yes**	**Probably yes** Generally balanced baseline characteristics across groups

					missing data across groups		
Bhatt 2021[85]	**Probably yes** Randomized, double-blind	**Probably yes** Randomized, double-blind	**Definitely yes** Double-blind (participant, investigator)	**Definitely yes**	**Definitely yes** There were 1.1% (60/5292) and 1.5% (82/5292) patients in SGLT2i and control groups with missing outcome data, respectively; missing outcome data were generally balanced across treatment groups, with similar reasons for missing data across groups	**Definitely yes**	**Probably yes** Generally balanced baseline characteristics across groups
Kohan,2013[86]	**Probably yes** Randomized, double-blind	**Probably yes** Randomized, double-blind	**Definitely yes** Double-blind (details not	**Definitely yes**	**Definitely no** There were 42.9% (72/168) and 48.8%	**Definitely yes**	**Probably yes** Generally balanced baseline

	reported)	(41/84) patients in SGLT2i and placebo groups with missing outcome data, respectively; missing outcome data were generally balanced across treatment groups, with similar reasons for missing data across groups	characteristics across groups

Table S3. Subgroup analyses of efficacy among different drug categories.

Drug type	Renal function	Weighted mean difference	95%CI	P (over effect)	P (subgroup difference)
**HbA1c (%)**					
**CANA**	≥90	-0.77	-0.91, -0.64	＜0.001	
	60-89	-0.81	-0.93, -0.68	＜0.001	
	45-59	-0.20	-0.69, 0.29	0.42	
	＜45	NA	NA	NA	
	total	-0.79	-0.90, -0.68	＜0.001	0.06
**DAPA**	≥90	-0.49	-0.68, -0.30	＜0.001	
	60-89	-0.32	-0.45, -0.20	＜0.001	
	45-59	-0.27	-0.51, -0.04	0.02	
	＜45	-0.27	-0.97, 0.43	0.45	
	total	-0.36	-0.47, -0.25	＜0.001	0.42
**EMPA**	≥90	-0.32	-0.56, -0.07	0.01	
	60-89	-0.56	-0.65, -0.46	＜0.001	
	45-59	-0.44	-0.61, -0.27	＜0.001	
	＜45	0.48	-0.06, 1.02	0.08	
	total	-0.49	-0.57, -0.40	＜0.001	＜0.001
**ERTU**	≥90	-0.67	-0.85, -0.49	＜0.001	
	60-89	-0.60	-0.93, -0.26	＜0.001	
	45-59	-0.09	-0.24, 0.06	0.23	
	＜45	NA	NA	NA	
	total	-0.56	-0.76, -0.37	＜0.001	＜0.001
**IPRA**	≥90	-0.48	-0.69, -0.27	＜0.001	

	60-89	-0.80	-1.13, -0.48	＜0.001	
	45-59	NA	NA	NA	
	＜45	NA	NA	NA	
	total	-0.71	-0.99, -0.44	＜0.001	0.1
**SOTA**	≥90	-0.38	-0.52, -0.24	＜0.001	
	60-89	-0.28	-0.37, -0.19	＜0.001	
	45-59	NA	NA	NA	
	＜45	-0.43	-0.48, -0.38	＜0.001	
	total	-0.36	-0.43, -0.28	＜0.001	0.01
**LUSEO**	≥90	NA	NA	NA	
	60-89	-1.13	-1.34, -0.92	＜0.001	
	45-59	-0.2	-0.42, 0.02	0.08	
	＜45	NA	NA	NA	
	total	-0.67	-1.58, 0.25	0.15	＜0.001
**TOFO**	≥90	NA	NA	NA	
	60-89	-0.91	-1.03, -0.78	＜0.001	
	45-59	NA	NA	NA	
	＜45	NA	NA	NA	
	total	-0.91	-1.03, -0.78	＜0.001	NA
**Weight (kg)**					
**CANA**	≥90	-2.5	-3.05, -1.95	＜0.001	
	60-89	-1.73	-2.05, -1.42	＜0.001	
	45-59		NA	NA	
	＜45		NA	NA	
	total	-1.86	-2.22, -1.50	＜0.001	0.02

**DAPA**	≥90	-2.07	-3.43, -0.71	0.003	
	60-89	-1.97	-2.75, -1.19	＜0.001	
	45-59	-1.25	-2.08, -0.42	0.003	
	＜45	-3.35	-4.86, -1.85	＜0.001	
	total	-2.04	-2.74, -1.34	＜0.001	0.11
**EMPA**	≥90	-2.17	-2.64, -1.69	＜0.001	
	60-89	-1.93	-2.11, -1.75	＜0.001	
	45-59	-1.17	-1.75, -0.59	＜0.001	
	＜45	-1.00	-2.57, 0.57	0.21	
	total	-1.94	-2.11, -1.77	＜0.001	0.03
**ERTU**	≥90	-1.88	-2.12, -1.64	＜0.001	
	60-89	-2.56	-3.36, -1.75	＜0.001	
	45-59	NA	NA	NA	
	＜45	NA	NA	NA	
	total	-2.27	-2.74, -1.80	＜0.001	0.12
**IPRA**	≥90	-1.83	-3.12,	0.006	
	60-89	-1.66	-2.16, -1.15	＜0.001	
	45-59	NA	NA	NA	
	＜45	NA	NA	NA	
	total	-1.68	-2.11, -1.25	＜0.001	0.81
**SOTA**	≥90	-2.83	-3.14, -2.51	＜0.001	
	60-89	-3.73	-4.89, -2.57	＜0.001	
	45-59	NA	NA	NA	
	＜45	-1.16	-1.27, -1.05	＜0.001	
	total	-2.73	-3.82, -1.65	＜0.001	＜0.001

**LUSEO**	≥90	NA	NA	NA	
	60-89	-1.27	-1.69, -0.85	＜0.001	
	45-59	-1.27	-1.84, -0.7	＜0.001	
	＜45	NA	NA	NA	
	total	-1.27	-1.61, -0.93	＜0.001	1.00
**TOFO**	≥90	NA	NA	NA	
	60-89	-2.06	-2.69, -1.42	＜0.001	
	45-59	NA	NA	NA	
	＜45	NA	NA	NA	
	total	-2.06	-2.69, -1.42	＜0.001	NA
**SBP (mmHg)**					
**CANA**	≥90	-4.61	-5.77, -3.45	＜0.001	
	60-89	-4.58	-5.49, -3.67	＜0.001	
	45-59	-3.60	-4.90, -2.30	＜0.001	
	＜45	-5.95	-8.89, -3.01	＜0.001	
	total	-4.51	-5.18, -3.84	＜0.001	0.43
**DAPA**	≥90	-3.49	-5.24, -1.74	＜0.001	
	60-89	-2.75	-3.94, -1.56	＜0.001	
	45-59	-2.97	-6.09, 0.15	0.06	
	＜45	-5.61	-8.97, -2.24	0.001	
	total	-3.17	-4.37, -1.97	＜0.001	0.45
**EMPA**	≥90	-3.61	-4.72, -2.50	＜0.001	
	60-89	-3.84	-4.67, -3.02	＜0.001	
	45-59	-4.30	-7.13, -1.47	0.003	
	＜45	-12.20	-19.75, -4.65	0.002	

	total	-3.87	-4.55, -3.20	＜0.001	0.17
**ERTU**	≥90	-3.80	-4.77, -2.83	＜0.001	
	60-89	-3.84	-4.69, -2.98	＜0.001	
	45-59	NA	NA	NA	
	＜45	NA	NA	NA	
	total	-3.82	-4.46, -3.18	＜0.001	0.96
**IPRA**	≥90	-5.5	-9.56, -1.44	0.008	
	60-89	-2.23	-3.97, -0.50	0.01	
	45-59	NA	NA	NA	
	＜45	NA	NA	NA	
	total	-2.74	-4.33, -1.14	＜0.001	0.15
**SOTA**	≥90	-3.45	-4.36, -2.55	＜0.001	
	60-89	-3.59	-5.04, -2.15	＜0.001	
	45-59	NA	NA	NA	
	＜45	-2.44	-2.84, -2.04	＜0.001	
	total	-2.67	-3.02, -2.31	＜0.001	0.06
**LUSEO**	≥90	NA	NA	NA	
	60-89	-4.1	-7.48, -0.72	0.02	
	45-59	-2.6	-7.91, 2.71	0.34	
	＜45	NA	NA	NA	
	total	-3.67	-6.52, -0.82	0.01	0.64
**TOFO**	≥90	NA	NA	NA	
	60-89	-3.88	-6.04, -1.72	＜0.001	
	45-59	NA	NA	NA	
	＜45	NA	NA	NA	

	total	-3.88	-6.04, -1.72	＜0.001	NA
**DBP (mmHg)**					
**CANA**	≥90	-1.74	-2.37, -1.12	＜0.001	
	60-89	-1.83	-2.19, -1.47	＜0.001	
	45-59	-2.20	-3.01, -1.39	＜0.001	
	＜45	-1.65	-3.41, 0.11	0.07	
	total	-1.86	-2.14, -1.57	＜0.001	0.83
**DAPA**	≥90	-1.80	-2.02, -1.59	＜0.001	
	60-89	-0.73	-1.45, -0.01	0.05	
	45-59	NA	NA	NA	
	＜45	-0.47	-2.62, 1.68	0.67	
	total	-1.70	-1.91, -1.50	＜0.001	0.01
**EMPA**	≥90	-2.17	-2.88, -1.46	＜0.001	
	60-89	-1.70	-2.02, -1.38	＜0.001	
	45-59	-1.50	-3.20, 0.20	0.08	
	＜45	-5.70	-9.69, -1.71	0.005	
	total	-1.79	-2.08, -1.51	＜0.001	0.16
**ERTU**	≥90	-1.23	-1.83, -0.63	＜0.001	
	60-89	-1.36	-1.91, -0.81	＜0.001	
	45-59	NA	NA	NA	
	＜45	NA	NA	NA	
	total	-1.30	-1.70, -0.89	＜0.001	0.76
**IPRA**	≥90	-2.7	-5.32, -0.08	0.04	
	60-89	-1.10	-2.33, 0.14	0.08	
	45-59	NA	NA	NA	

	＜45	NA	NA	NA	
	total	-1.39	-2.51, -0.27	0.01	0.28
**SOTA**	≥90	-1.20	-1.81, -0.59	＜0.001	
	60-89	-1.90	-2.80, -1.00	＜0.001	
	45-59	NA	NA	NA	
	＜45	-0.80	-1.05, -0.55	＜0.001	
	total	-0.92	-1.15, -0.70	＜0.001	0.04
**LUSEO**	≥90	NA	NA	NA	
	60-89	-2.50	-4.84, -0.16	0.04	
	45-59	0.30	-3.30, 3.90	0.87	
	＜45	NA	NA	NA	
	total	-1.67	-3.63, 0.30	0.10	0.20
**TOFO**	≥90	NA	NA	NA	
	60-89	-2.76	-4.28, -1.24	＜0.001	
	45-59	NA	NA	NA	
	＜45	NA	NA	NA	
	total	-2.76	-4.28, -1.24	＜0.001	NA
**eGFR (mL/min/1.73 m^2)**					
**CANA**	≥90	-1.78	-5.90, 2.33	0.40	
	60-89	NA	NA	NA	
	45-59	4.10	0.75, 7.45	0.02	
	＜45	NA	NA	NA	
	total	0.12	-4.29, 4.54	0.96	0.03
**DAPA**	≥90	-1.99	-4.24, 0.27	0.08	
	60-89	0.05	-1.16, 1.25	0.94	

	45-59	NA	NA	NA	
	＜45	0.88	0.47, 1.29	＜0.001	
	total	-0.35	-1.24, 0.55	0.45	0.03
**EMPA**	≥90	1.15	-0.17, 2.46	0.09	
	60-89	-0.33	-0.90, 0.24	0.26	
	45-59	-2.50	-4.08, -0.92	0.002	
	＜45	-0.30	-2.99, 2.39	0.83	
	total	-0.26	-0.78, 0.26	0.33	0.007
**ERTU**	≥90	1.54	-0.43, 3.52	0.12	
	60-89	NA	NA	NA	
	45-59	NA	NA	NA	
	＜45	NA	NA	NA	
	total	1.54	-0.43, 3.52	0.12	NA
**IPRA**	≥90	8.42	-5.15, 21.99	0.22	
	60-89	-1.21	-2.44, 0.01	0.05	
	45-59	NA	NA	NA	
	＜45	NA	NA	NA	
	total	-1.14	-2.35, 0.08	0.07	0.17
**SOTA**	≥90	-0.78	-1.82, 0.25	0.14	
	60-89	-1.25	-2.66, 0.16	0.08	
	45-59	NA	NA	NA	
	＜45	-1.25	-1.51, -0.99	＜0.001	
	total	-1.22	-1.47, -0.97	＜0.001	0.69
**LUSEO**	≥90	NA	NA	NA	
	60-89	-1.30	-3.78, 1.18	0.30	

	45-59	-2.50	-4.30, -0.70	0.007	
	＜45	NA	NA	NA	
	total	-2.09	-3.54, -0.63	0.005	0.44
**TOFO**	≥90	NA	NA	NA	
	60-89	-2.87	-4.84, -0.89	0.004	
	45-59	NA	NA	NA	
	＜45	NA	NA	NA	
	total	-2.87	-4.84, -0.89	0.004	NA

HbA1c, glycosylated hemoglobin; SBP, systolic blood pressure; DBP, diastolic blood pressure; eGFR, estimated glomerular filtrationrate; CANA, canagliflozin; DAPA, dapagliflozin; EMPA, empagliflozin; ERTU, ertugliflozin; IPRA, ipragliflozin; LUSEO, luseogliflozin; SOTA, sotagliflozin; TOFO, tofogliflozin; NA, not available.

Table S4. Subgroup analyses of efficacy among follow-up period.

Follow-up period	Renal function	Weighted mean difference	95%CI	P (over effect)	P (subgroup difference)
**HbA1c (%)**					
**＜1y**	≥90	-0.59	-0.68, -0.50	＜0.001	
	60-89	-0.70	-0.79, -0.62	＜0.001	
	45-59	-0.16	-0.27, -0.05	0.005	
	＜45				
	total	-0.65	-0.71, -0.58	＜0.001	＜0.001
**＞1y**	≥90	-0.13	-0.28, 0.02	0.08	
	60-89	-0.26	-0.36, -0.16	＜0.001	
	45-59	-0.41	-0.57, -0.25	＜0.001	
	＜45	-0.13	-0.67, 0.42	0.65	
	total	-0.23	-0.32, -0.15	＜0.001	0.09
**Weight (kg)**					
**＜1y**	≥90	-2.09	-2.72, -1.47	＜0.001	
	60-89	-1.74	-1.89, -1.60	＜0.001	
	45-59	-1.26	-1.73, -0.8	＜0.001	
	＜45				
	total	-1.86	-2.17, -1.56	＜0.001	0.08
**＞1y**	≥90	-2.24	-2.86, -1.62	＜0.001	
	60-89	-2.67	-3.40, -1.94	＜0.001	
	45-59	-1.17	-1.75, -0.59	＜0.001	
	＜45	-1.88	-3.04, -0.72	0.001	
	total	-2.47	-3.01, -1.93	＜0.001	0.009

**SBP (mmHg)**					
**＜1y**	≥90	-3.95	-4.83, -3.06	＜0.001	
	60-89	-3.73	-4.23, -3.24	＜0.001	
	45-59	-2.87	-5.56, -0.19	0.04	
	＜45	-5.95	-8.89, -3.01	＜0.001	
	total	-3.87	-4.39, -3.36	＜0.001	0.44
**＞1y**	≥90	-3.68	-4.49, -2.87	＜0.001	
	60-89	-3.82	-4.75, -2.88	＜0.001	
	45-59	-3.72	-4.91, -2.54	＜0.001	
	＜45	-5.32	-8.96, -1.67	0.004	
	total	-3.79	-4.39, -3.19	＜0.001	0.86
**DBP (mmHg)**					
**＜1y**	≥90	-1.76	-1.95, -1.57	＜0.001	
	60-89	-1.52	-1.76, -1.27	＜0.001	
	45-59	0.30	-3.30, 3.90	0.87	
	＜45	-1.65	-3.41, 0.11	0.07	
	total	-1.66	-1.81, -1.52	＜0.001	0.33
**＞1y**	≥90	-1.46	-1.99, -0.94	＜0.001	
	60-89	-1.90	-2.31, -1.49	＜0.001	
	45-59	-2.07	-2.80, -1.34	＜0.001	
	＜45	-1.30	-2.95, 0.35	0.12	
	total	-1.71	-2.06, -1.37	＜0.001	0.44
**eGFR (mL/min/1.73 m^2)**					
**＜1y**	≥90	-0.19	-1.37, 0.98	0.75	
	60-89	-0.82	-1.27, -0.38	＜0.001	

	45-59	-2.50	-4.30, -0.70	0.007	
	＜45	NA	NA	NA	
	total	-0.77	-1.22, -0.32	＜0.001	0.11
**＞1y**	≥90	-1.02	-2.68, 0.63	0.23	
	60-89	0.24	-0.59, 1.08	0.57	
	45-59	0.63	-5.83, 7.09	0.85	
	＜45	-0.21	-1.77, 1.34	0.79	
	total	-0.06	-0.70, 0.58	0.85	0.60

HbA1c, glycosylated hemoglobin; SBP, systolic blood pressure; DBP, diastolic blood pressure; eGFR, estimated glomerular filtrationrate.

Table S5. Subgroup analyses of different AEs.

AEs	Renal function	OR	95%CI	P (over effect)	P (subgroup difference)
**urinary tract infection**	≥90	1.18	0.99, 1.39	0.06	
	60-89	1.06	0.97, 1.15	0.22	
	45-59	0.92	0.61, 1.38	0.68	
	＜45	1.05	0.94, 1.19	0.38	
	total	1.07	1.00, 1.14	0.04	0.60
**genital tract infection**	≥90	3.67	2.85, 4.73	＜0.001	
	60-89	3.95	3.31, 4.71	＜0.001	
	45-59	3.24	1.24, 8.46	0.02	
	＜45	2.83	2.04, 3.93	＜0.001	
	total	3.69	3.23, 4.20	＜0.001	0.37
**amputation**	≥90	1.48	0.06, 36.49	0.81	
	60-89	1.19	0.68, 2.09	0.54	
	45-59	3.08	0.12, 76.30	0.49	
	＜45	0.93	0.66, 1.30	0.67	
	total	1.01	0.76, 1.34	0.96	0.78
**hypovolemia**	≥90	1.65	1.08, 2.53	0.02	
	60-89	1.12	0.99, 1.26	0.06	
	45-59	1.65	0.82, 3.30	0.16	
	＜45	1.37	1.18, 1.60	＜0.001	
	total	1.24	1.13, 1.35	＜0.001	0.07
**orthostatic hypotension**	≥90	2.77	0.30, 25.75	0.37	
	60-89	1.23	0.54, 3.76	0.62	

	45-59	NA	NA	NA	
	＜45	1.52	0.06, 37.71	0.80	
	total	1.38	0.66, 2.89	0.39	0.80
**bone fracture**	≥90	0.59	0.29, 1.18	0.13	
	60-89	0.98	0.82, 1.17	0.81	
	45-59	0.36	0.11, 1.13	0.08	
	＜45	1.10	0.90, 1.35	0.35	
	total	0.99	0.87, 1.13	0.93	0.10
**DKA**	≥90	6.47	2.49,16.83	＜0.001	
	60-89	2.78	1.51, 5. 14	0.001	
	45-59	3.08	0.12, 76.30	0.49	
	＜45	1.27	0.80, 2.03	0.31	
	total	2.23	1.59, 3.11	＜0.001	0.01
**hypoglycemia**	≥90	1.07	0.57, 2.04	0.83	
	60-89	0.96	0.74, 1.23	0.72	
	45-59	0.94	0.69, 1.27	0.67	
	＜45	0.79	0.57, 1.11	0.17	
	total	0.96	0.78, 1.18	0.69	0.78

AEs, adverse effect; OR, odds ratio; DKA, diabetic ketoacidosis.

Table S6. Subgroup analyses of different AEs in placebo-controlled RCTs.

AEs	renal function	OR	95%CI	P (over effect)	P (subgroup difference)
**urinary tract infection**	⩾90	1.12	0.92, 1.36	0.25	
	60-89	1.03	0.94, 1.13	0.53	
	45-59	0.92	0.61, 1.38	0.68	
	＜45	1.05	0.94, 1.19	0.38	
	total	1.05	0.98, 1.12	0.20	0.80
**genital tract infection**	⩾90	3.27	2.47, 4.34	＜0.001	
	60-89	3.50	2.88, 4.25	＜0.001	
	45-59	3.24	1.24, 8.46	0.02	
	＜45	2.83	2.04, 3.93	＜0.001	
	total	3.32	2.88, 3.82	＜0.001	0.75
**amputation**	⩾90	1.48	0.06, 36.49	0.81	
	60-89	1.19	0.68, 2.09	0.54	
	45-59	3.08	0.12, 76.30	0.49	
	＜45	0.93	0.66, 1.30	0.67	
	total	1.01	0.76, 1.34	0.96	0.78
**hypovolemia**	⩾90	2.15	1.27, 3.64	0.005	
	60-89	1.10	0.98, 1.25	0.11	
	45-59	1.65	0.82, 3.30	0.16	
	＜45	1.37	1.18, 1.60	＜0.001	
	total	1.24	1.13, 1.35	＜0.001	0.02
**orthostatic hypotension**	⩾90	2.77	0.30, 25.75	0.37	
	60-89	1.28	0.52, 3.13	0.59	

	45-59	NA	NA	NA	
	＜45	1.52	0.06, 37.71	0.80	
	total	1.45	0.65, 3.23	0.36	0.82
**bone fracture**	⩾90	0.62	0.30, 1.26	0.19	
	60-89	0.97	0.81, 1.16	0.72	
	45-59	0.36	0.11, 1.14	0.08	
	＜45	1.13	0.75, 1.71	0.54	
	total	0.98	0.86, 1.12	0.78	0.18
**DKA**	⩾90	6.20	2.27, 16.91	＜0.001	
	60-89	2.44	1.26, 4.72	0.008	
	45-59	3.08	0.12, 76.30	0.49	
	＜45	1.00	0.33, 3.02	1.00	
	total	2.14	1.27, 3.61	0.004	0.12
**hypoglycemia**	⩾90	1.57	1.29, 1.91	＜0.001	
	60-89	1.07	0.98, 1.16	0.14	
	45-59	0.94	0.74, 1.19	0.60	
	＜45	0.80	0.62, 1.05	0.11	
	total	1.09	1.02, 1.17	0.02	＜0.001

AEs, adverse effect; RCTs, randomized controlled trials; OR, odds ratio; DKA, diabetic ketoacidosis.

Table S7. Efficacy of SGLT2i treatment in patients with different levels of renal function in placebo-controlled RCTs.

**Efficacy outcomes**	**Renal function**	**Mean difference**	**95%CI**	**P (over effect)**	**P (subgroup difference)**
HbA1c (%)	⩾90	-0.58	-0.67, -0.50	＜0.001	
	60-89	-0.67	-0.74, -0.60	＜0.001	
	45-59	-0.22	-0.35, -0.09	＜0.001	
	＜45	-0.13	-0.67, 0.42	0.65	
	total	-0.61	-0.67, -0.56	＜0.001	＜0.001
Weight (kg)	⩾90	-2.07	-2.31, -1.83	＜0.001	
	60-89	-1.81	-1.98, -1.64	＜0.001	
	45-59	-1.23	-1.59, -0.86	＜0.001	
	＜45	-1.88	-3.04, -0.72	0.001	
	total	-1.85	-1.99, -1.70	＜0.001	0.002
SBP (mmHg)	⩾90	-3.62	-4.09, -3.15	＜0.001	
	60-89	-3.67	-4.16, -3.17	＜0.001	
	45-59	-3.58	-4.67, -2.50	＜0.001	
	＜45	-5.36	-7.98, -2.74	＜0.001	
	total	-3.64	-3.99, -3.30	＜0.001	0.65
DBP (mmHg)	⩾90	-1.45	-1.75, -1.14	＜0.001	
	60-89	-1.58	-1.79, -1.36	＜0.001	
	45-59	-1.98	-2.69, -1.26	＜0.001	
	＜45	-0.83	-1.08, -0.58	＜0.001	
	total	-1.32	-1.46, -1.18	＜0.001	＜0.001
eGFR (mL/min/1.73	⩾90	-0.45	-1.39,0.50	0.35	

m^{2})					
	60-89	-0.54	-1.00, -0.09	0.02	
	45-59	-0.68	-3.80, 2.45	0.67	
	＜45	-0.21	-1.77, -1.34	0.79	
	total	-0.54	-0.94, -0.14	0.008	0.98

HbA1c, glycosylated hemoglobin; SBP, systolic blood pressure; DBP, diastolic blood pressure; eGFR, estimated glomerular filtration rate.

Table S8. Efficacy of SGLT2i treatment in patients with different levels of renal function (follow-up period ≥12 weeks)

Efficacy outcomes	Renal function	Mean difference	95%CI	P (over effect)	P(subgroup difference)
HbA1c (%)	≥90	-0.49	-0.59,-0.39	＜0.001	
	60-89	-0.58	-0.66,-0.5	＜0.001	
	45-59	-0.22	-0.35,-0.09	＜0.001	
	＜45	-0.13	-0.67,0.42	0.65	
	total	-0.53	-0.59,-0.47	＜0.001	＜0.001
Weight (kg)	≥90	-2.13	-2.66,-1.59	＜0.001	
	60-89	-2.06	-2.31,-1.82	＜0.001	
	45-59	-1.23	-1.59,-0.86	＜0.001	
	＜45	-1.88	-3.04,-0.72	0.001	
	total	-2.05	-2.32,-1.79	＜0.001	0.002
SBP (mmHg)	≥90	-3.83	-4.57,-3.09	＜0.001	
	60-89	-3.79	-4.24,-3.34	＜0.001	
	45-59	-3.58	-4.67,-2.5	＜0.001	
	＜45	-5.36	-7.98,-2.74	＜0.001	
	total	-3.86	-4.29,-3.42	＜0.001	0.68
DBP (mmHg)	≥90	-1.72	-1.9,-1.55	＜0.001	
	60-89	-1.63	-1.83,-1.43	＜0.001	
	45-59	-1.98	-2.69,-1.26	＜0.001	
	＜45	-0.83	-1.08,-0.58	＜0.001	
	total	-1.5	-1.62,-1.39	＜0.001	＜0.001
eGFR (mL/min/1.73 m^2)	≥90	-0.43	-1.4,0.54	0.38	
	60-89	-0.46	-0.93,0	0.05	

	45-59	-0.68	-3.8,2.45	0.67	
	＜45	-0.21	-1.77,1.34	0.79	
	total	-0.48	-0.87,-0.08	0.02	0.99

HbA1c, glycosylated hemoglobin; SBP, systolic blood pressure; DBP, diastolic blood pressure; eGFR, estimated glomerular filtration rate.

Reference

1. Cusi K, Bril F, Barb D, et al. Effect of canagliflozin treatment on hepatic triglyceride content and glucose metabolism in patients with type 2 diabetes[J]. Diabetes Obes Metab, 2019, 21(4): 812-821.DOI:10.1111/dom.13584.
2. Ji L, Han P, Liu Y, et al. Canagliflozin in Asian patients with type 2 diabetes on metformin alone or metformin in combination with sulphonylurea[J]. Diabetes Obes Metab, 2015, 17(1): 23-31.DOI:10.1111/dom.12385.
3. Lavalle-González FJ, Januszewicz A, Davidson J, et al. Efficacy and safety of canagliflozin compared with placebo and sitagliptin in patients with type 2 diabetes on background metformin monotherapy: a randomised trial[J]. Diabetologia, 2013, 56(12): 2582-2592.
4. Wilding JP, Charpentier G, Hollander P, et al. Efficacy and safety of canagliflozin in patients with type 2 diabetes mellitus inadequately controlled with metformin and sulphonylurea: a randomised trial[J]. Int J Clin Pract, 2013, 67(12): 1267-1282.
5. Henry RR, Strange P, Zhou R, et al. Effects of Dapagliflozin on 24-Hour Glycemic Control in Patients with Type 2 Diabetes: A Randomized Controlled Trial[J]. Diabetes Technol Ther, 2018, 20(11): 715-724
6. Jabbour SA, Frías JP, Hardy E, et al. Safety and Efficacy of Exenatide Once Weekly Plus Dapagliflozin Once Daily Versus Exenatide or Dapagliflozin Alone in Patients With Type 2 Diabetes Inadequately Controlled With Metformin Monotherapy: 52-Week Results of the DURATION-8 Randomized Controlled Trial[J]. Diabetes Care, 2018, 41(10): 2136-2146
7. Ji L, Ma J, Li H, et al. Dapagliflozin as monotherapy in drug-naive Asian patients with type 2 diabetes mellitus: a randomized, blinded, prospective phase III study[J]. Clin Ther, 2014, 36(1): 84-100 e109.DOI:10.1016/j.clinthera.2013.11.002.
8. Mathieu C, Ranetti AE, Li D, et al. Randomized, Double-Blind, Phase 3 Trial of Triple Therapy With Dapagliflozin Add-on to Saxagliptin Plus Metformin in Type 2 Diabetes[J]. Diabetes Care, 2015, 38(11): 2009-2017.DOI:10.2337/dc15-0779.
9. Rosenstock J, Hansen L, Zee P, et al. Dual add-on therapy in type 2 diabetes poorly controlled with metformin monotherapy: a randomized double-blind trial of saxagliptin plus dapagliflozin addition versus single addition of saxagliptin or dapagliflozin to metformin[J]. Diabetes Care, 2015, 38(3): 376-383.DOI:10.2337/dc14-1142.
10. Wilding JP, Woo V, Soler NG, et al. Long-term efficacy of dapagliflozin in patients with type 2 diabetes mellitus receiving high doses of insulin: a randomized trial[J]. Ann Intern Med, 2012, 156(6): 405-415.DOI:10.7326/0003-4819-156-6-201203200-00003.
11. Ferdinand KC, Izzo JL, Lee J, et al. Antihyperglycemic and Blood Pressure Effects of Empagliflozin in Black Patients With Type 2 Diabetes Mellitus and Hypertension[J]. Circulation, 2019, 139(18): 2098-2109.DOI:10.1161/circulationaha.118.036568..

12. Hadjadj S, Rosenstock J, Meinicke T, et al. Initial Combination of Empagliflozin and Metformin in Patients With Type 2 Diabetes[J]. Diabetes Care, 2016, 39(10): 1718-1728.DOI:10.2337/dc16-0522.
13. Lewin A, DeFronzo RA, Patel S, et al. Initial combination of empagliflozin and linagliptin in subjects with type 2 diabetes[J]. Diabetes Care, 2015, 38(3): 394-402.DOI:10.2337/dc14-2365.
14. Ji L, Liu Y, Miao H, et al. Safety and efficacy of ertugliflozin in Asian patients with type 2 diabetes mellitus inadequately controlled with metformin monotherapy: VERTIS Asia[J]. Diabetes Obes Metab, 2019, 21(6): 1474-1482.
15. Pratley RE, Eldor R, Raji A, et al. Ertugliflozin plus sitagliptin versus either individual agent over 52 weeks in patients with type 2 diabetes mellitus inadequately controlled with metformin: The VERTIS FACTORIAL randomized trial[J]. Diabetes Obes Metab, 2018, 20(5): 1111-1120.
16. Rosenstock J, Frias J, Páll D, et al. Effect of ertugliflozin on glucose control, body weight, blood pressure and bone density in type 2 diabetes mellitus inadequately controlled on metformin monotherapy (VERTIS MET)[J]. Diabetes Obes Metab, 2018, 20(3): 520-529.DOI:10.1111/dom.13103.
17. Inoue H, Morino K, Ugi S, et al. Ipragliflozin, a sodium-glucose cotransporter 2 inhibitor, reduces bodyweight and fat mass, but not muscle mass, in Japanese type 2 diabetes patients treated with insulin: A randomized clinical trial[J]. J Diabetes Investig, 2019, 10(4): 1012-1021.
18. Lu CH, Min KW, Chuang LM, et al. Efficacy, safety, and tolerability of ipragliflozin in Asian patients with type 2 diabetes mellitus and inadequate glycemic control with metformin: Results of a phase 3 randomized, placebo-controlled, double-blind, multicenter trial[J]. J Diabetes Investig, 2016, 7(3): 366-373.
19. Danne T, Cariou B, Banks P, et al. HbA(1c) and Hypoglycemia Reductions at 24 and 52 Weeks With Sotagliflozin in Combination With Insulin in Adults With Type 1 Diabetes: The European inTandem2 Study[J]. Diabetes Care, 2018, 41(9): 1981-1990.DOI:10.2337/dc18-0342.
20. Garg SK, Henry RR, Banks P, et al. Effects of Sotagliflozin Added to Insulin in Patients with Type 1 Diabetes[J]. N Engl J Med, 2017, 377(24): 2337-2348.DOI:10.1056/NEJMoa1708337.
21. Zambrowicz B, Freiman J, Brown PM, et al. LX4211, a dual SGLT1/SGLT2 inhibitor, improved glycemic control in patients with type 2 diabetes in a randomized, placebo-controlled trial[J]. Clin Pharmacol Ther, 2012, 92(2): 158-169.
22. Leiter LA, Yoon KH, Arias P, Langslet G, Xie J, Balis DA, et al. Canagliflozin provides durable glycemic improvements and body weight reduction over 104 weeks versus glimepiride in patients with type 2 diabetes on metformin: a randomized, double-blind, phase 3 study[J].

Diabetes Care, 2015, 38(3): 355-364.DOI:10.2337/dc13-2762.

23. Handelsman Y, Mathieu C, Del Prato S, Johnsson E, Kurlyandskaya R, Iqbal N, et al. Sustained 52-week efficacy and safety of triple therapy with dapagliflozin plus saxagliptin versus dual therapy with sitagliptin added to metformin in patients with uncontrolled type 2 diabetes[J]. Diabetes Obes Metab, 2019, 21(4): 883-892.
24. Jeon HJ, Ku EJ, Oh TK. Dapagliflozin improves blood glucose in diabetes on triple oral hypoglycemic agents having inadequate glucose control[J]. Diabetes Res Clin Pract, 2018, 142: 188-194.DOI:10.1016/j.diabres.2018.05.013.
25. Ferrannini E, Berk A, Hantel S, Pinnetti S, Hach T, Woerle HJ, et al. Long-term safety and efficacy of empagliflozin, sitagliptin, and metformin: an active-controlled, parallel-group, randomized, 78-week open-label extension study in patients with type 2 diabetes[J]. Diabetes Care, 2013, 36(12): 4015-4021.
26. Miller S, Krumins T, Zhou H, Huyck S, Johnson J, Golm G, et al. Ertugliflozin and Sitagliptin Co-initiation in Patients with Type 2 Diabetes: The VERTIS SITA Randomized Study[J]. Diabetes Ther, 2018, 9(1): 253-268.
27. Bode B, Stenlöf K, Harris S, et al. Long-term efficacy and safety of canagliflozin over 104 weeks in patients aged 55-80 years with type 2 diabetes[J]. Diabetes Obes Metab, 2015, 17(3): 294-303.DOI:10.1111/dom.12428.
28. Forst T, Guthrie R, Goldenberg R, et al. Efficacy and safety of canagliflozin over 52 weeks in patients with type 2 diabetes on background metformin and pioglitazone[J]. Diabetes Obes Metab, 2014, 16(5): 467-477.
29. Inagaki N, Kondo K, Yoshinari T, et al. Efficacy and safety of canagliflozin in Japanese patients with type 2 diabetes: a randomized, double-blind, placebo-controlled, 12-week study[J]. Diabetes Obes Metab, 2013, 15(12): 1136-1145.
30. Inagaki N, Kondo K, Yoshinari T, et al. Efficacy and safety of canagliflozin monotherapy in Japanese patients with type 2 diabetes inadequately controlled with diet and exercise: a 24-week, randomized, double-blind, placebo-controlled, Phase III study[J]. Expert Opin Pharmacother, 2014, 15(11): 1501-1515.DOI:10.1517/14656566.2014.935764.
31. Inagaki N, Harashima S, Maruyama N, et al. Efficacy and safety of canagliflozin in combination with insulin: a double-blind, randomized, placebo-controlled study in Japanese patients with type 2 diabetes mellitus[J]. Cardiovasc Diabetol, 2016, 15: 89.
32. Kadowaki T, Inagaki N, Kondo K, et al. Efficacy and safety of canagliflozin as add-on therapy to teneligliptin in Japanese patients with type 2 diabetes mellitus: Results of a 24-week, randomized, double-blind, placebo-controlled trial[J]. Diabetes Obes Metab, 2017, 19(6): 874-882.
33. Rosenstock J, Chuck L, González-Ortiz M, et al. Initial Combination Therapy With Canagliflozin Plus Metformin Versus Each Component

as Monotherapy for Drug-Naïve Type 2 Diabetes[J]. Diabetes Care, 2016, 39(3): 353-362.DOI:10.2337/dc15-1736.
34. Stenlöf K, Cefalu WT, Kim KA, et al. Efficacy and safety of canagliflozin monotherapy in subjects with type 2 diabetes mellitus inadequately controlled with diet and exercise[J]. Diabetes Obes Metab, 2013, 15(4): 372-382.
35. Araki E, Onishi Y, Asano M, et al. Efficacy and safety of dapagliflozin over 1 year as add-on to insulin therapy in Japanese patients with type 2 diabetes: the DAISY (Dapagliflozin Added to patients under InSulin therapY) trial[J]. Diabetes Obes Metab, 2017, 19(4): 562-570.DOI:10.1111/dom.12853.
36. Bailey CJ, Morales Villegas EC, Woo V, et al. Efficacy and safety of dapagliflozin monotherapy in people with Type 2 diabetes: a randomized double-blind placebo-controlled 102-week trial[J]. Diabet Med, 2015, 32(4): 531-541.DOI:10.1111/dme.12624.
37. Bolinder J, Ljunggren Ö, Kullberg J, et al. Effects of dapagliflozin on body weight, total fat mass, and regional adipose tissue distribution in patients with type 2 diabetes mellitus with inadequate glycemic control on metformin[J]. J Clin Endocrinol Metab, 2012, 97(3): 1020-1031.DOI:10.1210/jc.2011-2260.
38. McMurray JJV, Solomon SD, Inzucchi SE, et al. Dapagliflozin in Patients with Heart Failure and Reduced Ejection Fraction[J]. N Engl J Med, 2019, 381(21): 1995-2008.DOI:10.1056/NEJMoa1911303.
39. Schumm-Draeger PM, Burgess L, Korányi L, et al. Twice-daily dapagliflozin co-administered with metformin in type 2 diabetes: a 16-week randomized, placebo-controlled clinical trial[J]. Diabetes Obes Metab, 2015, 17(1): 42-51.DOI:10.1111/dom.12387.
40. Strojek K, Yoon KH, Hruba V, et al. Effect of dapagliflozin in patients with type 2 diabetes who have inadequate glycaemic control with glimepiride: a randomized, 24-week, double-blind, placebo-controlled trial[J]. Diabetes Obes Metab, 2011, 13(10): 928-938.DOI:10.1111/j.1463-1326.2011.01434.x.
41. Weber MA, Mansfield TA, Cain VA, et al. Blood pressure and glycaemic effects of dapagliflozin versus placebo in patients with type 2 diabetes on combination antihypertensive therapy: a randomised, double-blind, placebo-controlled, phase 3 study[J]. Lancet Diabetes Endocrinol, 2016, 4(3): 211-220.DOI:10.1016/s2213-8587(15)00417-9.
42. Wilding JP, Norwood P, T'Joen C, et al. A study of dapagliflozin in patients with type 2 diabetes receiving high doses of insulin plus insulin sensitizers: applicability of a novel insulin-independent treatment[J]. Diabetes Care, 2009, 32(9): 1656-1662.
43. Yang W, Ma J, Li Y, et al. Dapagliflozin as add-on therapy in Asian patients with type 2 diabetes inadequately controlled on insulin with or without oral antihyperglycemic drugs: A randomized controlled trial[J]. J Diabetes, 2018, 10(7): 589-599.DOI:10.1111/1753-0407.12634.

44. Barnett AH, Mithal A, Manassie J, et al. Efficacy and safety of empagliflozin added to existing antidiabetes treatment in patients with type 2 diabetes and chronic kidney disease: a randomised, double-blind, placebo-controlled trial[J]. Lancet Diabetes Endocrinol, 2014, 2(5): 369-384.DOI:10.1016/s2213-8587(13)70208-0.
45. Häring HU, Merker L, Seewaldt-Becker E, et al. Empagliflozin as add-on to metformin plus sulfonylurea in patients with type 2 diabetes: a 24-week, randomized, double-blind, placebo-controlled trial[J]. Diabetes Care, 2013, 36(11): 3396-3404.
46. Häring HU, Merker L, Seewaldt-Becker E, et al. Empagliflozin as add-on to metformin in patients with type 2 diabetes: a 24-week, randomized, double-blind, placebo-controlled trial[J]. Diabetes Care, 2014, 37(6): 1650-1659.DOI:10.2337/dc13-2105.
47. Hattori S. Anti-inflammatory effects of empagliflozin in patients with type 2 diabetes and insulin resistance[J]. Diabetol Metab Syndr, 2018, 10: 93.
48. Kario K, Okada K, Kato M, et al. 24-Hour Blood Pressure-Lowering Effect of an SGLT-2 Inhibitor in Patients with Diabetes and Uncontrolled Nocturnal Hypertension: Results from the Randomized, Placebo-Controlled SACRA Study[J]. Circulation, 2018, 139(18): 2089-2097.
49. Kadowaki T, Haneda M, Inagaki N, et al. Empagliflozin monotherapy in Japanese patients with type 2 diabetes mellitus: a randomized, 12-week, double-blind, placebo-controlled, phase II trial[J]. Adv Ther, 2014, 31(6): 621-638.DOI:10.1007/s12325-014-0126-8.
50. Kawamori R, Haneda M, Suzaki K, et al. Empagliflozin as add-on to linagliptin in a fixed-dose combination in Japanese patients with type 2 diabetes: Glycaemic efficacy and safety profile in a 52-week, randomized, placebo-controlled trial[J]. Diabetes Obes Metab, 2018, 20(9): 2200-2209.
51. Kovacs CS, Seshiah V, Swallow R, et al. Empagliflozin improves glycaemic and weight control as add-on therapy to pioglitazone or pioglitazone plus metformin in patients with type 2 diabetes: a 24-week, randomized, placebo-controlled trial[J]. Diabetes Obes Metab, 2014, 16(2): 147-158.DOI:10.1111/dom.12188.
52. Packer M, Anker SD, Butler J, et al. Cardiovascular and Renal Outcomes with Empagliflozin in Heart Failure[J]. N Engl J Med, 2020, 383(15): 1413-1424.DOI:10.1056/NEJMoa2022190.
53. Roden M, Weng J, Eilbracht J, et al. Empagliflozin monotherapy with sitagliptin as an active comparator in patients with type 2 diabetes: a randomised, double-blind, placebo-controlled, phase 3 trial[J]. Lancet Diabetes Endocrinol, 2013, 1(3): 208-219.DOI:10.1016/s2213-8587(13)70084-6.

54. Rosenstock J, Jelaska A, Frappin G, et al. Improved glucose control with weight loss, lower insulin doses, and no increased hypoglycemia with empagliflozin added to titrated multiple daily injections of insulin in obese inadequately controlled type 2 diabetes[J]. Diabetes Care, 2014, 37(7): 1815-1823.DOI:10.2337/dc13-3055.
55. Rosenstock J, Jelaska A, Zeller C, et al. Impact of empagliflozin added on to basal insulin in type 2 diabetes inadequately controlled on basal insulin: a 78-week randomized, double-blind, placebo-controlled trial[J]. Diabetes Obes Metab, 2015, 17(10): 936-948.
56. Ross S, Thamer C, Cescutti J, et al. Efficacy and safety of empagliflozin twice daily versus once daily in patients with type 2 diabetes inadequately controlled on metformin: a 16-week, randomized, placebo-controlled trial[J]. Diabetes Obes Metab, 2015, 17(7): 699-702.DOI:10.1111/dom.12469.
57. Dagogo-Jack S, Liu J, Eldor R, et al. Efficacy and safety of the addition of ertugliflozin in patients with type 2 diabetes mellitus inadequately controlled with metformin and sitagliptin: The VERTIS SITA2 placebo-controlled randomized study[J]. Diabetes Obes Metab, 2018, 20(3): 530-540.
58. Terra SG, Focht K, Davies M, et al. Phase III, efficacy and safety study of ertugliflozin monotherapy in people with type 2 diabetes mellitus inadequately controlled with diet and exercise alone[J]. Diabetes Obes Metab, 2017, 19(5): 721-728.DOI:10.1111/dom.12888.
59. Bando Y, Tohyama H, Aoki K, et al. Ipragliflozin lowers small, dense low-density lipoprotein cholesterol levels in Japanese patients with type 2 diabetes mellitus[J]. J Clin Transl Endocrinol, 2016, 6: 1-7.
60. Han KA, Chon S, Chung CH, et al. Efficacy and safety of ipragliflozin as an add-on therapy to sitagliptin and metformin in Korean patients with inadequately controlled type 2 diabetes mellitus: A randomized controlled trial[J]. Diabetes Obes Metab, 2018, 20(10): 2408-2415.
61. Ishihara H, Yamaguchi S, Nakao I, et al. Efficacy and safety of ipragliflozin as add-on therapy to insulin in Japanese patients with type 2 diabetes mellitus (IOLITE): a multi-centre, randomized, placebo-controlled, double-blind study[J]. Diabetes Obes Metab, 2016, 18(12): 1207-1216.
62. Kashiwagi A, Takahashi H, Ishikawa H, et al. A randomized, double-blind, placebo-controlled study on long-term efficacy and safety of ipragliflozin treatment in patients with type 2 diabetes mellitus and renal impairment: results of the long-term ASP1941 safety evaluation in patients with type 2 diabetes with renal impairment (LANTERN) study[J]. Diabetes Obes Metab, 2015, 17(2): 152-160.

63. Seino Y, Sasaki T, Fukatsu A, et al. Efficacy and safety of luseogliflozin added to insulin therapy in Japanese patients with type 2 diabetes: a multicenter, 52-week, clinical study with a 16-week, double-blind period and a 36-week, open-label period[J]. Curr Med Res Opin, 2018, 34(6): 981-994.DOI:10.1080/03007995.2018.1441816.
64. Buse JB, Garg SK, Rosenstock J, et al. Sotagliflozin in Combination With Optimized Insulin Therapy in Adults With Type 1 Diabetes: The North American inTandem1 Study[J]. Diabetes Care, 2018, 41(9): 1970-1980.
65. Kaku K, Watada H, Iwamoto Y, et al. Efficacy and safety of monotherapy with the novel sodium/glucose cotransporter-2 inhibitor tofogliflozin in Japanese patients with type 2 diabetes mellitus: a combined Phase 2 and 3 randomized, placebo-controlled, double-blind, parallel-group comparative study[J]. Cardiovasc Diabetol, 2014, 13: 65.
66. Terauchi Y, Tamura M, Senda M, et al. Efficacy and safety of tofogliflozin in Japanese patients with type 2 diabetes mellitus with inadequate glycaemic control on insulin therapy (J-STEP/INS): Results of a 16-week randomized, double-blind, placebo-controlled multicentre trial[J]. Diabetes Obes Metab, 2017, 19(10): 1397-1407.
67. Schernthaner G, Gross JL, Rosenstock J, Guarisco M, Fu M, Yee J, et al. Canagliflozin compared with sitagliptin for patients with type 2 diabetes who do not have adequate glycemic control with metformin plus sulfonylurea: a 52-week randomized trial[J]. Diabetes Care, 2013, 36(9): 2508-2515
68. Nauck MA, Del Prato S, Meier JJ, Durán-García S, Rohwedder K, Elze M, et al. Dapagliflozin versus glipizide as add-on therapy in patients with type 2 diabetes who have inadequate glycemic control with metformin: a randomized, 52-week, double-blind, active-controlled noninferiority trial[J]. Diabetes Care, 2011, 34(9): 2015-2022
69. Müller-Wieland D, Kellerer M, Cypryk K, Skripova D, Rohwedder K, Johnsson E, et al. Efficacy and safety of dapagliflozin or dapagliflozin plus saxagliptin versus glimepiride as add-on to metformin in patients with type 2 diabetes[J]. Diabetes Obes Metab, 2018, 20(11): 2598-2607
70. Cho KY, Nakamura A, Omori K, Takase T, Miya A, Manda N, et al. Effect of switching from pioglitazone to the sodium glucose co-transporter-2 inhibitor dapagliflozin on body weight and metabolism-related factors in patients with type 2 diabetes mellitus: An open-label, prospective, randomized, parallel-group comparison trial[J]. Diabetes Obes Metab, 2019, 21(3): 710-714

71. Scott R, Morgan J, Zimmer Z, Lam RLH, O'Neill EA, Kaufman KD, et al. A randomized clinical trial of the efficacy and safety of sitagliptin compared with dapagliflozin in patients with type 2 diabetes mellitus and mild renal insufficiency: The CompoSIT-R study[J]. Diabetes Obes Metab, 2018, 20(12): 2876-2884
72. Shimizu M, Suzuki K, Kato K, Jojima T, Iijima T, Murohisa T, et al. Evaluation of the effects of dapagliflozin, a sodium-glucose co-transporter-2 inhibitor, on hepatic steatosis and fibrosis using transient elastography in patients with type 2 diabetes and non-alcoholic fatty liver disease[J]. Diabetes Obes Metab, 2019, 21(2): 285-292.DOI:10.1111/dom.13520.
73. Hayashi T, Fukui T, Nakanishi N, Yamamoto S, Tomoyasu M, Osamura A, et al. Dapagliflozin decreases small dense low-density lipoprotein-cholesterol and increases high-density lipoprotein 2-cholesterol in patients with type 2 diabetes: comparison with sitagliptin[J]. Cardiovasc Diabetol, 2017, 16(1): 8
74. [13] Zinman B, Lachin JM, Inzucchi SE. Empagliflozin, Cardiovascular Outcomes, and Mortality in Type 2 Diabetes[J]. N Engl J Med, 2016, 374(11): 1094.DOI:10.1056/NEJMc1600827.
75. Araki E, Tanizawa Y, Tanaka Y, Taniguchi A, Koiwai K, Kim G, et al. Long-term treatment with empagliflozin as add-on to oral antidiabetes therapy in Japanese patients with type 2 diabetes mellitus[J]. Diabetes Obes Metab, 2015, 17(7): 665-674.DOI:10.1111/dom.12464.
76. Hollander P, Hill J, Johnson J, Wei Jiang Z, Golm G, Huyck S, et al. Results of VERTIS SU extension study: safety and efficacy of ertugliflozin treatment over 104 weeks compared to glimepiride in patients with type 2 diabetes mellitus inadequately controlled on metformin[J]. Curr Med Res Opin, 2019, 35(8): 1335-1343.DOI:10.1080/03007995.2019.1583450.
77. Tsurutani Y, Nakai K, Inoue K, Azuma K, Mukai S, Maruyama S, et al. Comparative study of the effects of ipragliflozin and sitagliptin on multiple metabolic variables in Japanese patients with type 2 diabetes: A multicentre, randomized, prospective, open-label, active-controlled study[J]. Diabetes Obes Metab, 2018, 20(11): 2675-2679.DOI:10.1111/dom.13421.
78. Takashima H, Yoshida Y, Nagura C, et al. Renoprotective effects of canagliflozin, a sodium glucose cotransporter 2 inhibitor, in type 2 diabetes patients with chronic kidney disease: A randomized open-label prospective trial[J]. Diab Vasc Dis Res, 2018, 15(5): 469-472.DOI:10.1177/1479164118782872.
79. Fioretto P, Del Prato S, Buse JB, et al. Efficacy and safety of dapagliflozin in patients with type 2 diabetes and moderate renal impairment (chronic kidney disease stage 3A): The DERIVE Study[J]. Diabetes Obes Metab, 2018, 20(11): 2532-2540.

80. Pollock C, Stefánsson B, Reyner D, et al. Albuminuria-lowering effect of dapagliflozin alone and in combination with saxagliptin and effect of dapagliflozin and saxagliptin on glycaemic control in patients with type 2 diabetes and chronic kidney disease (DELIGHT): a randomised, double-blind, placebo-controlled trial[J]. Lancet Diabetes Endocrinol, 2019, 7(6): 429-441.DOI:10.1016/s2213-8587(19)30086-5.
81. Grunberger G, Camp S, Johnson J, et al. Ertugliflozin in Patients with Stage 3 Chronic Kidney Disease and Type 2 Diabetes Mellitus: The VERTIS RENAL Randomized Study[J]. Diabetes Ther, 2018, 9(1): 49-66.
82. Haneda M, Seino Y, Inagaki N, et al. Influence of Renal Function on the 52-Week Efficacy and Safety of the Sodium Glucose Cotransporter 2 Inhibitor Luseogliflozin in Japanese Patients with Type 2 Diabetes Mellitus[J]. Clin Ther, 2016, 38(1): 66-88 e20.DOI:10.1016/j.clinthera.2015.10.025.
83. Yale JF, Bakris G, Cariou B, et al. Efficacy and safety of canagliflozin in subjects with type 2 diabetes and chronic kidney disease[J]. Diabetes Obes Metab, 2013, 15(5): 463-473.
84. Heerspink HJL, Stefánsson BV, Correa-Rotter R, et al. Dapagliflozin in Patients with Chronic Kidney Disease[J]. N Engl J Med, 2020, 383(15): 1436-1446.DOI:10.1056/NEJMoa2024816.
85. Bhatt DL, Szarek M, Pitt B, et al. Sotagliflozin in Patients with Diabetes and Chronic Kidney Disease[J]. N Engl J Med, 2021, 384(2): 129-139.DOI:10.1056/NEJMoa2030186.
86. Kohan DE, Fioretto P, Tang W, et al. Long-term study of patients with type 2 diabetes and moderate renal impairment shows that dapagliflozin reduces weight and blood pressure but does not improve glycemic control[J]. Kidney Int, 2014, 85(4): 962-971.
